# pH-Controlled isomerization kinetics of *ortho*-disubstituted benzamidines: *E*/*Z* isomerism and axial chirality

**DOI:** 10.3762/bjoc.21.120

**Published:** 2025-08-04

**Authors:** Ryota Kimura, Satoshi Ichikawa, Akira Katsuyama

**Affiliations:** 1 Faculty of Pharmaceutical Sciences, Hokkaido University, Kita-12, Nishi-6, Kita-ku, Sapporo 060-0812, Japan; 2 Center for Research and Education on Drug Discovery, Faculty of Pharmaceutical Sciences, Hokkaido University, Kita-12, Nishi-6, Kita-ku, Sapporo 060-0812, Japan

**Keywords:** atropisomer, conformation, isomerization, molecular switch, organobase

## Abstract

pH-Responsive molecular switches and motors are a class of organic molecules whose three-dimensional structure can be changed by acid–base stimuli. To date, pH-responsive molecular switches have been developed using various functional groups, but further advances require expanding the range of pH-responsive systems and discovering new molecular architectures. Here, we investigate the pH-responsive behavior of *ortho*-disubstituted benzamidine, which generates atropisomers and *E*/*Z* isomers. The amidine moiety allows modulation of the C–N and C–N/C–C rotational barriers by protonation, providing a novel approach to control the kinetics of isomerization via pH adjustment. The results showed that protonation of the amidine moiety significantly suppresses both C–N bond rotation and C–N/C–C concerted rotation, demonstrating the potential of *ortho*-disubstituted benzamidine derivatives as a novel pH-responsive molecular switch.

## Introduction

pH-Responsive molecular motors and switches are a class of functional organic molecules capable of reversible structural and electronic changes triggered by protonation and deprotonation [1–19]. This class of molecules has the capacity to regulate three-dimensional structures and motions of molecules through simple acid–base stimuli. This provides a high degree of control over their behavior, allowing for both tunability and predictability. Among the various types of pH-sensitive molecular switches, those that rely on isomerism caused by the rotation of molecular bonds are attracting attention because they can apply the most basic property of molecular conformation to molecular switches [19]. For example, hydrazone-based molecular switches undergo reversible *E*/*Z* isomerization around the C=N bond [1–7], with protonation significantly shifting the equilibrium. Beyond double-bond isomerization, pH stimuli have also been employed to modulate rotational barriers in axially chiral anilines [10–11] and a triazine molecule [9]. In particular, protonation of a remote basic site in N–C axially chiral anilines significantly increases the rotational barrier by attenuating resonance stabilization in the transition state. One important issue in this field is the development of molecules that can precisely control the range of pH to which they can respond. Furthermore, it is also important to discover new molecular skeletons, as there is a lack of variation in the substructures that act as pH-responsive molecular switches.

**Figure 1:**
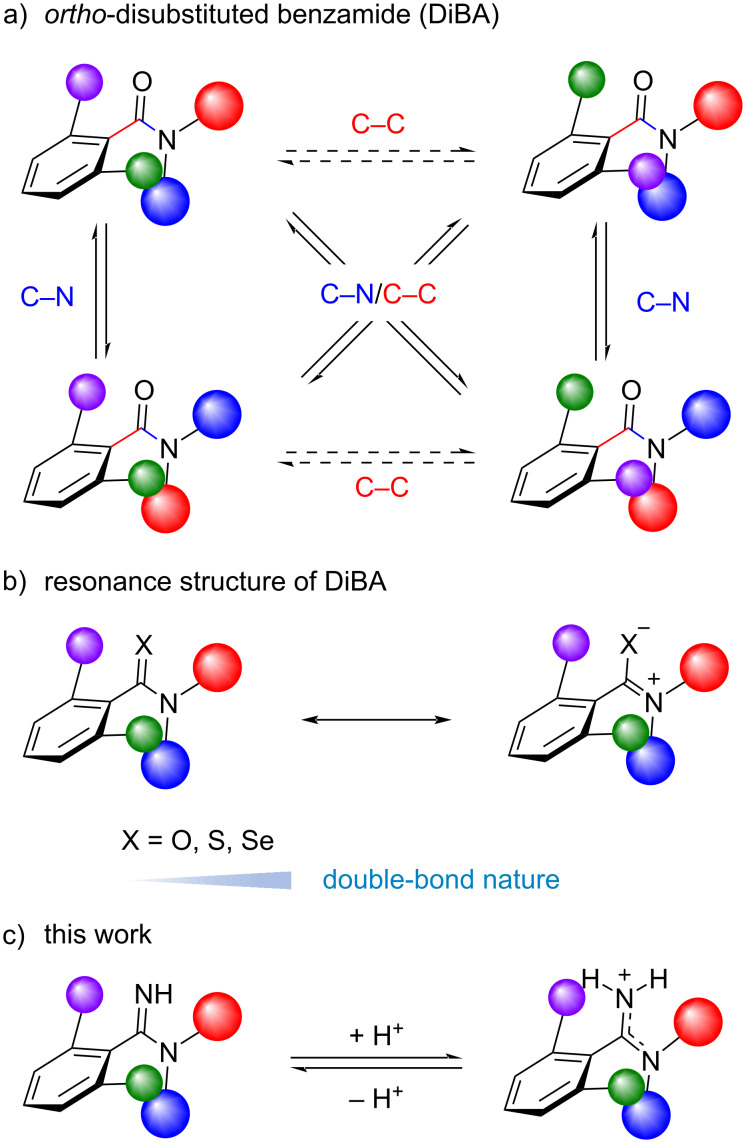
a) Structural features of DiBA. b) Resonance structure of the amide moiety of DiBA. c) Molecular form and protonated structure of *ortho*-disubstituted benzamidine.

Previously, we reported that chalcogen substitution of an *ortho*-disubstituted benzamide (DiBA) increases the rotational barrier of the C–N and C–N/C–C concerted rotation (Figure 1a,b) [20]. The observation can be explained mainly by the double-bond nature of the chalcogen amide C–N bond, which is attributed to a zwitterionic resonance structure of chalcogen amide. It has been shown that a late periodic chalcogen amide has a lower energy π* orbital (C=S or C=Se), resulting in an increase in the contribution of the zwitterionic resonance structure [21–23]. Based on this consideration, an *ortho*-disubstituted benzamidine, which is generated by formal substitution of the carbonyl oxygen atom of DiBA to an NH group, could act as a pH-responsive molecular switch (Figure 1c). Namely, the double-bond nature of an amidine moiety can be altered by the protonation of the amidine nitrogen atom. This suggests the possibility of controlling the rate of C–N rotation and C–N/C–C rotation by adjusting the pH of the solvent and the p*K*_a_ of the amidine. Herein, we report our findings on the structural properties of an *ortho*-disubstituted benzamidine. Similar to DiBA, an *ortho*-disubstituted benzamidine has two types of stereoisomers: *E*/*Z* isomers arising from the amidine C–N bond and atropisomers generated from the constrained C–C axis. Both C–N rotation and C–N/C–C rotation are suppressed by the protonation of the amidine moiety, and the rate of the isomerization can be controlled by the basicity of the amidine moiety.

## Results and Discussion

**Figure 2:**
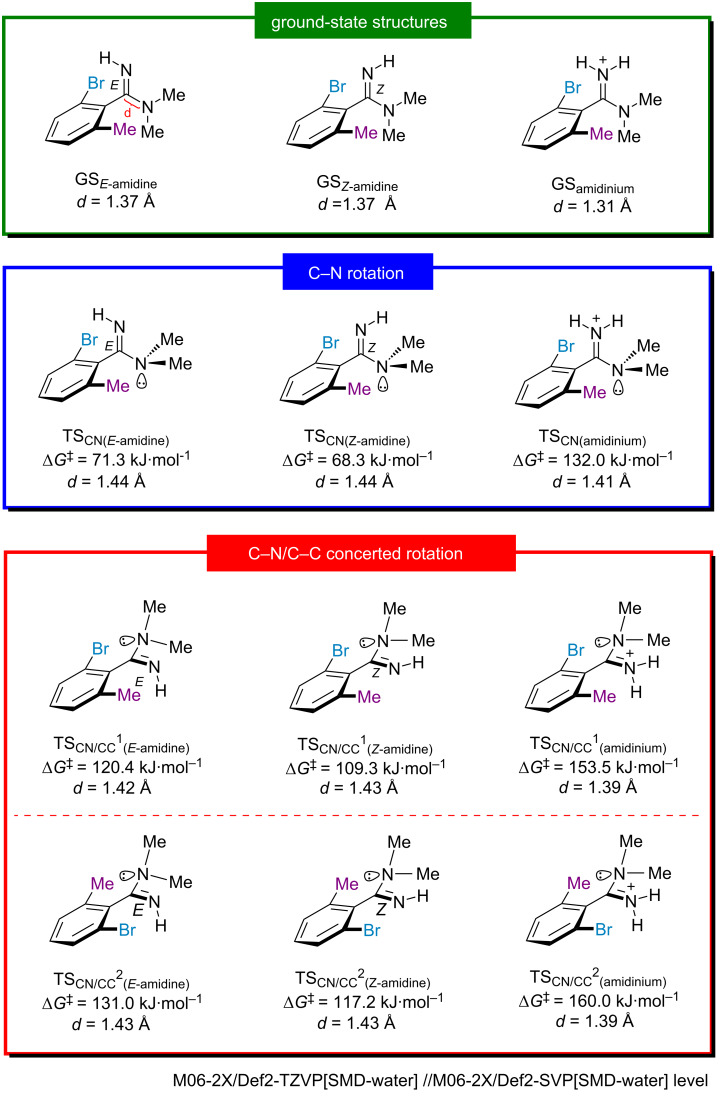
Rotational barriers of 2-bromo-*N*,*N*,6-trimethylbenzimidamide and its protonated form calculated by the DFT method.

To the best of our knowledge, although the structural analysis of the *E*/*Z* isomerism of amidine [24–25] and the effect of the protonation of an amidine moiety on the rotational barrier of the C–N bond [26] have been studied, the separation of amidine *E*/*Z* isomers has not been reported yet. To verify our hypothesis that the rotational barrier of the benzamidine changes upon protonation, density functional theory (DFT) calculations were performed for the C–N and C–N/C–C rotations of the molecular form and of the protonated 2-bromo-*N*,*N*,6-trimethylbenzimidamide as a model compound (Figure 2). Several transition states (TSs) were found depending on the configuration of the amidine N–H and the rotational direction. The C–N bond rotation of the molecular form of amidine was calculated to be 68 kJ·mol^−1^ for *Z*-amidine and 71 kJ·mol^−1^ for *E-*amidine. Protonation of the amidine moiety drastically increases the rotational barrier (132 kJ·mol^−1^), suggesting that the protonation suppresses the C–N bond rotation via the increased double-bond nature of the C–N axis. A similar trend was found for the C–N/C–C concerted rotation (Figure 2). Compared with the C–N bond rotation, the C–N/C–C bond rotation requires a higher rotational barrier as in the parent chalcogen amides, regardless of whether it is in the molecular form or protonated state, and the protonation of the nitrogen atom increases the activation energy by 33–43 kJ·mol^−1^. To investigate how protonation affects the double-bond nature of the amidine moiety, we calculated the distance between the imino carbon atom and the nitrogen atom of the NMe₂ moiety (*d*) for local minimum and transition-state structures (Figure 2). In the local minimum and transition state of the C–N rotation and C–N/C–C concerted rotation, protonation leads to a shortening of the C–N bond by 0.6 Å and 0.3–0.4 Å, respectively. This result indicates that protonation has a greater impact on the local minimum structures, as the p orbital of the nitrogen atom in the NMe₂ moiety and the C=N π orbital are located in the same plane, allowing for effective conjugation between them. The DFT study clearly showed that the rotational barriers of *ortho*-disubstituted benzamidine can be modulated by the protonation or deprotonation of the amidine moiety.

**Figure 3:**
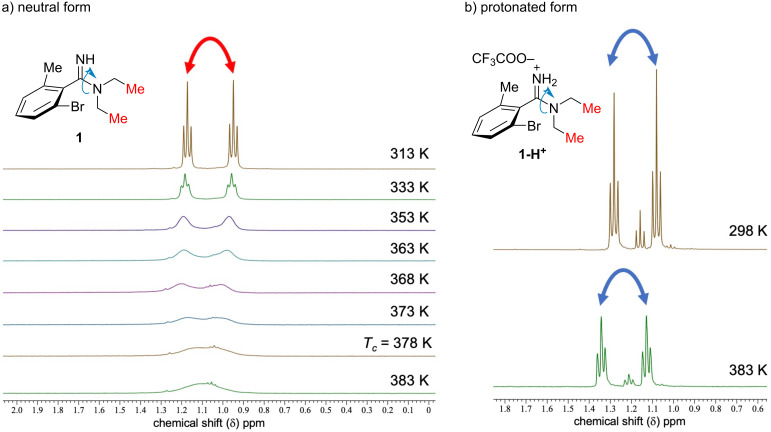
Comparison of VT-NMR spectra of a) amidine **1** and b) its trifluoroacetate salt **1-H^+^** in DMSO-*d*_6_ (400 MHz).

Next, we experimentally examined the C–N rotation of 2-bromo-*N*,*N*-diethyl-6-methylbenzimidamide (**1**). First, the effect of the protonation on the C–N bond rotation was investigated by variable temperature nuclear magnetic resonance (VT-NMR) spectra (Figure 3) in DMSO-*d*_6_ [27]. In the case of the molecular form of amidine **1** (Figure 3a), the signals corresponding to the two methyl groups resulting from the amidine *E*/*Z* isomerism were observed separately at 313 K (1.17 and 0.95 ppm), and they gradually fused as the temperature increased. The activation energy of *E*/*Z* isomerization was calculated to be 77 kJ·mol^−1^ from the observed coalescence temperature (*T*_c_ = 378 K). On the other hand, the two methyl signals of amidine **1** trifluoroacetate salt were not coalesced even at 383 K, indicating that the C–N bond rotation was sufficiently slow on the NMR time scale (Figure 3b).

**Figure 4:**
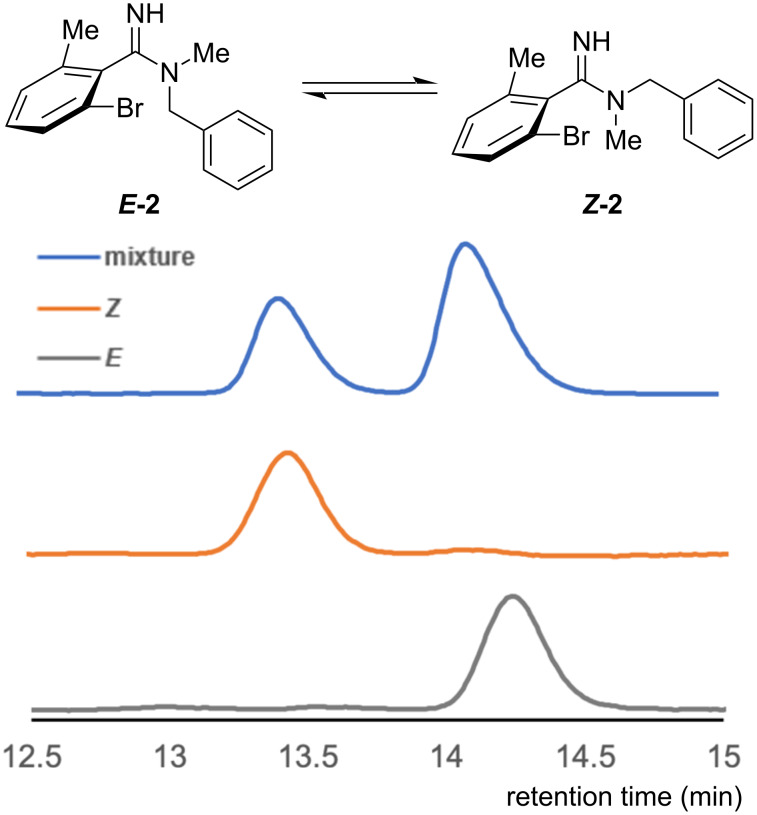
Separation and isolation of amidine *E*/*Z* isomers by RP-HPLC. The mobile phase contained CF_3_CO_2_H to protonate the amidine moiety.

To separate each *E*/*Z* isomer, amidine **2** with two different substituents on the same nitrogen atom, was prepared, and racemic **2** was analyzed by reversed-phase high performance liquid chromatography (RP-HPLC) using an acidic mobile phase [H_2_O (0.1% CF_3_CO_2_H)/MeCN 72:28]. As shown in Figure 4, the HPLC analysis of amidine **2** showed two peaks corresponding to the *E* and *Z* isomers which could be explained by the existence of *E*/*Z* isomers of amidine **2** trifluoroacetate salt. After separation of the two peaks, HPLC analysis of each separated component showed single peaks, suggesting that the *E* and *Z* isomers of amidine **2** trifluoroacetate salt could be separated by standard RP-HPLC. The configuration of the amidine moiety of each component was characterized by NOE experiments (for details, see Supporting Information File 1). The NMR and HPLC experiments clearly indicate that protonation of the amidine moiety increases the rotational barrier of the C–N bond, a result which is consistent with our DFT study.

**Figure 5:**
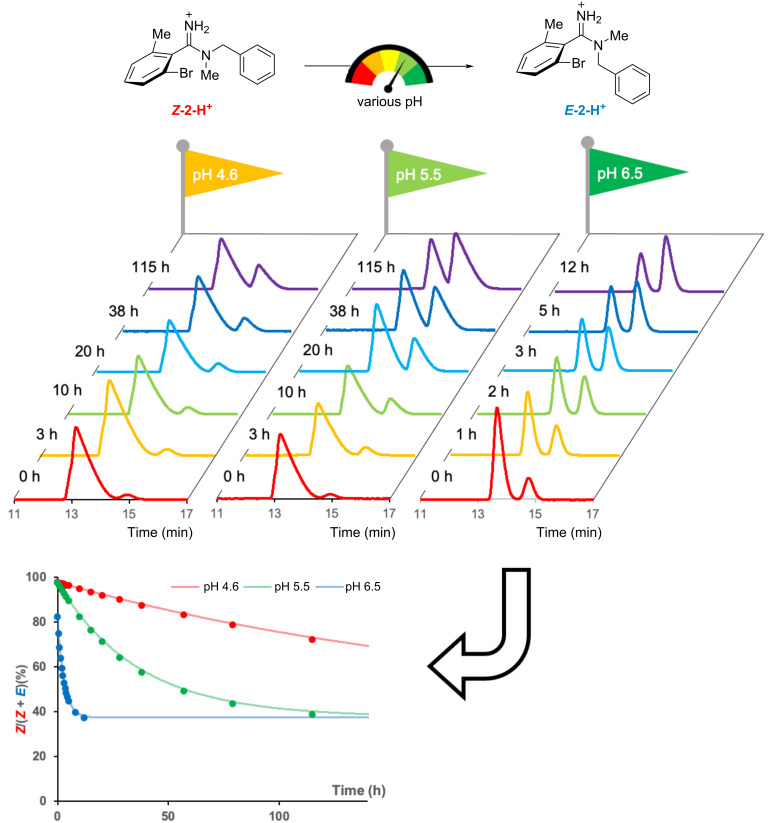
Kinetic analysis of the isomerization of ***Z*-2-H^+^** to ***E*-2-H^+^** at different pH (pH 4.6, 5.5, and 6.5). In the bottom graph, each circle represents the experimental ratio of the *Z* isomer at each time point, and each curve shows the theoretical value obtained from the curve-fitting analysis of the experimental data.

**Figure 6:**
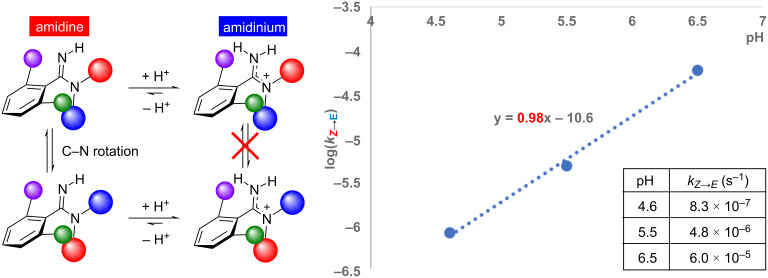
Correlation between *E*/*Z* isomerization rate constant and pH. The result indicates that C–N rotation from the molecular form of amidine was dominant in the observed isomerization.

Since it was found that the *E*/*Z* isomers of amidine could be isolated as a TFA salt, the kinetics of the C–N bond rotation at different pH values was then evaluated. The *E*/*Z* ratio of the isolated ***Z*-2** trifluoroacetate salt (***Z*-2-H^+^**) in three buffers (pH 4.6, 5.5, and 6.5, respectively) was calculated by RP-HPLC at different times at 20 °C (Figure 5). The curve fitting analysis of the obtained data points yielded an apparent first-order rate constant (*k*) of 8.3 × 10^−7^·s^−1^ at pH 4.6, 4.8 × 10^−6^·s^−1^ at pH 5.5, and 6.0 × 10^−5^·s^−1^ at pH 6.5. These results indicate that isomerization was suppressed as pH decreased, as expected. Plotting log(*k*) versus pH showed a linear relationship with a slope of 1 (Figure 6). This result means that a change of one unit in pH results in about a 10-fold change in the isomerization rate, considering the Henderson–Hasselbalch equation, where pH changes of one increases or decreases the amount of molecular form by a factor of ten. The experimental results, showing that the pH dependence of the isomerization rate closely matches that of the molecular form of the amidine fraction, suggest that the observed C–N isomerization mainly proceeds from the molecular form. These results suggest that the C–N bond rotation in amidines may be governed by the fraction of the molecular form species present at a given pH, reflecting the basicity of the amidine. Motivated by this finding, we next investigated the relationship between amidine basicity and the isomerization rate.

**Figure 7:**
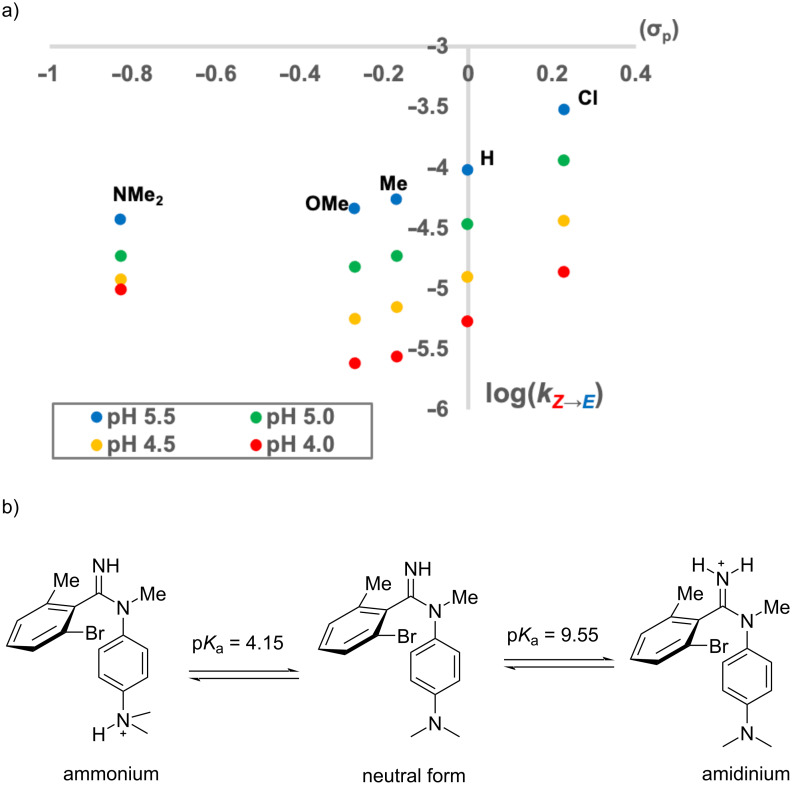
a) Correlation between isomerization rate constant and electronic effects of the substituents. b) Possible protonated states of compound **7**.

To systematically modulate the basicity of amidine, we designed *N*-phenylbenzimidamides **3**–**7**, with different electron-donating or electron-withdrawing substituents at the *para*-position of the phenyl ring conjugated with the amidine moiety. The same kinetic analysis was performed for compounds **3**–**7** at pH 4.0, 4.5, 5.0, and 5.5, and the results are summarized in Table 1. In addition, the calculated p*K*_a_ values of the amidines are also shown in Table 1. As a result, the C–N isomerization was accelerated by an electron-withdrawing chloro substituent, and electron-donating substituents (Me, OMe and NMe_2_ groups) decreased the isomerization rate. Figure 7a illustrates the effect of the electronic property of each substituent, which is represented as the Hammett substituent constant [28], on the rate constant at different pH. Consistent with the results for amidine **2**, amidines **3**–**7** showed smaller rate constants at more acidic pH, suggesting that isomerization of the molecular form species was also predominant for amidines **3**–**7**. The results at the same pH suggest that the isomerization rate varies systematically depending on the electron-donating or electron-withdrawing property of the substituent, indicating that the isomerization rate can be controlled by the basicity of the amidine. It is noteworthy that the pH dependence was relatively smaller in the case of amidine **7** (X = NMe_2_). This fact could be rationalized by the existence of different protonation states of amidine **7** (Figure 7b). In the case of amidine **7**, both the amidine moiety and the NMe_2_ group could be protonated under acidic conditions, and the NMe_2_-protonated species has a similar double-bond nature for the C–N bond as the molecular form. Thus, it can be assumed that the presence of the NMe_2_-protonated species reduced the apparent population of the protonated amidine under acidic conditions, resulting in a decrease in the inhibitory effect of the C–N rotation due to the decrease in pH.

**Table 1:** The p*K*_a_ value of each compound and rate constants of *E*/*Z* isomerization at various pH values.

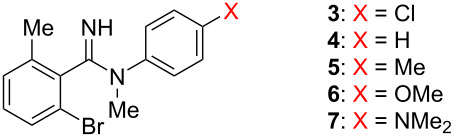				

compound	p*K*_a_^a^	pH	*k* (10^−6^·s^−1^)	
			*Z* to *E*	*E* to *Z*

**3**	9.36	4.0	14.0	20.6
		4.5	36.6	53.4
		5.0	115.0	159.8
		5.5	305.3	427.8

**4**	10.02	4.0	5.4	10.8
		4.5	12.7	24.5
		5.0	34.3	66.3
		5.5	97.7	186.2

**5**	10.31	4.0	2.8	4.6
		4.5	7.1	12.5
		5.0	18.8	32.4
		5.5	54.7	97.8

**6**	10.32	4.0	2.4	4.1
		4.5	5.6	10.5
		5.0	18.8	32.4
		5.5	47.0	85.8

**7**	10.82	4.0	10.1	15.1
		4.5	12.2	18.2
		5.0	18.7	28.1
		5.5	37.9	57.1

^a^Calculated by Jaguar program (Schrödinger, LLC, 2024).

**Figure 8:**
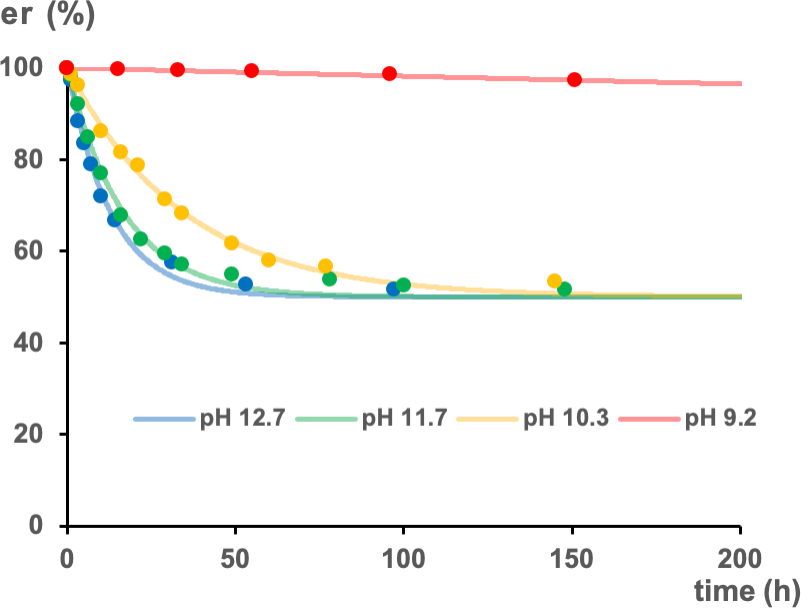
Analysis of the rate of racemization of **1** at various pH at 70 °C. Each circle shows the experimental er at each time point, and each curve shows the theoretical value obtained from the curve fitting analysis of the experimental data.

As the rotation of the C–N bond of the *ortho*-disubstituted benzamidine could be controlled by the protonation of the amidine moiety, we then focused on the chiral axis of the *ortho*-disubstituted benzamidine. Our DFT calculations suggested that the double-bond nature of the C–N bond in the transition state of the C–N/C–C concerted rotation was decreased due to the twisted structure, and the activation energy varies depending on whether the amidine was protonated or in its molecular form (Figure 2). To investigate the effect of protonation on the stability of the chiral axis, amidine **1**, which has identical substituents on the amidine nitrogen and thus does not generate *E*/*Z* isomers, was selected. In the chiral HPLC analysis using an acidic eluent [MeCN/H_2_O (containing 0.5% CF_3_CO_2_H) 20:80], the trifluoroacetate salt of amidine **1** was observed as two peaks corresponding to the atropisomers arising from the C–C axis. Each atropisomer could be separated by chiral HPLC, and the isomer eluting earlier was used for the following kinetic analysis. The effect of pH on the kinetics of the racemization was investigated. The isomer was heated in the buffer (pH 9.2, 10.3, 11.7, and 12.7) at 70 °C, and the er value was monitored at each time point (Figure 8). The rate constants obtained from the curve fitting analysis of the experimental results significantly changed in the examined pH range (Table 2), and the rate constants increased with rising pH. Notably, at pH 9.2, racemization proceeded minimally, even under high temperature conditions. Furthermore, it was confirmed that racemization requires a higher pH where a higher proportion of the amidine exists in its molecular form. This result was consistent with our DFT calculations, which showed that the rotational barrier of the C–N/C–C concerted rotation is higher than that of C–N rotation. This finding is consistent with the calculated p*K*_a_ of 10.6 for amidine **1**, indicating that racemization is minimal under the conditions where most of the amidine remains in its protonated form. As pH was increased above the p*K*_a_, the proportion of the molecular form of amidine increased, leading to a significant increase in the isomerization rate. These observations underscore the significant influence of pH on the racemization kinetics of amidine **1**, as shown in Table 1. As a result, it was found that as well as the C–N bond rotation the racemization rate could be controlled by the protonation of the basic amidine moiety. This result indicates the possibility of controlling the stability of the chiral axis by the basicity of the amidine, as well as the C–N bond, as mentioned above.

**Table 2:** Rate constants of racemization of **1** at various pH at 70 °C.

pH	*k*_rac_ (10^−6^·s^−1^)

9.2	0.05
10.3	4.0
11.7	8.3
12.7	10.7

## Conclusion

In conclusion, we demonstrated that the rate of the C–N bond rotation and racemization of *ortho*-disubstituted benzamidine could be controlled by the protonation of the amidine moiety and showed the potential for application as a pH-responsive molecular switch based on changes in the rotation rate of the two axes. For the C–N bond rotation, the basicity of the amidine moiety had a clear impact on the rate of bond rotation, and the finding could be helpful to the rational design of pH-responsive molecular switches based on the *ortho*-disubstituted benzamidine. The study presented here also shows the usefulness of the single-atom substitution strategy on DiBA. By replacing the oxygen atom of DiBA with a nitrogen atom (and thus NH), which has been shown to be photo-responsive when replaced with sulfur or selenium, the pH-responsive property was acquired. Such minimal changes in physical properties can be made with little effect on the molecular structure and are widely used in areas such as carbon materials. If the three-dimensional structure of molecules can be manipulated by various external stimuli through such modifications, we can expect to develop useful molecular switches that can be applied to various fields, such as functional materials, biological probes, and drugs.

## Supporting Information

File 1 Detailed experimental procedures, spectral data and HPLC charts.

## Data Availability

All data that supports the findings of this study is available in the published article and/or the supporting information of this article.
