# Supplementary material for: pH-Controlled isomerization kinetics of ortho-disubstituted benzamidines: E/Z isomerism and axial chirality
Source: Beilstein J Org Chem. 2025 Aug 4;21:1568–76. doi: 10.3762/bjoc.21.120 (PMC12337994; doi:10.3762/bjoc.21.120)
Supplement: File 1 — Detailed experimental procedures, spectral data and HPLC charts. [file Beilstein_J_Org_Chem-21-1568-s001.pdf]

BEILSTEIN JOURNAL OF ORGANIC CHEMISTRY

# Supporting Information

for

# pH-Controlled isomerization kinetics of *ortho*-disubstituted benzamidines: *E*/*Z* isomerism and axial chirality

Ryota Kimura, Satoshi Ichikawa and Akira Katsuyama

*Beilstein J. Org. Chem.* **2025,** *21,* 1568–1576. doi:10.3762/bjoc.21.120

# Detailed experimental procedures, spectral data and HPLC charts

License and Terms: This is a supporting information file under the terms of the Creative Commons Attribution License (https://creativecommons.org/licenses/by/4.0). Please note that the reuse, redistribution and reproduction in particular requires that the author(s) and source are credited and that individual graphics may be subject to special legal provisions.
The license is subject to the *Beilstein Journal of Organic Chemistry* terms and conditions: (https://www.beilstein-journals.org/bjoc/terms)

## Contents


1. **General experimental methods** **S3**

2. **Preparation of compounds** **S5**

3. **Kinetic study using vt-NMR method** **S9**

4. **Separation of the *E*/*Z* isomers** **S12**

5. **Monitoring of the *E*/*Z* isomerization** **S16**

6. **Monitoring of the racemization** **S41**

7. **Assignment of the *E*/*Z* configuration assignment** **S68**

8. **Quantum chemical calculations** **S69**

9. **NMR spectrums of the synthesized compounds** **S75**

10. **LR-MS and HR-MS charts of the synthesized compounds** **S89**

11. **Reference** **S103**

## 1. General experimental methods

All reactions were performed under argon atmosphere. The weight of the starting materials and the products were not calibrated. Materials were purchased from commercial suppliers and used without further purification, unless otherwise noted. Solvents are distilled according to the standard protocol. All reactions requiring heating were conducted by using SynFlex, unless otherwise noted. Analytical thin layer chromatography (TLC) was performed on Merck silica gel 60 F254 plates. Normal-phase column chromatography and the filtration was performed on Merck silica gel 60 (63-200 μm) or Kanto Chemical Silica Gel 60N (spherical, neutral, 63-210 μm). Hi-flash column chromatography was performed on YAMAZEN Hi-FlashTM column silica gel (30 μm).

$^{1}$H NMR were measured in DMSO-$d_6$ solution and reported in parts per million (ppm) referenced to residual solvent peaks of DMSO-$d_6$ (2.50 ppm) using JEOL ECS 400 (400 MHz), JEOL ECX 400P (400 MHz), or JEOL ECZ 400 (400 MHz) spectrophotometers. $^{13}$C NMR were measured in DMSO-$d_6$ solution and referenced to residual solvent peaks of DMSO-$d_6$ (39.52 ppm) using JEOL ECS 400 (100 MHz), JEOL ECX 400P (100 MHz) or JEOL ECZ 400 (100 MHz) spectrophotometers. Coupling constant ($J$) was reported in hertz (Hz). Abbreviations of multiplicity were as follows; s: singlet, d; doublet, t: triplet, q: quartet, m: multiplet. Data were presented as follows; chemical shift (multiplicity, integration, coupling constant). Assignment was based on $^{1}$H– $^{1}$H COSY NMR spectra and NOE spectra.

Mass spectra were obtained on Advion expression CMS or Thermo Scientific Exactive Plus.The mass analyzer type used for the HRMS measurements was TOF. Optical rotations were determined on Rudolph Research Analytical Autopol IV automatic polarimeter.

High performance liquid chromatography (HPLC) was performed on one of the following systems: (1) JASCO PU-2086 Plus as a pomp, JASCO HV-2088-06 as a flow line selection valve, JASCO MD-2018 Plus as a detector, JASCO FC-2088-30 as a fraction collector controller, ADVANTECCHF122SC as a fraction collector, JASCO LC-NetII/ADC as a hardware interface, and JASCO ChromNAV as a system controller; (2) JASCO PU-2086 Plus as a pomp, JASCO UV-2075 Plus as a detector, JASCO FC-2088-30 as a fraction collector controller, ADVANTEC CHF122SC as a fraction collector, JASCO LC-NetII/ADC as a hardware interface, and JASCO ChromNAV as a system controller; (3) JASCO PU-4086-Binary as a pomp, JASCO MD-4015 as a photo diode array detector, JASCO FCC as a fraction collector controller, ADVANTEC CHF122SC as a fraction collector, JASCO LC-NetII/ADC as a hardware interface, JASCO AS-4058 as a preparative autosampler and JASCO ChromNAV as a system controller; (4) JASCO PU-4086-Binary as a pomp, JASCO UV4075 as a detector, JASCO FCC as a fraction collector controller, ADVANTEC CHF122SC as a fraction collector, JASCO LC-NetII/ADC as a hardware interface, JASCO AS-4058 as a preparative autosampler and JASCO ChromNAV as a system controller.

Liquid chromatography-mass spectrometry (LC-MS) was performed on either of the following systems: (1) Shimadzu LC-2050C as a HPLC system, FCV-0206 as a flow-line selection valve, LCMS-2050 as a Liquid chromatograph mass spectrometer, and LabSolutions as a system controller; (2) Shimadzu Prominence-i LC-2030C Plus as a HPLC system, FCV-20AH$_2$ as a flow-line selection valve, LCMS-8040 as a Liquid chromatograph mass spectrometer, and LabSolutions as a system controller.

Buffer solutions at pH 12.7 and 11.7 were prepared using aqueous solutions of $Na_3PO_4$ and $Na_2HPO_4$. A buffer solution at pH 10.3 was prepared using aqueous solutions of $Na_2CO_3$ and $NaHCO_3$. A buffer solution at pH 9.2 was prepared using aqueous solutions of $NH_3$ and $NH_4Cl$. A buffer solution at pH 6.5 was prepared using aqueous solutions of $Na_2HPO_4$ and $NaH_2PO_4$. Buffer solutions at pH 5.5, 5.0, 4.6, 4.5, and 4.0 were prepared using aqueous solutions of $CH_3COONa$ and $CH_3COOH$.

## 2. Preparation of compounds

All *ortho*-disubstituted benzamidines were synthesized in two steps from 2-methylbenzonitrile. 2-Bromo-6-methylbenzonitrile is a known compound reported in ref. 1.

**Scheme S2.1**. Synthesis of the *ortho*-disubstituted benzamidines

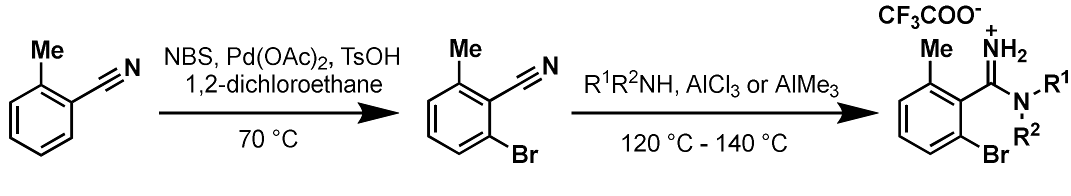


**2-Bromo-*N*,*N*-diethyl-6-methylbenzimidamide (1) trifluoroacetate salt**

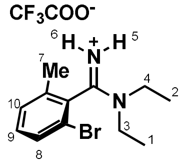

A mixture of 2-bromo-6-methylbenzonitrile (196 mg, 1.0 mmol) and diethylamine (2.19 g, 30 mmol) was treated with $AlCl_3$ (760 mg, 5.0 mmol) at 120 °C for 1 h under microwave irradiation. The reaction was quenched with 1 M *aq*. NaOH (5 mL), and the mixture was partitioned between $CH_2Cl_2$ and 1 M *aq*. NaOH. The aqueous phase was extracted with $CH_2Cl_2$. The organic phase was dried ($Na_2SO_4$), filtered, and concentrated *in vacuo*. The residue was purified by ODS silica gel column chromatography (φ 3.5 cm × 10 cm, 30-60-100% MeCN (+0.1% TFA)/$H_2O$ (+0.1% TFA)) to afford **1** (332 mg, 0.87 mmol, 87%) as a trifluoroacetate salt as a yellow oil.

$^1$H NMR (DMSO-$d_6$, 400 MHz) δ 9.62 (s, 1H, H-5), 9.35 (s, 1H, H-6), 7.71-7.67 (m, 1H, H-8), 7.49-7.47 (m, 2H, H-9, H-10), 3.73 (dq, 1H, H-4, $J_{gem}$ = 14.4, $J_{4,2}$ = 7.2 Hz), 3.66 (dq, 1H, H-4, $J_{gem}$ = 14.4, $J_{4,2}$ = 7.2 Hz), 3.21 (dq, 1H, H-3, $J_{gem}$ = 14.4, $J_{3,1}$ = 7.2 Hz), 3.16 (dq, 1H, H-3, $J_{gem}$ = 14.4, $J_{3,1}$ = 7.2 Hz), 2.29 (s, 3H, H-7), 1.28 (dd, 3H, H-2, $J_{2,4}$ = $J_{2,4}$ = 7.2 Hz), 1.08 (dd, 3H, H-1, $J_{1,3}$ = $J_{1,3}$ = 7.2 Hz); $^{13}$C NMR (DMSO-$d_6$, 100 MHz) δ 161.2, 158.2, 157.9, 137.8, 132.8, 130.5, 130.4, 130.1, 120.0, 117.9, 114.9, 46.2, 42.3, 18.9, 12.6, 10.6; ESIMS-LR *m/z* 269.1 [$(M+H)^+$]; ESIMS-HR calcd. for $C_{12}H_{18}N_2Br$ 269.0648, found 269.0648.

***N*-Benzyl-2-bromo-*N*,6-dimethylbenzimidamide (2) trifluoroacetate salt**

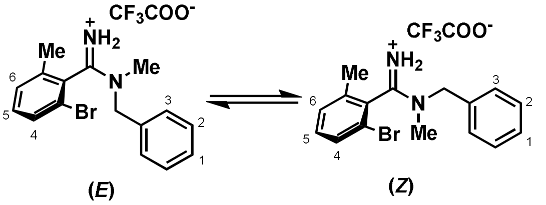


A mixture of 2-bromo-6-methylbenzonitrile (392 mg, 2.0 mmol) and N-methylbenzylamine (266 mg, 2.2 mmol) was treated with AlCl3 (266 mg, 2.0 mmol) at 140 °C for 15 h. The reaction was quenched with 1 M aq. NaOH (5 mL), and the mixture was partitioned between CH2Cl2 and 1 M aq. NaOH. The aqueous phase was extracted with CH2Cl2. The organic phase was dried (Na2SO4), filtered, and concentrated in vacuo. The residue was purified by ODS silica gel column chromatography (φ 3.5 cm × 10 cm, 30-60-100% MeCN (+0.1% TFA)/H2O (+0.1% TFA)) to afford 2 (239 mg, 0.75 mmol, 38%) as a trifluoroacetate salt as a yellow oil.

$^1$H NMR (DMSO-$d_6$, 400 MHz, a mixture of *E*/*Z* isomers). Selected data for ***Z*-2**: δ 9.95 (s, 1H, *N*-H), 9.84 (s, 1H, *N*-H), 7.70 (d, 1H, H-4, $J_{4,5}$ = 9.0 Hz), 7.51-7.41 (m, 5H, H-1, H-2, H-5, H-6), 7.39-3.31 (m, 2H, H-3), 5.03 (d, 1H,

*N*-C$H_2$Ph, $J_{gem}$ = 15.5 Hz), 4.88 (d, 1H, *N*-C$H_2$Ph, $J_{gem}$ = 15.5 Hz), 2.80 (s, 3H, *N*-Me), 2.30 (s, 3H, *C*-Me). Selected data for ***E*-2**: δ 9.90 (s, 1H, *N*-H), 9.54 (s, 1H, *N*-H), 7.71 (d, 1H, H-4, $J_{4,5}$ = 7.2 Hz), 7.51-7.41 (m, 3H, H-1, H-5, H-6), 7.39-3.31 (m, 2H, H-2), 7.16 (dd, 2H, H-3 $J_{3,2}$ = 7.4, $J_{3,1}$ = 2.0 Hz), 4.48 (d, 1H, *N*-C$H_2$Ph, $J_{gem}$ = 15.5 Hz), 4.42 (d, 1H, *N*-C$H_2$Ph, $J_{gem}$ = 15.5 Hz), 3.27 (s, 3H, *N*-Me), 2.20 (s, 3H, *C*-Me); $^{13}$C NMR (DMSO-$d_6$, 100 MHz, a mixture of *E*/*Z* isomers) δ 163.2, 163.0, 158.2, 157.8, 138.6, 137.7, 133.7, 133.3, 133.0, 132.9, 130.6, 130.6, 130.2, 130.1, 130.0, 129.0, 128.8, 128.5, 128.4, 128.2, 120.3, 119.6, 118.0, 115.1, 56.1, 53.2, 38.1, 36.9, 19.1, 18.9; ESIMS-LR *m*/*z* 317.1 [(M+H)$^+$]; ESIMS-HR calcd. for $C_{16}H_{18}N_2Br$ 317.0648, found 317.0641.

**2-Bromo-*N*-(4-chlorophenyl)-*N*,6-dimethylbenzimidamide (3) trifluoroacetate salt**

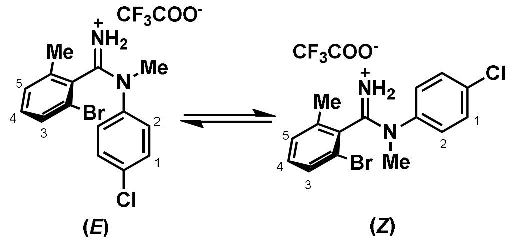


A mixture of 2-bromo-6-methylbenzonitrile (196 mg, 1.0 mmol) and 4-chloro-*N*-methylaniline (156 mg, 1.1 mmol) was treated with $AlCl_3$ (133 mg, 1.0 mmol) at 140 °C for 3.5 h. The reaction was quenched with 1 M *aq*. NaOH (5 mL), and the mixture was partitioned between $CH_2Cl_2$ and 1 M *aq*. NaOH. The aqueous phase was extracted with $CH_2Cl_2$. The organic phase was dried ($Na_2SO_4$), filtered, and concentrated *in vacuo*. The residue was purified by ODS silica gel column chromatography (φ 3.5 cm × 10 cm, 30-60-100% MeCN (+0.1% TFA)/ $H_2O$ (+0.1% TFA)) to afford **3** (388 mg, 0.86 mmol, 86%) as a trifluoroacetate salt as a brown oil.

$^1$H NMR (DMSO-$d_6$, 400 MHz, a mixture of *E*/*Z* isomers). Selected data for ***Z*-3**: δ 10.03 (s, 1H, *N*-H), 9.10 (s, 1H, *N*-H), 7.79-7.73 (m, 3H, Ar), 7.67-7.63 (m, 2H, Ar), 7.56-7.51 (m, 2H, Ar), 3.17 (s, 3H, *N*-Me), 2.46 (s, 3H, *C*-Me). Selected data for ***E*-3**: δ 10.37 (s, 1H, *N*-H), 10.01 (s, 1H, *N*-H), 7.50-7.45 (m, 1H, Ar), 7.41 (s, 4H, Ar), 7.30-7.25 (m, 2H, Ar), 3.64 (s, 3H, *N*-Me), 2.36 (s, 3H, *C*-Me); $^{13}$C NMR (DMSO-$d_6$, 100 MHz, a mixture of *E*/*Z* isomers) δ 163.9, 163.1, 158.1, 157.8, 140.4, 138.3, 138.1, 137.9, 134.9, 133.6, 133.2, 132.7, 131.2, 130.5, 130.4, 130.2, 130.1, 129.7, 129.6, 129.3, 128.1, 127.2, 120.3, 119.8, 118.1, 115.1, 42.0, 41.2, 19.6, 19.0; ESIMS-LR *m*/*z* 337.0 [(M+H)$^+$] ; ESIMS-HR calcd. for $C_{15}H_{15}N_2BrCl$ 337.0102, found 337.0097.

**2-Bromo-*N*,6-dimethyl-*N*-phenylbenzimidamide (4) trifluoroacetate salt**

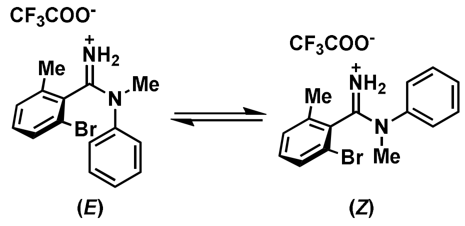


A mixture of 2-bromo-6-methylbenzonitrile (196 mg, 1.0 mmol) and *N*-methylaniline (118 mg, 1.1 mmol) was treated with $AlCl_3$ (133 mg, 1.0 mmol) at 140 °C for 16 h. The reaction was quenched with 1 M *aq*. NaOH (5 mL), and the mixture was partitioned between $CH_2Cl_2$ and 1 M *aq*. NaOH. The aqueous phase was extracted with $CH_2Cl_2$. The organic phase was dried ($Na_2SO_4$), filtered, and concentrated *in vacuo*. The residue was purified by ODS silica gel column chromatography (φ 3.5 cm × 10 cm, 30-60-100% MeCN (+0.1% TFA)/$H_2O$ (+0.1% TFA)) to afford **4** (376 mg, 0.90 mmol, 90%) as a trifluoroacetate salt as a black oil.

$^1$H NMR (DMSO-$d_6$, 400 MHz, a mixture of *E*/*Z* isomers). Selected data for ***Z*-4**: δ 9.94 (s, 1H, *N*-H), 8.96 (s, 1H, *N*-H), 7.78-7.21 (m, 8H, Ar), 3.18 (s, 3H, *N*-Me), 2.48 (s, 3H, *C*-Me). Selected data for ***E*-4**: δ 10.27 (s, 1H, *N*-H),

9.90 (s, 1H, *N*-H), 7.78-7.21 (m, 8H, Ar), 3.66 (s, 3H, *N*-Me), 2.36 (s, 3H, *C*-Me); $^{13}$C NMR (DMSO-$d_6$, 100 MHz, a mixture of *E*/*Z* isomers) δ 163.5, 163.0, 158.2, 157.9, 141.6, 139.1, 138.2, 138.0, 133.1, 132.6, 131.2, 130.7, 130.5, 130.3, 130.0, 129.8, 129.6, 129.3, 129.2, 125.8, 125.1, 120.5, 119.9, 117.8, 114.9, 42.2, 41.3, 19.7, 19.0; ESIMS-LR *m*/*z* 303.1 [(M+H)$^+$]; ESIMS-HR calcd. for $C_{15}H_{16}N_2Br$ 303.0491, found 303.0488.

**2-Bromo-*N*,6-dimethyl-*N*-(4-tolyl)benzimidamide (5) trifluoroacetate salt**

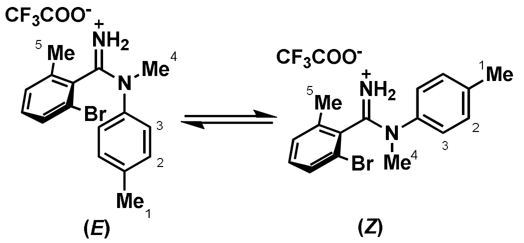


A mixture of 2-bromo-6-methylbenzonitrile (196 mg, 1.0 mmol) and *N*,4-dimethylaniline (133 mg, 1.1 mmol) was treated with $AlCl_3$ (133 mg, 1.0 mmol) at 140 °C for 6 h. The reaction was quenched with 1 M *aq*. NaOH (5 mL), and the mixture was partitioned between $CH_2Cl_2$ and 1 M *aq*. NaOH. The aqueous phase was extracted with $CH_2Cl_2$. The organic phase was dried ($Na_2SO_4$), filtered, and concentrated *in vacuo*. The residue was purified by ODS silica gel column chromatography (φ 3.5 cm × 10 cm, 30-60-100% MeCN (+0.1% TFA)/$H_2O$ (+0.1% TFA)) to afford **5** (163 mg, 0.76 mmol, 76%) as a trifluoroacetate salt as a brown oil.

$^1$H NMR (DMSO-$d_6$, 400 MHz, a mixture of *E*/*Z* isomers). Selected data for ***Z*-5**: δ 10.22 (s, 1H, *N*-H), 9.86 (s, 1H, *N*-H), 7.76-7.74 (m, 1H, Ar), 7.57-7.44 (m, 4H, Ar), 7.30-7.22 (m, 2H, Ar), 3.15 (s, 3H, H-4), 2.46 (s, 3H, H-1), 2.42 (s, 3H, H-5). Selected data for ***E*-5**: δ 9.90 (s, 1H, *N*-H), 8.90 (s, 1H, *N*-H), 7.57-7.44 (m, 2H, Ar) 7.30-7.22 (m, 3H, Ar), 7.11 (d, 2H, H-2, $J_{2,3}$ = 8.2 Hz), 3.62 (s, 3H, H-4), 2.35 (s, 3H, H-5), 2.20 (s, 3H, H-1); $^{13}$C NMR (DMSO-$d_6$, 100 MHz, a mixture of *E*/*Z* isomers) δ 163.6, 163.0, 158.2, 157.9, 139.9, 139.3, 138.8, 138.1, 138.0, 136.6, 133.1, 132.5, 131.6, 130.8, 130.5, 130.2, 130.1, 129.8, 129.7 129.6, 125.5, 124.8, 120.5, 119.9, 117.9, 114.9, 42.2, 41.4, 20.8, 20.5, 19.7, 19.0; ESIMS-LR *m*/*z* 317.1 [(M+H)$^+$]; ESIMS-HR calcd. for $C_{16}H_{18}$ $N_2Br$ 317.0648, found 317.0644.

**2-Bromo-*N*-(4-methoxyphenyl)-*N*,6-dimethylbenzimidamide (6) trifluoroacetate salt**

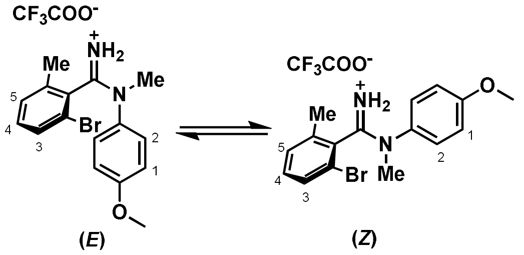


A mixture of 2-bromo-6-methylbenzonitrile (196 mg, 1.0 mmol) and 4-methoxy-*N*-methylaniline (151 mg, 1.1 mmol) was treated with $AlMe_3$ (1.4 M in hexane, 2 mL, 2.8 mmol) at 140 °C for 3 h. The reaction was quenched with 1 M *aq*. NaOH (5 mL), and the mixture was partitioned between $CH_2Cl_2$ and 1 M *aq*. NaOH. The aqueous phase was extracted with $CH_2Cl_2$. The organic phase was dried ($Na_2SO_4$), filtered, and concentrated *in vacuo*. The residue was purified by ODS silica gel column chromatography (φ 3.5 cm × 10 cm, 30-60-100% MeCN (+0.1% TFA)/$H_2O$ (+0.1% TFA)) to afford **6** (140 mg, 0.31 mmol, 31%) as a trifluoroacetate salt as a brown oil.

$^1$H NMR (DMSO-$d_6$, 400 MHz, a mixture of *E*/*Z* isomers). Selected data for ***Z*-6**: δ 9.87 (s, 1H, *N*-H), 8.88 (s, 1H, *N*-H), 7.75(dd, 1H, H-3, $J_{3,5}$ = 1.8, $J_{3,4}$ = 7.2 Hz), 7.53 (dd, 1H, H-4, $J_{4,3}$ = $J_{4,5}$ = 7.2 Hz), 7.51 (dd, 1H, H-5, $J_{5,3}$ = 1.8 Hz, $J_{5,4}$ = 7.2 Hz), 7.51 (d, 2H, H-2, $J_{2,1}$ = 9.0 Hz), 7.22 (d, 2H, H-1, $J_{1,2}$ = 9.0 Hz), 3.85 (s, 3H, *O*-Me), 3.14 (s, 3H, *N*-Me), 2.46 (s, 3H, *C*-Me). Selected data for ***E*-6**: δ 10.18 (s, 1H, *N*-H), 9.81 (s, 1H, *N*-H), 7.46 (dd, 1H, H-3, $J_{3,5}$ =

2.5, $J_{3,4}$ = 6.5 Hz), 7.32 (d, 2H, H-2, $J_{2,1}$ = 9.0 Hz). 7.27-7.24 (m, 2H, Ar), 6.84 (d, 2H, H-1, $J_{1,2}$ = 9.0 Hz), 3.67 (s, 3H, *O*-Me), 3.61 (s, 3H, *N*-Me), 2.35 (s, 3H, *C*-Me); $^{13}$C NMR (DMSO-$d_6$, 100 MHz, a mixture of *E*/*Z* isomers) δ 163.8, 163.2, 160.1, 159.2, 158.3, 158.0, 138.0, 133.1, 132.5, 131.6, 130.5, 130.2, 130.1, 129.9, 129.6, 127.1, 126.3, 120.4, 119.9, 116.2, 114.3, 55.7, 55.4, 42.3, 41.6, 19.7, 19.0; ESIMS-LR *m*/*z* 333.1 [(M+H)$^+$]; ESIMS-HR calcd. for $C_{16}H_{18}ON_2Br$ 333.0597, found 333.0592.

**2-Bromo-*N*-(4-(dimethylamino)phenyl)-*N*,6-dimethylbenzimidamide (7) trifluoroacetate salt**

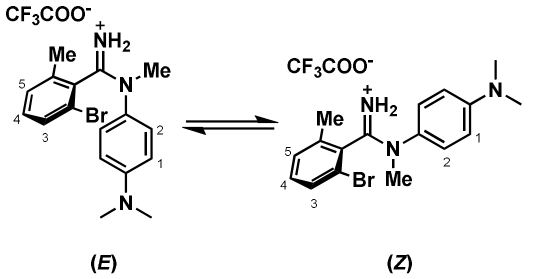


A mixture of 2-bromo-6-methylbenzonitrile (196 mg, 1.0 mmol) and $N^1$,$N^1$,$N^4$-trimethylbenzene-1,4-diamine (165 mg, 1.1 mmol) was treated with $AlCl_3$ (133 mg, 1.0 mmol) at 140 °C for 14 h. The reaction was quenched with 1 M *aq*. NaOH (5 mL), and the mixture was partitioned between $CH_2Cl_2$ and 1 M *aq*. NaOH. The aqueous phase was extracted with $CH_2Cl_2$. The organic phase was dried ($Na_2SO_4$), filtered, and concentrated *in vacuo*. The residue was purified by ODS silica gel column chromatography (φ 3.5 cm × 10 cm, 30-60-100% MeCN (+0.1% TFA)/$H_2O$ (+0.1% TFA)) to afford **7** (269 mg, 0.58 mmol, 58%) as a trifluoroacetate salt as a purple oil.

$^1$H NMR (DMSO-$d_6$, 400 MHz, a mixture of *E*/*Z* isomers). Selected data for ***Z*-7**: δ 9.77 (s, 1H, *N*-H), 8.76 (s, 1H, *N*-H), 7.74 (dd, 1H, H-3, $J_{vic}$ = 7.2, $J_{vic}$ = 1.8 Hz), 7.54-7.51 (m, 2H, H-4, H-5), 7.34 (d, 2H, H-2, $J_{2,1}$ = 9.0 Hz), 6.92 (d, 2H, H-1, $J_{1,2}$ = 9.0 Hz), 3.11 (s, 3H, *N*-Me), 3.00 (s, 6H, *N*-Me$_2$), 2.45 (s, 3H, *C*-Me). Selected data for ***E*-7**: δ 10.04 (s, 1H, *N*-H), 9.68 (s, 1H, *N*-H), 7.49-7.46 (m, 1H, H-3), 7.25-7.22 (m, 2H, H-4, H-5), 7.17 (d, 2H, H-2, $J_{2,1}$ = 9.2 Hz), 6.53 (d, 2H, H-1, $J_{1,2}$ = 9.2 Hz), 3.57 (s, 3H, *N*-Me), 2.82 (s, 6H, *N*-Me$_2$), 2.34 (s, 3H, *C*-Me); $^{13}$C NMR (DMSO-$d_6$, 100 MHz, a mixture of *E*/*Z* isomers) δ 163.7, 163.1, 158.4, 158.0, 150.9, 149.9, 137.9, 133.0, 132.3, 131.2, 130.5, 130.2, 130.2, 130.0, 129.5, 127.0, 126.0, 125.3, 120.5, 119.9, 117.3, 114.4, 113.5, 111.5, 42.4, 41.7, 19.7, 19.0; ESIMS-LR *m*/*z* 346.1 [(M+H)$^+$]; ESIMS-HR calcd. for $C_{17}H_{21}N_3Br$ 346.0913, found 346.0907.

## 3. Kinetic study using vt-NMR method

Variable-temperature (vt) $^1$H NMR experiments were carried out to prove that protonated amidine has a higher activation energy of *E*/*Z* isomerization than neutral form of amidine. DMSO-$d_6$ was used as the solvent. In the case of neutral form, spectrum varied drastically with temperature. Then, focusing on the peaks of the two terminal methyl groups, the activation energy of *E*/*Z* isomerization was determined using the following equation (eq. 1). The coalescence temperature was 378 K and the difference in chemical shifts at 298 K was 88.4 Hz. From these values, the activation energy in DMSO-$d_6$ was determined to be 76.6 kJ·mol$^{-1}$. On the other hand, spectrum of protonated amidine showed littile change with temperature.

$$\Delta G^{\ddagger} = RT_c\left[22.96 + \ln\left(\frac{T_c}{\Delta\nu}\right)\right] \quad \text{(eq. 1)}$$

$T_c$ = coalescence temperature (K), $\Delta\nu$ = difference in chemical shifts in hertz ($\delta_A$ - $\delta_B$)
$R$ = 8.314 J·K$^{-1}$ mol$^{-1}$, $\Delta G^{\ddagger}$ = activation energy

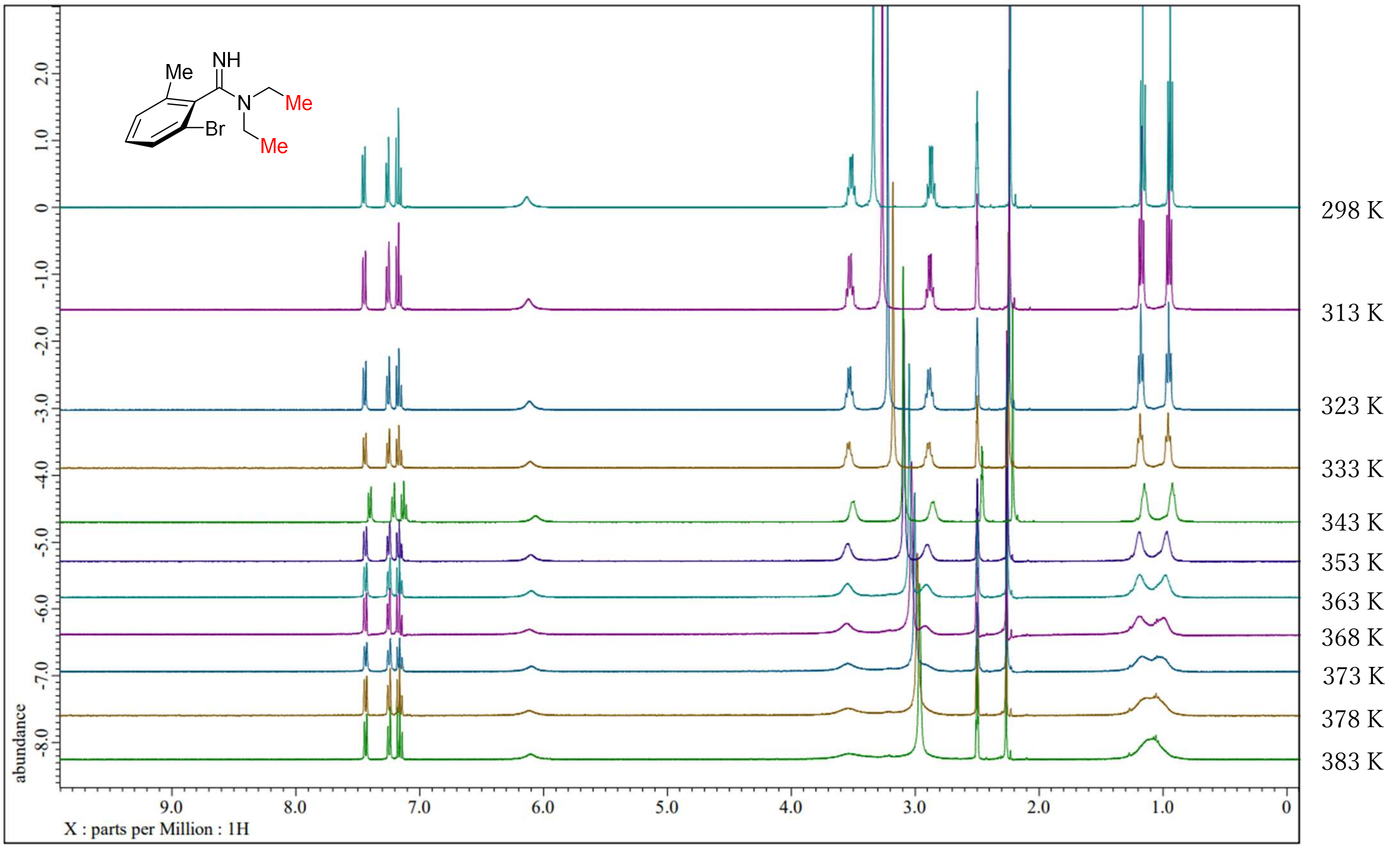


**Figure S3.1**. Overall view of vt-$^1$H NMR spectrum of the neutral form of **1**

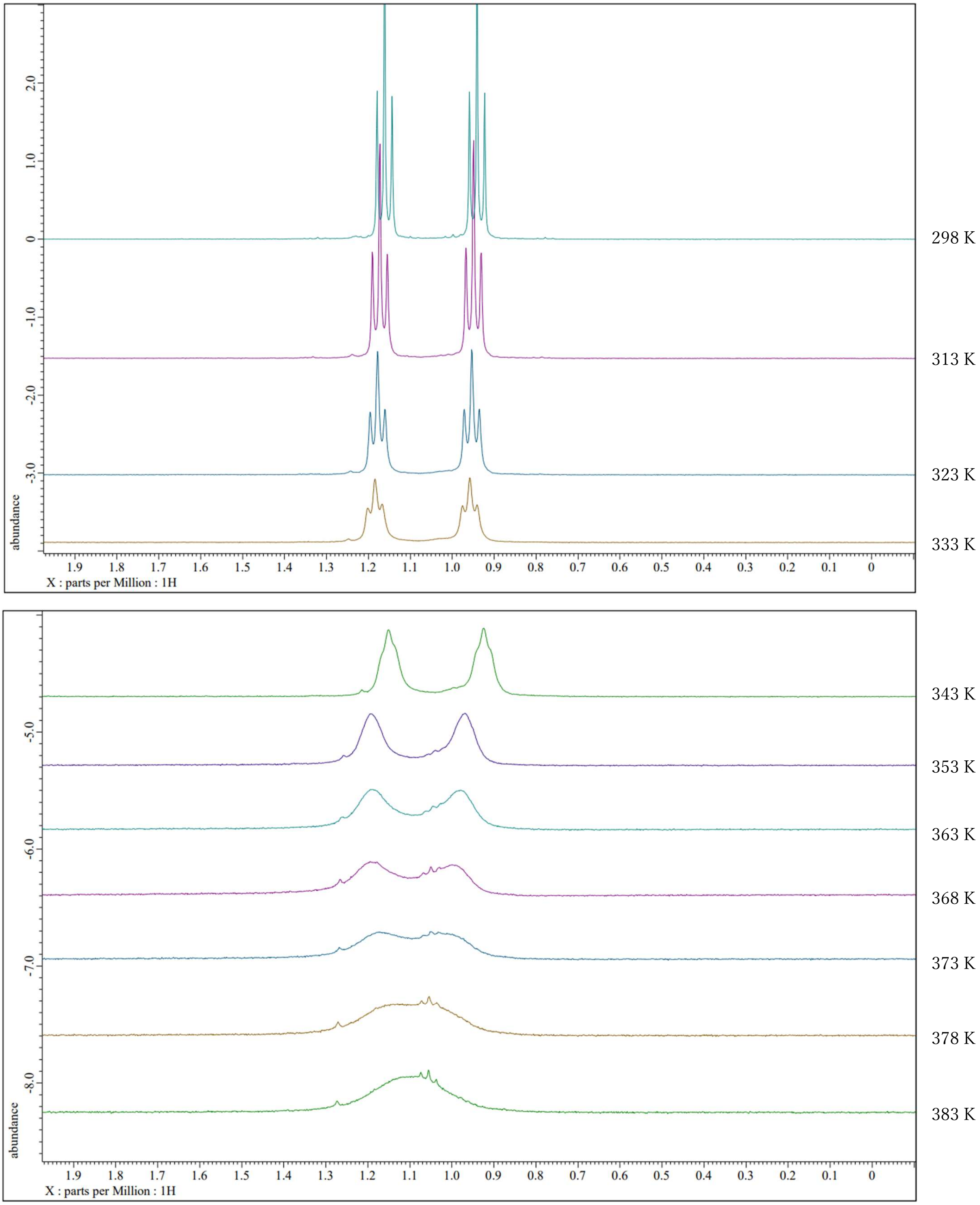


**Figure S3.2**. Enlarged view of vt-$^{1}$H NMR spectrum of neutral form of **1**

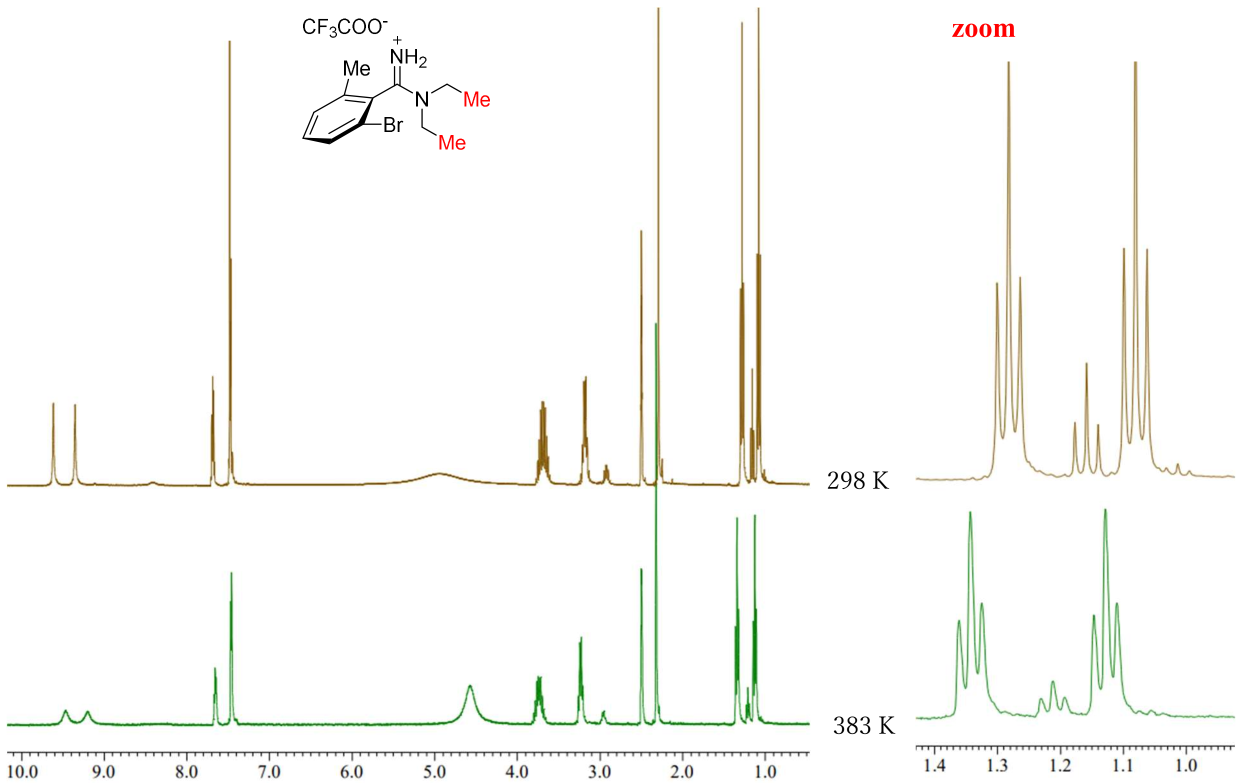


**Figure S3.3**. Overall and enlarged view of vt-$^{1}$H NMR spectrum of trifluoroacetate salt of **1**

## 4. Separation of the *E*/*Z* isomers

A *E*/*Z* mixture of synthesized amidines was purified by RP-HPLC. The purity of the isomers was determined by LC-MS. The Purification by RP-HPLC was performed under the following conditions. Since the eluent contained trifluoroacetic acid, each amidine was observed as its trifluoroacetate salt in the HPLC chart shown in Figure S4.1-4.6.

Conditions for compound **2**
column: COSMOSIL Packed Column 5$C_{18}$-AR-II 20 mmID×250 mm, flow rate: 8.0 mL/min, eluent: A; 0.1% trifluoro acetic acid in $H_2O$, B; MeCN, 28% B in A isocratic elusion, detection: UV (254 nm).

Conditions for compound **3**
column: COSMOSIL Packed Column 5$C_{18}$-AR-II 20 mmID×250 mm, flow rate: 8.0 mL/min, eluent: A; 0.1% trifluoro acetic acid in $H_2O$, B; MeCN, 32% B in A isocratic elusion, detection: UV (254 nm).

Conditions for compound **4**
column: COSMOSIL Packed Column 5$C_{18}$-AR-II 20 mmID×250 mm, flow rate: 8.0 mL/min, eluent: A; 0.1% trifluoro acetic acid in $H_2O$, B; MeCN, 28% B in A isocratic elusion, detection: UV (254 nm).

Conditions for compound **5**
column: COSMOSIL Packed Column 5$C_{18}$-AR-II 20 mmID×250 mm, flow rate: 8.0 mL/min, eluent: A; 0.1% trifluoro acetic acid in $H_2O$, B; MeCN, 28% B in A isocratic elusion, detection: UV (254 nm).

Conditions for compound **6**
column: COSMOSIL Packed Column 5$C_{18}$-AR-II 20 mmID×250 mm, flow rate: 8.0 mL/min, eluent: A; 0.1% trifluoro acetic acid in $H_2O$, B; MeCN, 28% B in A isocratic elusion, detection: UV (254 nm).

Conditions for compound **7**
column: COSMOSIL Packed Column 5$C_{18}$-AR-II 20 mmID×250 mm, flow rate: 8.0 mL/min, eluent: A; 0.1% trifluoro acetic acid in $H_2O$, B; MeCN, 28% B in A isocratic elusion, detection: UV (254 nm).

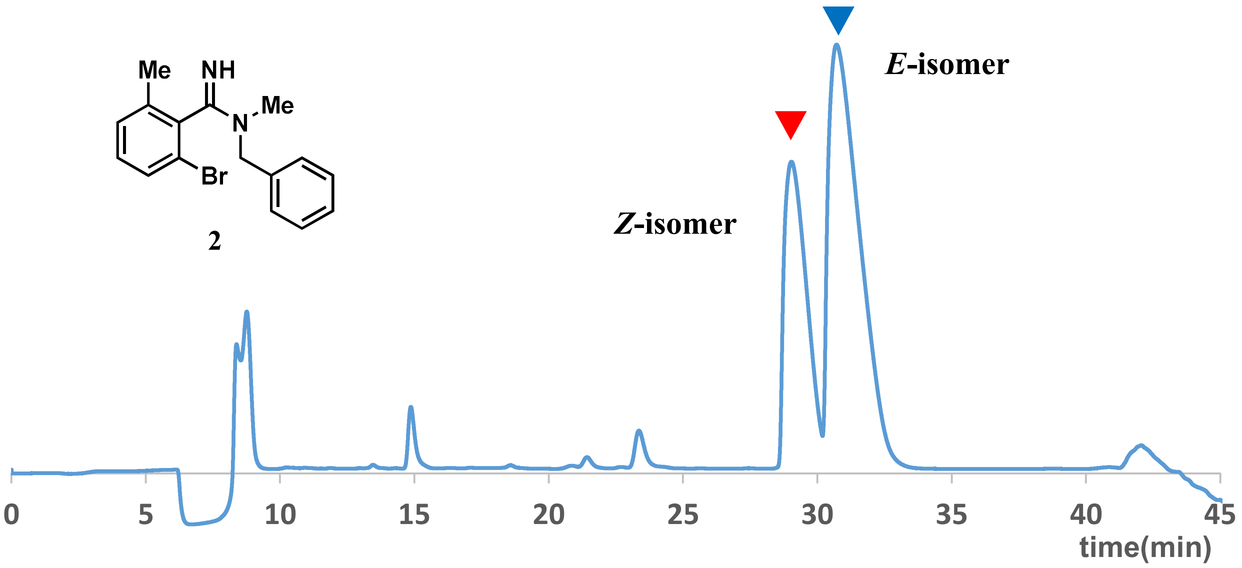


**Figure S4.1**. HPLC chart of *E*- and *Z*- isomer of amidine **2**

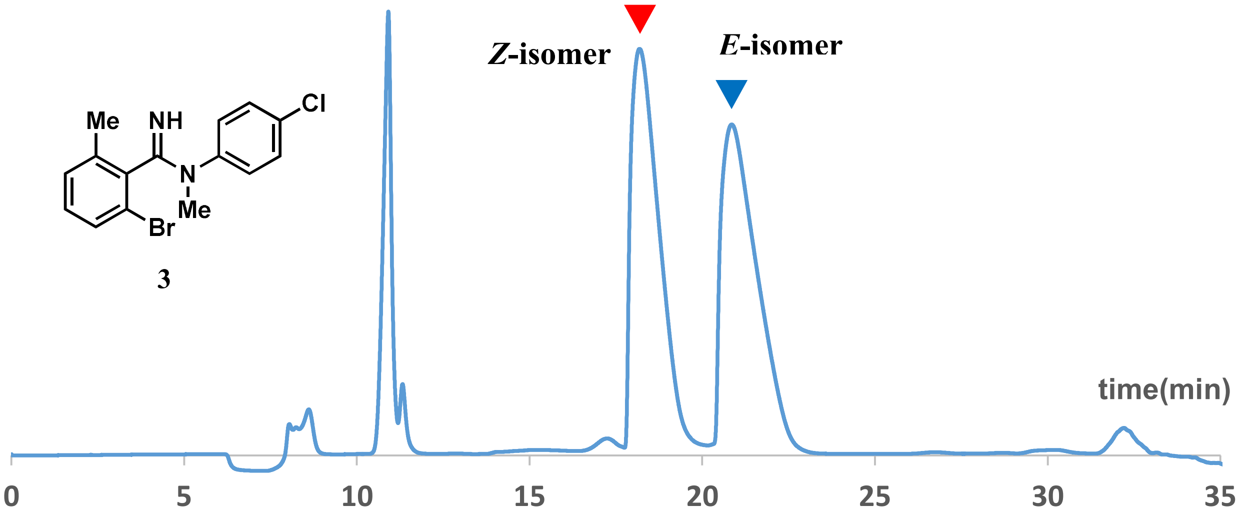


**Figure S4.2**. HPLC chart of *E*- and *Z*- isomer of amidine **3**

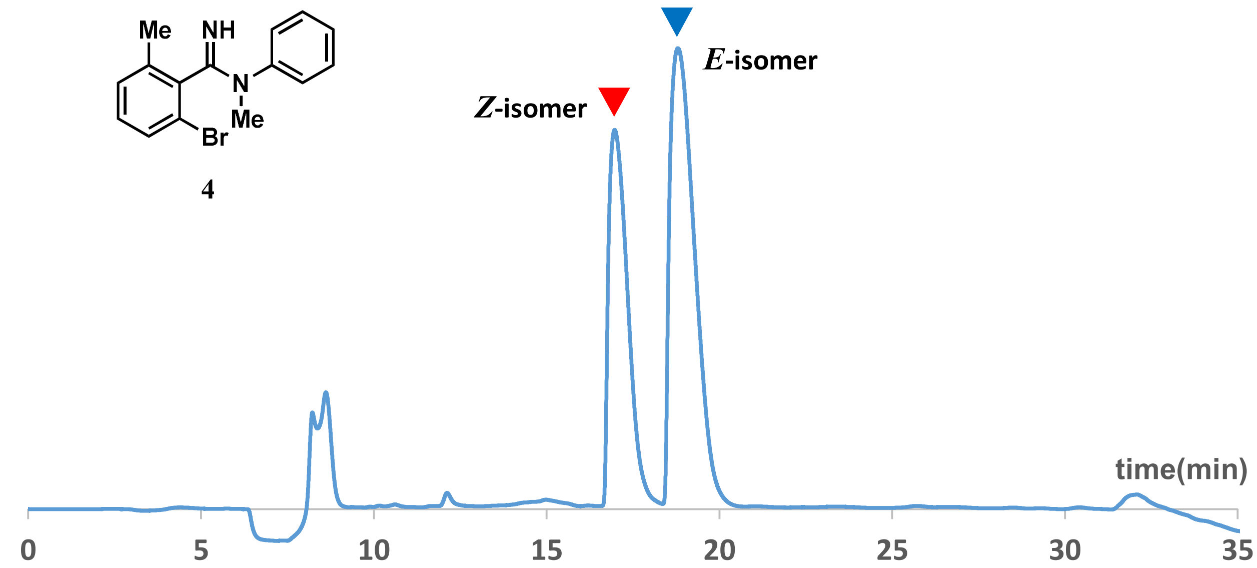


**Figure S4.3**. HPLC chart of *E*- and *Z*- isomer of amidine **4**

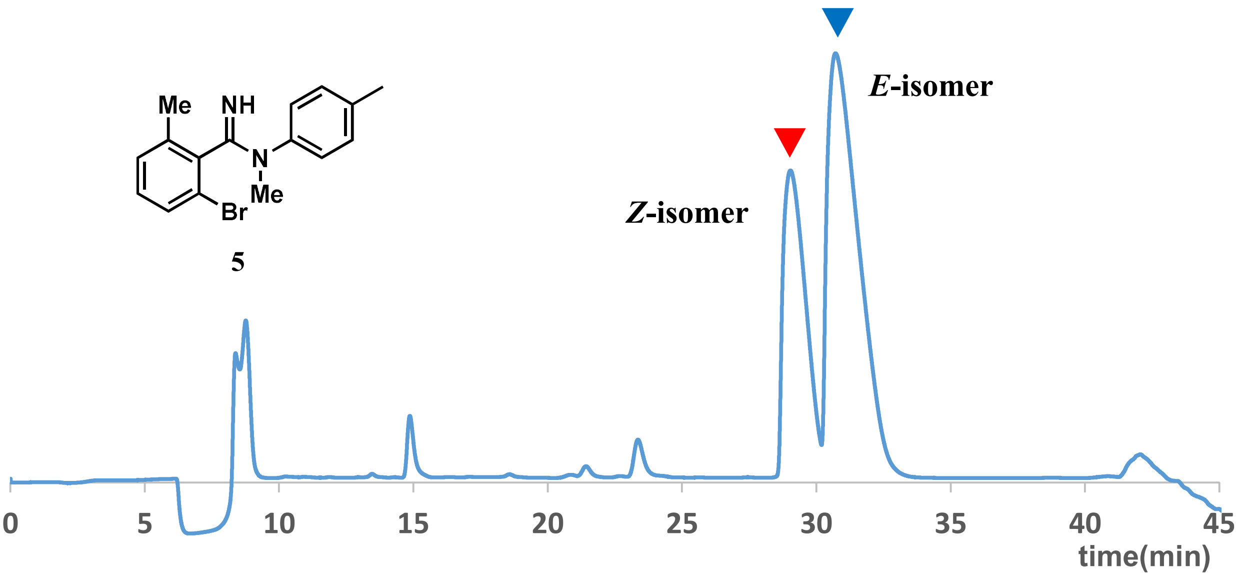


**Figure S4.4**. HPLC chart of *E*- and *Z*- isomer of amidine **5**

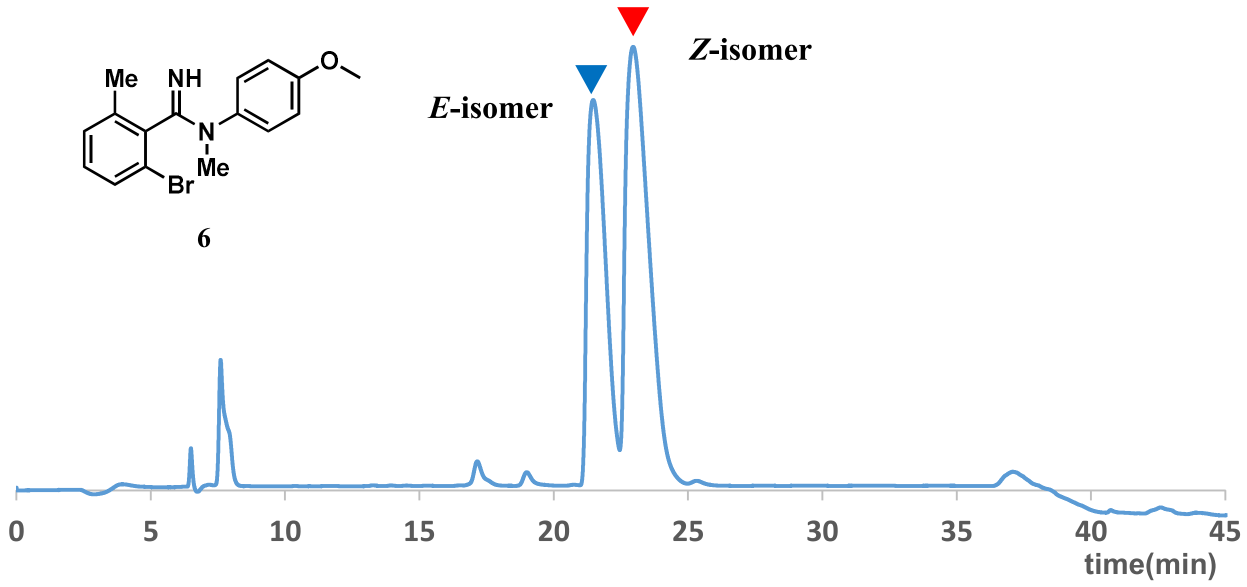


**Figure S4.5**. HPLC chart of *E*- and *Z*- isomer of amidine **6**

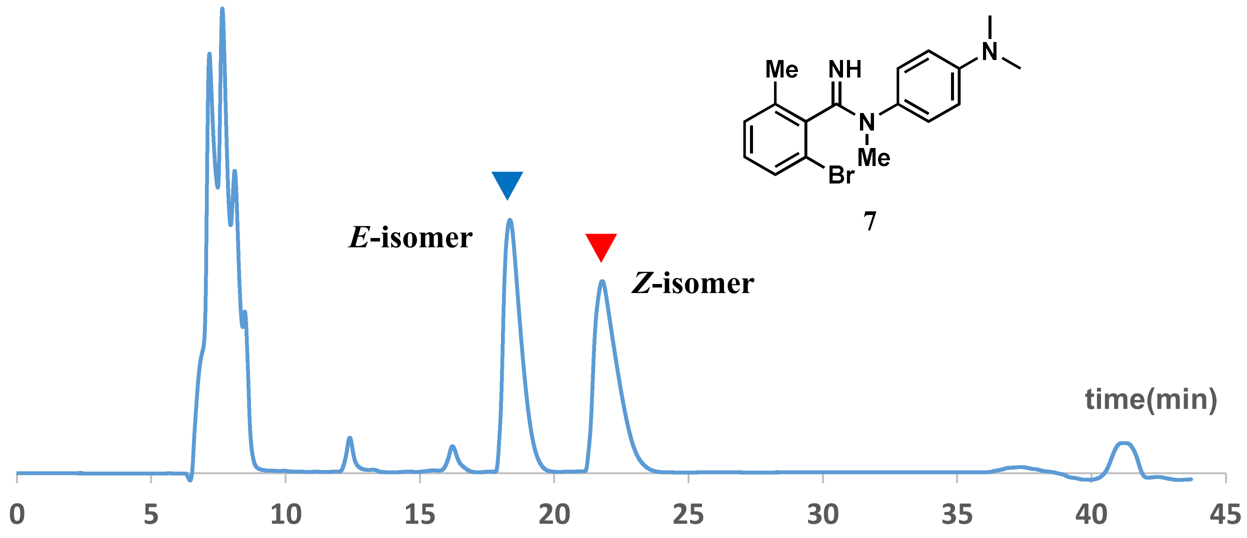


**Figure S4.6**. HPLC chart of *E*- and *Z*- isomer of amidine **7**

## 5. Monitoring of the *E*/*Z*-isomerization

Procedure A (for compound **2**)

After the separation of the *E*/*Z* isomers, a solution of the *Z* isomer was concentrated *in vacuo* and diluted with a buffer, and the resulting solution was maintained at 20 °C. The progress of the isomerization was monitored by LC-MS analysis.

Procedure B (for compounds **3-7**)

After the separation of the *E*/*Z* isomers, a solution of the *Z* isomer was diluted with a buffer and MeCN (final ratio: buffer/MeCN = 95/5), and the resulting solution was maintained at 30 °C. The progress of the isomerization was monitored by LC-MS analysis.

The ratio of the absorbance coefficients at 254 nm for each geometric isomer was determined by comparing the integral ratio of $^1$H NMR spectra and the area ratio of UV chromatogram of the LC-MS analysis (detailed conditions were described below). The ratio of the absorbance coefficients was used to calculate the molar ratios at each time point from the UV chromatogram of the LC-MS analysis. The ratio of each isomer was plotted as a function of time, and curve-fitting analysis of the experimental results using differential equations describing the isomerization (eq. 2, 3), to calculate kinetic constant *k* (Figure S5.1-5.23).

$$\frac{d[E]}{dt} = k_{Z \to E}[Z] - k_{E \to Z}[E] \quad \text{(eq. 2)}$$

$$\frac{d([E]+[Z])}{dt} = 0 \quad \text{(eq. 3)}$$

LC-MS conditions:

For compound **2**
column: J'sphere ODS-M80, 150 × 4.6 mmI.D., S-4 μm, 8 nm, flow rate: 0.4 mL/min, eluent: A; 0.1% aqueous formic acid in $H_2O$, B; MeCN, 25% B in A isocratic elusion, detection: UV (254 nm)

For compound **3**
column: J'sphere ODS-M80, 150 × 4.6 mmI.D., S-4 μm, 8 nm, flow rate: 0.4 mL/min, eluent: A; 0.1% aqueous formic acid in $H_2O$, B; MeCN, 28% B in A isocratic elusion, detection: UV (254 nm)

For compound **4**
column: J'sphere ODS-M80, 150 × 4.6 mmI.D., S-4 μm, 8 nm, flow rate: 0.4 mL/min, eluent: A; 0.1% aqueous formic acid in $H_2O$, B; MeCN, 24% B in A isocratic elusion, detection: UV (254 nm)

For compound **5**
column: J'sphere ODS-M80, 150 × 4.6 mmI.D., S-4 μm, 8 nm, flow rate: 0.4 mL/min, eluent: A; 0.1% aqueous formic acid in $H_2O$, B; MeCN, 24% B in A isocratic elusion, detection: UV (254 nm)

For compound **6**
column: J'sphere ODS-M80, 150 × 4.6 mmI.D., S-4 μm, 8 nm, flow rate: 0.4 mL/min, eluent: A; 0.1% aqueous formic acid in $H_2O$, B; MeCN, 24% B in A isocratic elusion, detection: UV (254 nm)

For compound **7**
column: J'sphere ODS-M80, 150 × 4.6 mmI.D., S-4 μm, 8 nm, flow rate: 0.4 mL/min, eluent: A; 0.1% aqueous formic acid in $H_2O$, B; MeCN, 28% B in A isocratic elusion, detection: UV (254 nm)

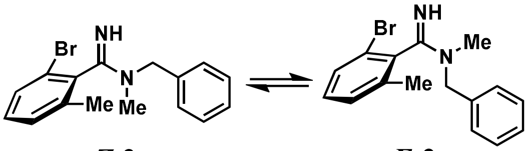


***Z*-2** ***E*-2**

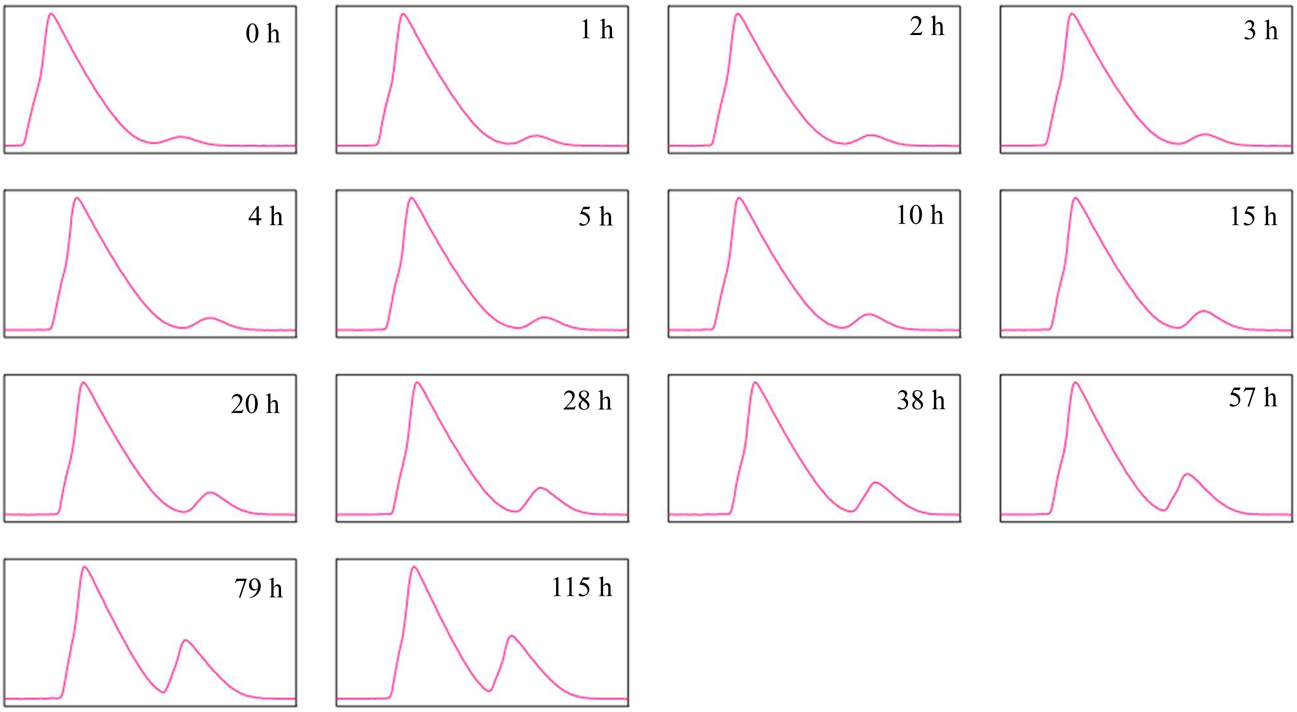


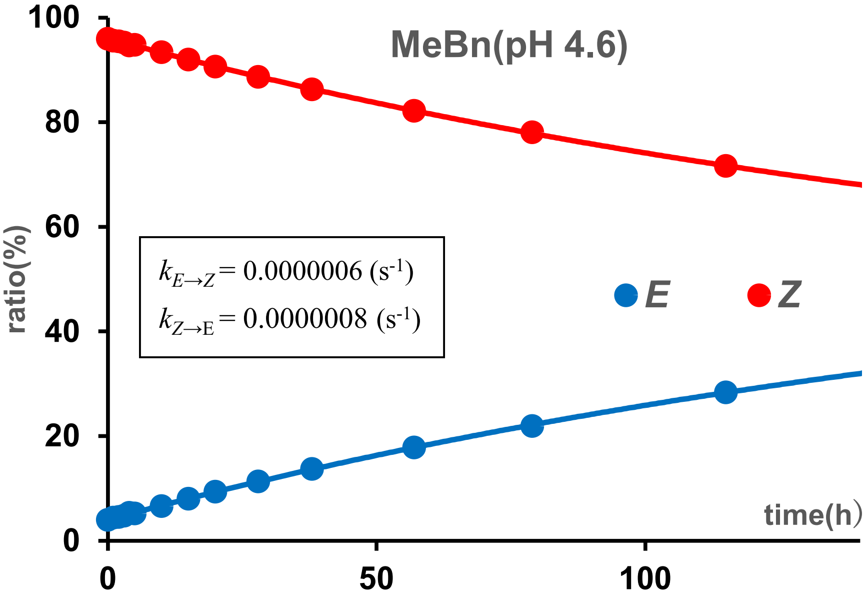


**ratio**

| time (h) | *E* | *Z* |
|---|---|---|
| 0 | 4.03 | 95.97 |
| 1 | 4.42 | 95.58 |
| 2 | 4.54 | 95.46 |
| 3 | 4.77 | 95.23 |
| 4 | 5.33 | 94.67 |
| 5 | 5.24 | 94.76 |
| 10 | 6.64 | 93.36 |
| 15 | 8.04 | 91.96 |
| 20 | 9.39 | 90.61 |
| 28 | 11.37 | 88.63 |
| 38 | 13.72 | 86.28 |
| 57 | 17.86 | 82.14 |
| 79 | 21.96 | 78.04 |
| 115 | 28.39 | 71.61 |

**Conditions: 20 °C in 100 mM acetate buffer**

**Figure S5.1**. Isomerization of compound **2** at pH 4.6

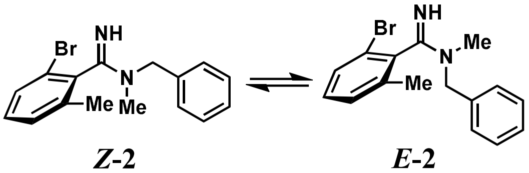


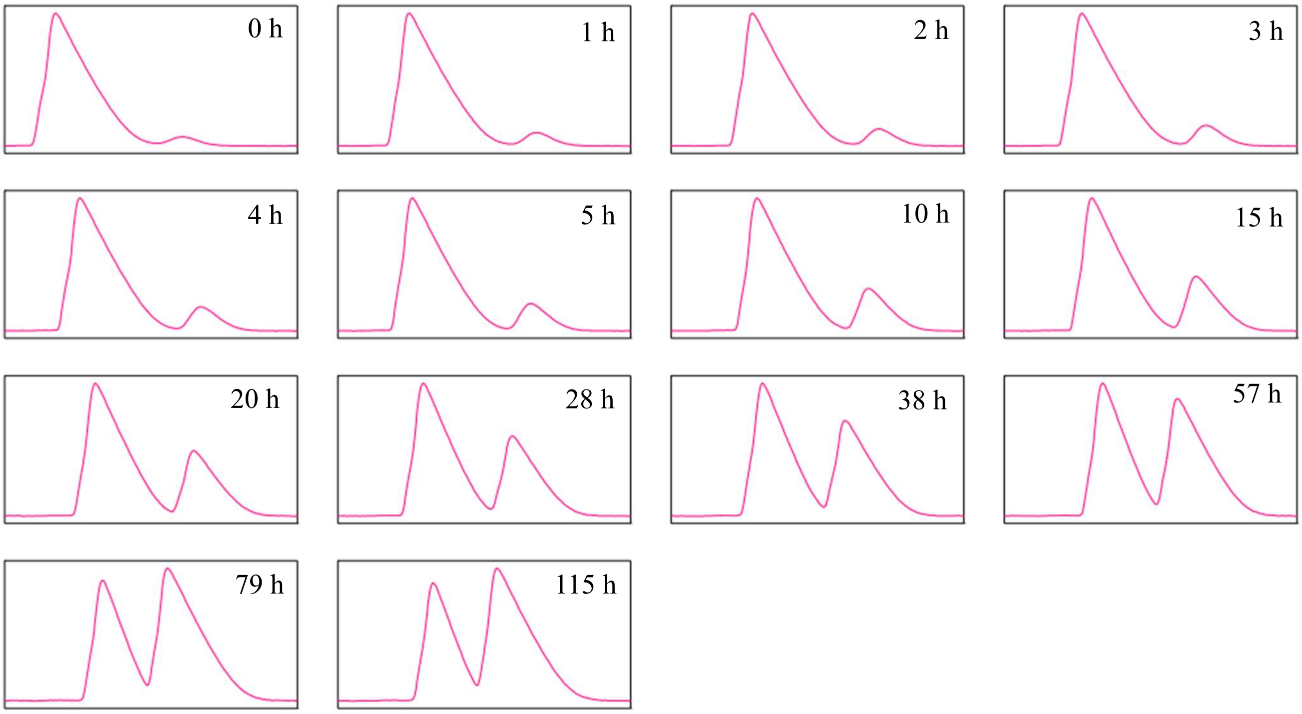


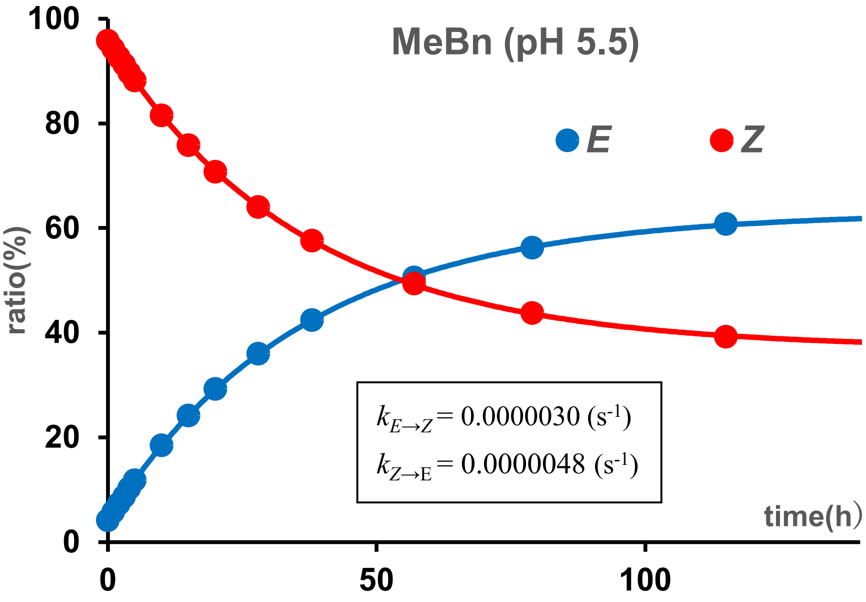


**ratio**

| time (h) | *E* | *Z* |
|---|---|---|
| 0 | 4.18 | 95.82 |
| 1 | 5.74 | 94.26 |
| 2 | 7.32 | 92.68 |
| 3 | 8.71 | 91.29 |
| 4 | 10.29 | 89.71 |
| 5 | 11.81 | 88.19 |
| 10 | 18.48 | 81.52 |
| 15 | 24.16 | 75.84 |
| 20 | 29.25 | 70.75 |
| 28 | 35.98 | 64.02 |
| 38 | 42.42 | 57.58 |
| 57 | 50.57 | 49.43 |
| 79 | 56.23 | 43.77 |
| 115 | 60.78 | 39.22 |

**Conditions: 20 °C in 100 mM acetate buffer**

**Figure S5.2**. Isomerization of compound **2** at pH 5.5

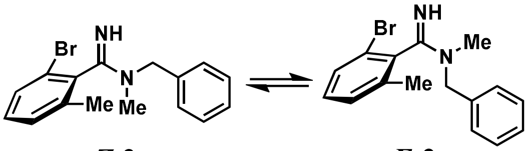


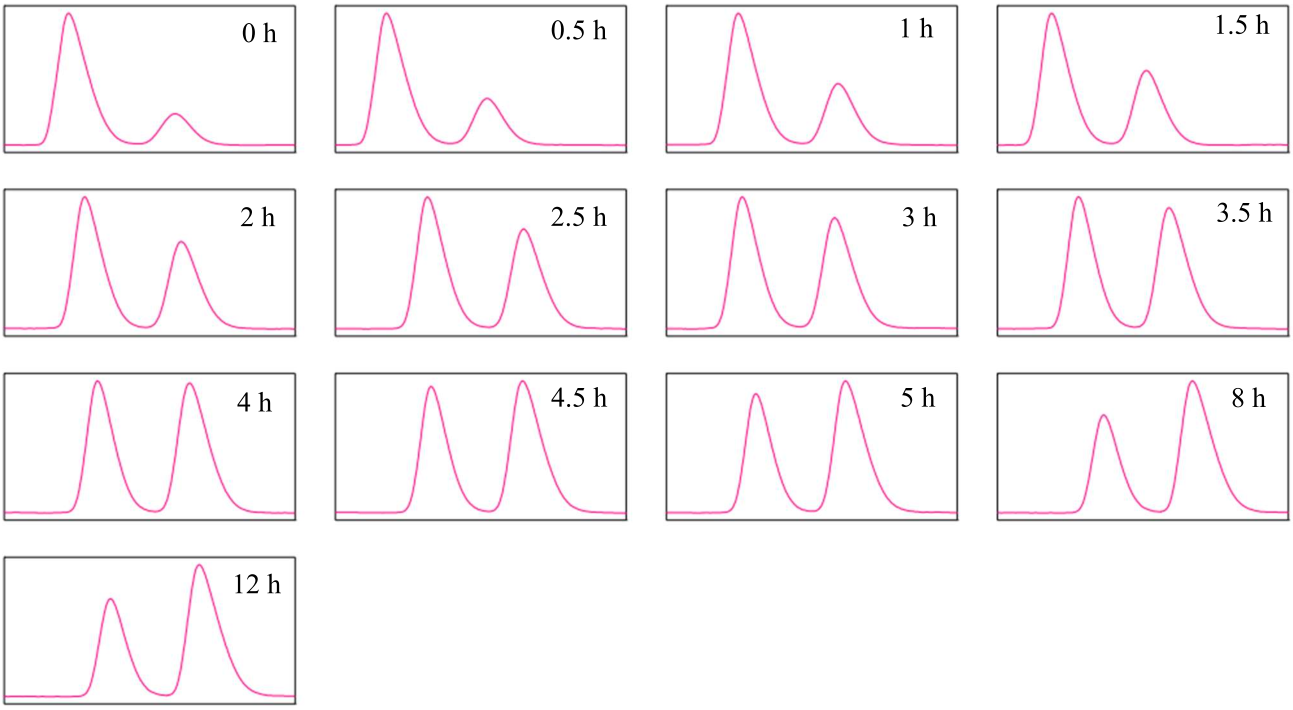


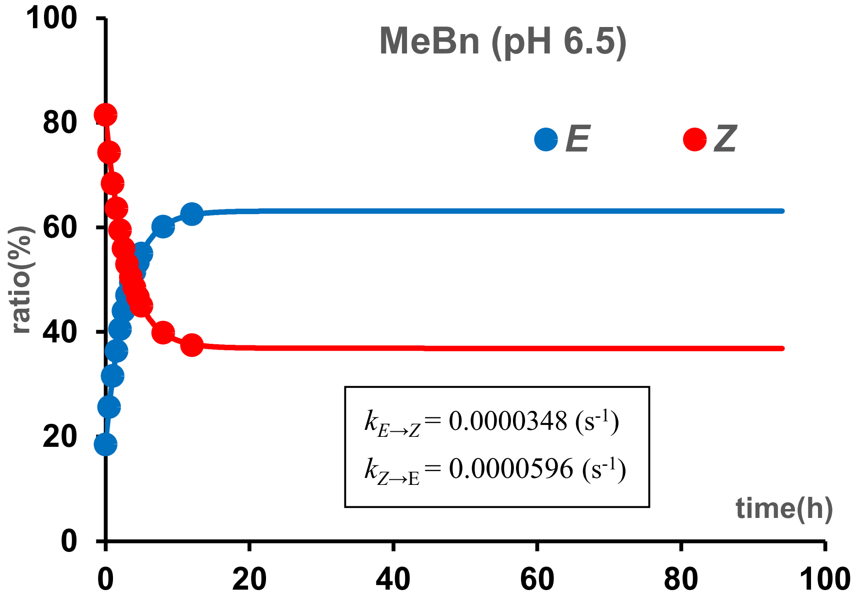


**ratio**

| time (h) | *E* | *Z* |
|---|---|---|
| 0 | 18.51 | 81.49 |
| 0.5 | 25.68 | 74.32 |
| 1 | 31.61 | 68.39 |
| 1.5 | 36.36 | 63.64 |
| 2 | 40.54 | 59.46 |
| 2.5 | 44.01 | 55.99 |
| 3 | 47.03 | 52.97 |
| 3.5 | 49.50 | 50.50 |
| 4 | 51.48 | 48.52 |
| 4.5 | 53.31 | 46.69 |
| 5 | 54.96 | 45.04 |
| 8 | 60.13 | 39.87 |
| 12 | 62.50 | 37.50 |

**Conditions: 20 °C in 100 mM phosphate buffer**

**Figure S5.3**. Isomerization of compound **2** at pH 6.5

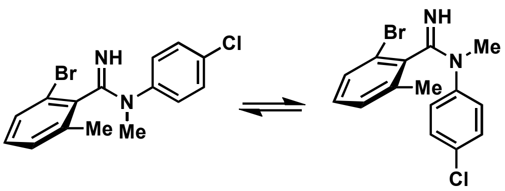


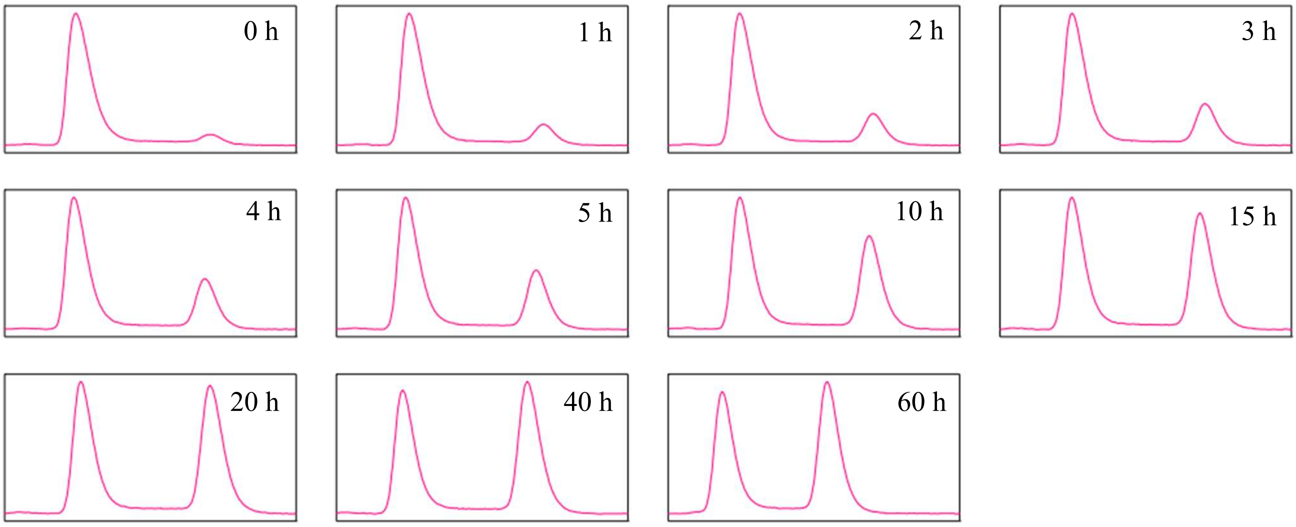


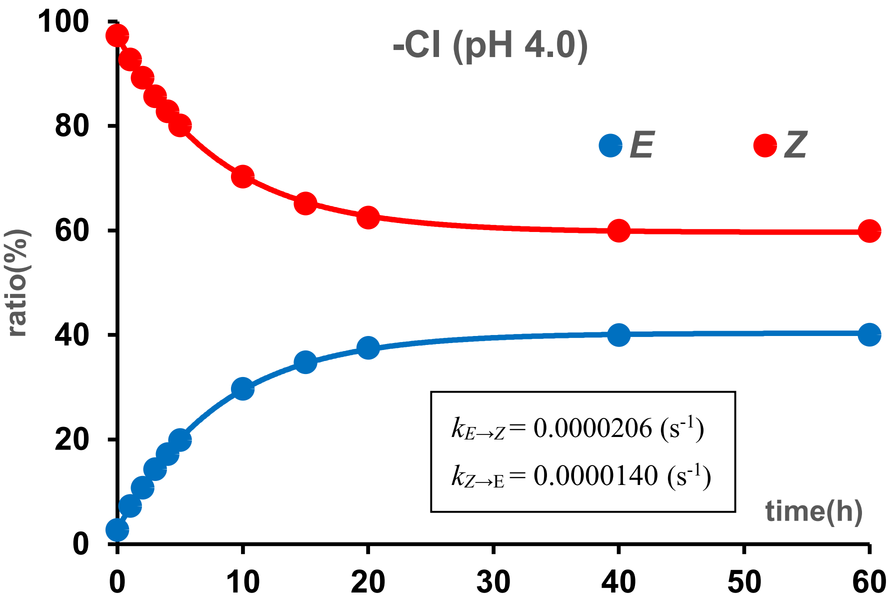


**ratio**

| time (h) | *E* | *Z* |
|---|---|---|
| 0 | 2.69 | 97.31 |
| 1 | 7.32 | 92.68 |
| 2 | 10.79 | 89.21 |
| 3 | 14.35 | 85.65 |
| 4 | 17.24 | 82.76 |
| 5 | 19.92 | 80.08 |
| 10 | 29.69 | 70.31 |
| 15 | 34.83 | 65.17 |
| 20 | 37.53 | 62.47 |
| 40 | 40.02 | 59.98 |
| 60 | 40.08 | 59.92 |

**Conditions: 30 °C in 200 mM acetate buffer/MeCN = 95/5**

**Figure S5.4**. Isomerization of compound **3** at pH 4.0

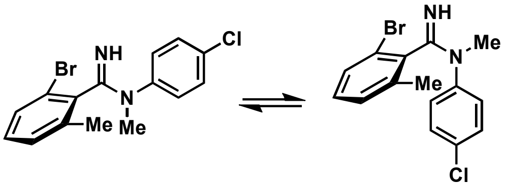


*Z*-3 *E*-3

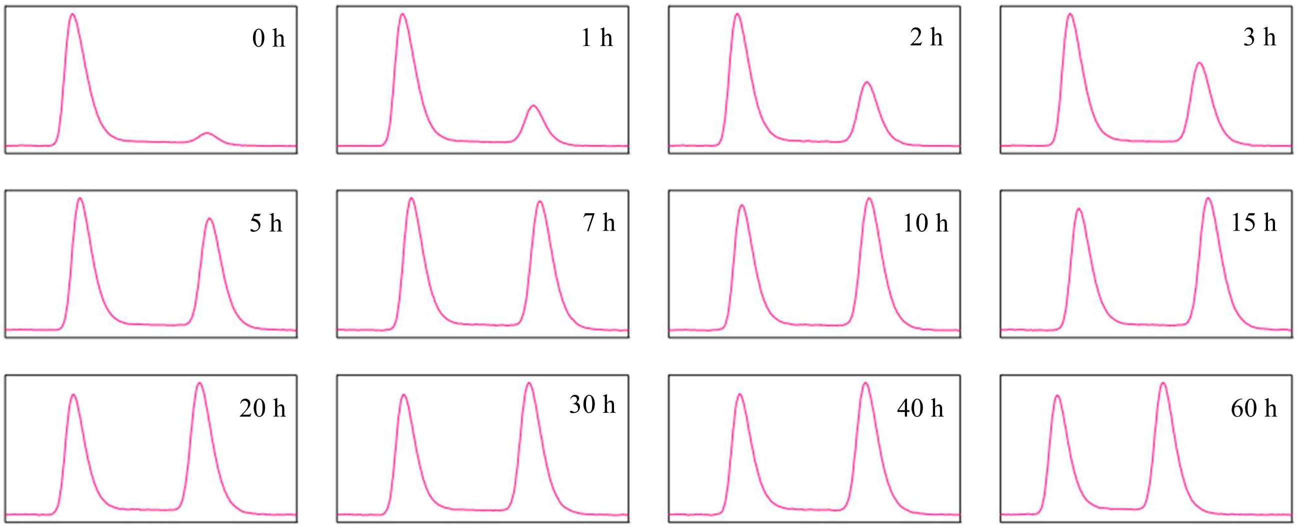


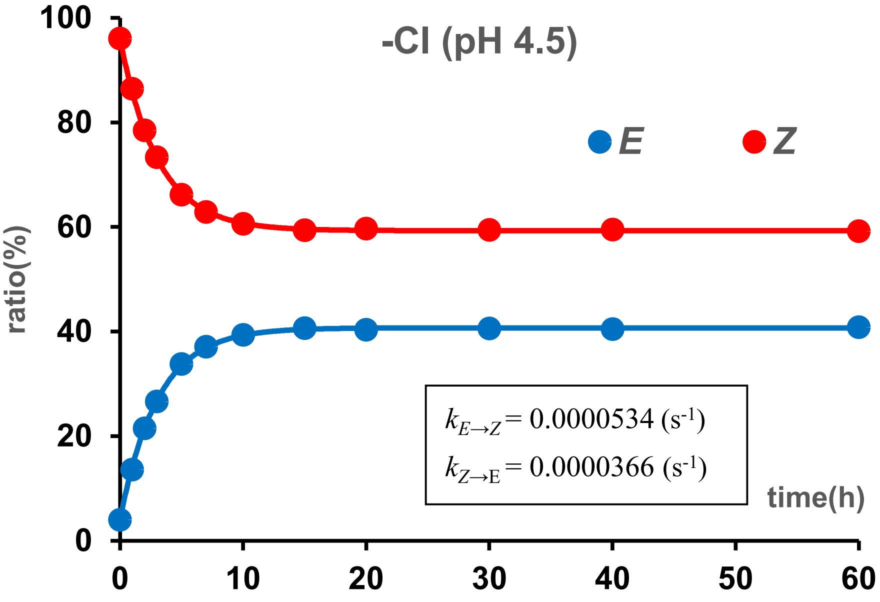


**ratio**

| time (h) | *E* | *Z* |
|---|---|---|
| 0 | 3.96 | 96.04 |
| 1 | 13.57 | 86.43 |
| 2 | 21.52 | 78.48 |
| 3 | 26.64 | 73.36 |
| 5 | 33.80 | 66.20 |
| 7 | 37.11 | 62.89 |
| 10 | 39.37 | 60.63 |
| 15 | 40.64 | 59.36 |
| 20 | 40.32 | 59.68 |
| 30 | 40.57 | 59.43 |
| 40 | 40.50 | 59.50 |
| 60 | 40.82 | 59.18 |

**Conditions: 30 °C in 200 mM acetate buffer/MeCN = 95/5**

**Figure S5.5**. Isomerization of compound **3** at pH 4.5

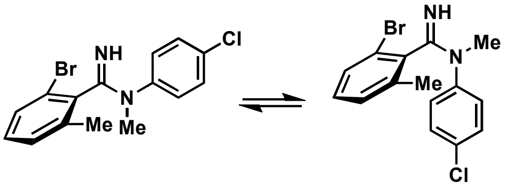


*Z*-3 *E*-3

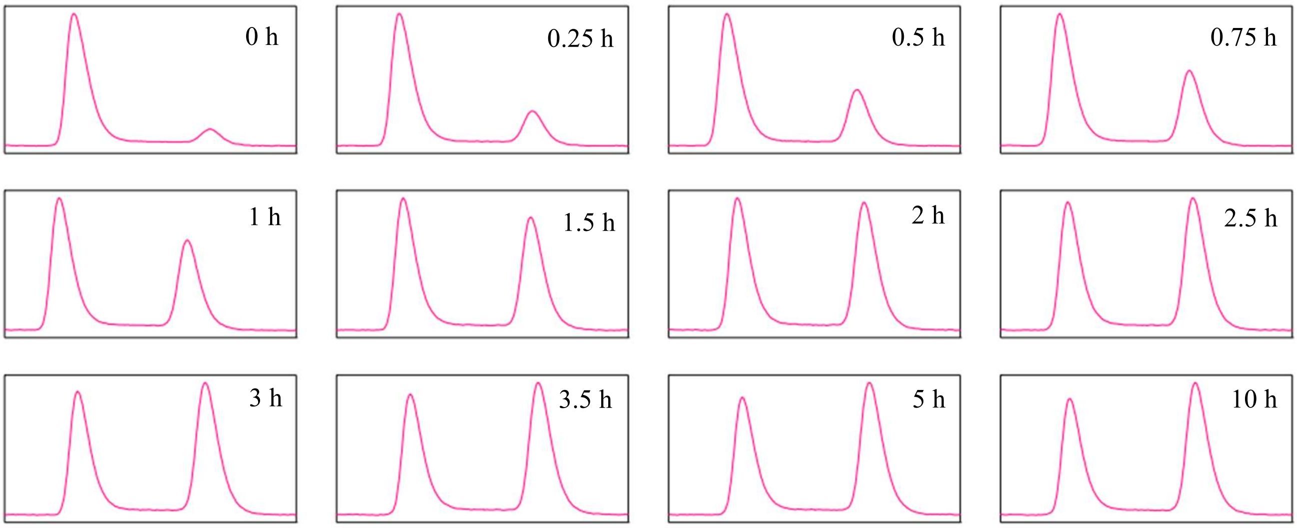


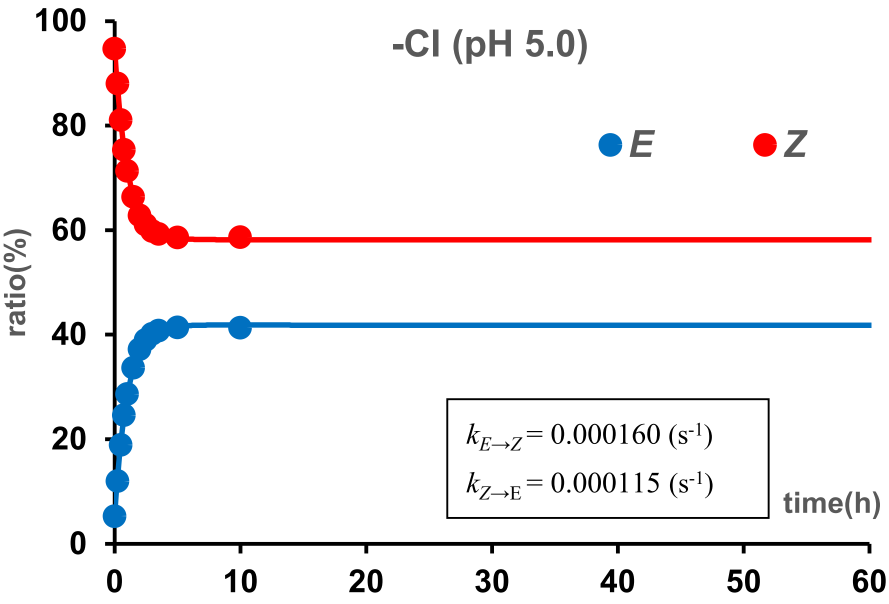


**ratio**

| time (h) | *E* | *Z* |
|---|---|---|
| 0 | 5.29 | 94.71 |
| 0.25 | 11.98 | 88.02 |
| 0.5 | 18.94 | 81.06 |
| 0.75 | 24.61 | 75.39 |
| 1 | 28.68 | 71.32 |
| 1.5 | 33.65 | 66.35 |
| 2 | 37.21 | 62.79 |
| 2.5 | 38.98 | 61.02 |
| 3 | 40.16 | 59.84 |
| 3.5 | 40.76 | 59.24 |
| 5 | 41.41 | 58.59 |
| 10 | 41.32 | 58.68 |

**Conditions: 30 °C in 200 mM acetate buffer/MeCN = 95/5**

**Figure S5.6**. Isomerization of compound **3** at pH 5.0

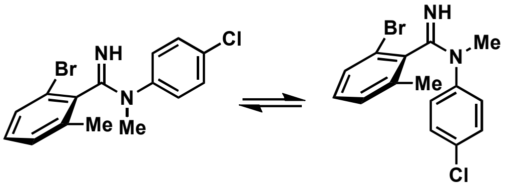


***Z*-3** ⇌ ***E*-3**

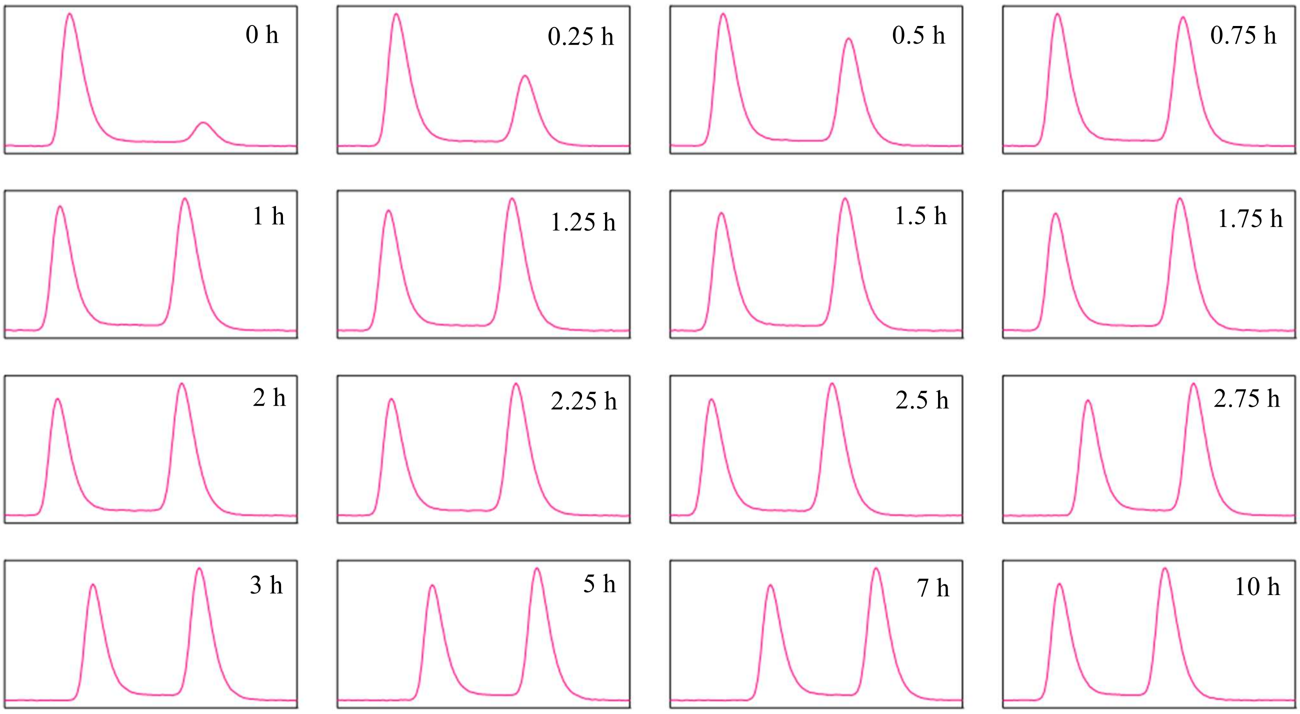


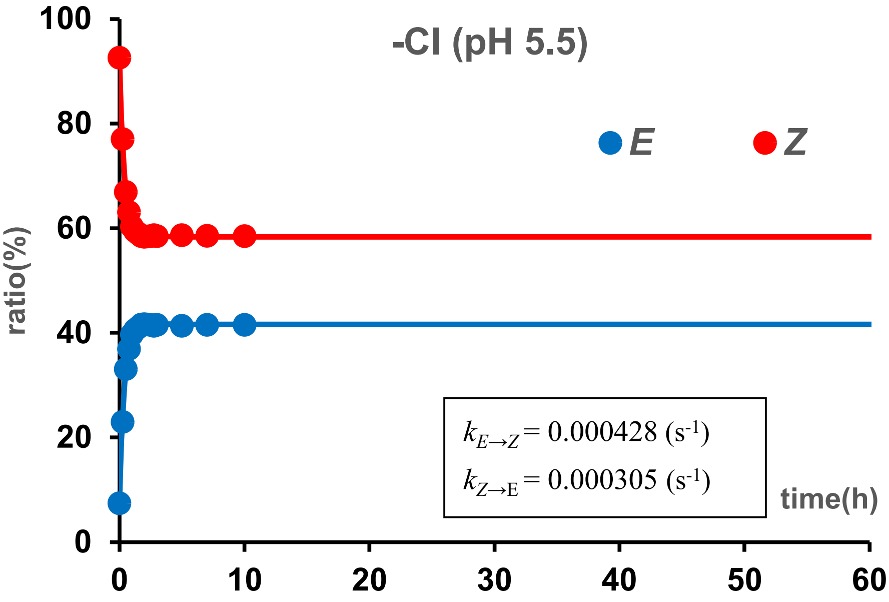


**ratio**

| time (h) | *E* | *Z* |
|---|---|---|
| 0 | 7.40 | 92.60 |
| 0.25 | 22.96 | 77.04 |
| 0.5 | 33.07 | 66.93 |
| 0.75 | 36.93 | 63.07 |
| 1 | 39.71 | 60.29 |
| 1.25 | 40.52 | 59.48 |
| 1.5 | 41.03 | 58.97 |
| 1.75 | 41.55 | 58.45 |
| 2 | 41.62 | 58.38 |
| 2.25 | 41.61 | 58.39 |
| 2.5 | 41.53 | 58.47 |
| 2.75 | 41.31 | 58.69 |
| 3 | 41.55 | 58.45 |
| 5 | 41.36 | 58.64 |
| 7 | 41.49 | 58.51 |
| 10 | 41.52 | 58.48 |

**Conditions: 30 °C in 200 mM acetate buffer/MeCN = 95/5**

**Figure S5.7**. Isomerization of compound **3** at pH 5.5

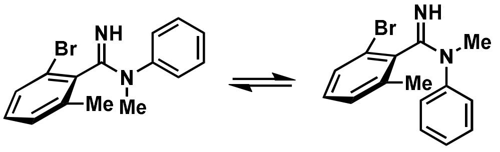


***Z*-4** ⇌ ***E*-4**

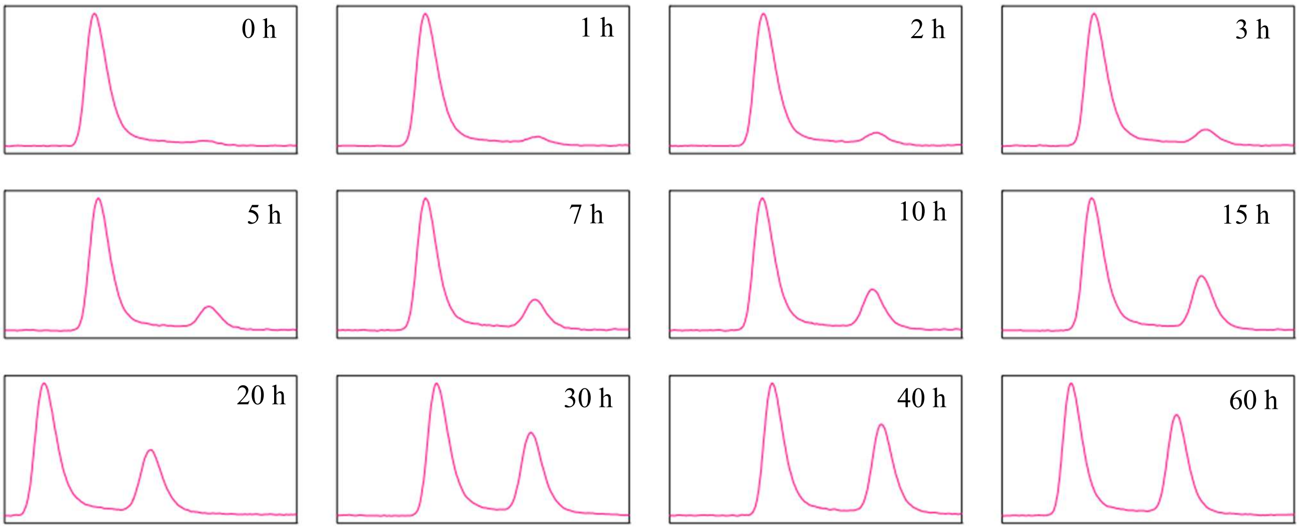


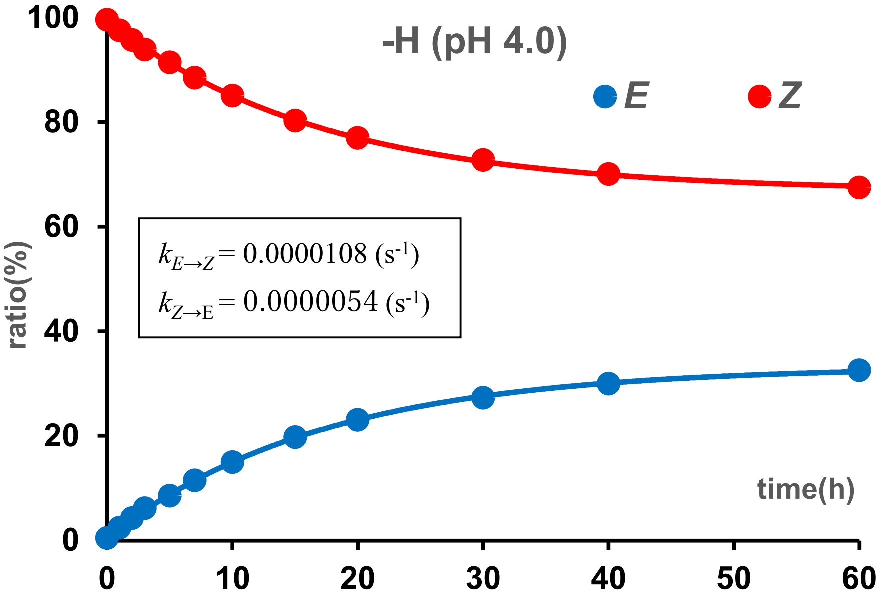


**ratio**

| time (h) | *E* | *Z* |
|---|---|---|
| 0 | 1.09 | 98.91 |
| 1 | 2.44 | 97.56 |
| 2 | 4.29 | 95.71 |
| 3 | 6.14 | 93.86 |
| 5 | 8.56 | 91.44 |
| 7 | 11.48 | 88.52 |
| 10 | 14.96 | 85.04 |
| 15 | 19.72 | 80.28 |
| 20 | 23.04 | 76.96 |
| 30 | 27.26 | 72.74 |
| 40 | 29.95 | 70.05 |
| 60 | 32.51 | 67.49 |

**Conditions: 30 °C in 200 mM acetate buffer/MeCN = 95/5**

**Figure S5.8**. Isomerization of compound **4** at pH 4.0

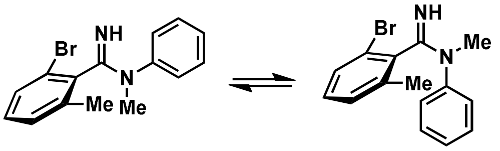


***Z*-4** ⇌ ***E*-4**

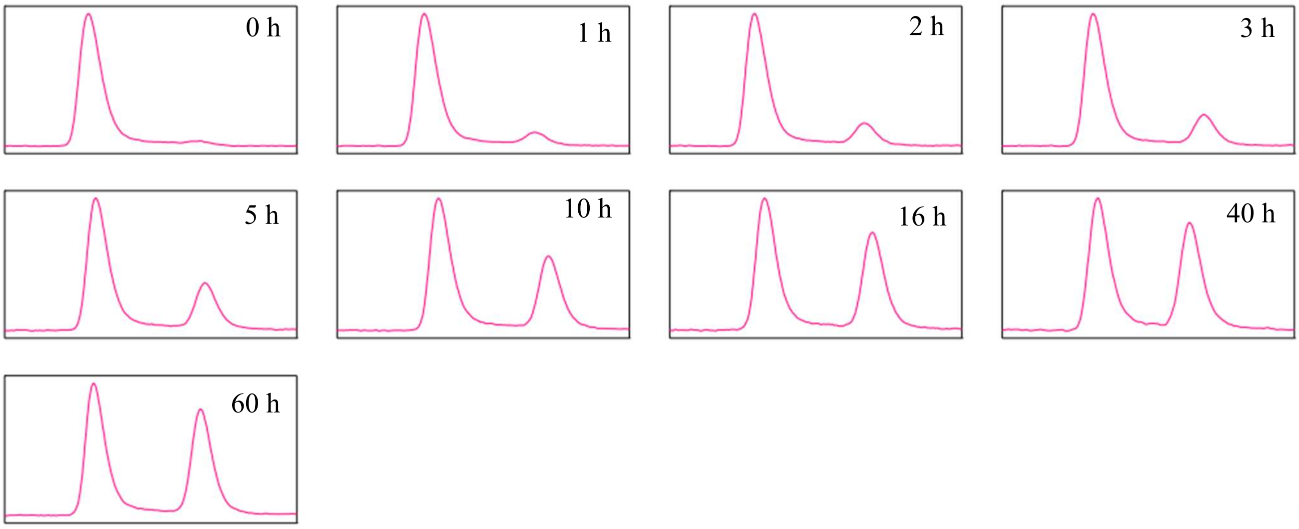


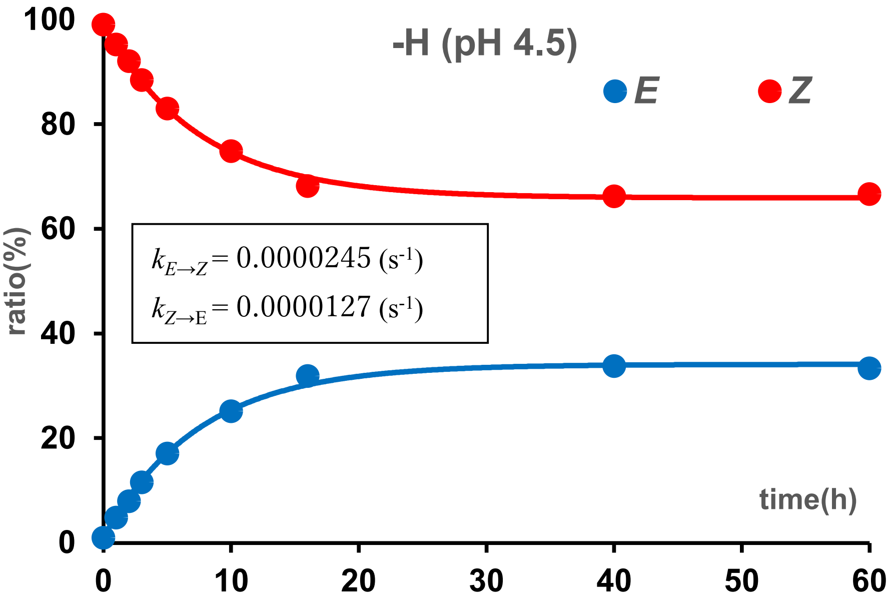


**ratio**

| time (h) | *E* | *Z* |
|---|---|---|
| 0 | 0.99 | 99.01 |
| 1 | 4.79 | 95.21 |
| 2 | 7.93 | 92.07 |
| 3 | 11.56 | 88.44 |
| 5 | 17.03 | 82.97 |
| 10 | 25.17 | 74.83 |
| 16 | 31.86 | 68.14 |
| 40 | 33.79 | 66.21 |
| 60 | 33.34 | 66.66 |

**Conditions: 30 °C in 200 mM acetate buffer/MeCN = 95/5**

**Figure S5.9**. Isomerization of compound **4** at pH 4.5

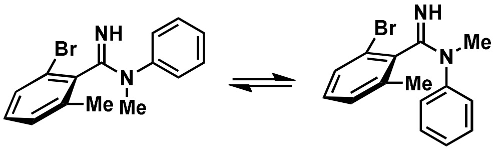


***Z*-4** ***E*-4**

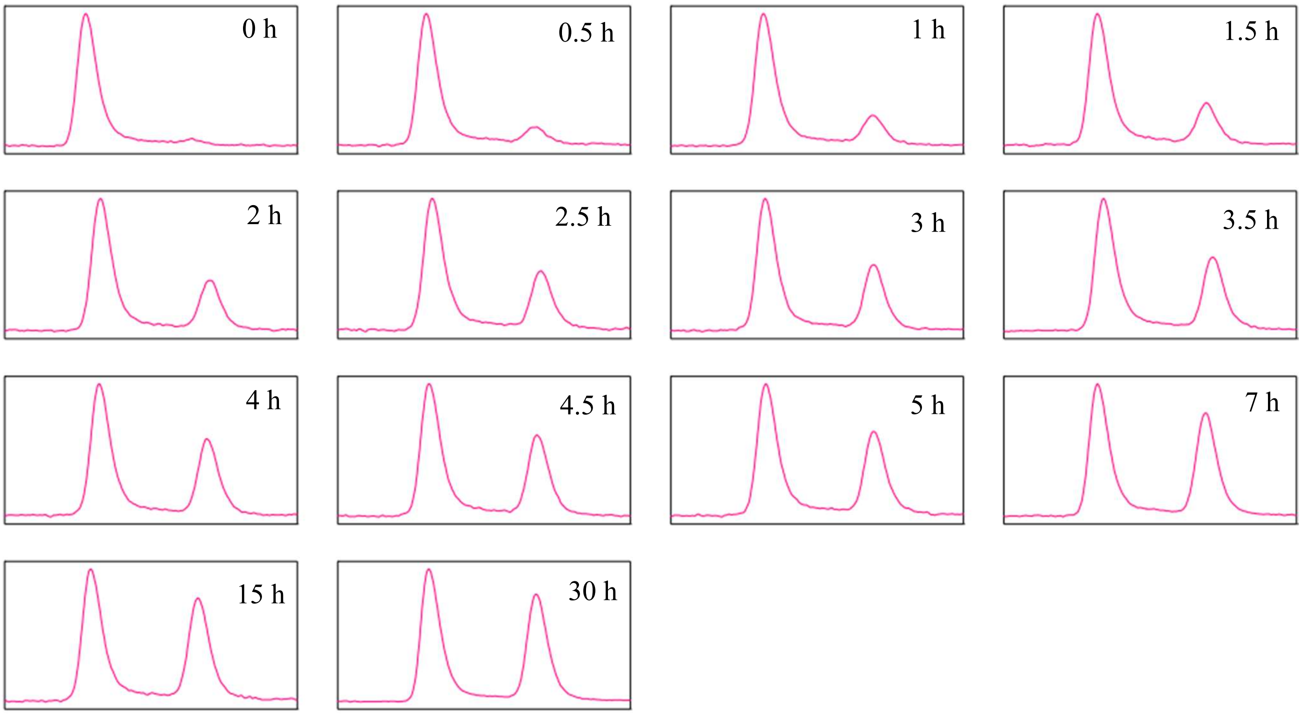


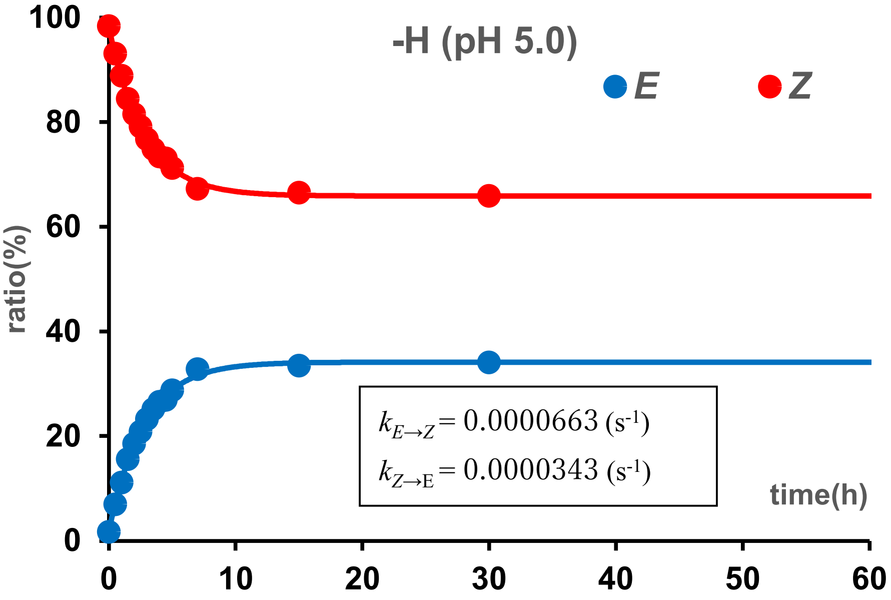


**Conditions: 30 °C in 200 mM acetate buffer/MeCN = 95/5**

**ratio**

| time (h) | *E* | *Z* |
|---|---|---|
| 0 | 1.68 | 98.32 |
| 0.5 | 6.93 | 93.07 |
| 1 | 11.14 | 88.86 |
| 1.5 | 15.56 | 84.44 |
| 2 | 18.51 | 81.49 |
| 2.5 | 20.85 | 79.15 |
| 3 | 23.27 | 76.73 |
| 3.5 | 25.15 | 74.85 |
| 4 | 26.54 | 73.46 |
| 4.5 | 26.92 | 73.08 |
| 5 | 28.75 | 71.25 |
| 7 | 32.77 | 67.23 |
| 15 | 33.49 | 66.51 |
| 30 | 34.08 | 65.92 |

**Figure S5.10**. Isomerization of compound **4** at pH 5.0

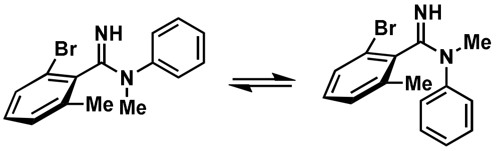


***Z*-4** ***E*-4**

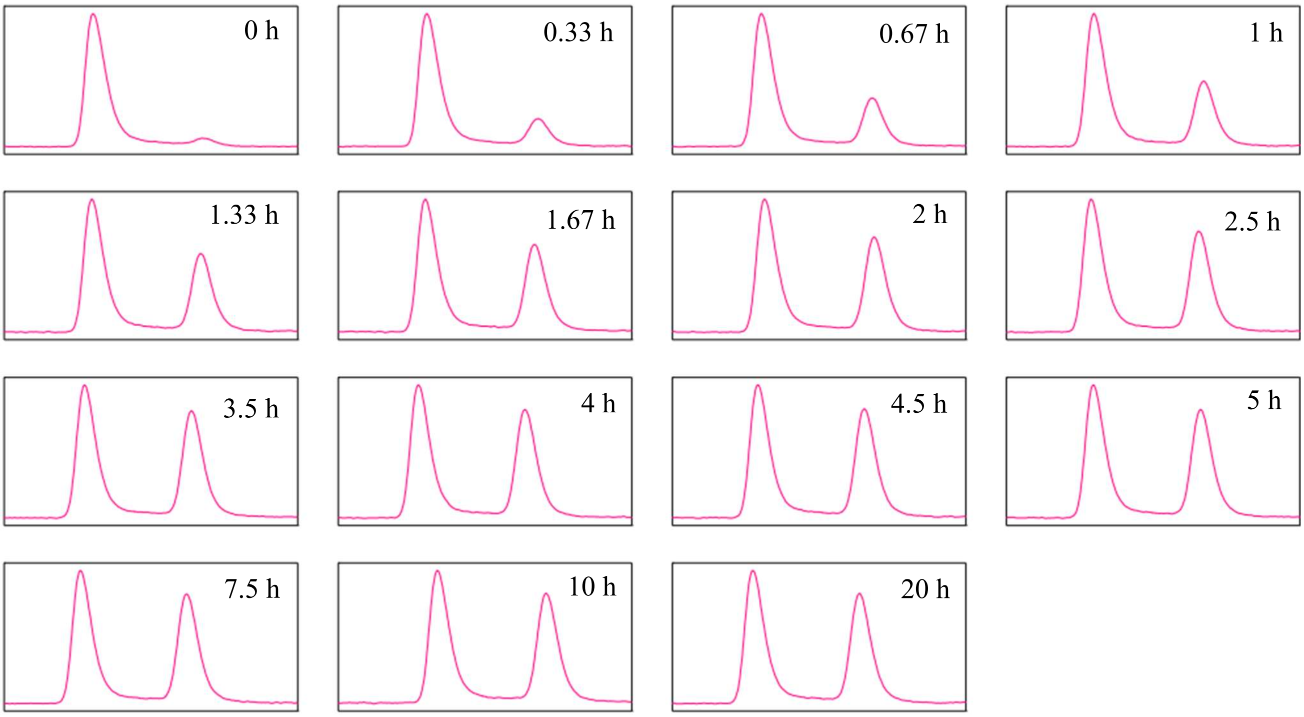


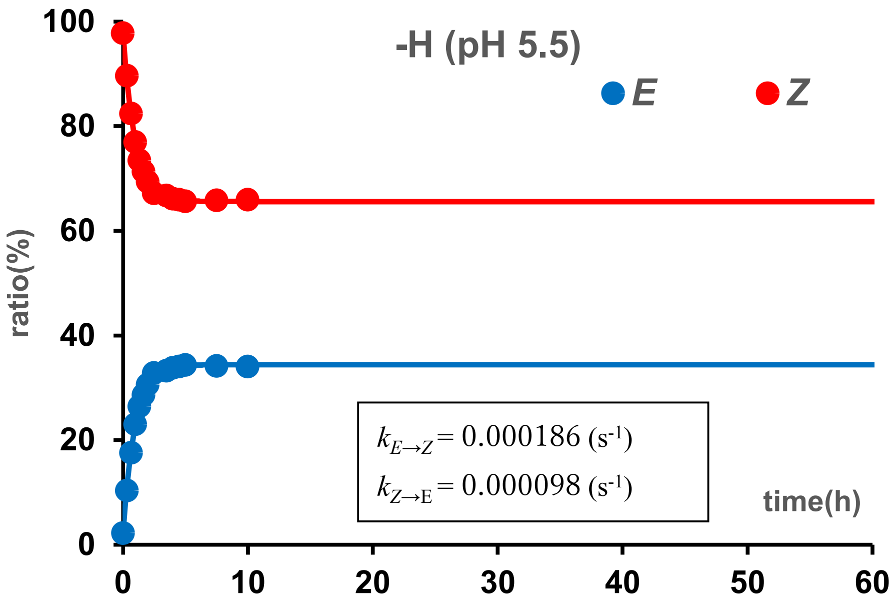


**Conditions: 30 °C in 200 mM acetate buffer/MeCN = 95/5**

**ratio**

| time (h) | *E* | *Z* |
|---|---|---|
| 0 | 2.21 | 97.79 |
| 0.33 | 10.35 | 89.65 |
| 0.67 | 17.57 | 82.43 |
| 1 | 23.02 | 76.98 |
| 1.33 | 26.49 | 73.51 |
| 1.67 | 28.66 | 71.34 |
| 2 | 30.62 | 69.38 |
| 2.5 | 32.83 | 67.17 |
| 3.5 | 33.26 | 66.74 |
| 4 | 33.84 | 66.16 |
| 4.5 | 34.04 | 65.96 |
| 5 | 34.37 | 65.63 |
| 7.5 | 34.16 | 65.84 |
| 10 | 34.03 | 65.97 |
| 20 | 34.20 | 65.80 |

**Figure S5.11**. Isomerization of compound **4** at pH 5.5

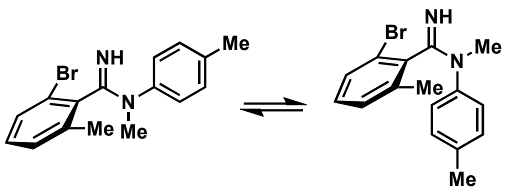


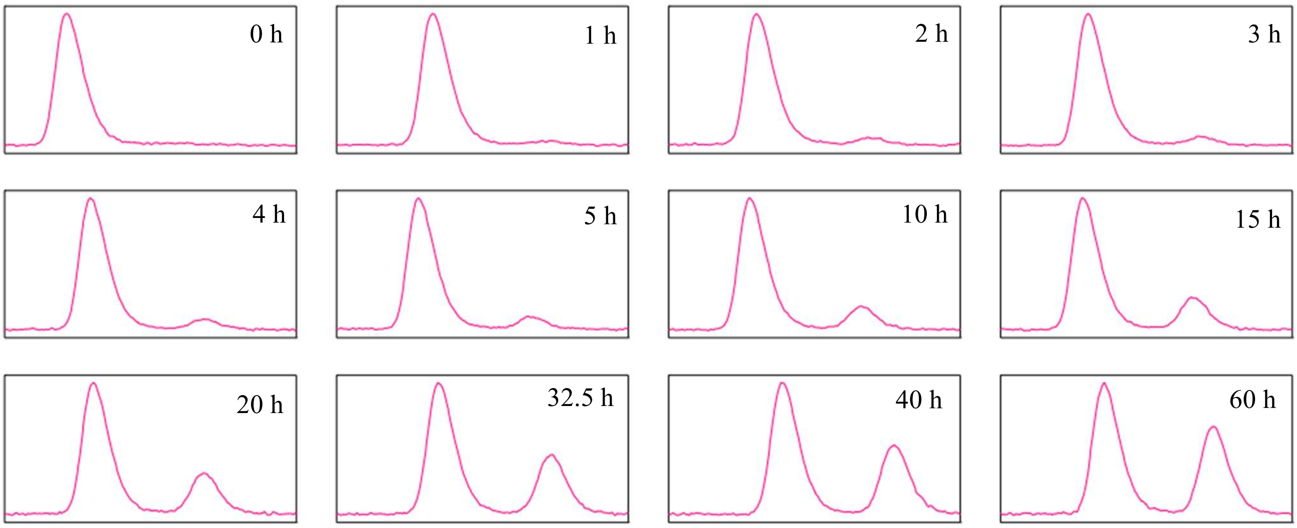


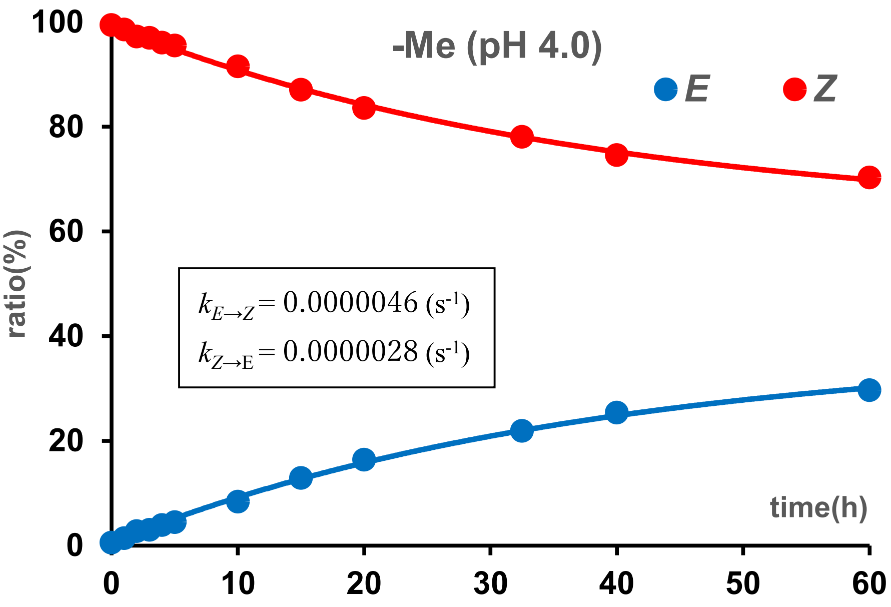


**ratio**

| time (h) | *E* | *Z* |
|---|---|---|
| 0 | 0.57 | 99.43 |
| 1 | 1.41 | 98.59 |
| 2 | 2.73 | 97.27 |
| 3 | 2.97 | 97.03 |
| 4 | 3.89 | 96.11 |
| 5 | 4.49 | 95.51 |
| 10 | 8.41 | 91.59 |
| 15 | 12.89 | 87.11 |
| 20 | 16.40 | 83.60 |
| 32.5 | 21.90 | 78.10 |
| 40 | 25.40 | 74.60 |
| 60 | 29.69 | 70.31 |

**Conditions: 30 °C in 200 mM acetate buffer/MeCN = 95/5**

**Figure S5.12**. Isomerization of compound **5** at pH 4.0

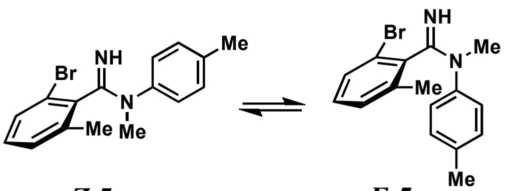


*Z*-**5** *E*-**5**

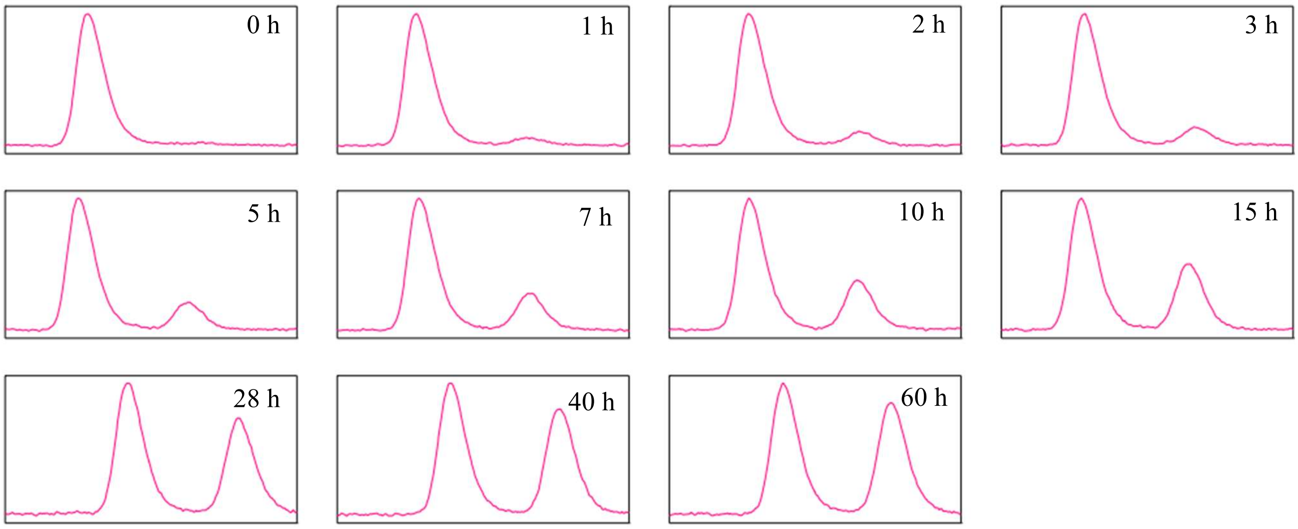


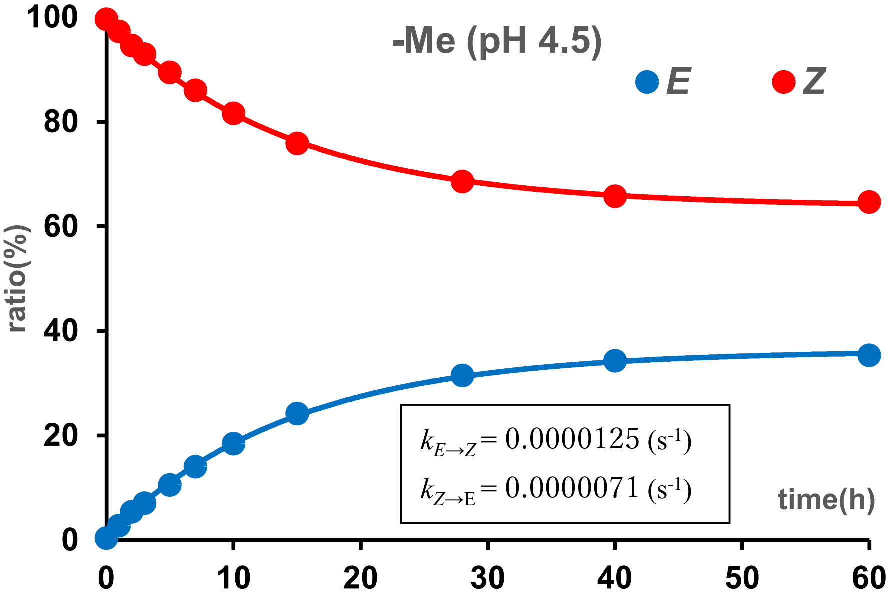


**ratio**

| time (h) | *E* | *Z* |
|---|---|---|
| 0 | 0.39 | 99.61 |
| 1 | 2.73 | 97.27 |
| 2 | 5.41 | 94.59 |
| 3 | 7.07 | 92.93 |
| 5 | 10.56 | 89.44 |
| 7 | 14.01 | 85.99 |
| 10 | 18.43 | 81.57 |
| 15 | 24.16 | 75.84 |
| 28 | 31.46 | 68.54 |
| 40 | 34.28 | 65.72 |
| 60 | 35.33 | 64.67 |

**Conditions: 30 °C in 200 mM acetate buffer/MeCN = 95/5**

**Figure S5.13**. Isomerization of compound **5** at pH 4.5

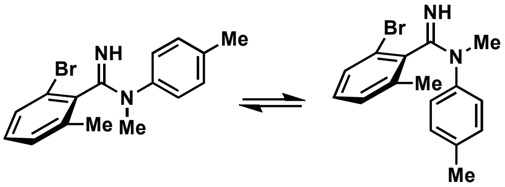


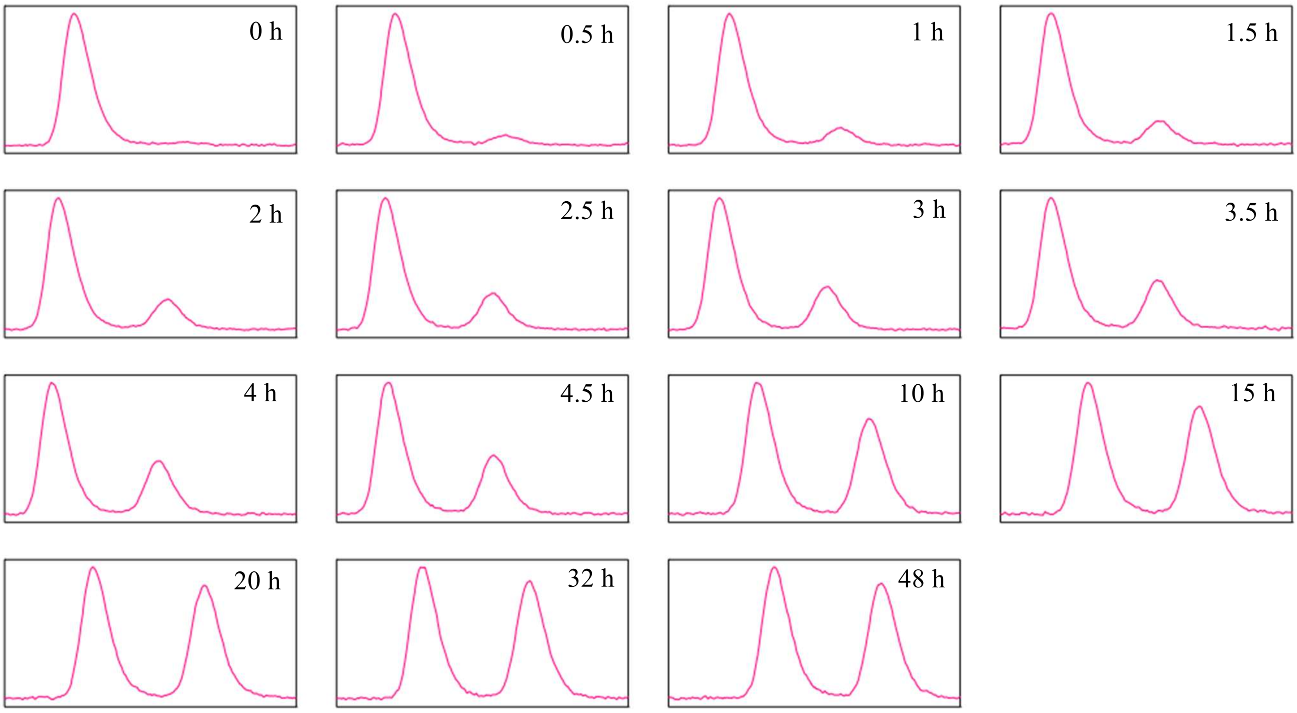


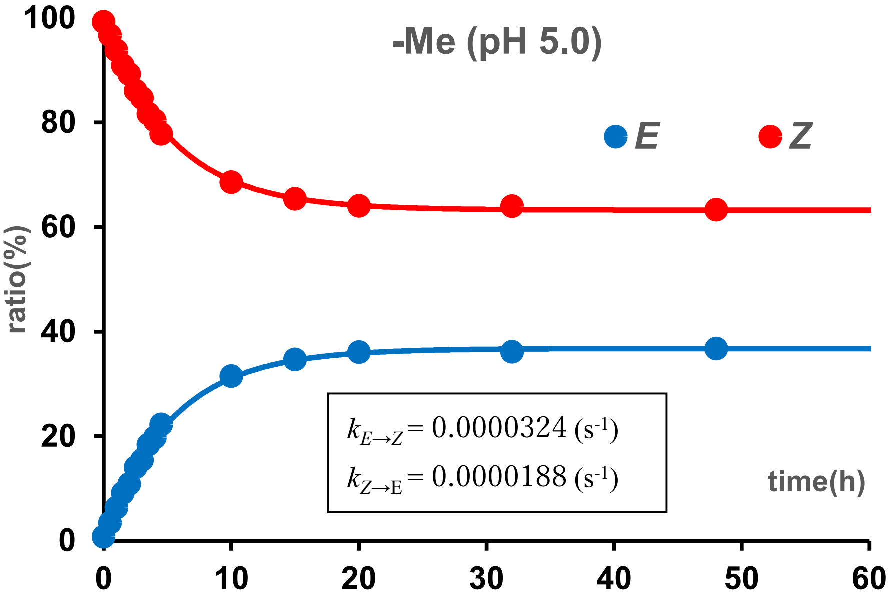


**Conditions: 30 °C in 200 mM acetate buffer/MeCN = 95/5**

**ratio**

| time (h) | *E* | *Z* |
|---|---|---|
| 0 | 0.79 | 99.21 |
| 0.5 | 3.40 | 96.60 |
| 1 | 6.26 | 93.74 |
| 1.5 | 9.10 | 90.90 |
| 2 | 10.80 | 89.20 |
| 2.5 | 13.97 | 86.03 |
| 3 | 15.39 | 84.61 |
| 3.5 | 18.35 | 81.65 |
| 4 | 19.67 | 80.33 |
| 4.5 | 22.21 | 77.79 |
| 10 | 31.44 | 68.56 |
| 15 | 34.64 | 65.36 |
| 20 | 36.00 | 64.00 |
| 32 | 36.09 | 63.91 |
| 48 | 36.72 | 63.28 |

**Figure S5.14**. Isomerization of compound **5** at pH 5.0

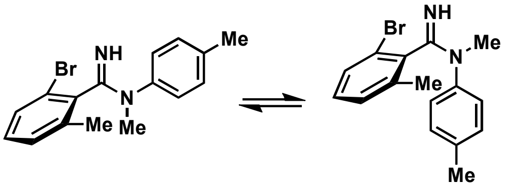


*Z*-5 *E*-5

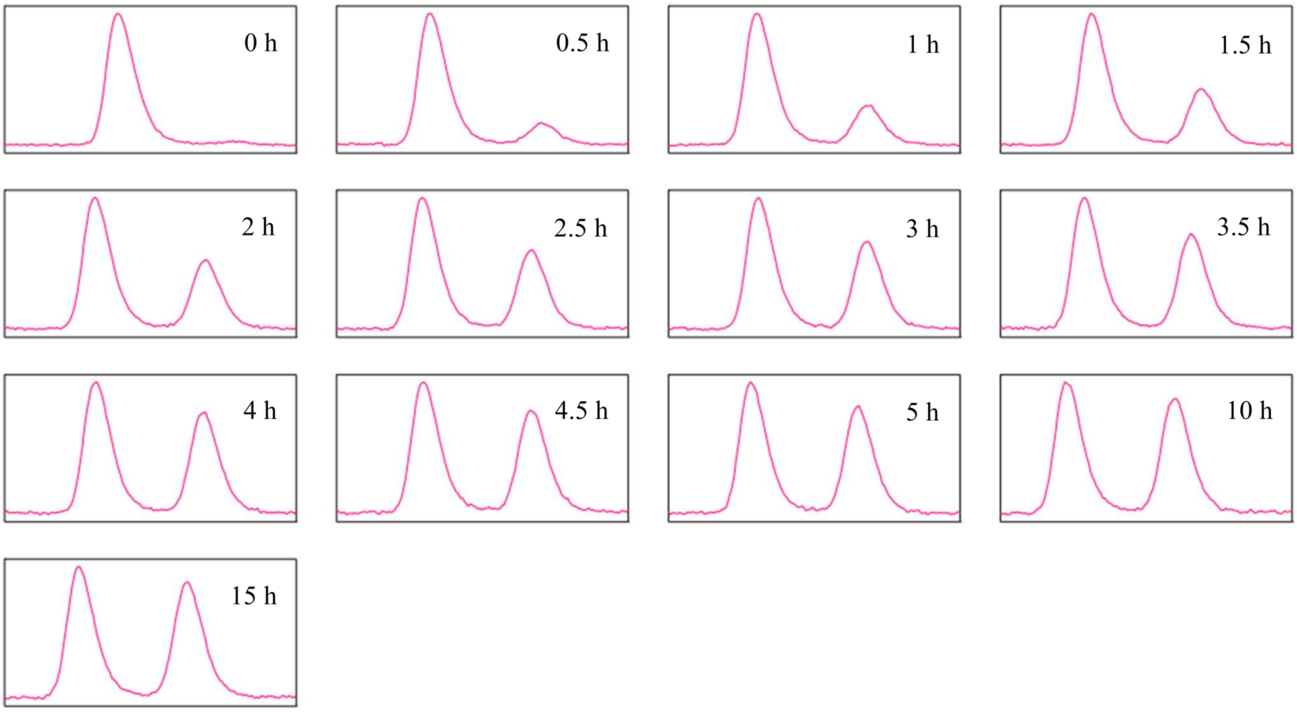


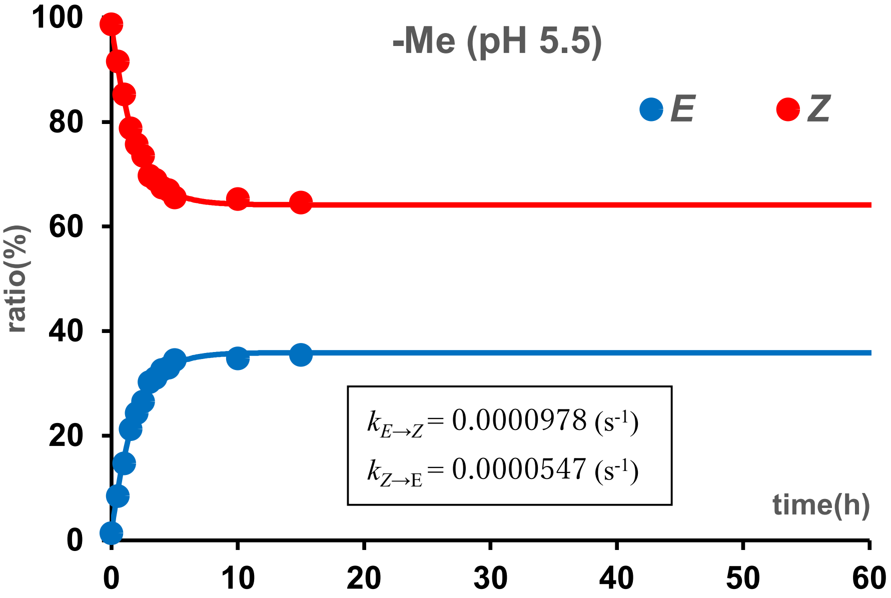


**ratio**

| time (h) | *E* | *Z* |
|---|---|---|
| 0 | 1.33 | 98.67 |
| 0.5 | 8.45 | 91.55 |
| 1 | 14.71 | 85.29 |
| 1.5 | 21.24 | 78.76 |
| 2 | 24.31 | 75.69 |
| 2.5 | 26.51 | 73.49 |
| 3 | 30.25 | 69.75 |
| 3.5 | 31.06 | 68.94 |
| 4 | 32.55 | 67.45 |
| 4.5 | 33.01 | 66.99 |
| 5 | 34.47 | 65.53 |
| 10 | 34.78 | 65.22 |
| 15 | 35.43 | 64.57 |

**Conditions: 30 °C in 200 mM acetate buffer/MeCN = 95/5**

**Figure S5.15**. Isomerization of compound **5** at pH 5.5

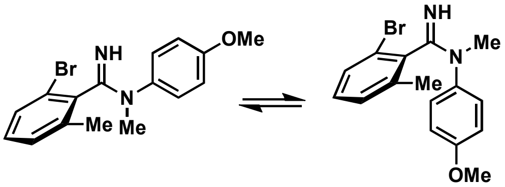


***Z*-6** ***E*-6**

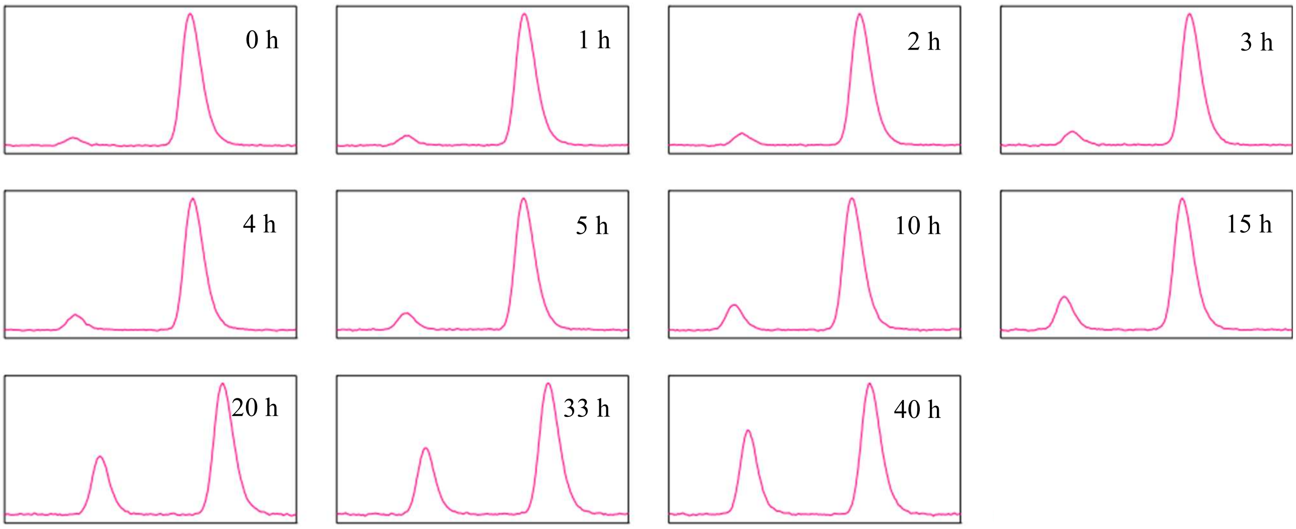


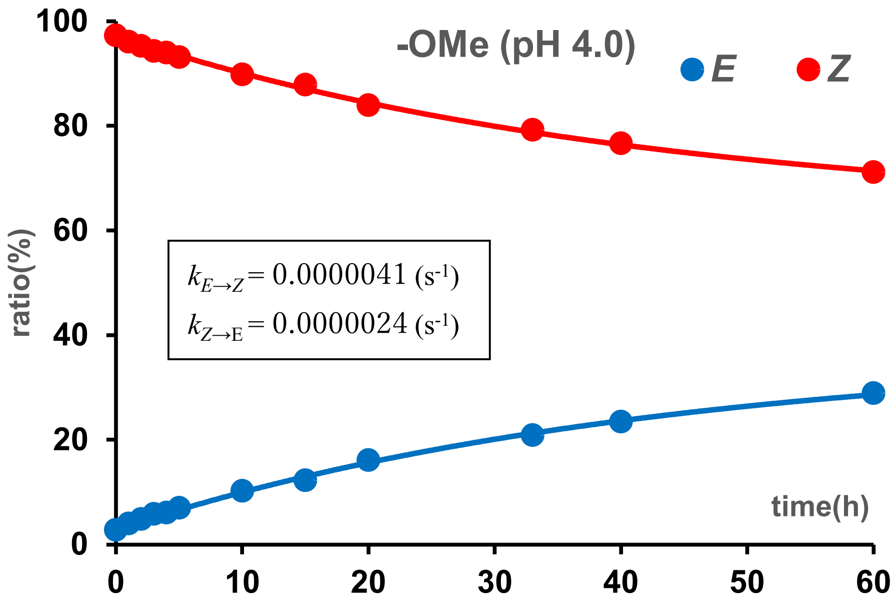


**ratio**

| time (h) | *E* | *Z* |
|---|---|---|
| 0 | 2.78 | 97.22 |
| 1 | 3.98 | 96.02 |
| 2 | 4.81 | 95.19 |
| 3 | 5.79 | 94.21 |
| 4 | 6.06 | 93.94 |
| 5 | 6.92 | 93.08 |
| 10 | 10.22 | 89.78 |
| 15 | 12.20 | 87.80 |
| 20 | 16.09 | 83.91 |
| 33 | 20.82 | 79.18 |
| 40 | 23.41 | 76.59 |
| 60 | 28.88 | 71.12 |

**Conditions: 30 °C in 200 mM acetate buffer/MeCN = 95/5**

**Figure S5.16**. Isomerization of compound **6** at pH 4.0

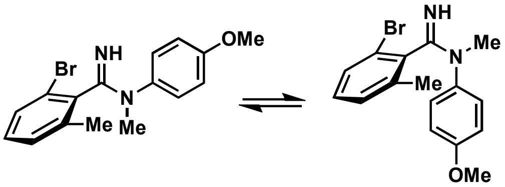


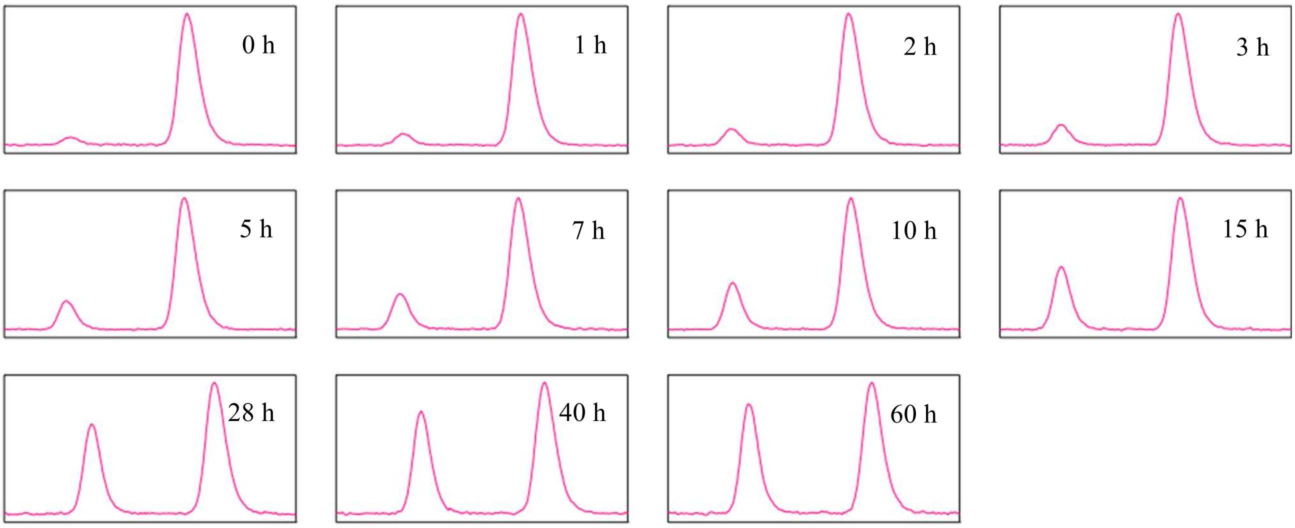


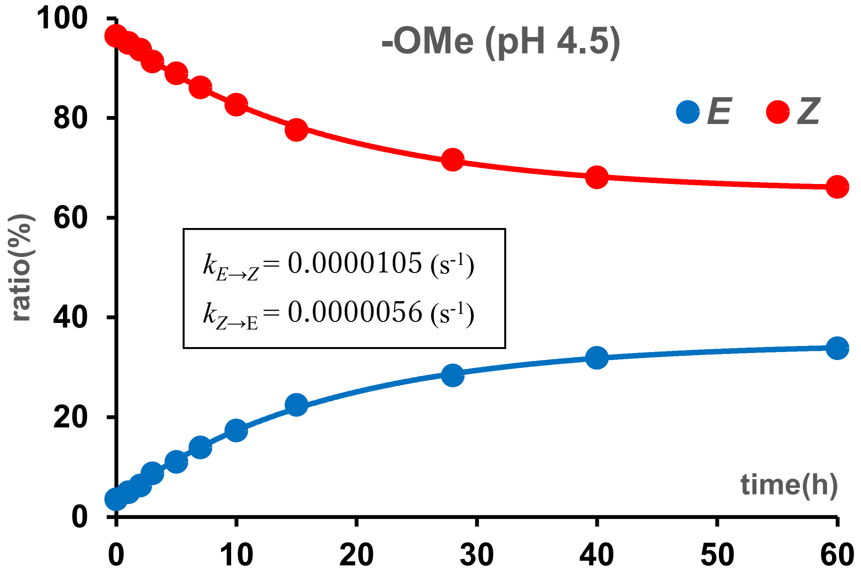


**ratio**

| time (h) | *E* | *Z* |
|---|---|---|
| 0 | 3.52 | 96.48 |
| 1 | 4.94 | 95.06 |
| 2 | 6.29 | 93.71 |
| 3 | 8.69 | 91.31 |
| 5 | 11.03 | 88.97 |
| 7 | 13.91 | 86.09 |
| 10 | 17.35 | 82.65 |
| 15 | 22.43 | 77.57 |
| 28 | 28.37 | 71.63 |
| 40 | 31.90 | 68.10 |
| 60 | 33.84 | 66.16 |

**Conditions: 30 °C in 200 mM acetate buffer/MeCN = 95/5**

**Figure S5.17**. Isomerization of compound **6** at pH 4.5

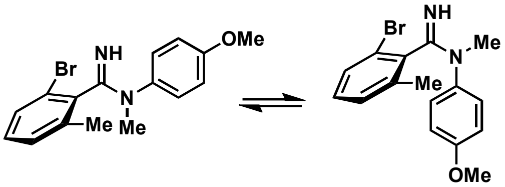


***Z*-6** ***E*-6**

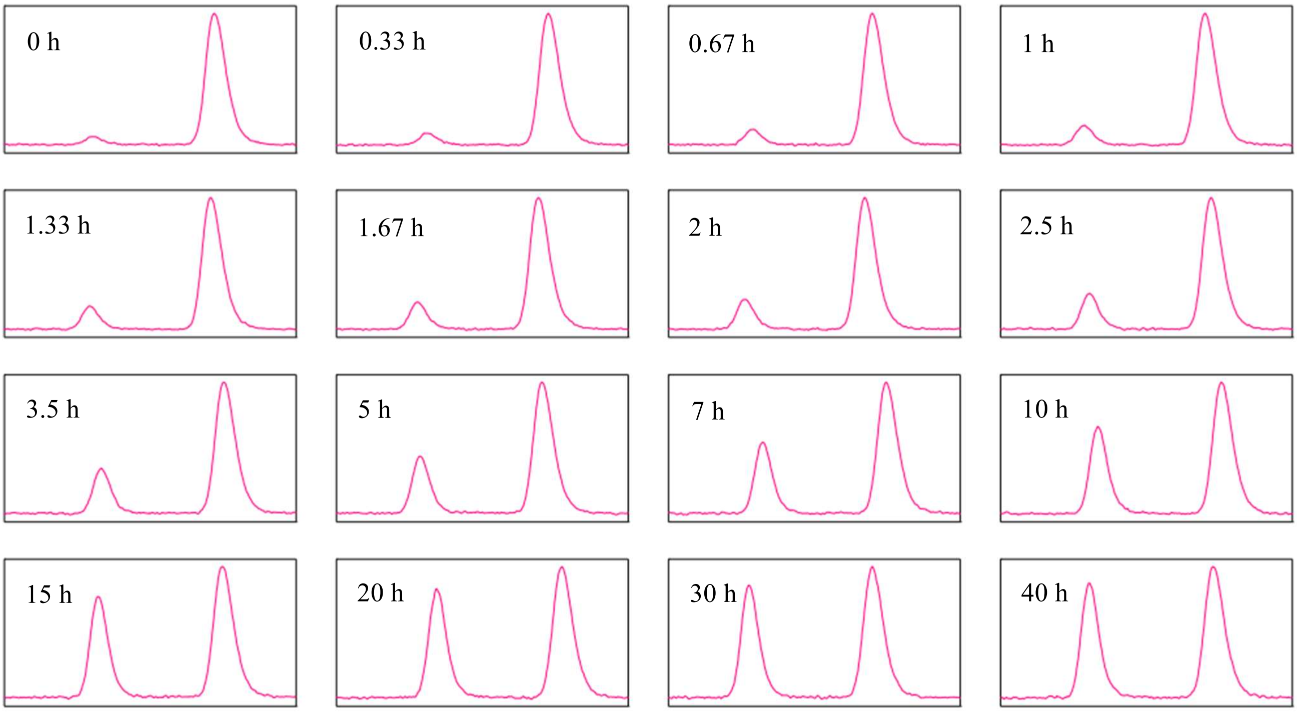


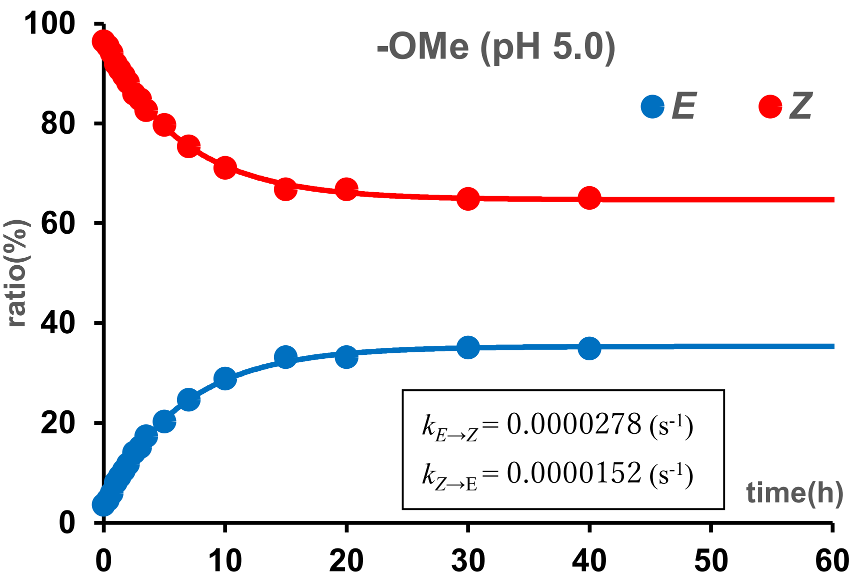


**ratio**

| time (h) | *E* | *Z* |
|---|---|---|
| 0 | 3.57 | 96.43 |
| 0.33 | 4.53 | 95.47 |
| 0.67 | 5.89 | 94.11 |
| 1 | 8.06 | 91.94 |
| 1.33 | 9.23 | 90.77 |
| 1.67 | 10.45 | 89.55 |
| 2 | 11.75 | 88.25 |
| 2.5 | 14.08 | 85.92 |
| 3.5 | 17.35 | 82.65 |
| 5 | 20.30 | 79.70 |
| 7 | 24.63 | 75.37 |
| 10 | 28.88 | 71.12 |
| 15 | 33.18 | 66.82 |
| 20 | 33.20 | 66.80 |
| 30 | 35.11 | 64.89 |
| 40 | 34.93 | 65.07 |

**Conditions: 30 °C in 200 mM acetate buffer/MeCN = 95/5**

**Figure S5.18**. Isomerization of compound **6** at pH 5.0

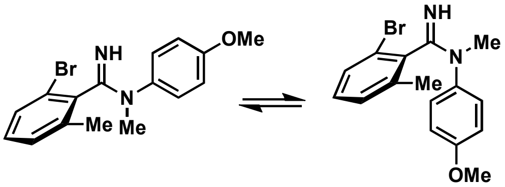


***Z*-6** ***E*-6**

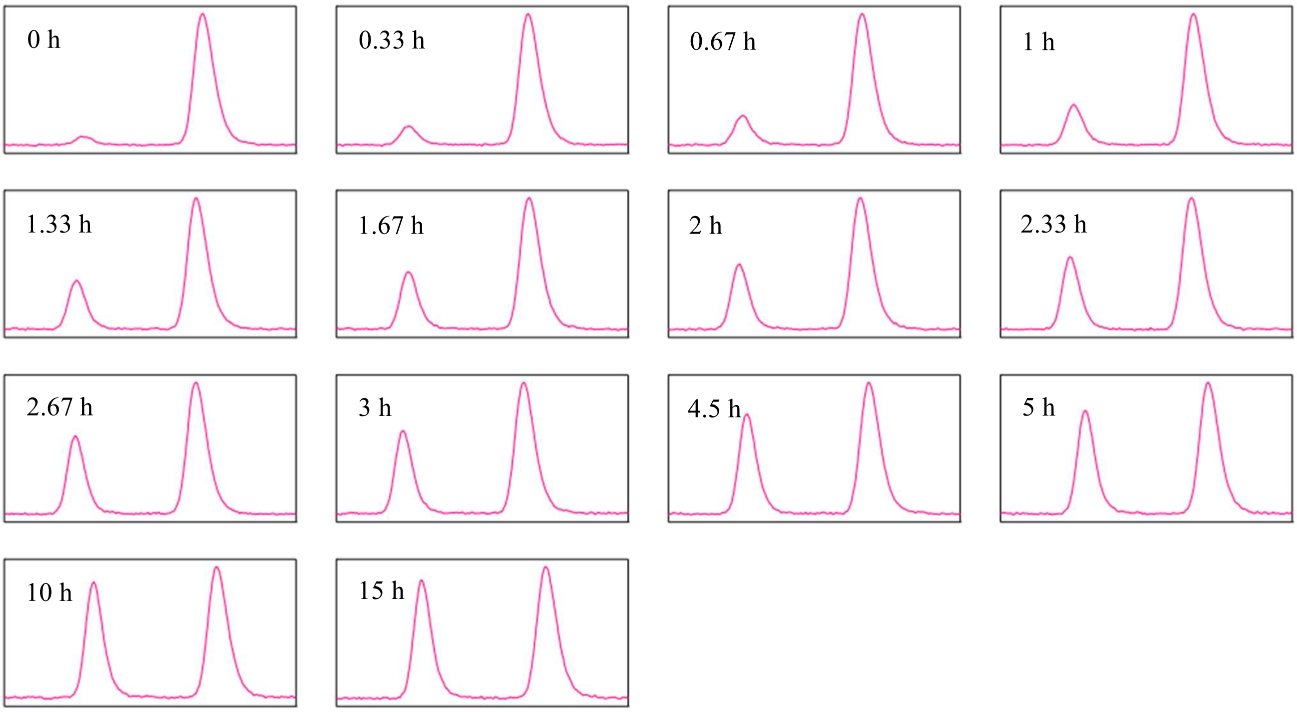


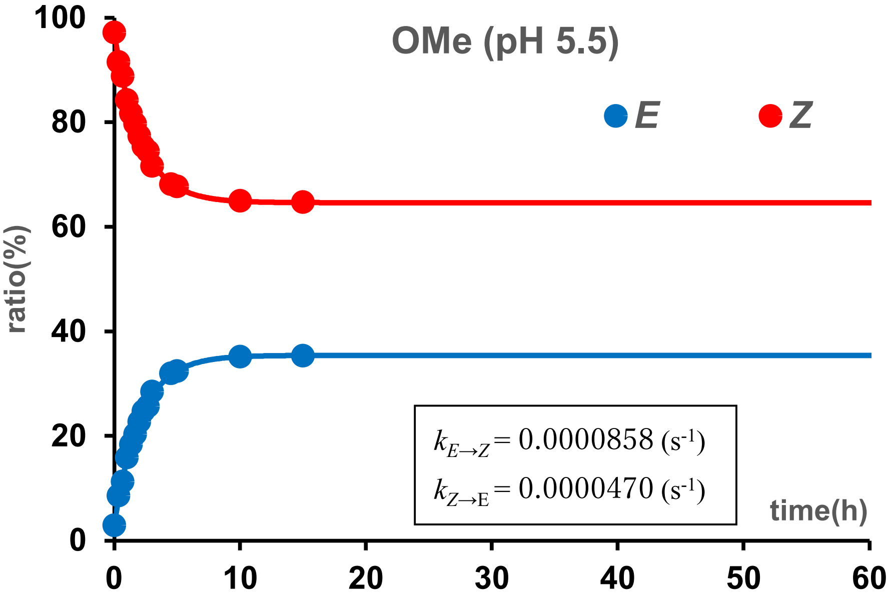


**Conditions: 30 °C in 200 mM acetate buffer/MeCN = 95/5**

**ratio**

| time (h) | *E* | *Z* |
|---|---|---|
| 0 | 2.90 | 97.10 |
| 0.33 | 8.51 | 91.49 |
| 0.67 | 11.24 | 88.76 |
| 1 | 15.80 | 84.20 |
| 1.33 | 18.35 | 81.65 |
| 1.67 | 20.32 | 79.68 |
| 2 | 22.67 | 77.33 |
| 2.33 | 24.71 | 75.29 |
| 2.67 | 25.60 | 74.40 |
| 3 | 28.41 | 71.59 |
| 4.5 | 31.92 | 68.08 |
| 5 | 32.33 | 67.67 |
| 10 | 35.11 | 64.89 |
| 15 | 35.31 | 64.69 |

**Figure S5.19**. Isomerization of compound **6** at pH 5.5

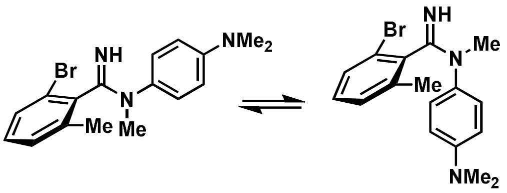


***Z*-7** ***E*-7**

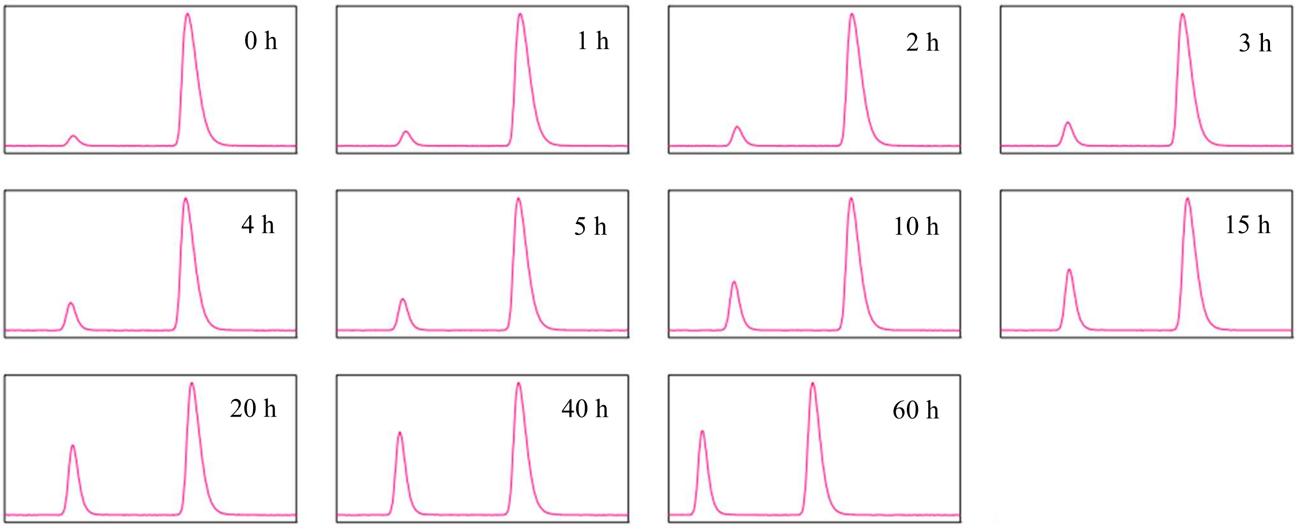


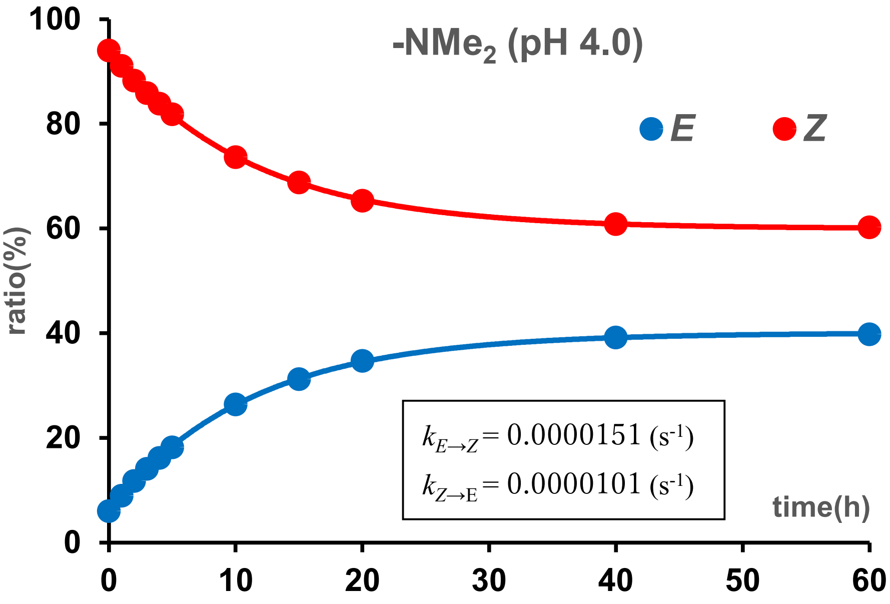


**ratio**

| time (h) | $E$ | $Z$ |
|---|---|---|
| 0 | 5.96 | 94.04 |
| 1 | 8.94 | 91.06 |
| 2 | 11.72 | 88.28 |
| 3 | 14.10 | 85.90 |
| 4 | 16.16 | 83.84 |
| 5 | 18.19 | 81.81 |
| 10 | 26.36 | 73.64 |
| 15 | 31.22 | 68.78 |
| 20 | 34.71 | 65.29 |
| 40 | 39.15 | 60.85 |
| 60 | 39.78 | 60.22 |

**Conditions: 30 °C in 200 mM acetate buffer/MeCN = 95/5**

**Figure S5.20**. Isomerization of compound **7** at pH 4.0

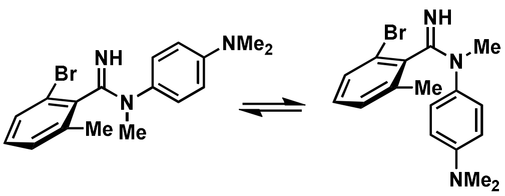


***Z*-7** ***E*-7**

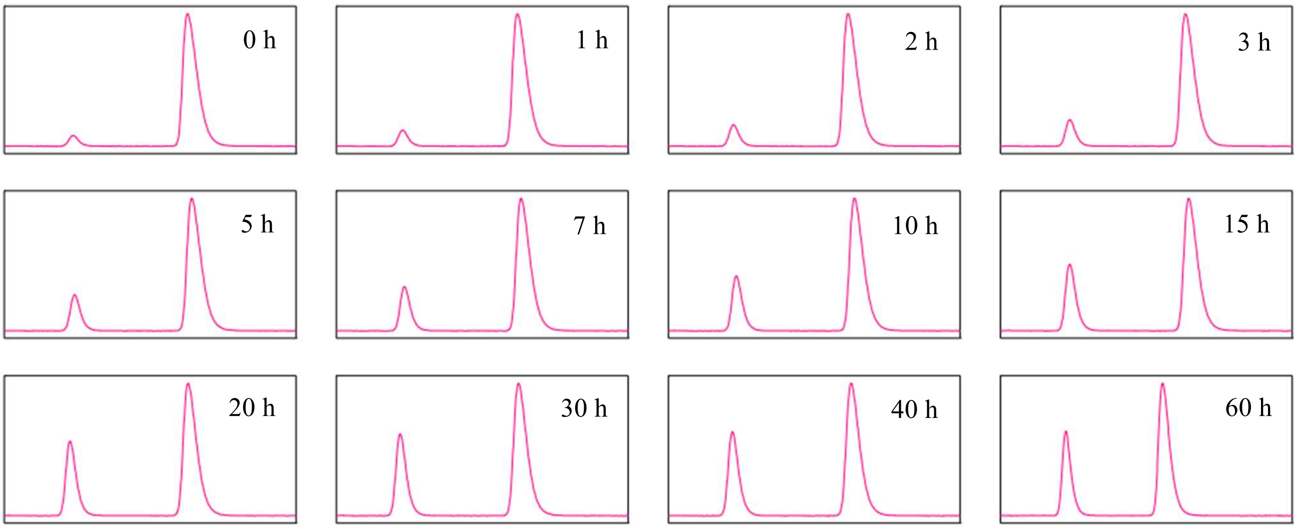


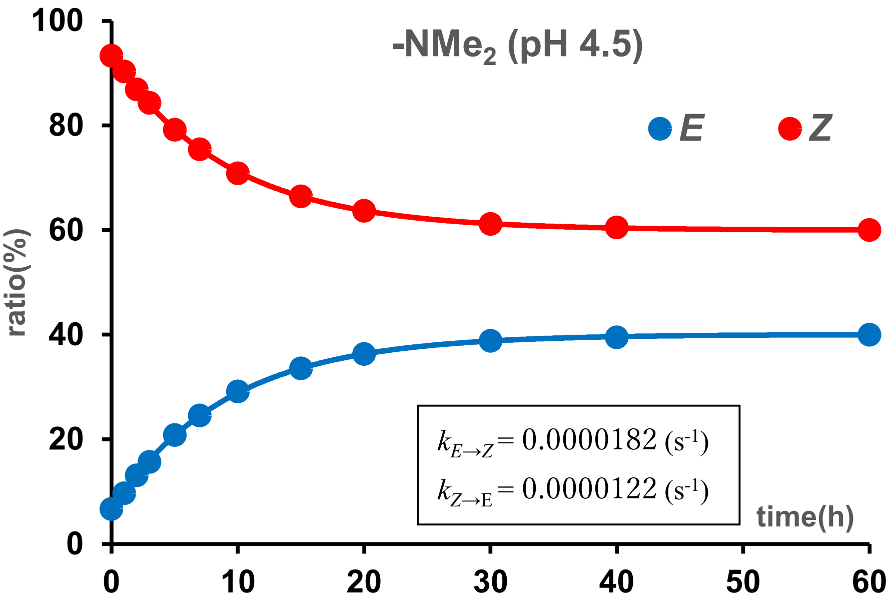


**ratio**

| time (h) | *E* | *Z* |
|---|---|---|
| 0 | 6.67 | 93.33 |
| 1 | 9.69 | 90.31 |
| 2 | 13.11 | 86.89 |
| 3 | 15.67 | 84.33 |
| 5 | 20.85 | 79.15 |
| 7 | 24.59 | 75.41 |
| 10 | 29.13 | 70.87 |
| 15 | 33.57 | 66.43 |
| 20 | 36.32 | 63.68 |
| 30 | 38.85 | 61.15 |
| 40 | 39.51 | 60.49 |
| 60 | 39.97 | 60.03 |

**Conditions: 30 °C in 200 mM acetate buffer/MeCN = 95/5**

**Figure S5.21**. Isomerization of compound **7** at pH 4.5

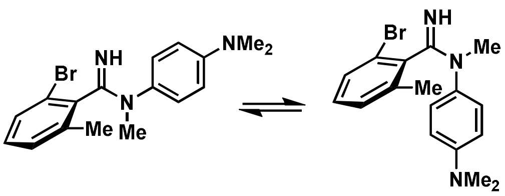


***Z*-7** ***E*-7**

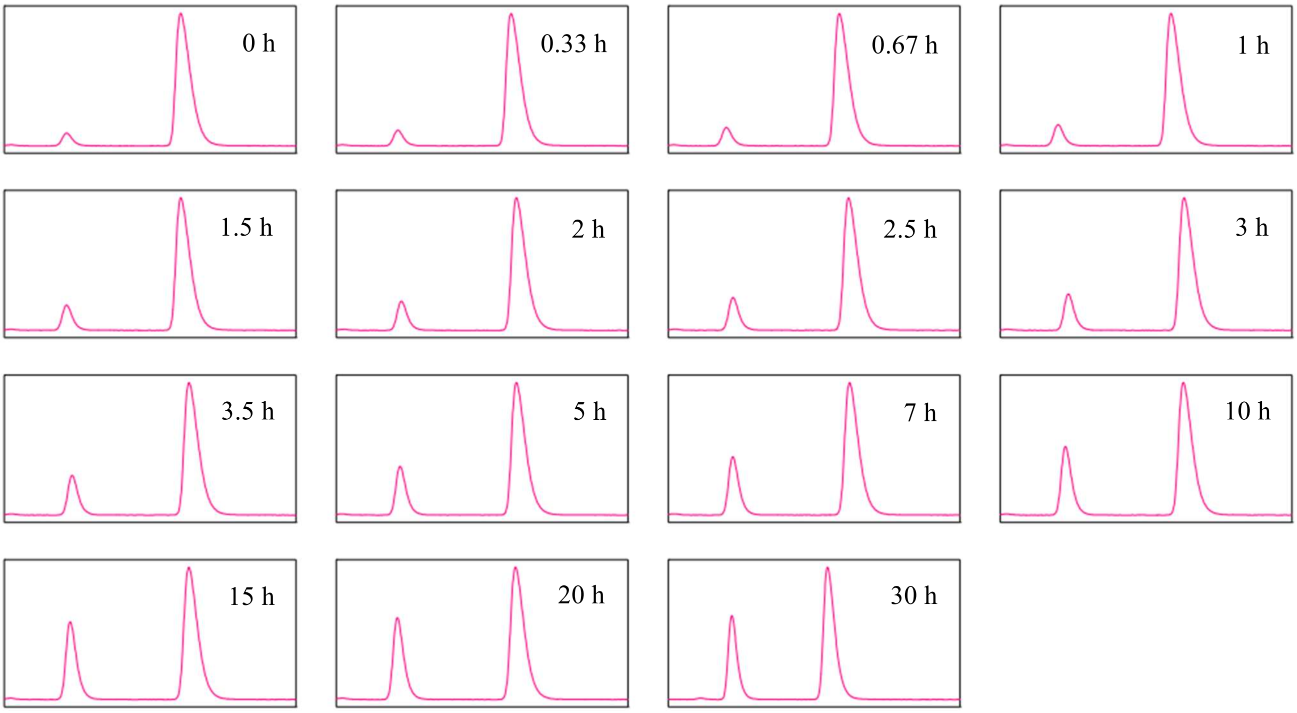


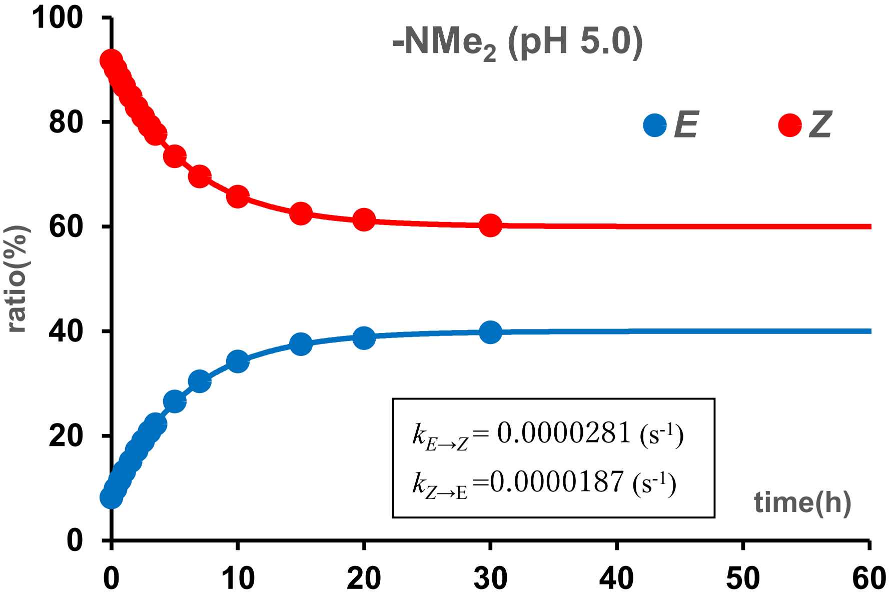


**ratio**

| time (h) | *E* | *Z* |
|---|---|---|
| 0 | 8.23 | 91.77 |
| 0.33 | 9.83 | 90.17 |
| 0.67 | 11.53 | 88.47 |
| 1 | 13.16 | 86.84 |
| 1.5 | 15.11 | 84.89 |
| 2 | 17.18 | 82.82 |
| 2.5 | 18.95 | 81.05 |
| 3 | 20.74 | 79.26 |
| 3.5 | 22.24 | 77.76 |
| 5 | 26.58 | 73.42 |
| 7 | 30.42 | 69.58 |
| 10 | 34.24 | 65.76 |
| 15 | 37.50 | 62.50 |
| 20 | 38.69 | 61.31 |
| 30 | 39.79 | 60.21 |

**Conditions: 30 °C in 200 mM acetate buffer/MeCN = 95/5**

**Figure S5.22**. Isomerization of compound **7** at pH 5.0

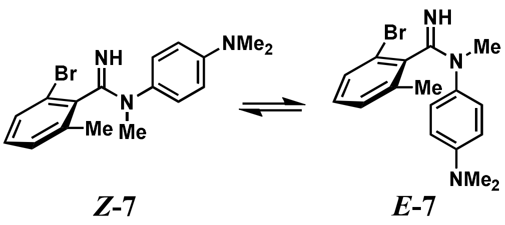


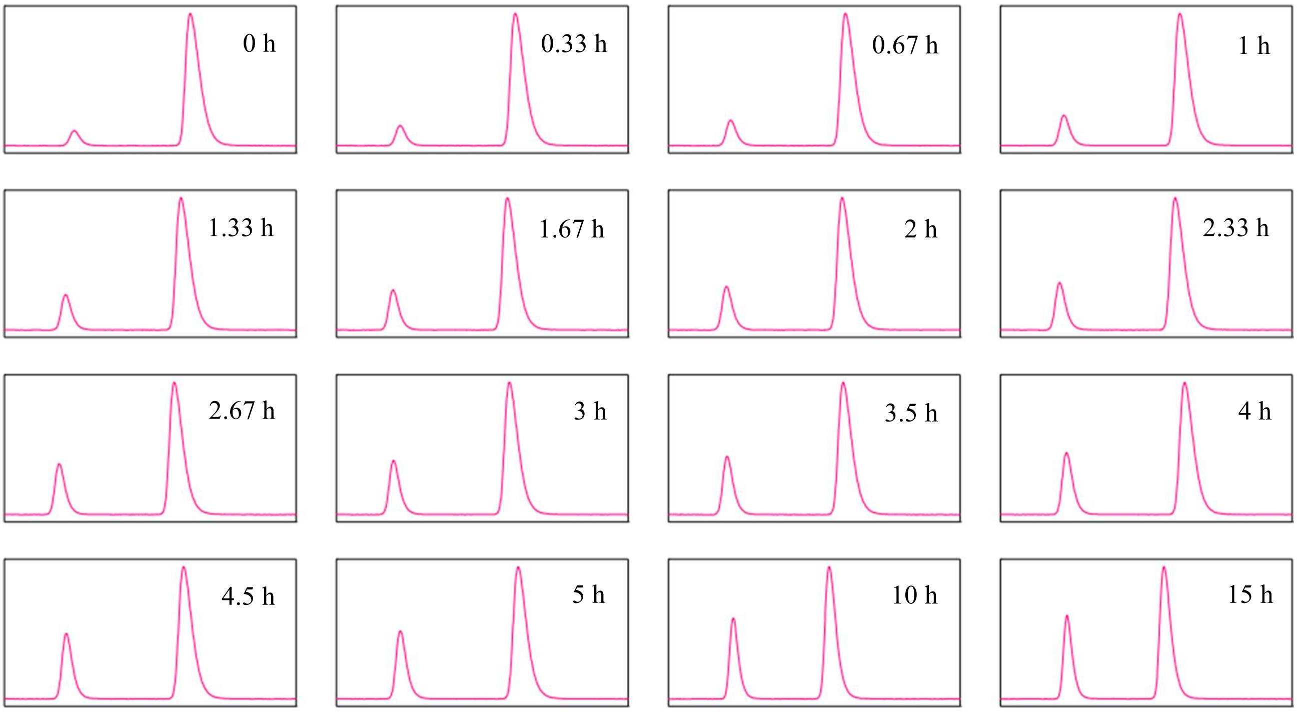


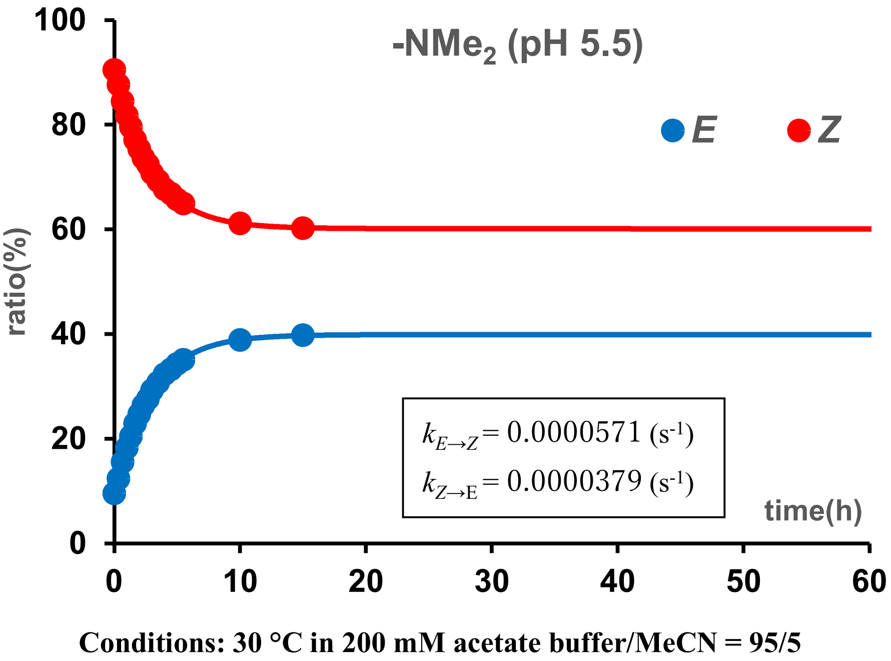


**ratio**

| time (h) | *E* | *Z* |
|---|---|---|
| 0 | 9.52 | 90.48 |
| 0.33 | 12.36 | 87.64 |
| 0.67 | 15.54 | 84.46 |
| 1 | 18.22 | 81.78 |
| 1.33 | 20.46 | 79.54 |
| 1.67 | 22.86 | 77.14 |
| 2 | 24.66 | 75.34 |
| 2.33 | 26.39 | 73.61 |
| 2.67 | 27.59 | 72.41 |
| 3 | 29.27 | 70.73 |
| 3.5 | 30.70 | 69.30 |
| 4 | 32.32 | 67.68 |
| 4.5 | 33.20 | 66.80 |
| 5 | 34.28 | 65.72 |
| 10 | 38.85 | 61.15 |
| 15 | 39.79 | 60.21 |

**Figure S5.23**. Isomerization of compound **7** at pH 5.5

## 6. Monitoring of the racemization

Prior to monitoring of the racemization, racemic amidine **1** was purified by chiral RP-HPLC to afford each enantiomer, which was used to the following investigation (Figure S6.1-6.3).

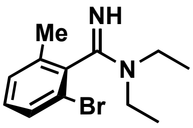


**Chiral RP-HPLC conditions:**

**column: CHIRALPAK IK, 5μm, 4.6 mmI.D.×250 mmL;**

**eluent: 0.5% TFA in $H_2O$/MeCN = 80/20;**

**flow rate: 1.5 mL/min; detection: UV 254 nm**

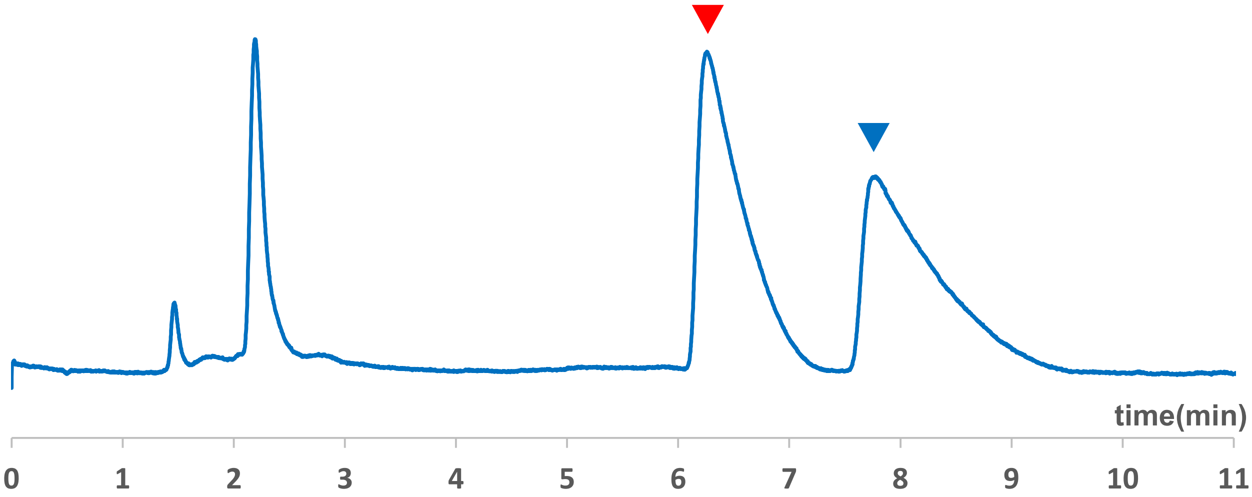


**Figure S6.1**. Chiral HPLC chart of racemic compound **1**

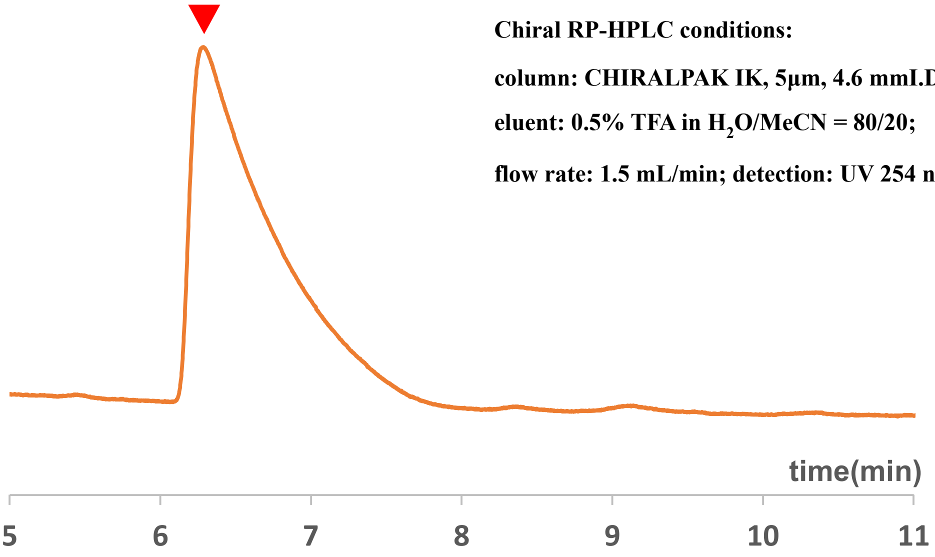


**Chiral RP-HPLC conditions:**

**column: CHIRALPAK IK, 5μm, 4.6 mmI.D.×250 mmL;**

**eluent: 0.5% TFA in $H_2O$/MeCN = 80/20;**

**flow rate: 1.5 mL/min; detection: UV 254 nm**

**Figure S6.2**. Reanalysis of the 1st eluted enantiomer of compound **1**

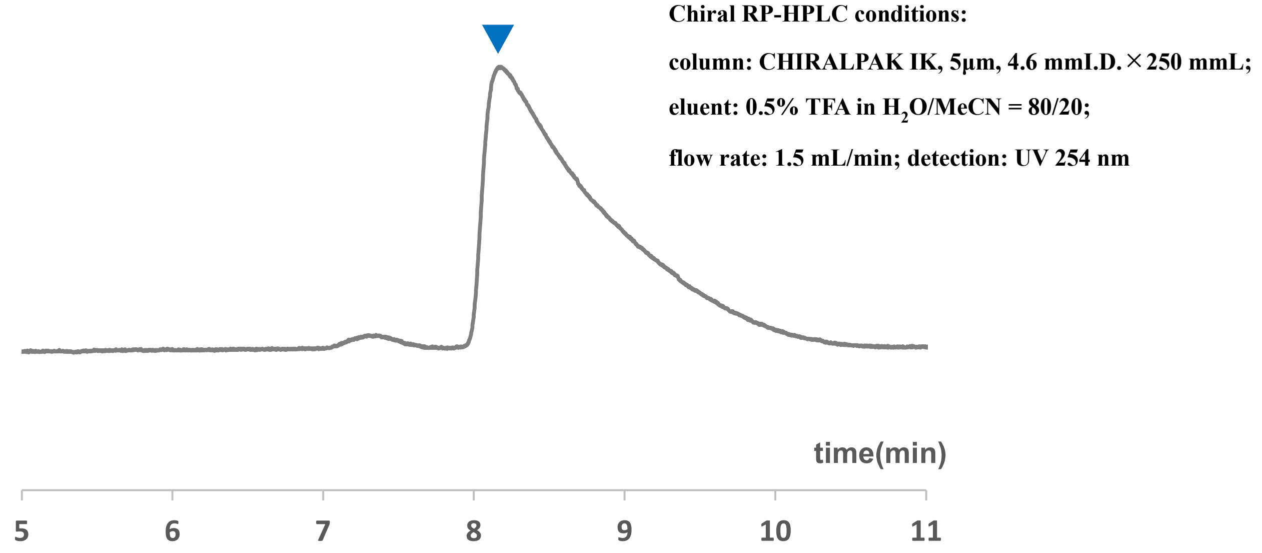


**Figure S6.3**. Reanalysis of the 2nd eluted enantiomer of compound **1**

After the separation of each enantiomer, a solution of the enantiomer that elutes first was concentrated in *vacuo*, and diluted with a buffer (pH =9.2, 10.3, 11,7 or 12.7) and MeCN (final ratio: buffer/MeCN = 9/1). The resulting solution was maintained at 70 °C in an incubator. The progress of the racemization was monitored by chiral RP-HPLC analysis (detailed conditions were described below).

The ratio of each isomer was plotted as a function of time, and curve-fitting analysis of the experimental results using differential equations describing the isomerization (eq. 4, 5), to calculate kinetic constant $k$ (Figure S6.4-6.7).

$$\frac{d[1st]}{dt} = k_{rac}[2nd] - k_{rac}[1st] \text{ (eq. 4)}$$

$$\frac{d([1st]+[2nd])}{dt} = 0 \text{ (eq. 5)}$$

**<u>Chiral RP-HPLC conditions for the racemization experiments</u>**

column: CHIRALPAK IK, 5µm, 4.6 mmI.D.×250 mmL, flow rate: 1.2 mL/min, eluent: A; 0.5% TFA in $H_2O$, B; MeCN, 30% B in A isocratic elusion, detection: UV (254 nm)

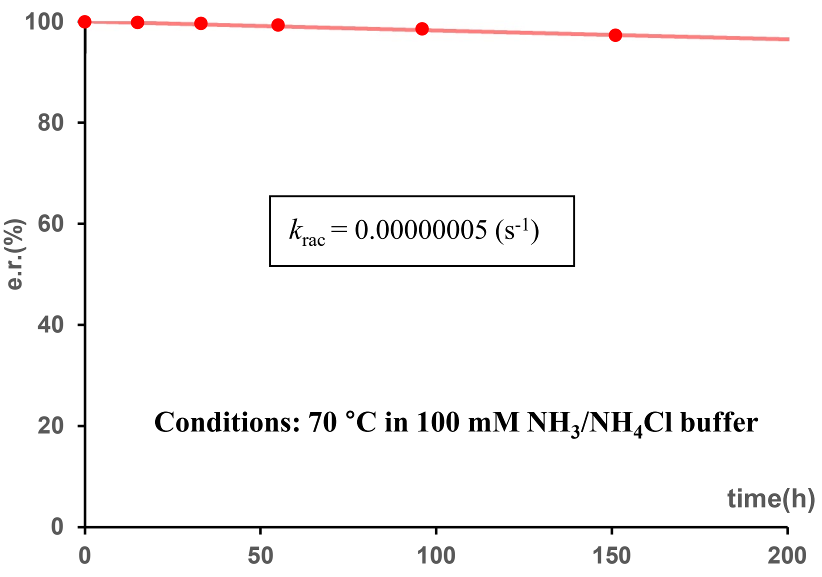


**ratio**

| time(h) | 1st | 2nd |
|---|---|---|
| 0 | 100.00 | 0.00 |
| 15 | 99.84 | 0.16 |
| 33 | 99.64 | 0.36 |
| 55 | 99.33 | 0.67 |
| 96 | 98.60 | 1.40 |
| 151 | 97.32 | 2.68 |
| 220 | 95.87 | 4.13 |
| 342 | 94.80 | 5.21 |

**Figure S6.4**. Racemization of compound **1** at pH 9.2

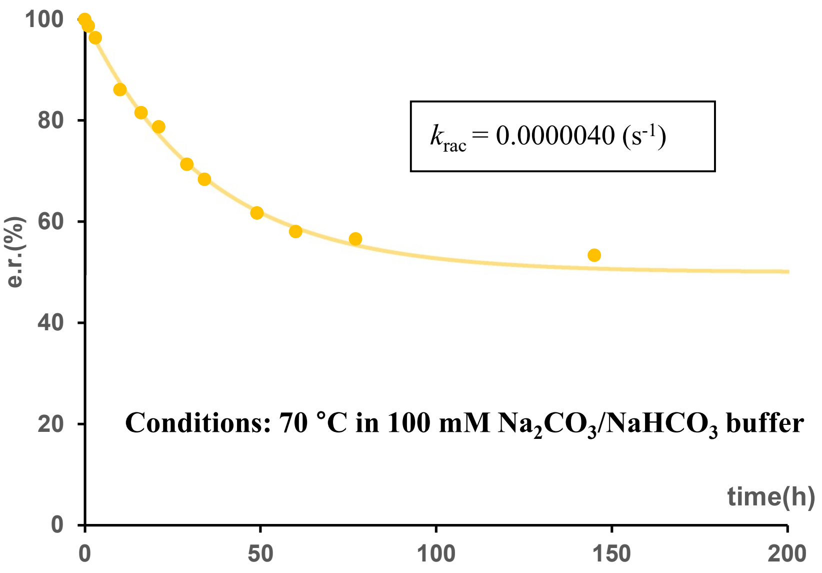


**ratio**

| time(h) | 1st | 2nd |
|---|---|---|
| 0 | 100.00 | 0.00 |
| 1 | 98.70 | 1.30 |
| 3 | 96.36 | 3.64 |
| 10 | 86.09 | 13.91 |
| 16 | 81.57 | 18.43 |
| 21 | 78.76 | 21.24 |
| 29 | 71.37 | 28.63 |
| 34 | 68.37 | 31.63 |
| 49 | 61.69 | 38.31 |
| 60 | 58.07 | 41.93 |
| 77 | 56.59 | 43.41 |
| 145 | 53.33 | 46.67 |

**Figure S6.5**. Racemization of compound **1** at pH 10.3

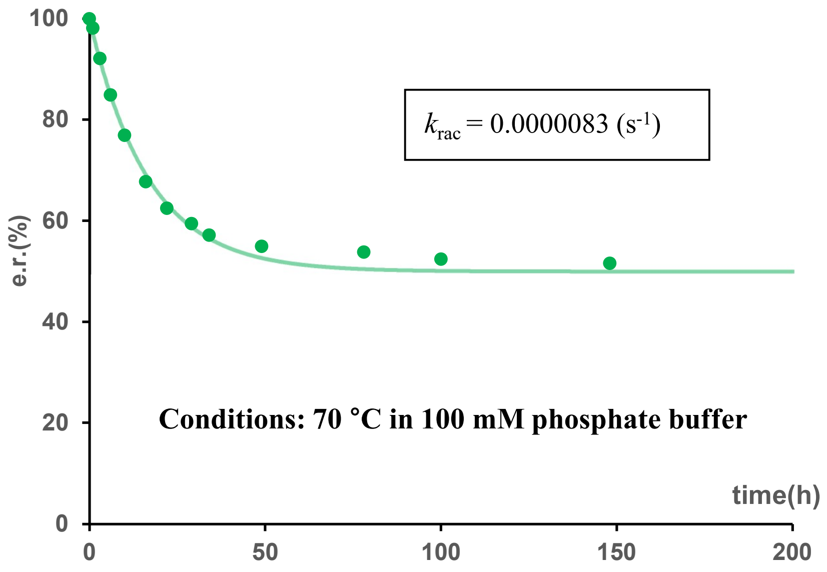


**ratio**

| time(h) | 1st | 2nd |
|---|---|---|
| 0 | 100.00 | 0.00 |
| 1 | 98.16 | 1.84 |
| 3 | 92.15 | 7.85 |
| 6 | 84.93 | 15.07 |
| 10 | 76.97 | 23.03 |
| 16 | 67.80 | 32.21 |
| 22 | 62.52 | 37.49 |
| 29 | 59.47 | 40.53 |
| 34 | 57.22 | 42.79 |
| 49 | 54.97 | 45.03 |
| 78 | 53.85 | 46.15 |
| 100 | 52.46 | 47.54 |
| 148 | 51.63 | 48.37 |

**Figure S6.6**. Racemization of compound **1** at pH 11.7

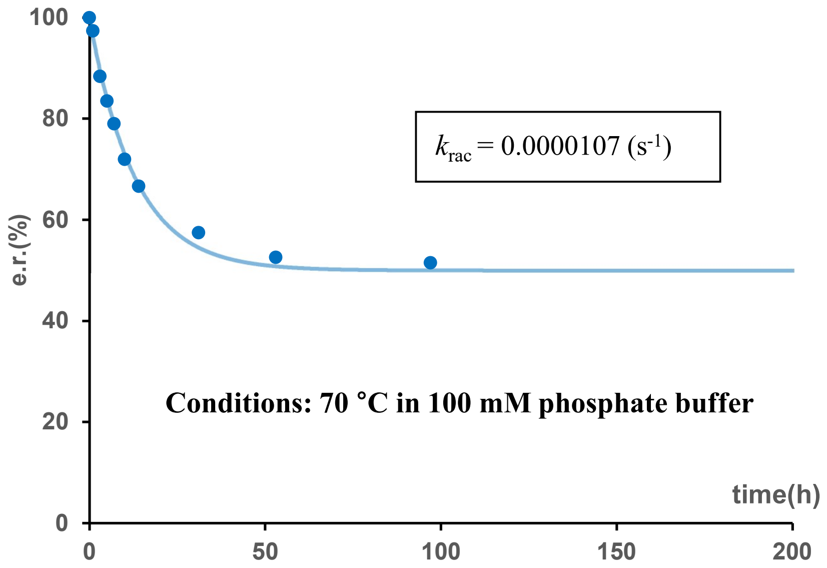


**ratio**

| time(h) | 1st | 2nd |
|---|---|---|
| 0 | 100.00 | 0.00 |
| 1 | 97.45 | 2.55 |
| 3 | 88.40 | 11.60 |
| 5 | 83.52 | 16.48 |
| 7 | 79.07 | 20.93 |
| 10 | 72.02 | 27.98 |
| 14 | 66.68 | 33.32 |
| 31 | 57.50 | 42.50 |
| 53 | 52.65 | 47.35 |
| 97 | 51.57 | 48.44 |

**Figure S6.7**. Racemization of compound **1** at pH 12.7

**Chiral RP-HPLC charts at each time point**

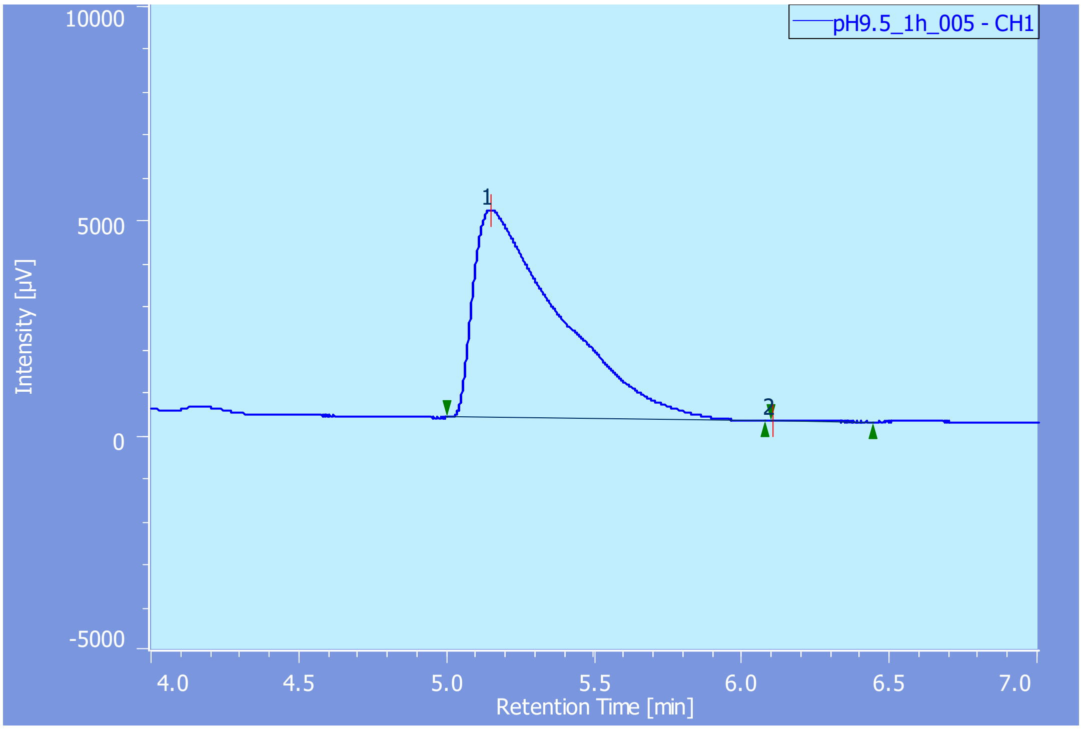


**Figure S6.8**. HPLC chart of **1** at pH 9.2 (0 h after heating)

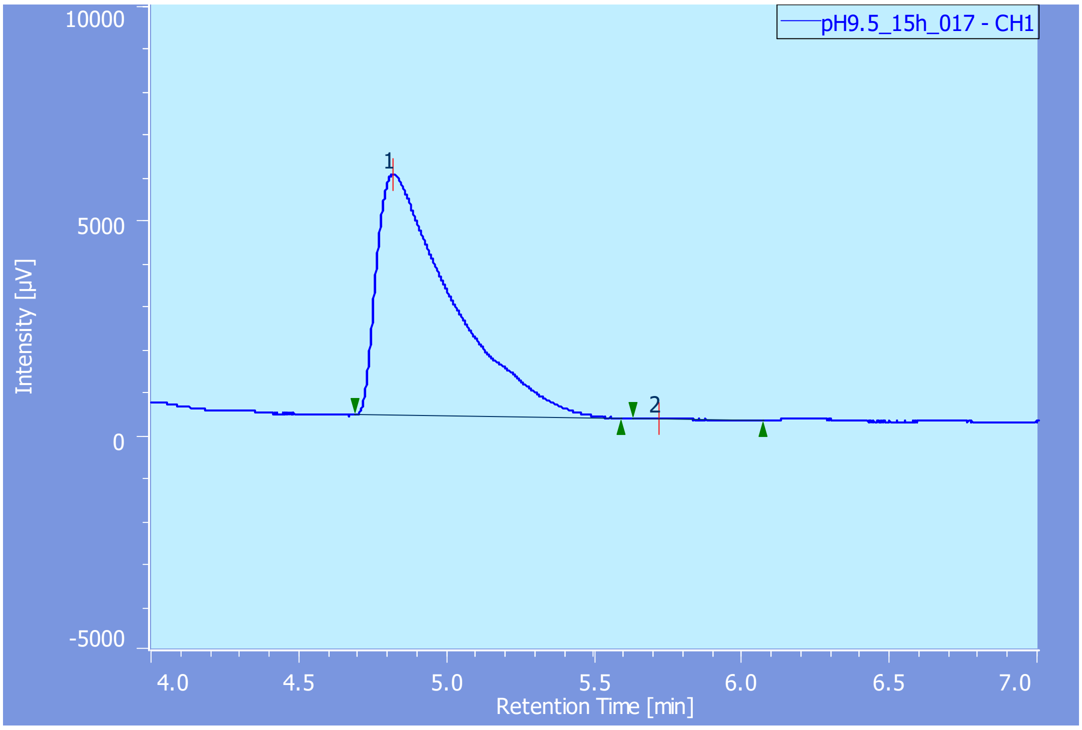


**Figure S6.9**. HPLC chart of **1** at pH 9.2 (15 h after heating)

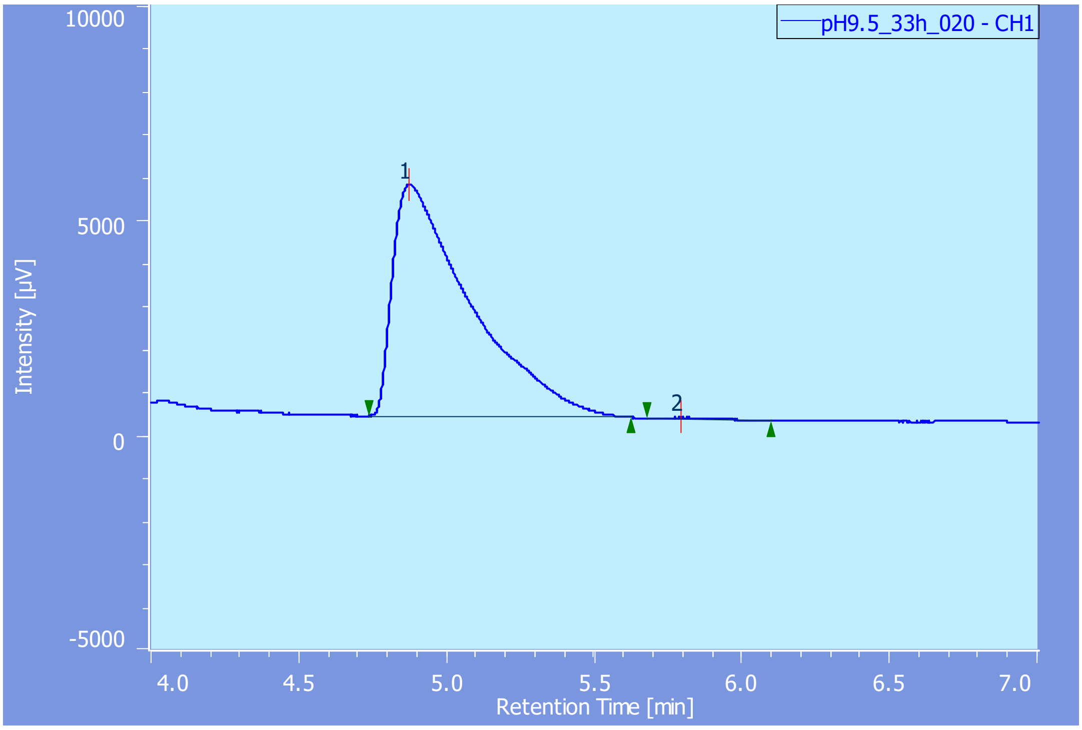


**Figure S6.10**. HPLC chart of **1** at pH 9.2 (33 h after heating)

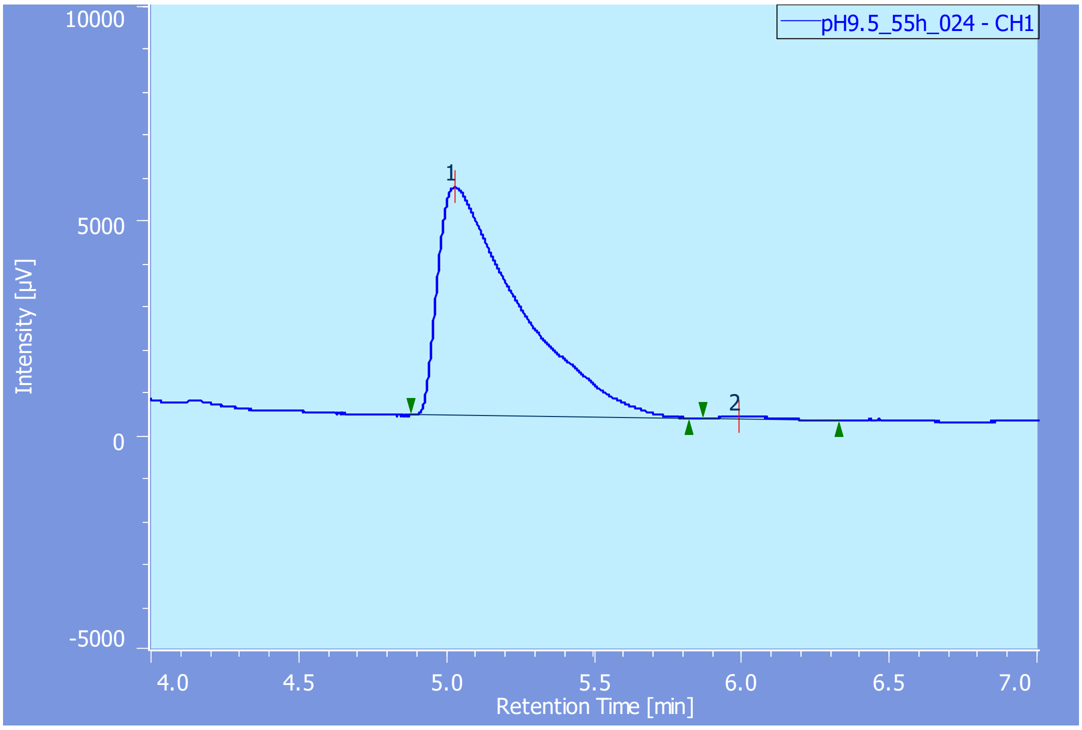


**Figure S6.11**. HPLC chart of **1** at pH 9.2 (55 h after heating)

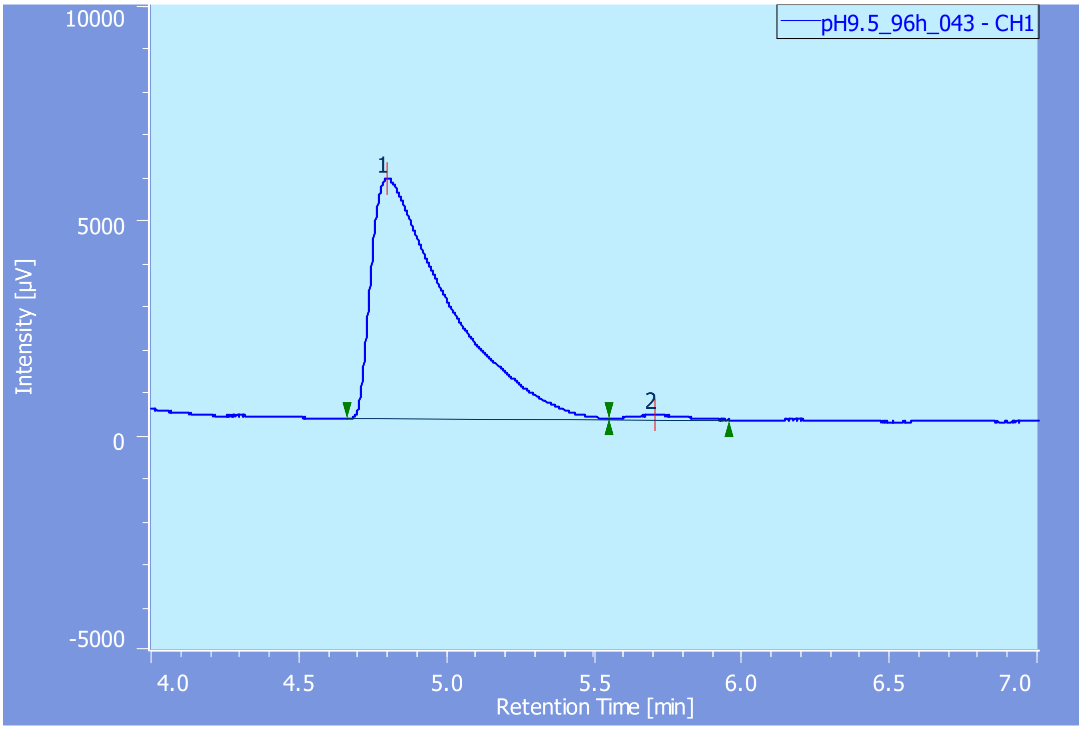


**Figure S6.12**. HPLC chart of **1** at pH 9.2 (96 h after heating)

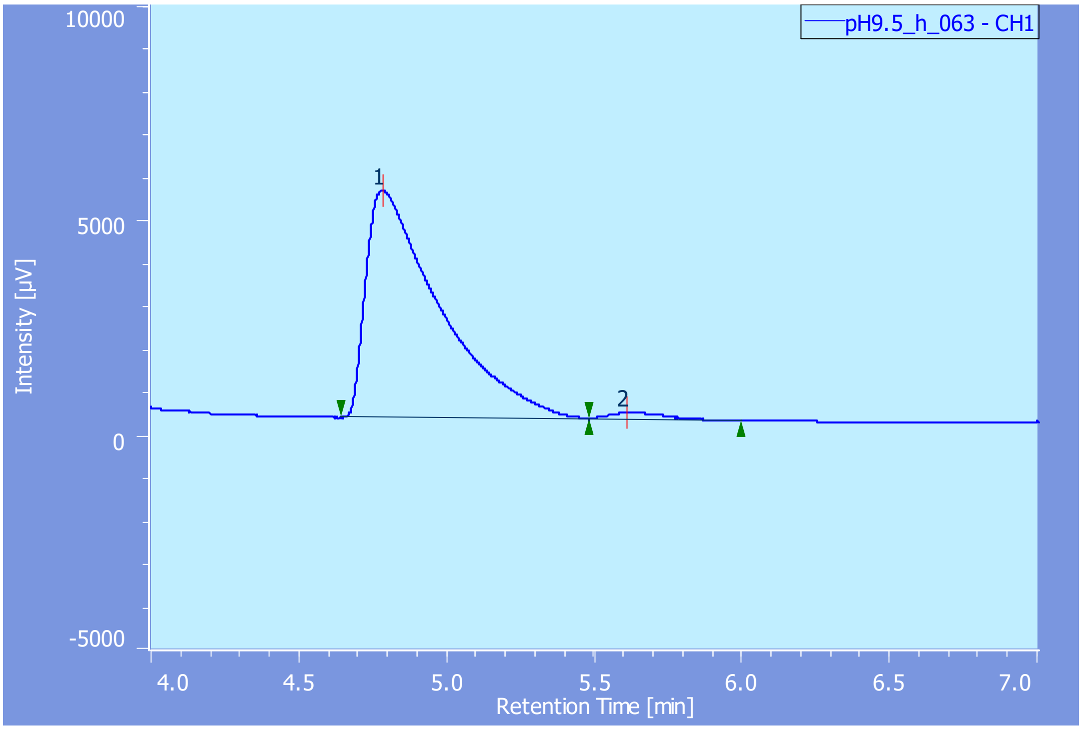


**Figure S6.13**. HPLC chart of **1** at pH 9.2 (151 h after heating)

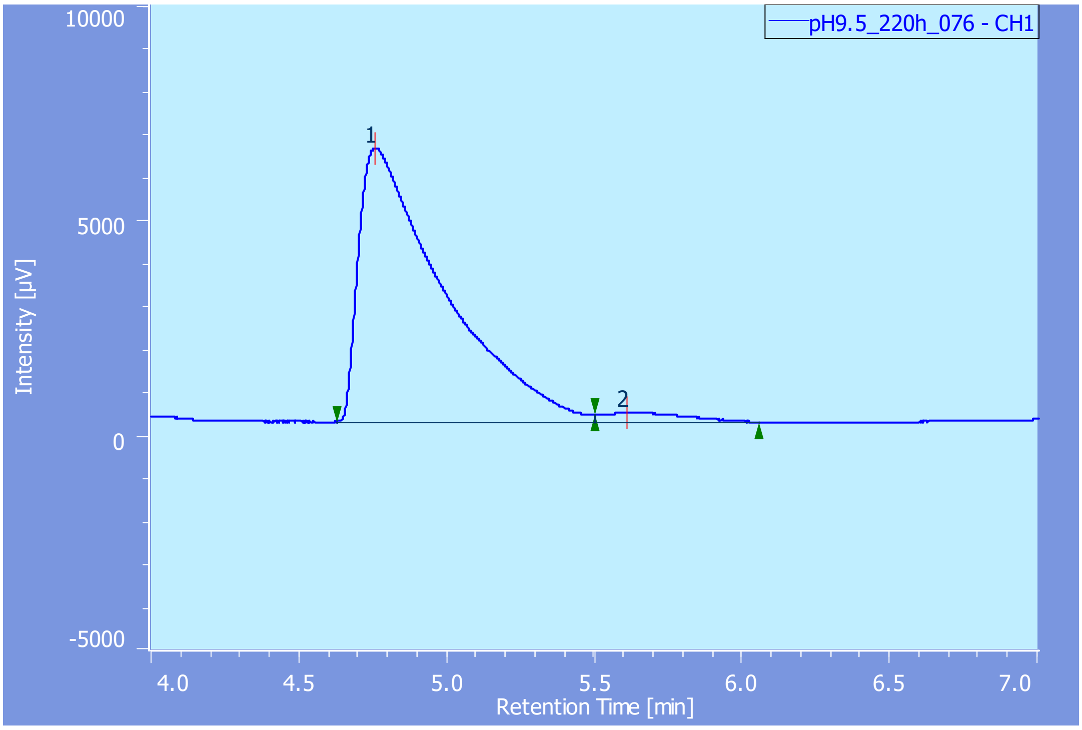


**Figure S6.14**. HPLC chart of **1** at pH 9.2 (220 h after heating)

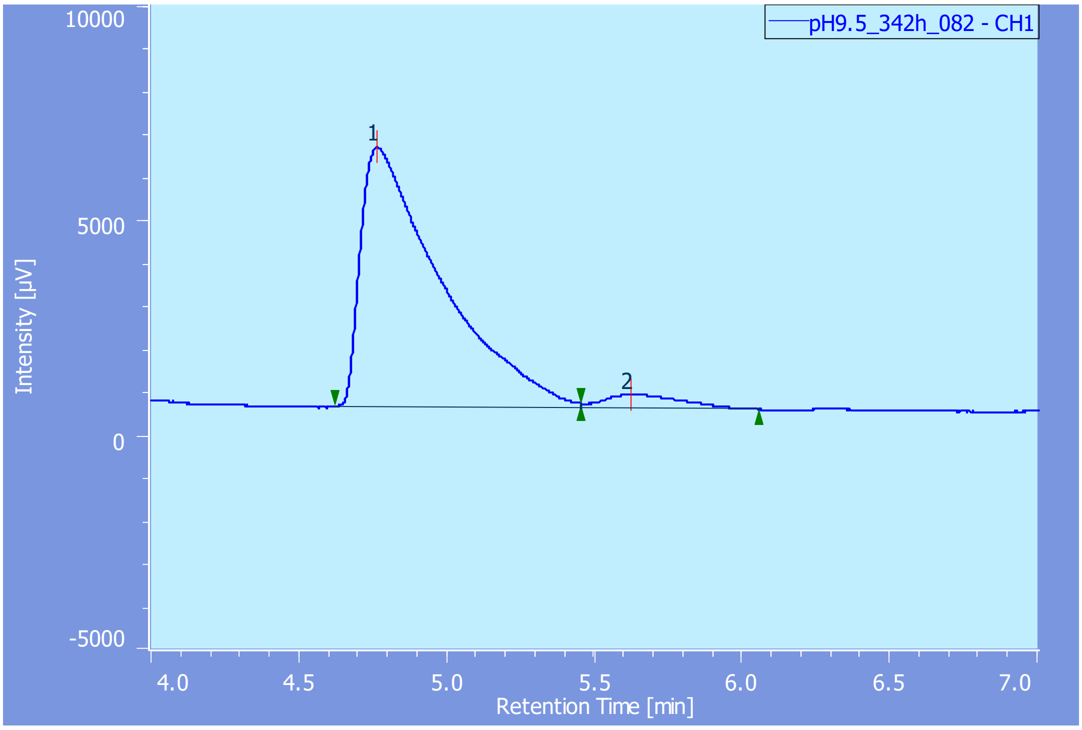


**Figure S6.15**. HPLC chart of **1** at pH 9.2 (342 h after heating)

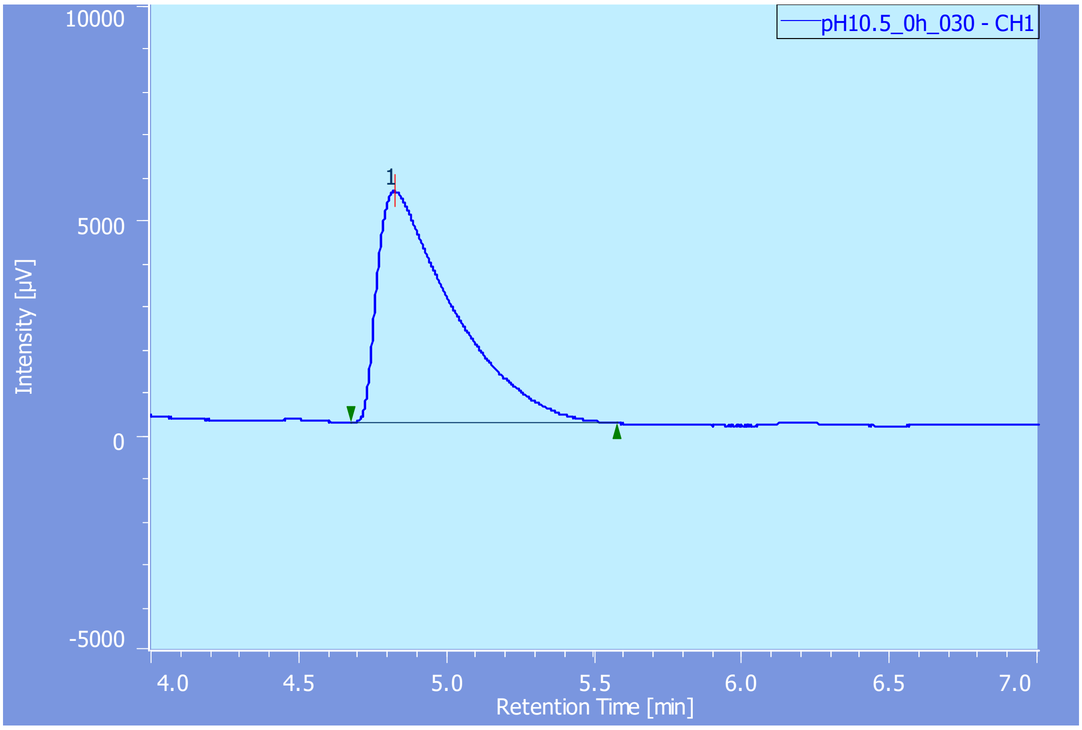


**Figure S6.16**. HPLC chart of **1** at pH 10.3 (0 h after heating)

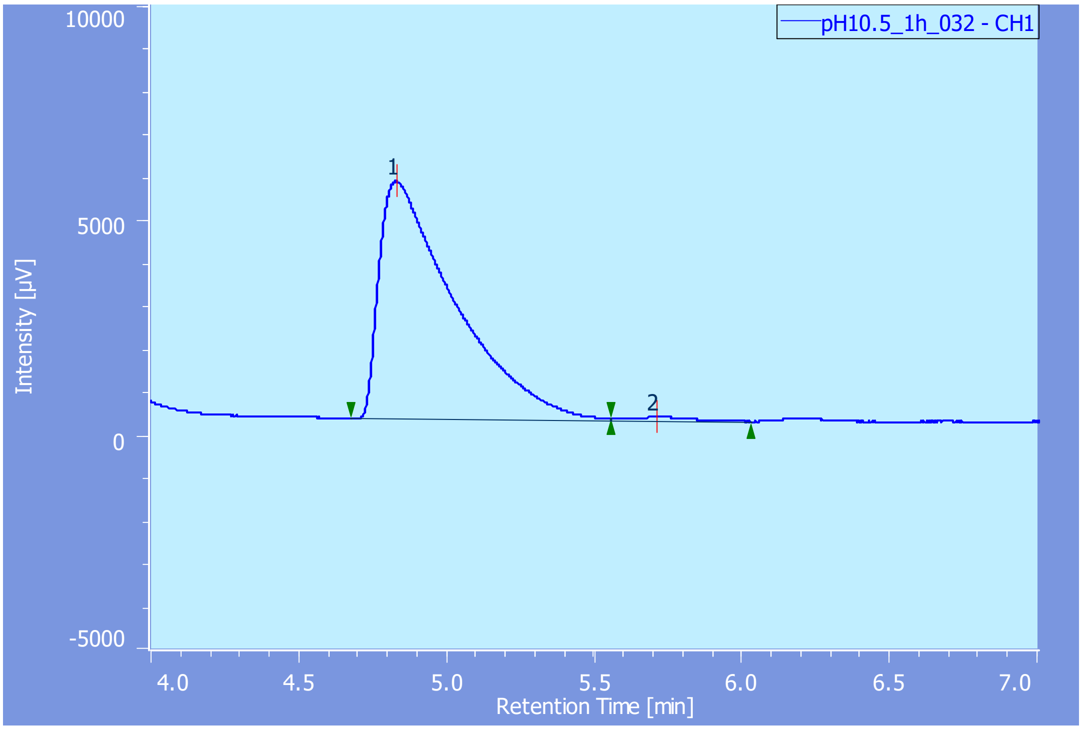


**Figure S6.17**. HPLC chart of **1** at pH 10.3 (1 h after heating)

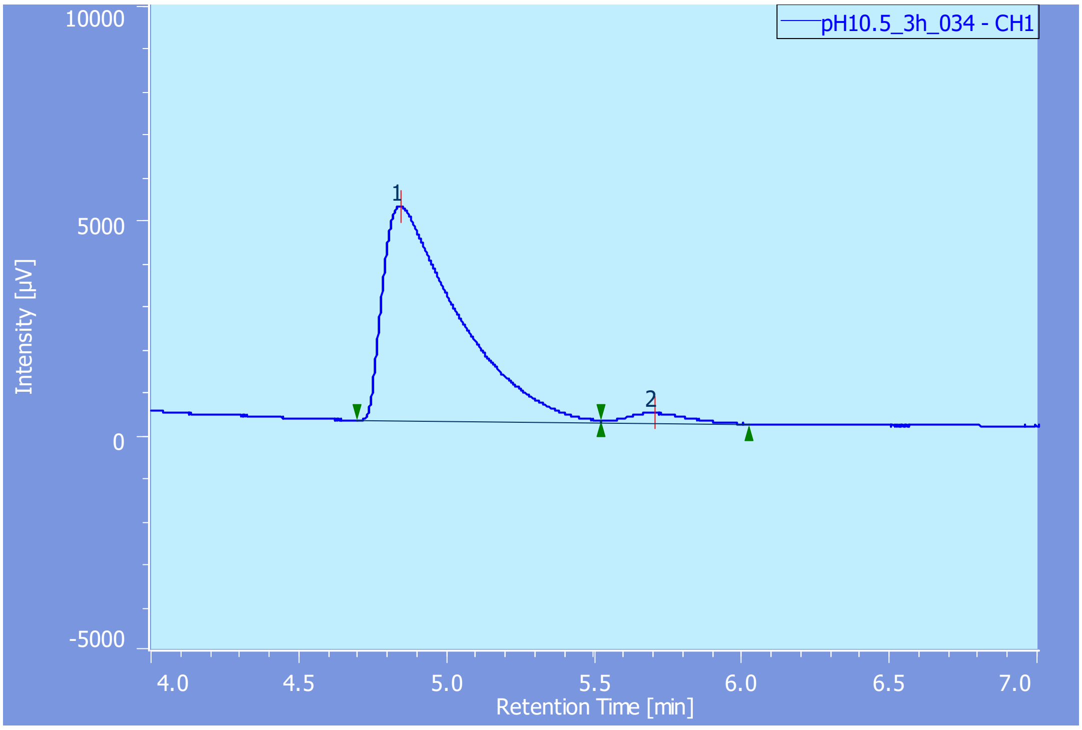


**Figure S6.18**. HPLC chart of **1** at pH 10.3 (3 h after heating)

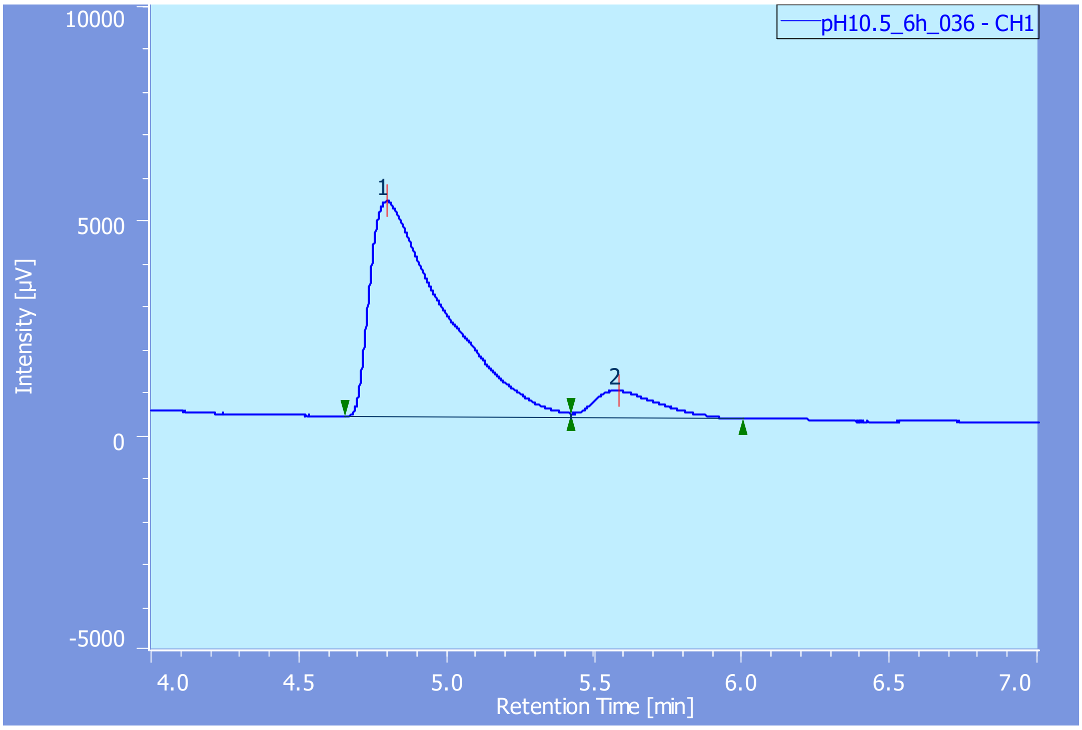


**Figure S6.19**. HPLC chart of **1** at pH 10.3 (6 h after heating)

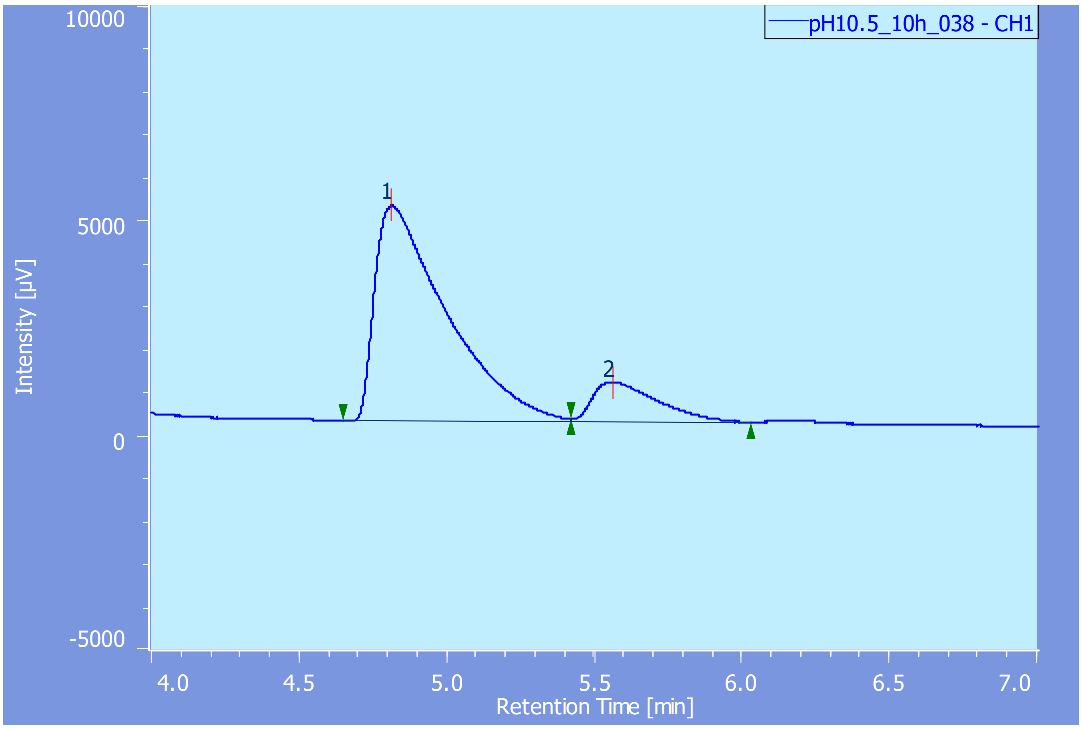


**Figure S6.20**. HPLC chart of **1** at pH 10.3 (10 h after heating)

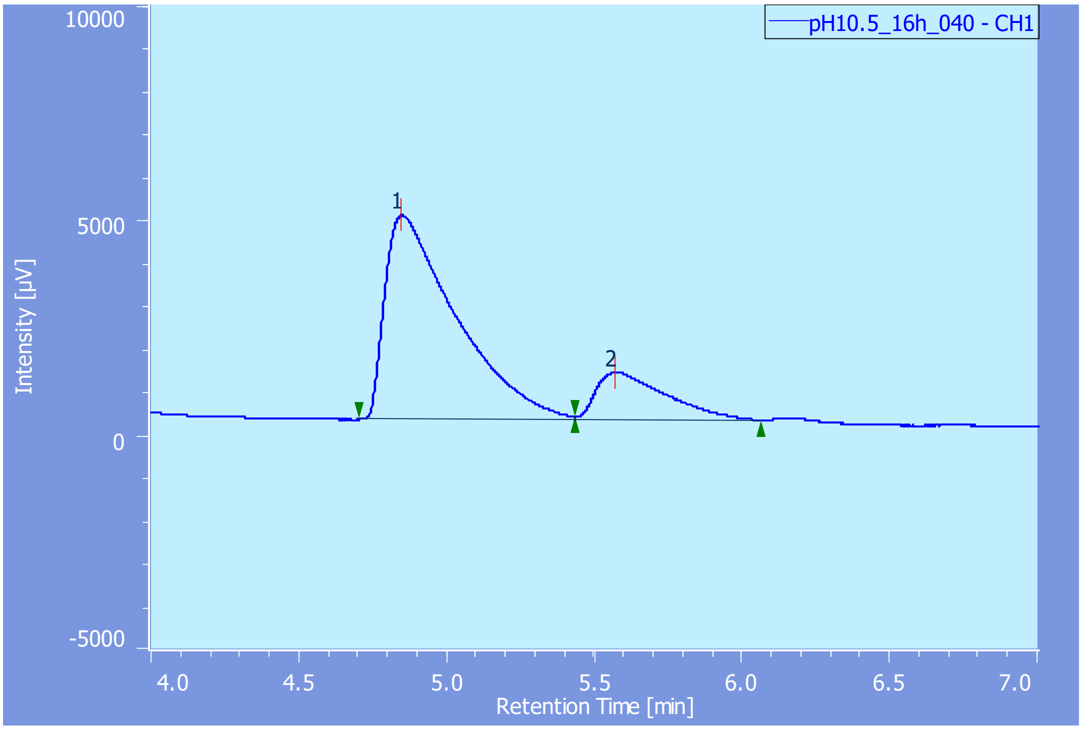


**Figure S6.21**. HPLC chart of **1** at pH 10.3 (16 h after heating)

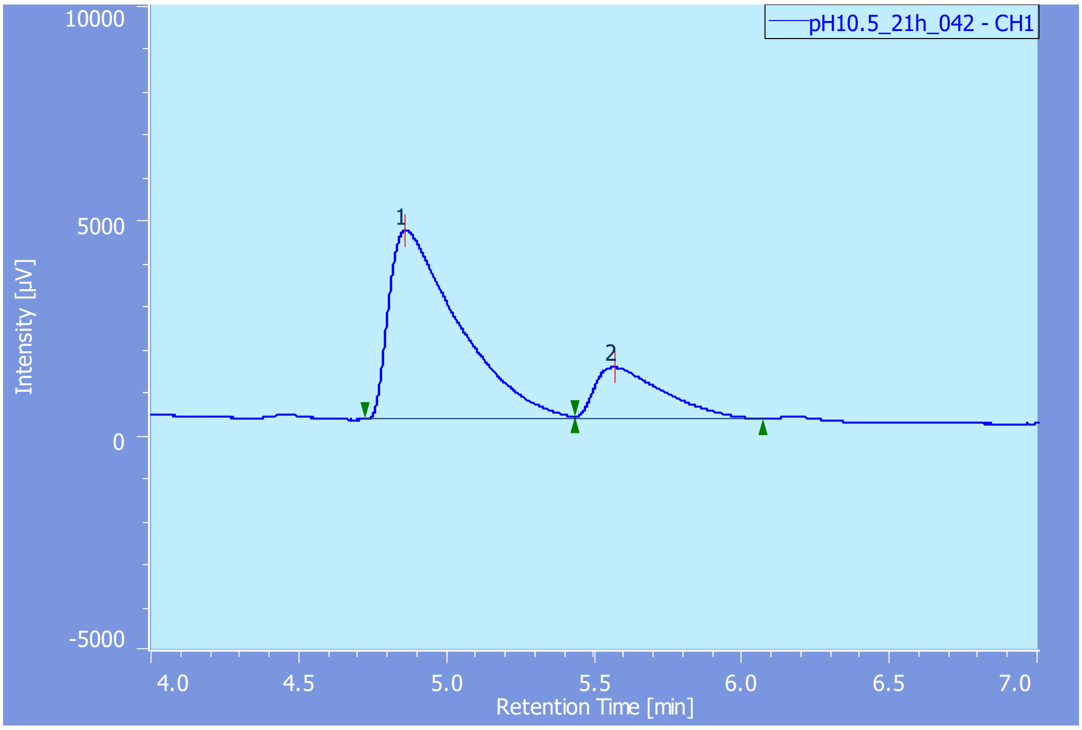


**Figure S6.22**. HPLC chart of **1** at pH 10.3 (21 h after heating)

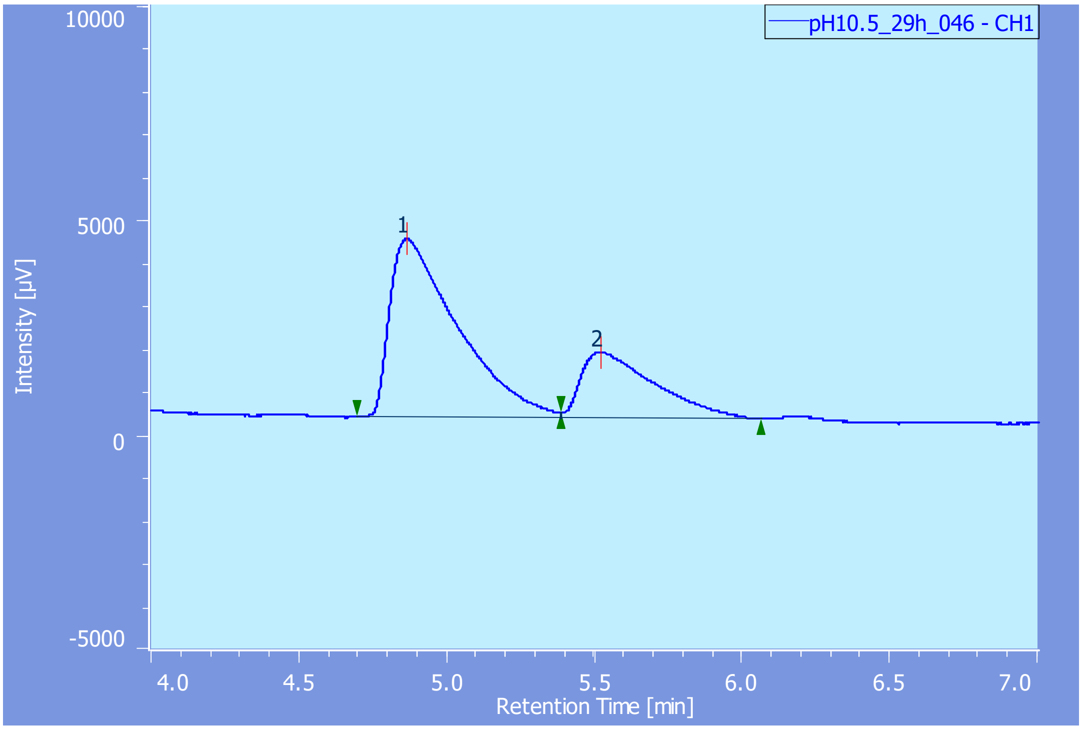


**Figure S6.23**. HPLC chart of **1** at pH 10.3 (29 h after heating)

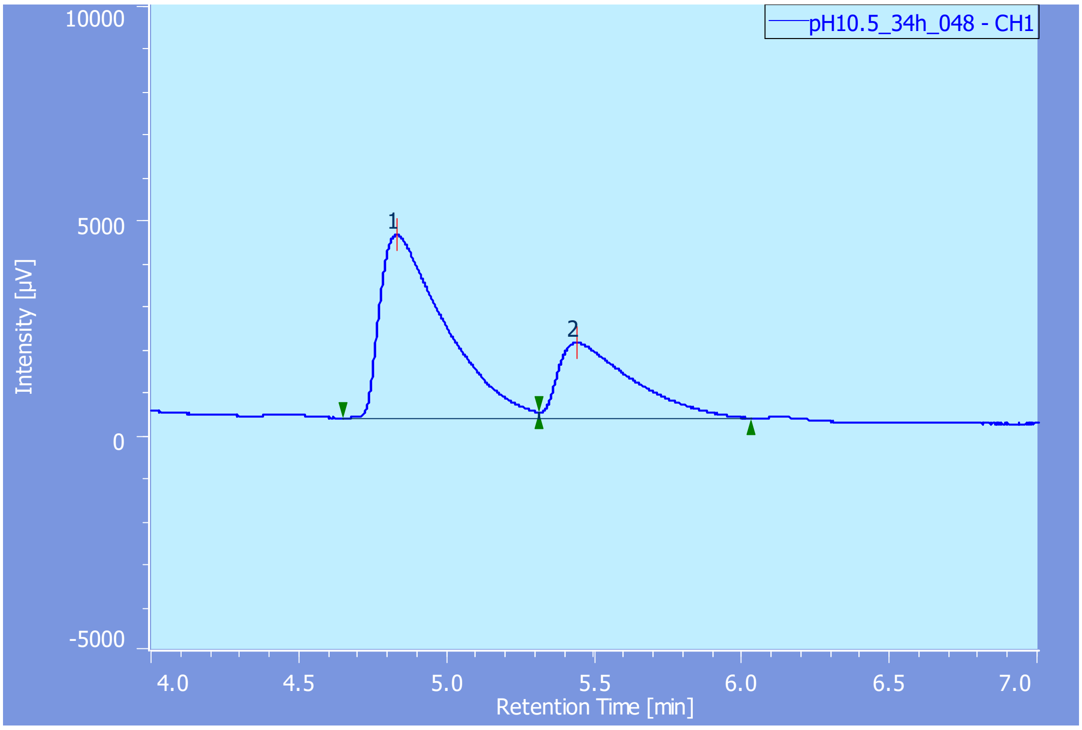


**Figure S6.24**. HPLC chart of **1** at pH 10.3 (34 h after heating)

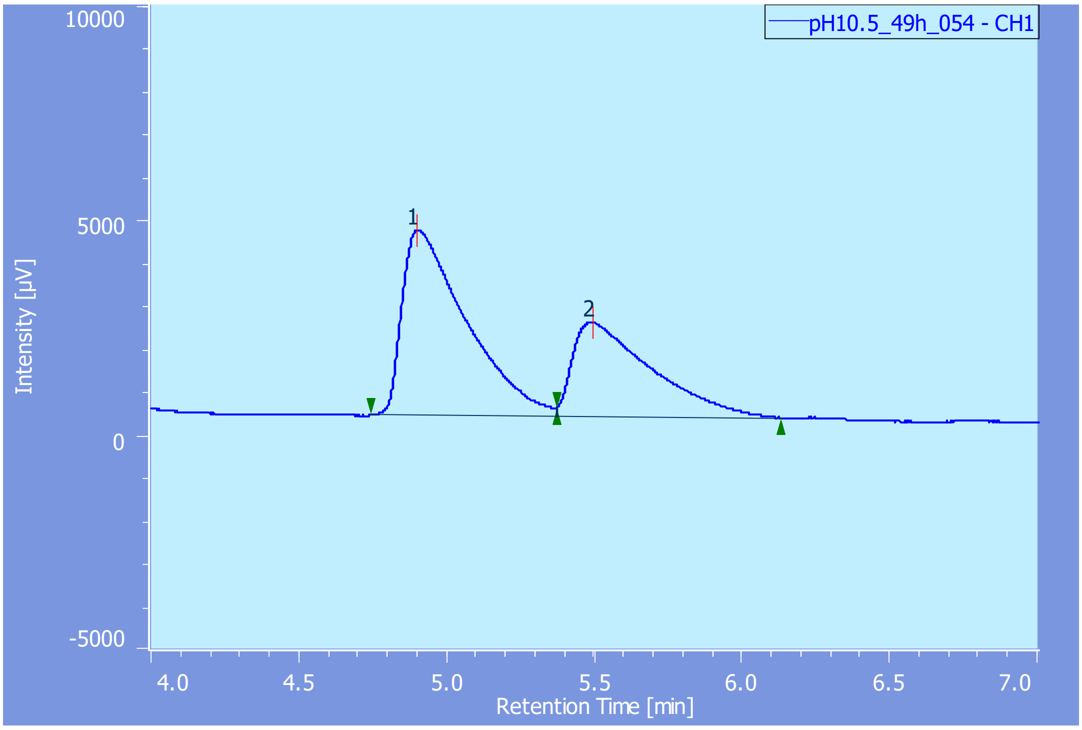


**Figure S6.25**. HPLC chart of **1** at pH 10.3 (49 h after heating)

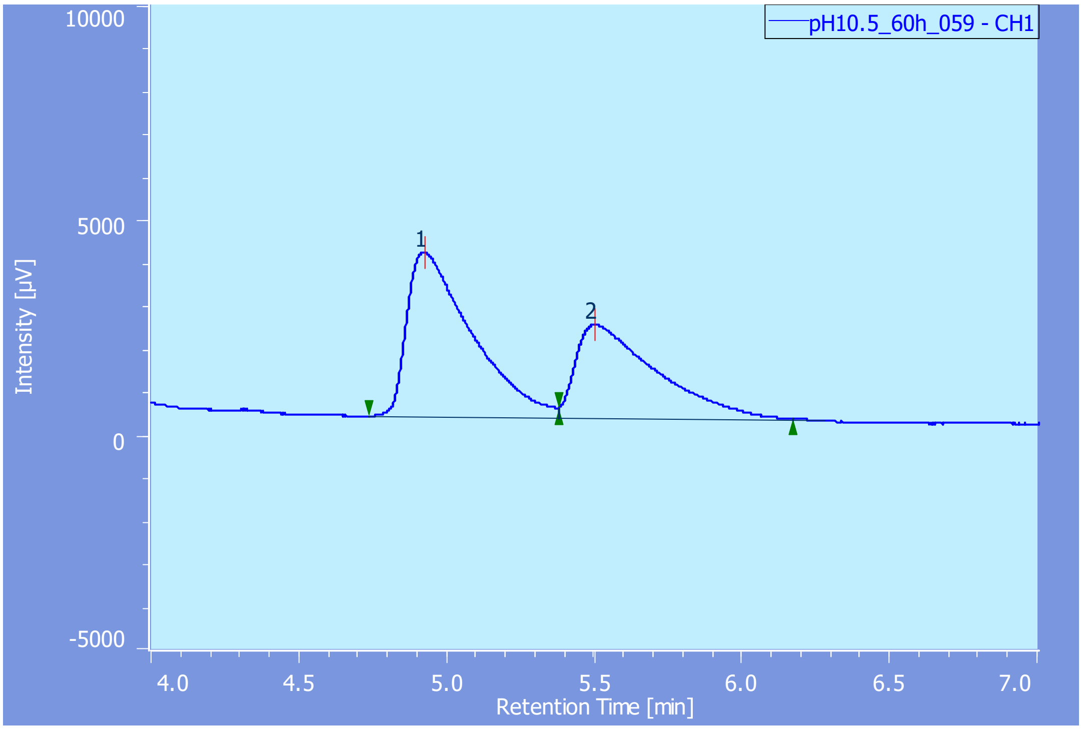


**Figure S6.26**. HPLC chart of **1** at pH 10.3 (60 h after heating)

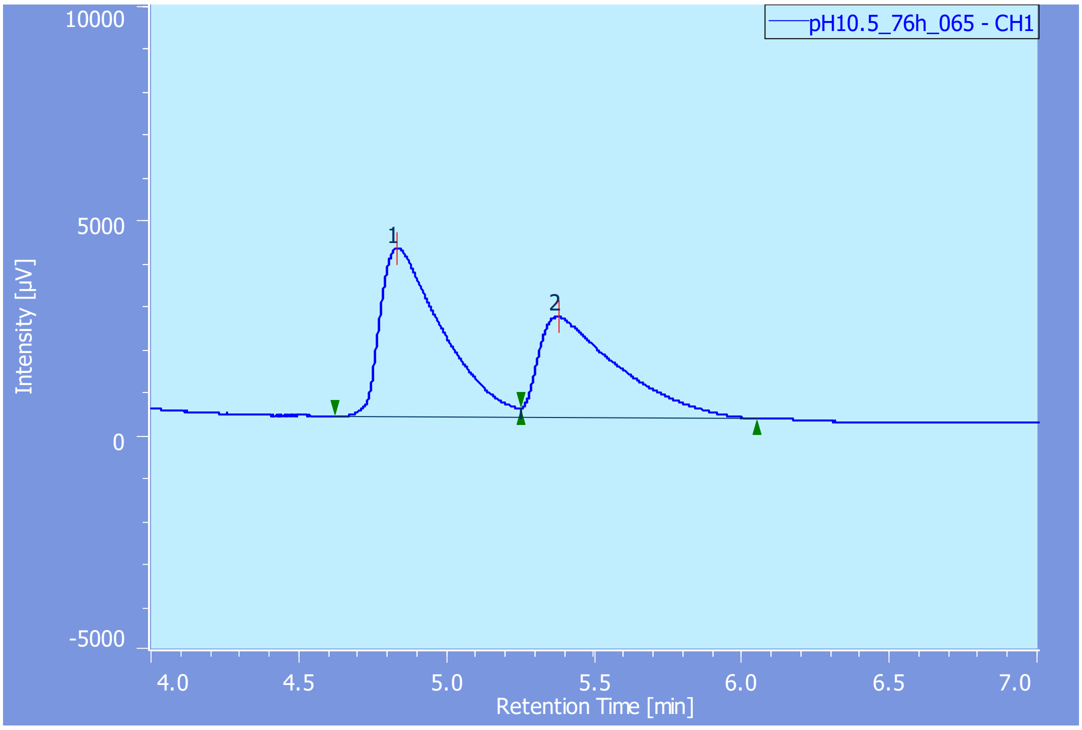


**Figure S6.27**. HPLC chart of **1** at pH 10.3 (76 h after heating)

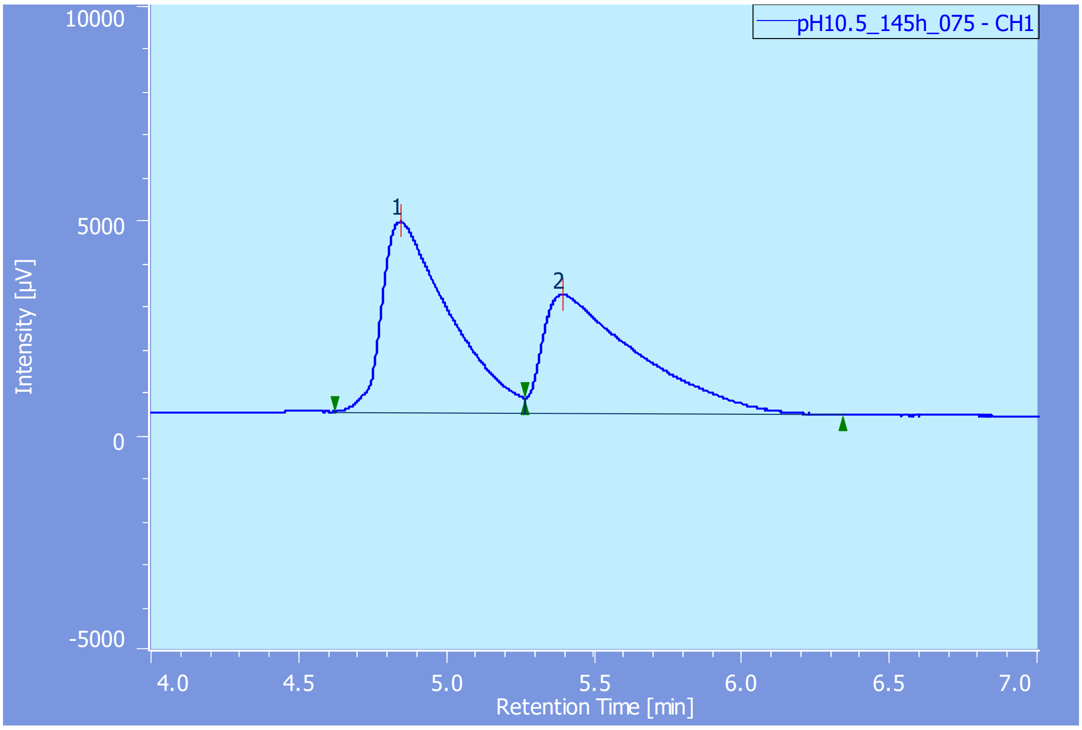


**Figure S6.28**. HPLC chart of **1** at pH 10.3 (145 h after heating)

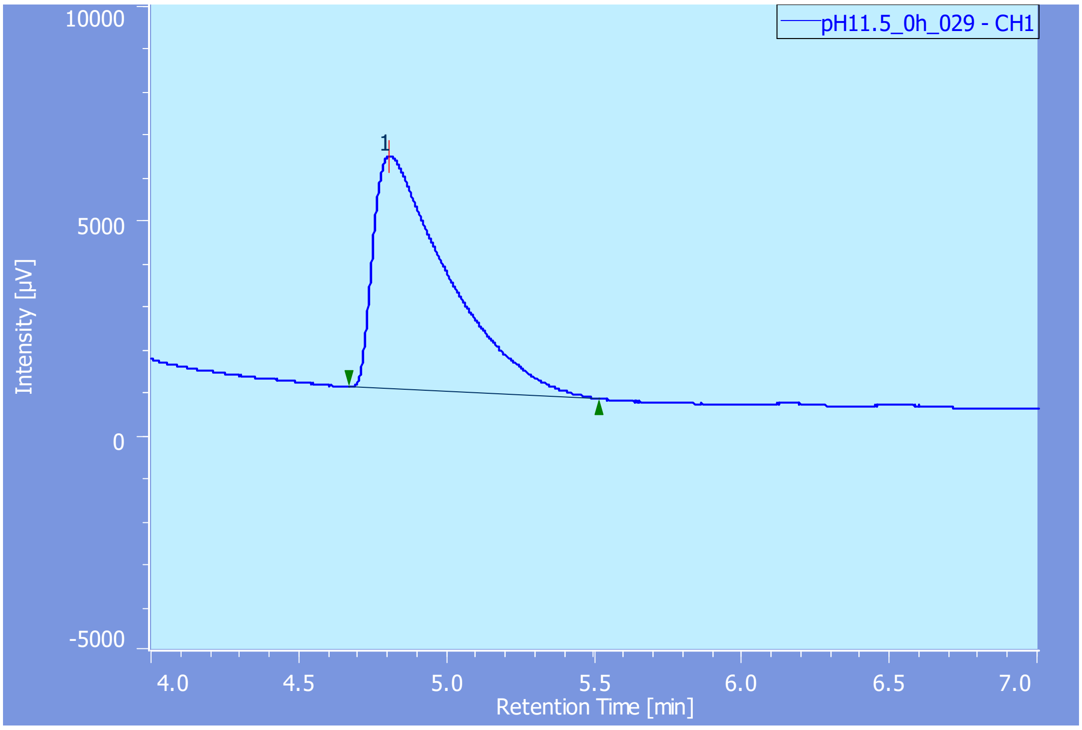


**Figure S6.29**. HPLC chart of **1** at pH 11.7 (0 h after heating)

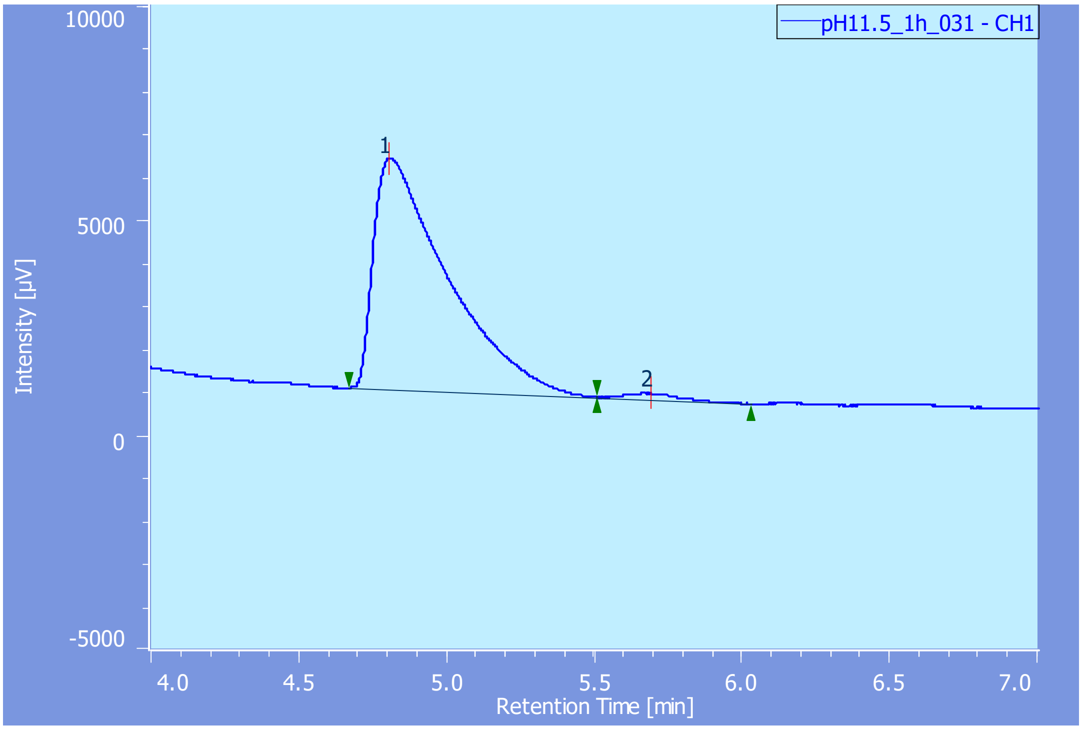


**Figure S6.30**. HPLC chart of **1** at pH 11.7 (1 h after heating)

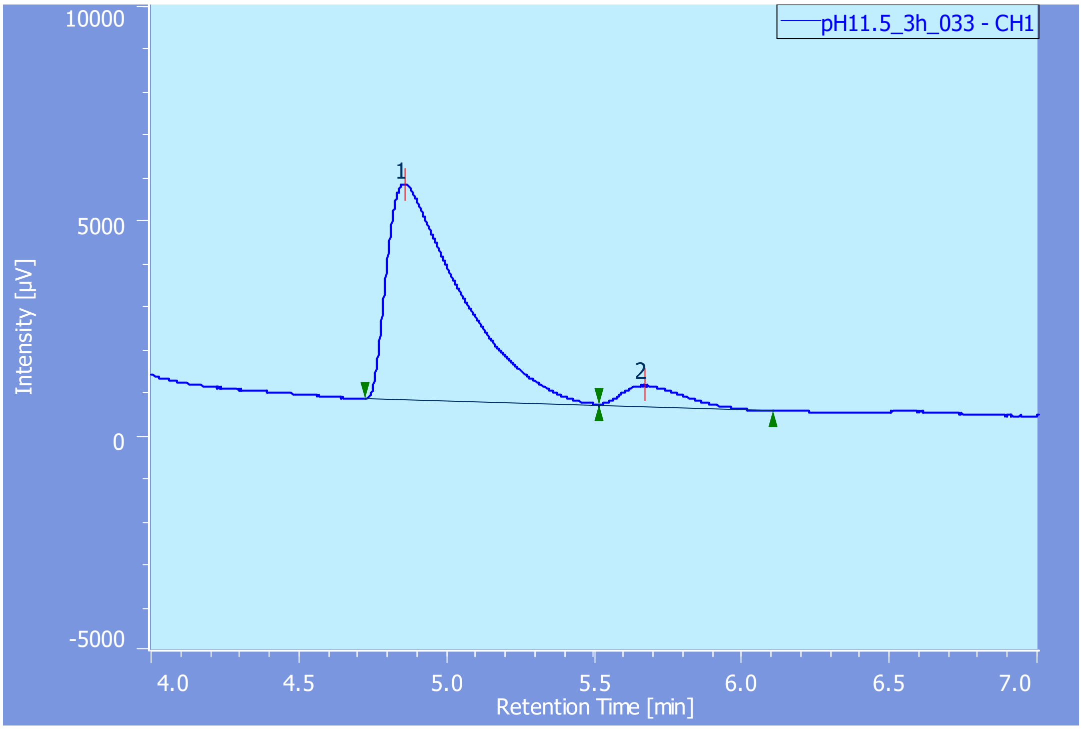


**Figure S6.31**. HPLC chart of **1** at pH 11.7 (3 h after heating)

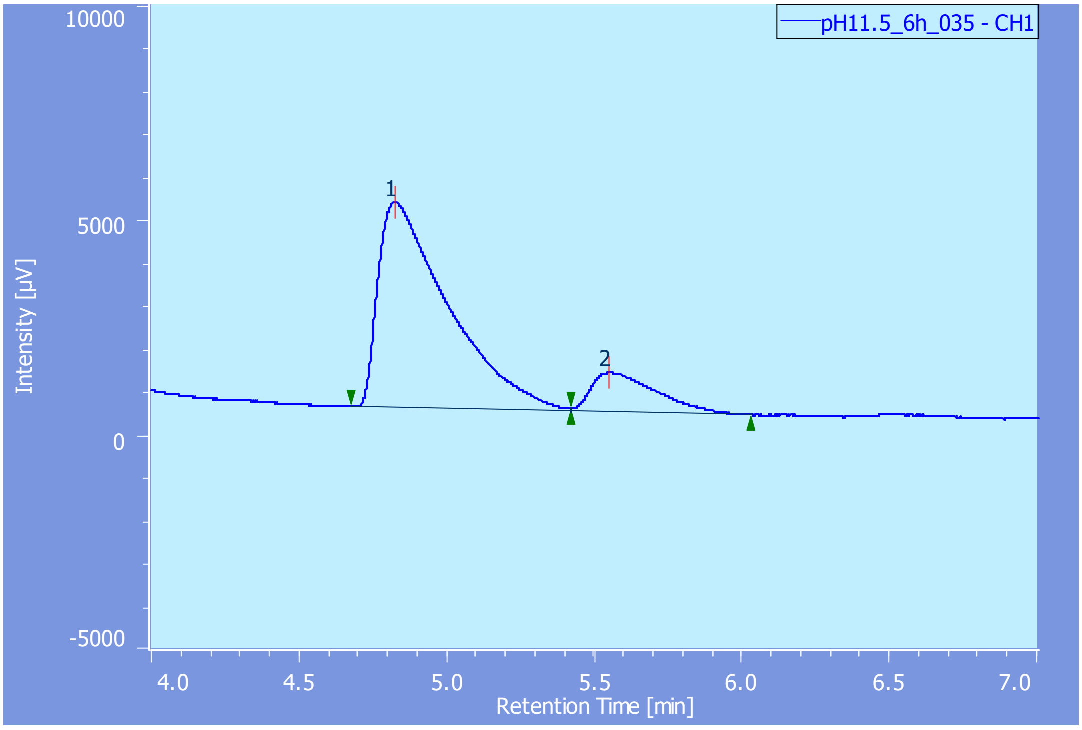


**Figure S6.32**. HPLC chart of **1** at pH 11.7 (6 h after heating)

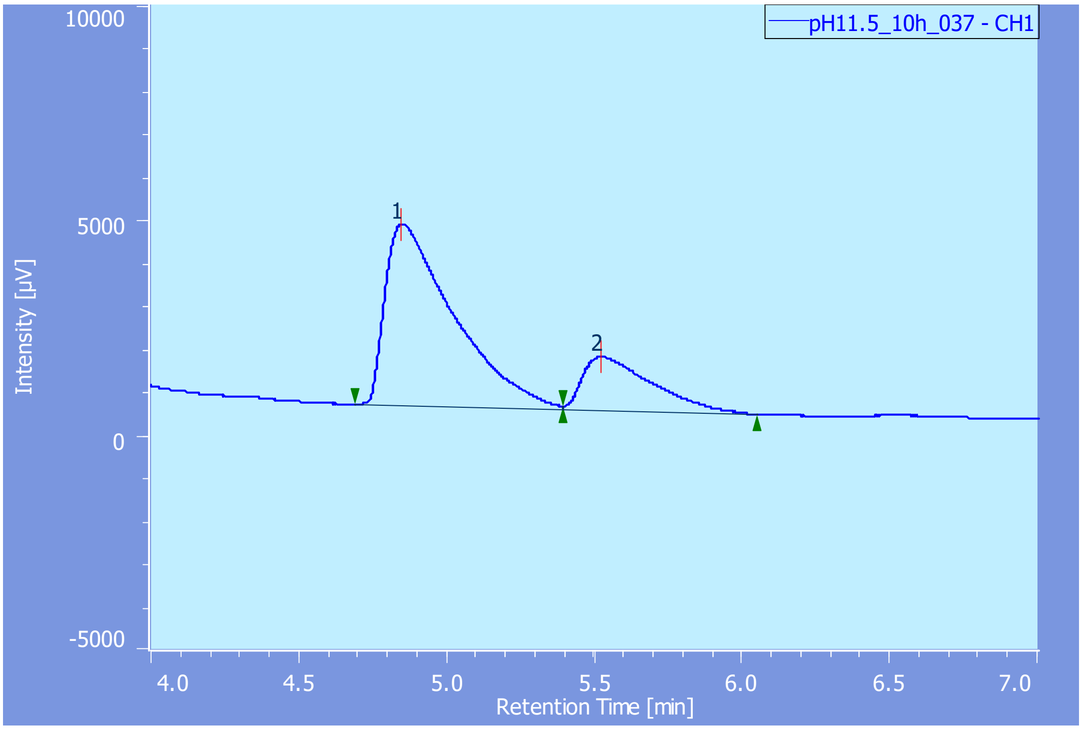


**Figure S6.33**. HPLC chart of **1** at pH 11.7 (10 h after heating)

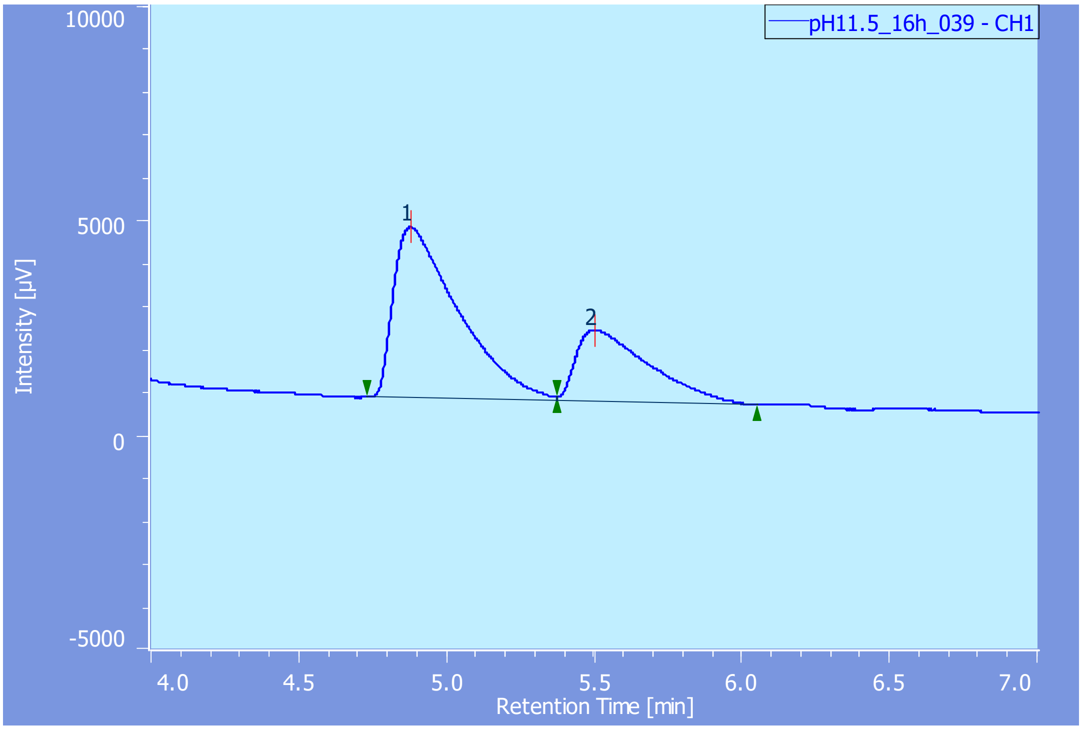


**Figure S6.34**. HPLC chart of **1** at pH 11.7 (16 h after heating)

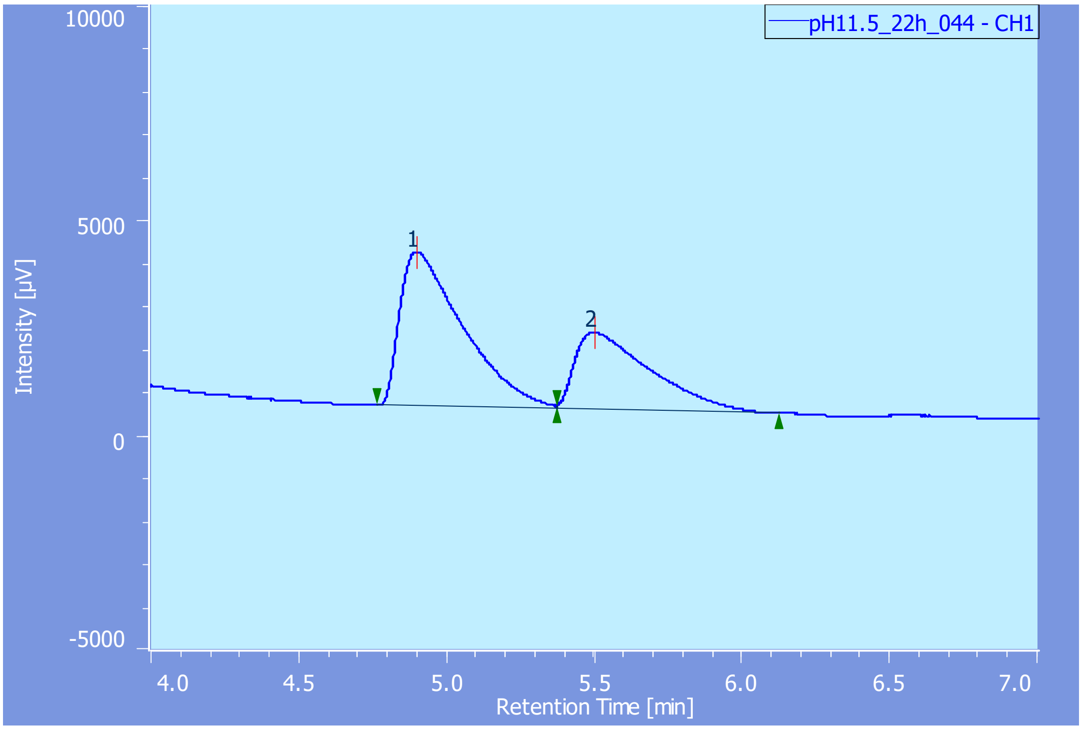


**Figure S6.35**. HPLC chart of **1** at pH 11.7 (22 h after heating)

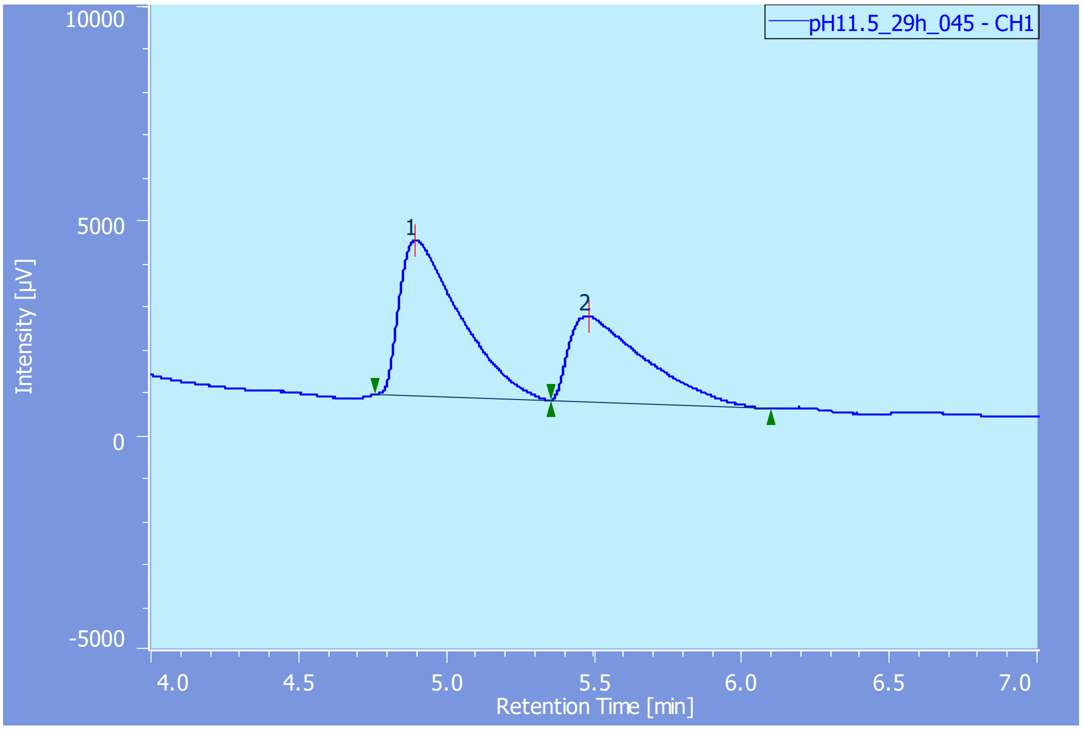


**Figure S6.36**. HPLC chart of **1** at pH 11.7 (29 h after heating)

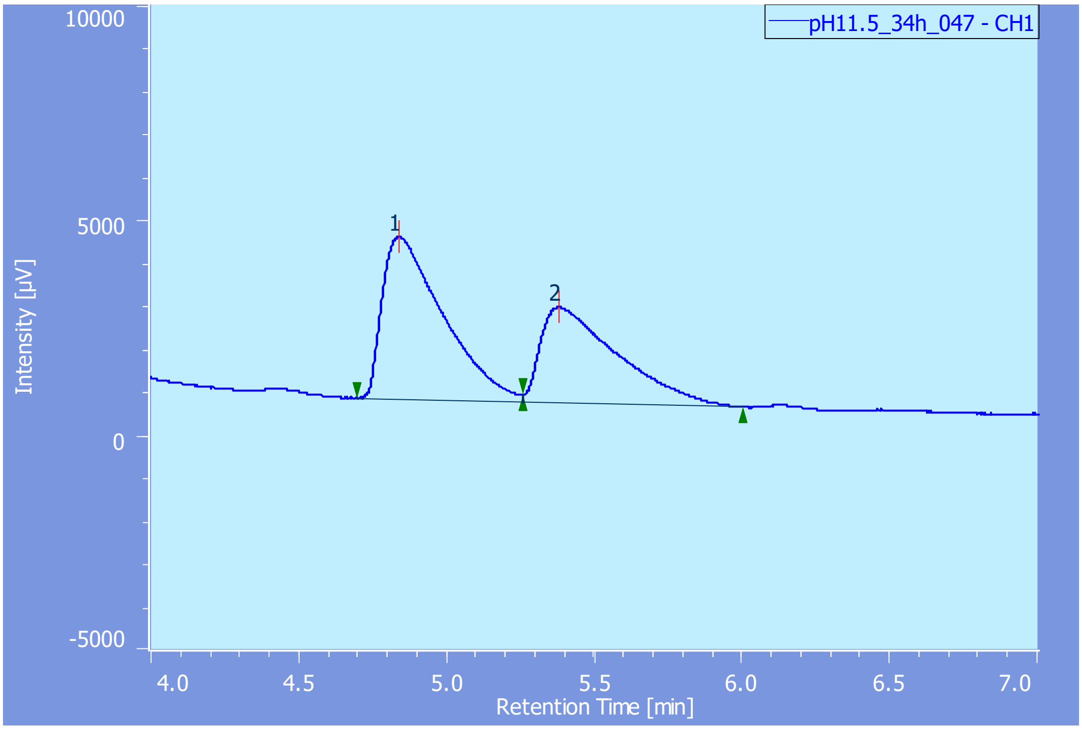


**Figure S6.37**. HPLC chart of **1** at pH 11.7 (34 h after heating)

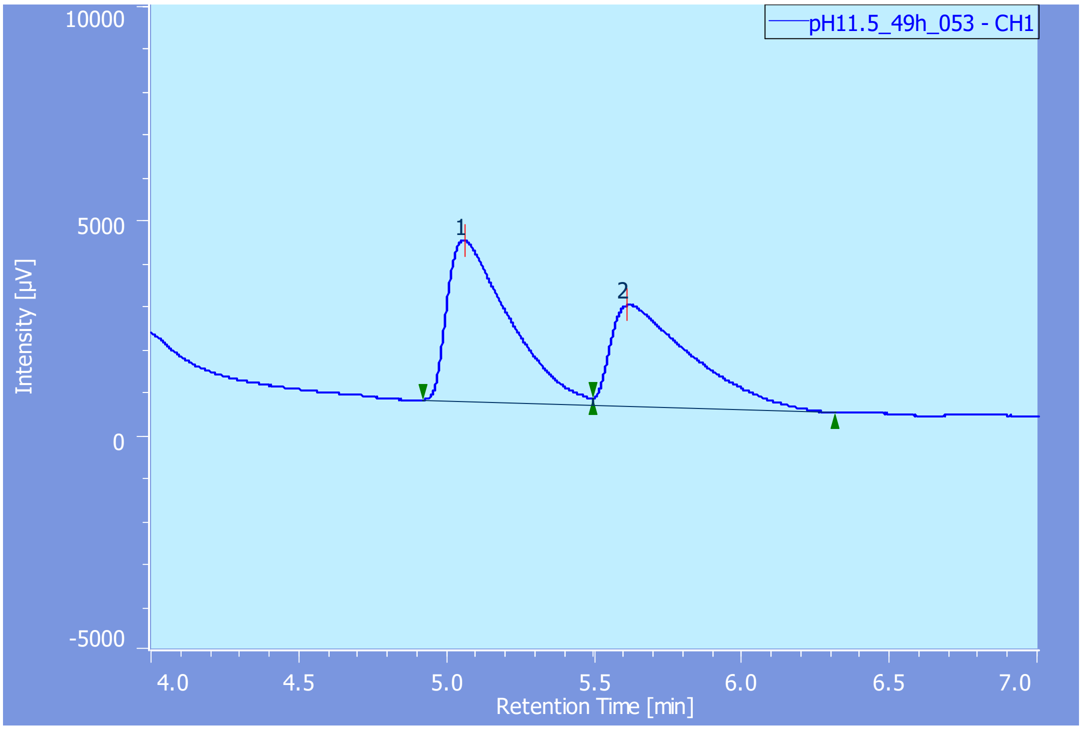


**Figure S6.38**. HPLC chart of **1** at pH 11.7 (49 h after heating)

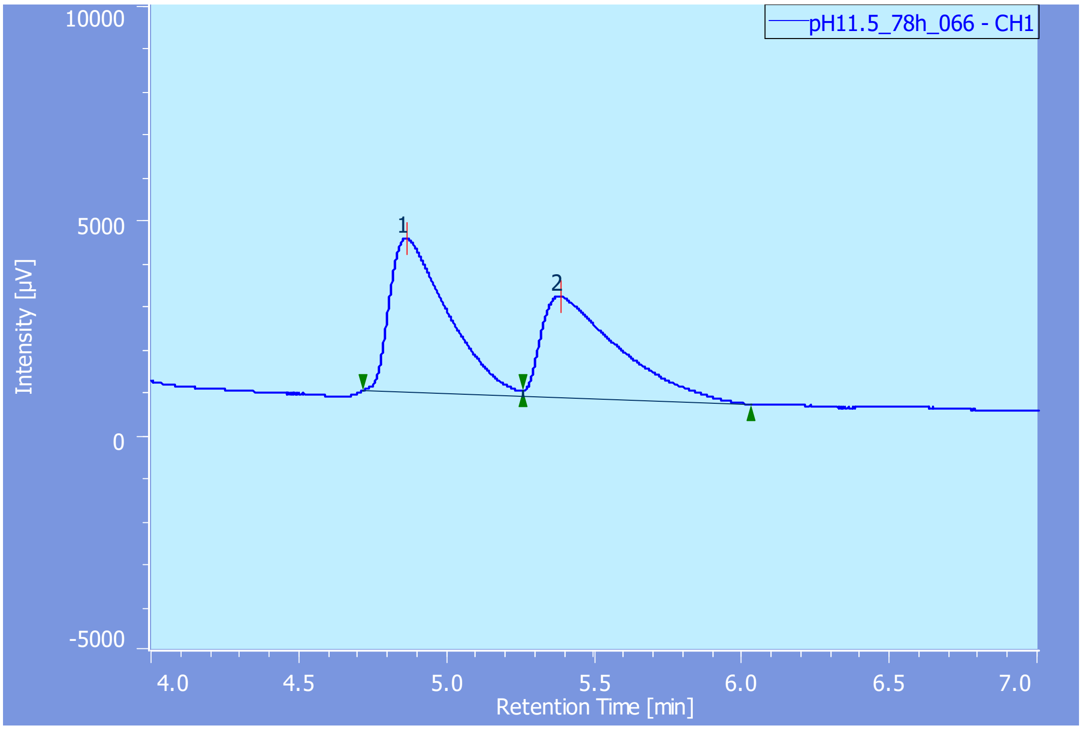


**Figure S6.39**. HPLC chart of **1** at pH 11.7 (78 h after heating)

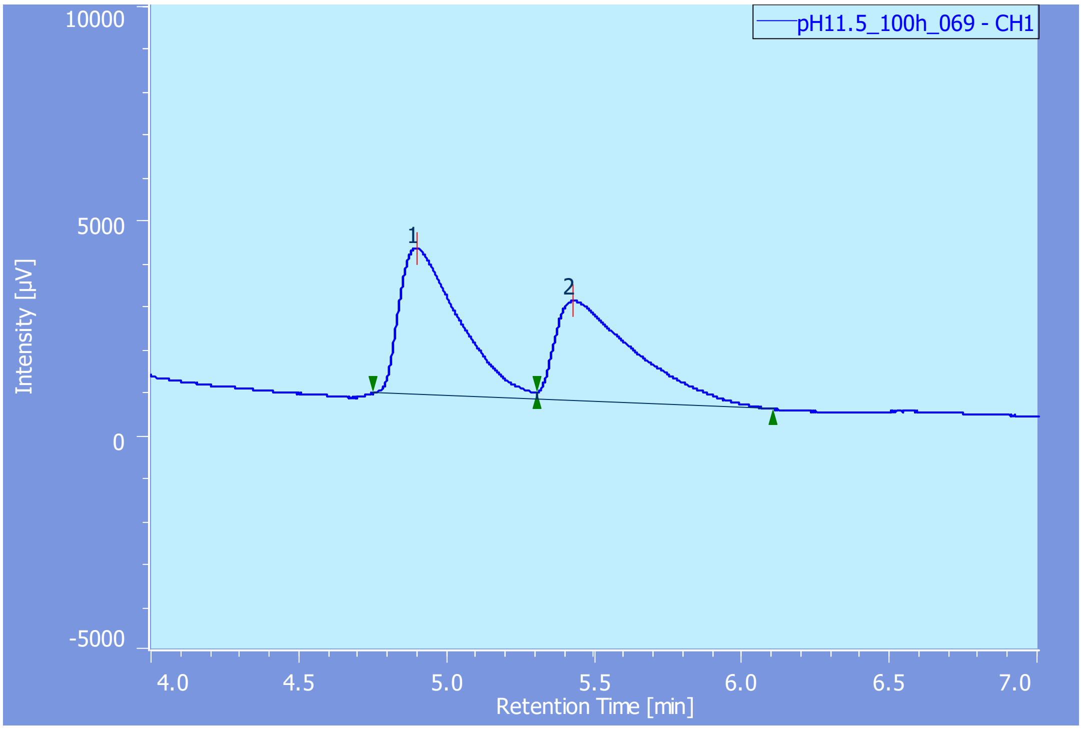


**Figure S6.40**. HPLC chart of **1** at pH 11.7 (100 h after heating)

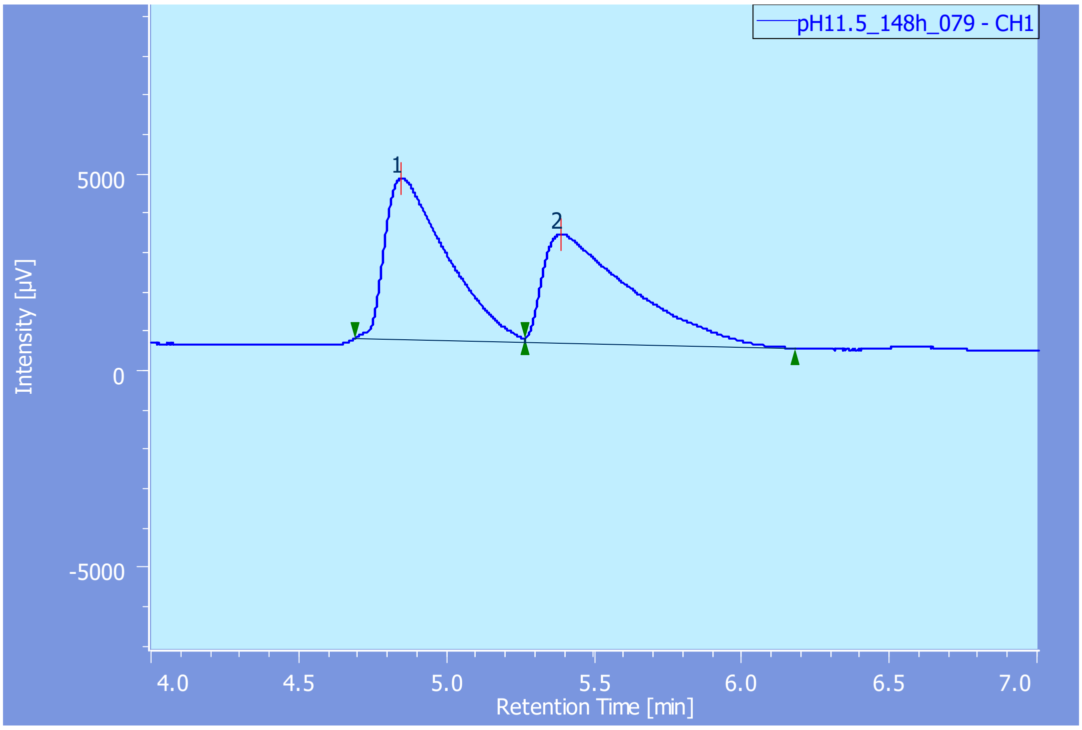


**Figure S6.41**. HPLC chart of **1** at pH 11.7 (148 h after heating)

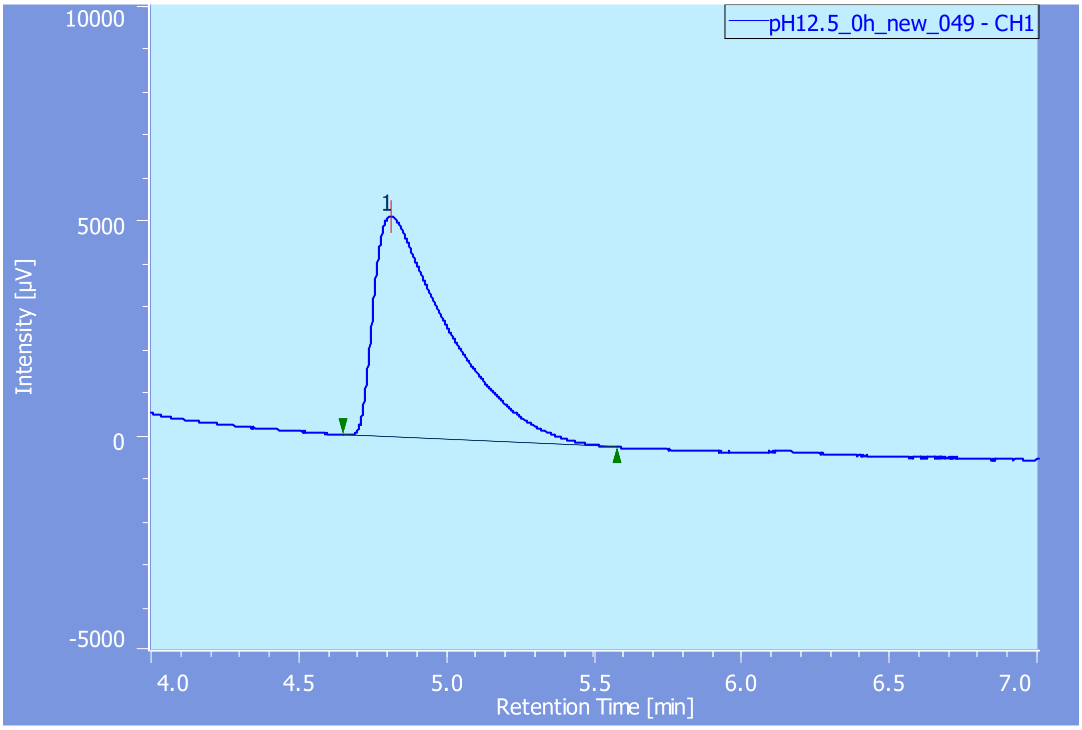


**Figure S6.42**. HPLC chart of **1** at pH 12.7 (0 h after heating)

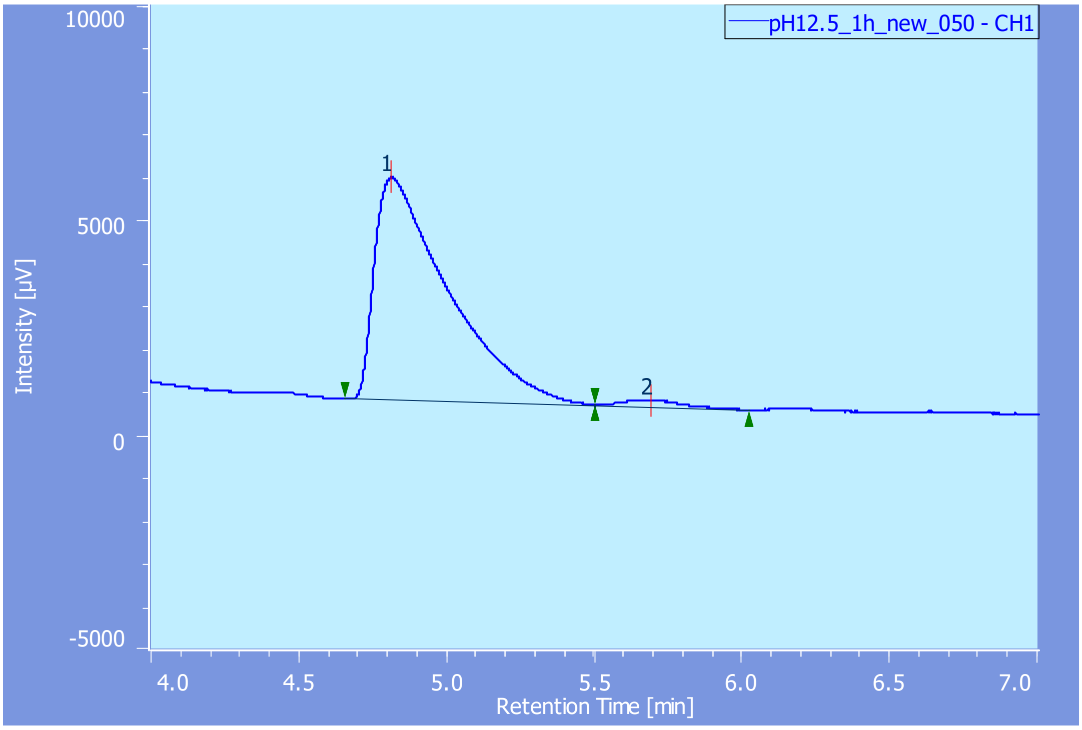


**Figure S6.43**. HPLC chart of **1** at pH 12.7 (1 h after heating)

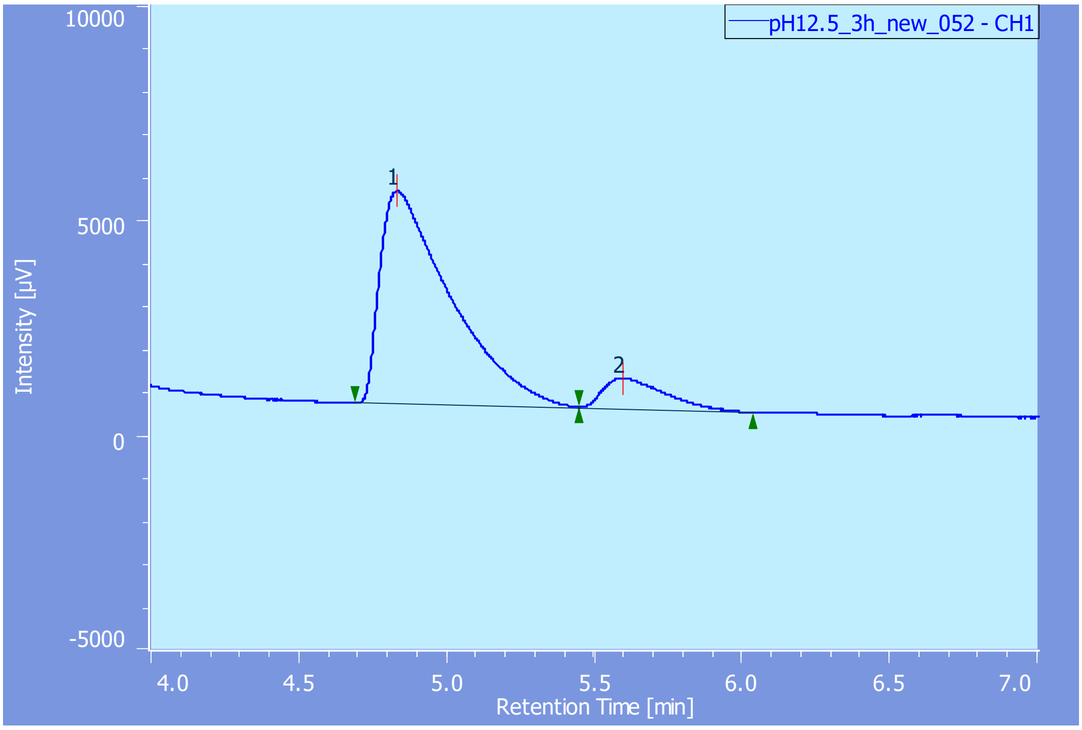


**Figure S6.44**. HPLC chart of **1** at pH 12.7 (3 h after heating)

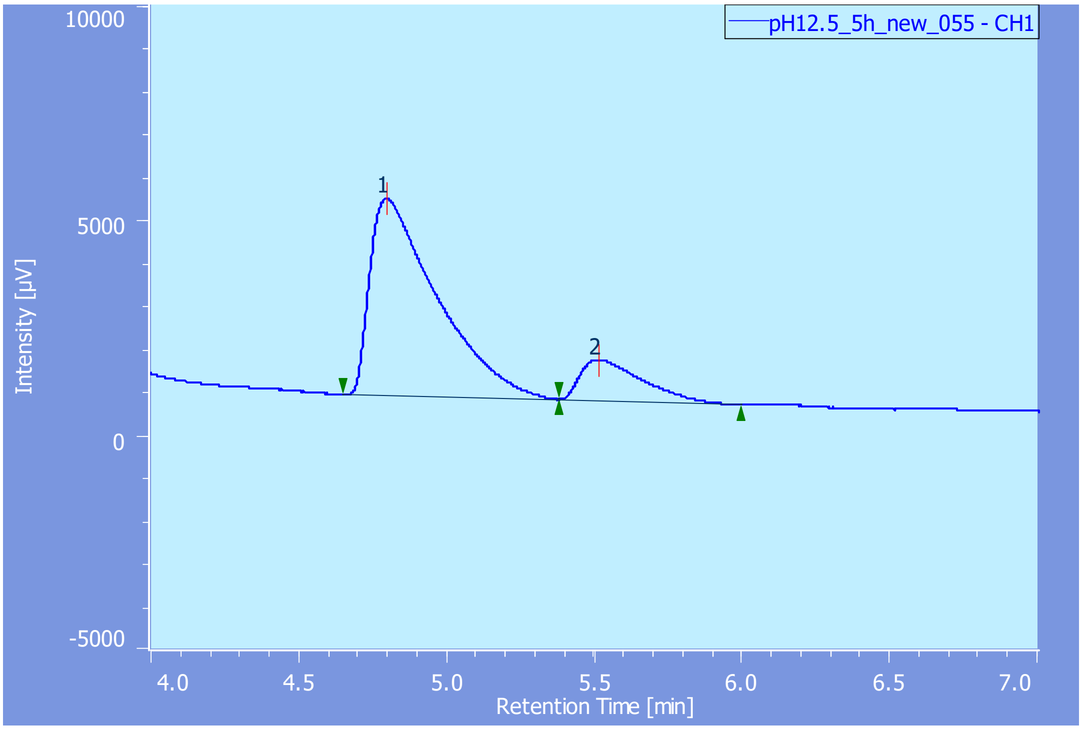


**Figure S6.45**. HPLC chart of **1** at pH 12.7 (5 h after heating)

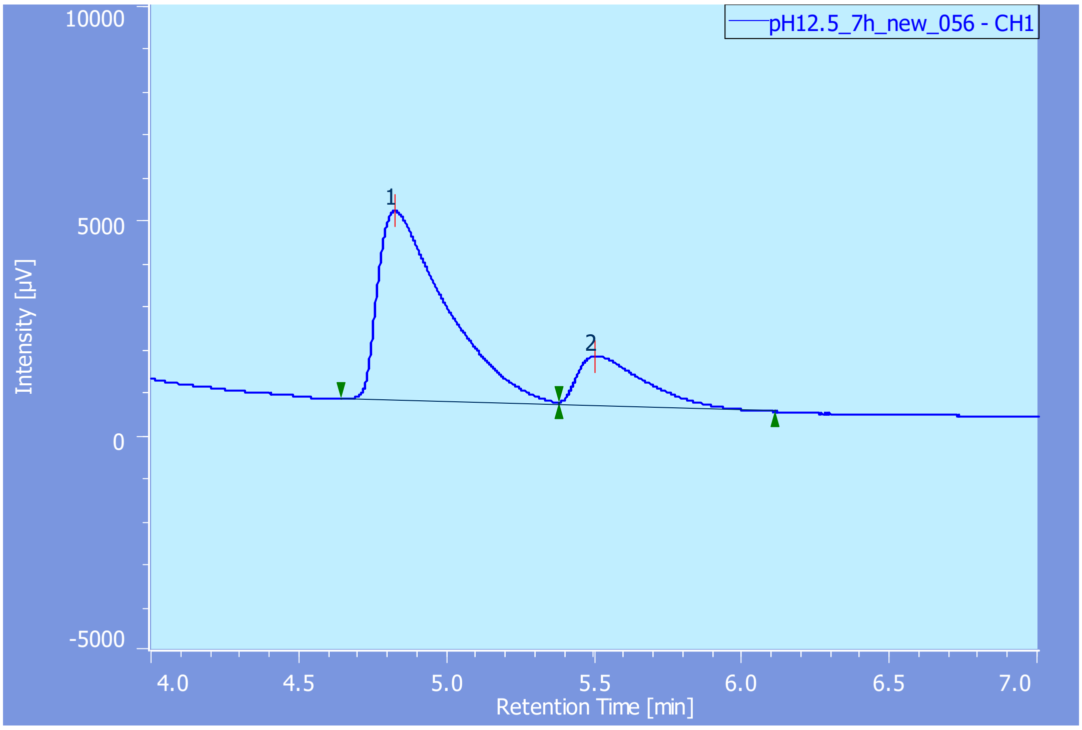


**Figure S6.46**. HPLC chart of **1** at pH 12.7 (7 h after heating)

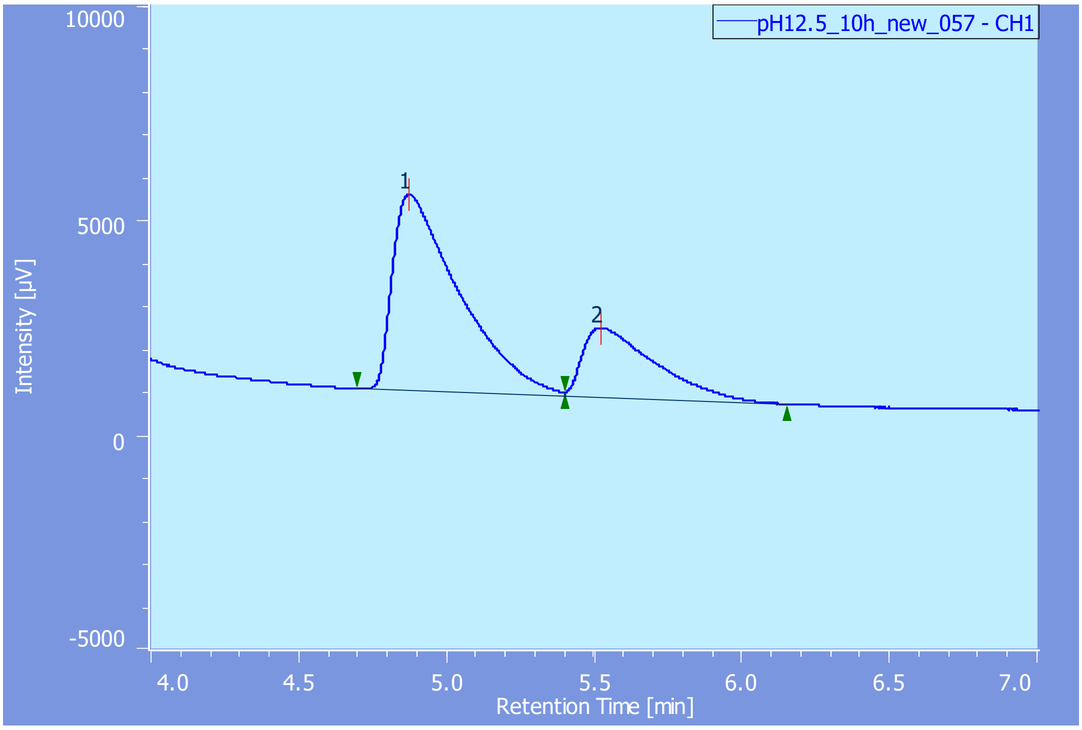


**Figure S6.47**. HPLC chart of **1** at pH 12.7 (10 h after heating)

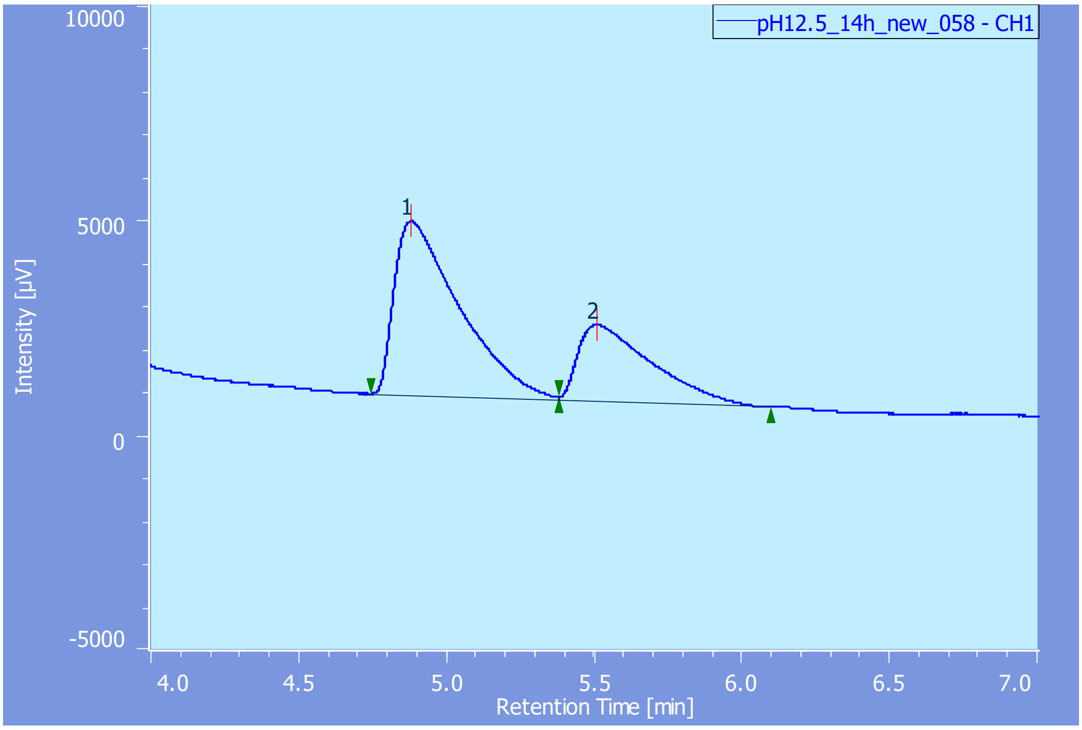


**Figure S6.48**. HPLC chart of **1** at pH 12.7 (14 h after heating)

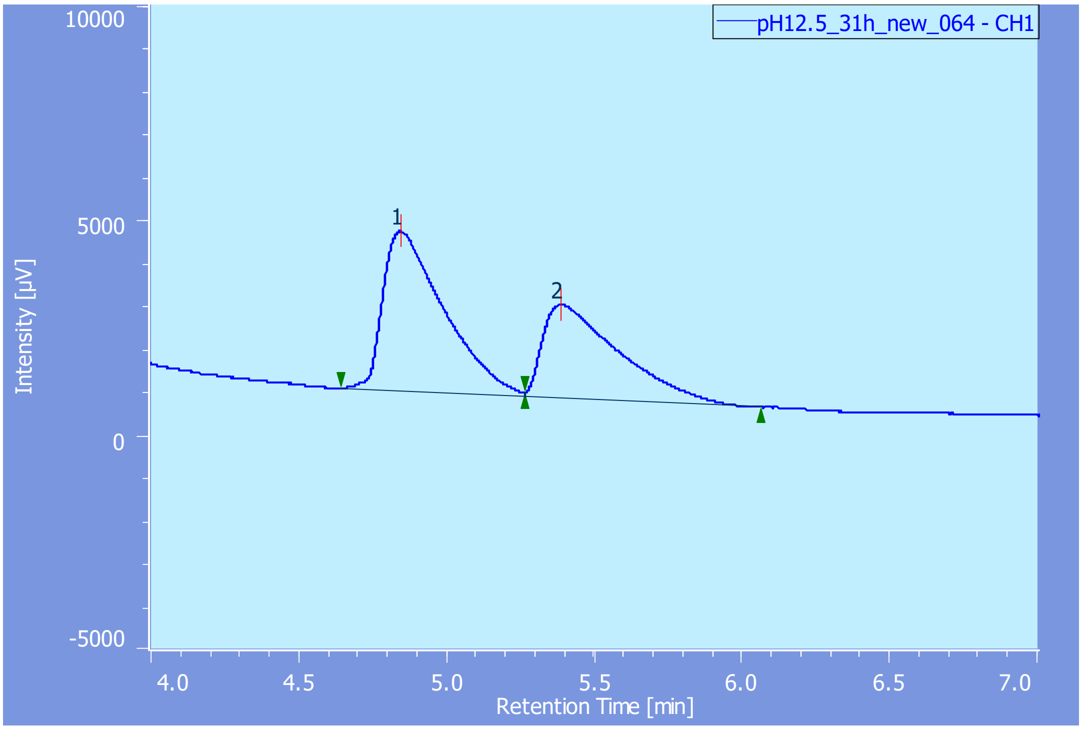


**Figure S6.49**. HPLC chart of **1** at pH 12.7 (31 h after heating)

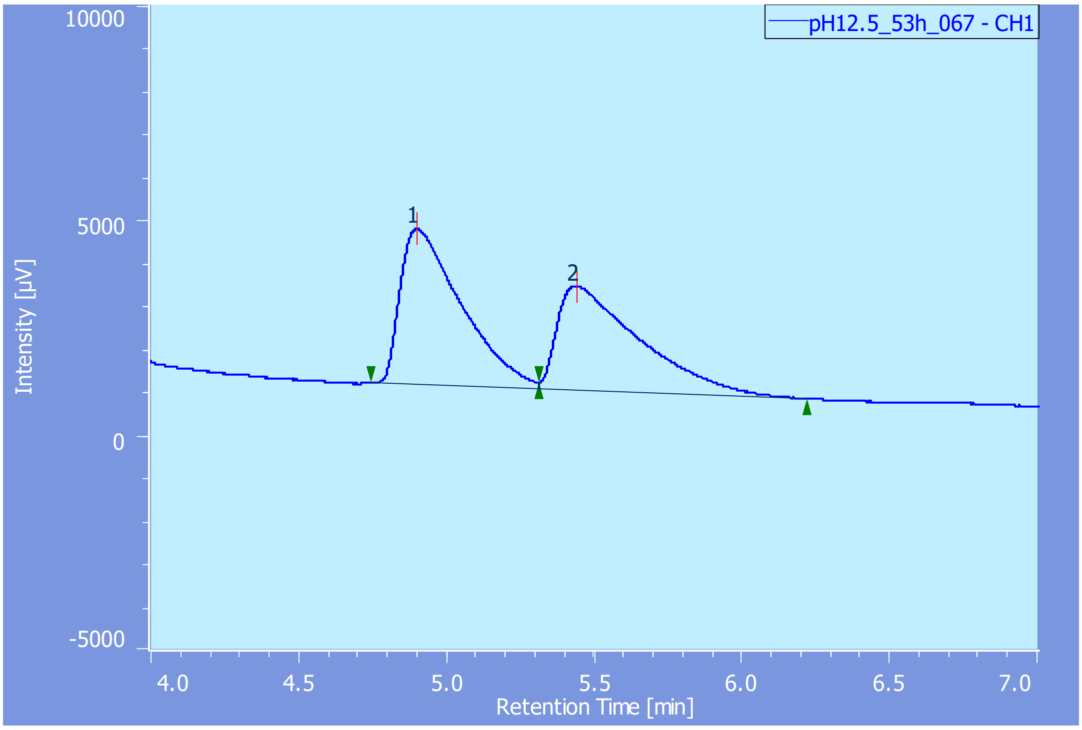


**Figure S6.50**. HPLC chart of **1** at pH 12.7 (53 h after heating)

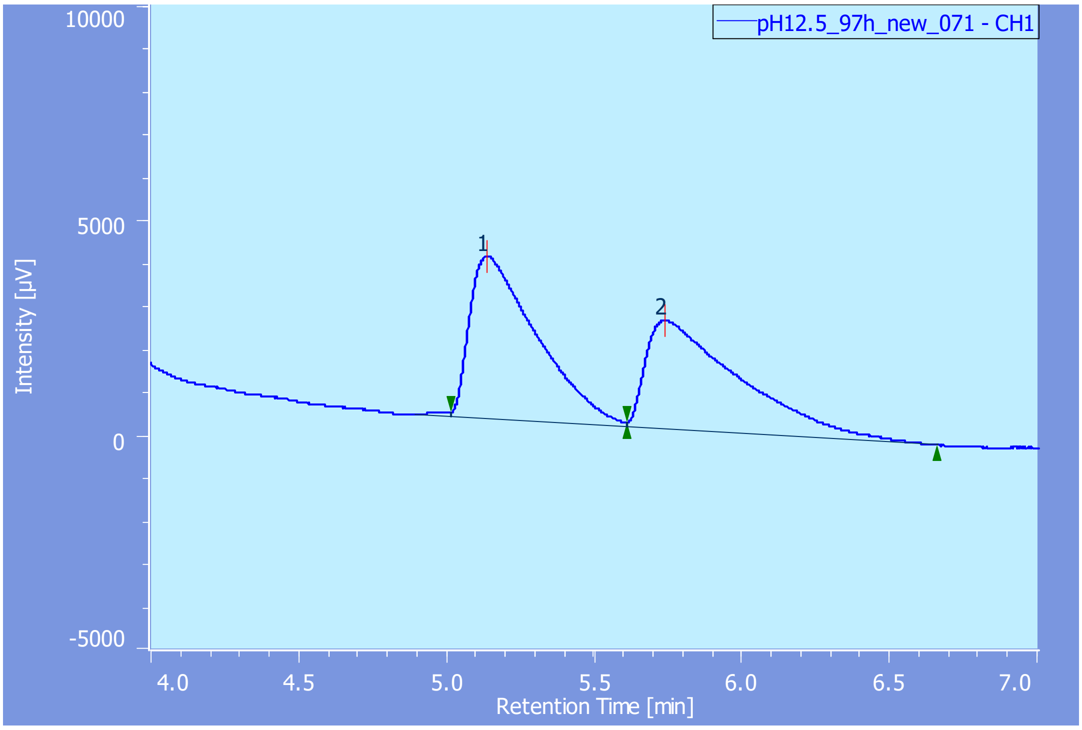


**Figure S6.51**. HPLC chart of **1** at pH 12.7 (97 h after heating)

## 7. Assignment of the *E*/*Z* configuration of benzamidines

*E* or *Z* configuration of compounds **1-7** were determined by $^{1}$H NMR and NOE experiments. The *E* and *Z* isomers can be clearly distinguished by NOE correlation from the *N*-Me groups, as shown in Figure S7.1.

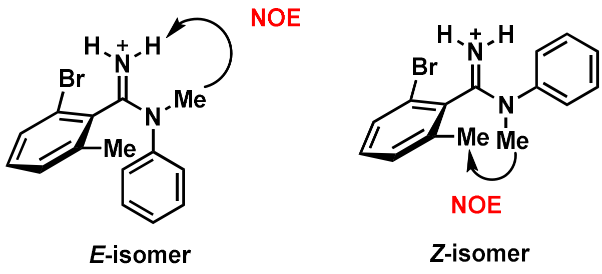


**Figure S7.1**. Determination of *E*/*Z* configuration by the NOE experiment

## 8. Quantum chemical calculations

**Rotational barriers of the thermal isomerization calculated by DFT method.**

The structures of equilibrium or transition state in a singlet state were optimized at M06-2X/Def2-SVP[SMD-water] level of theory. Vibrational calculations were performed at the same level to confirm that no imaginary frequencies (equilibrium structures) or one imaginary frequency (transition structure) exist. We also performed IRC calculation to confirm the desired connection from the transition state. Calculated equilibrium or transition state structures were shown in Figure S7.1. Gibbs free energies were calculated as the sum of the electronic energy (M06-2X/Def2-TZVP[SMD-water]) and the thermal correction term from the vibrational calculation at the same level to the optimization. All calculated energy were summarized in Table S8.1. All the DFT calculations were performed using Gaussian 16 [2].

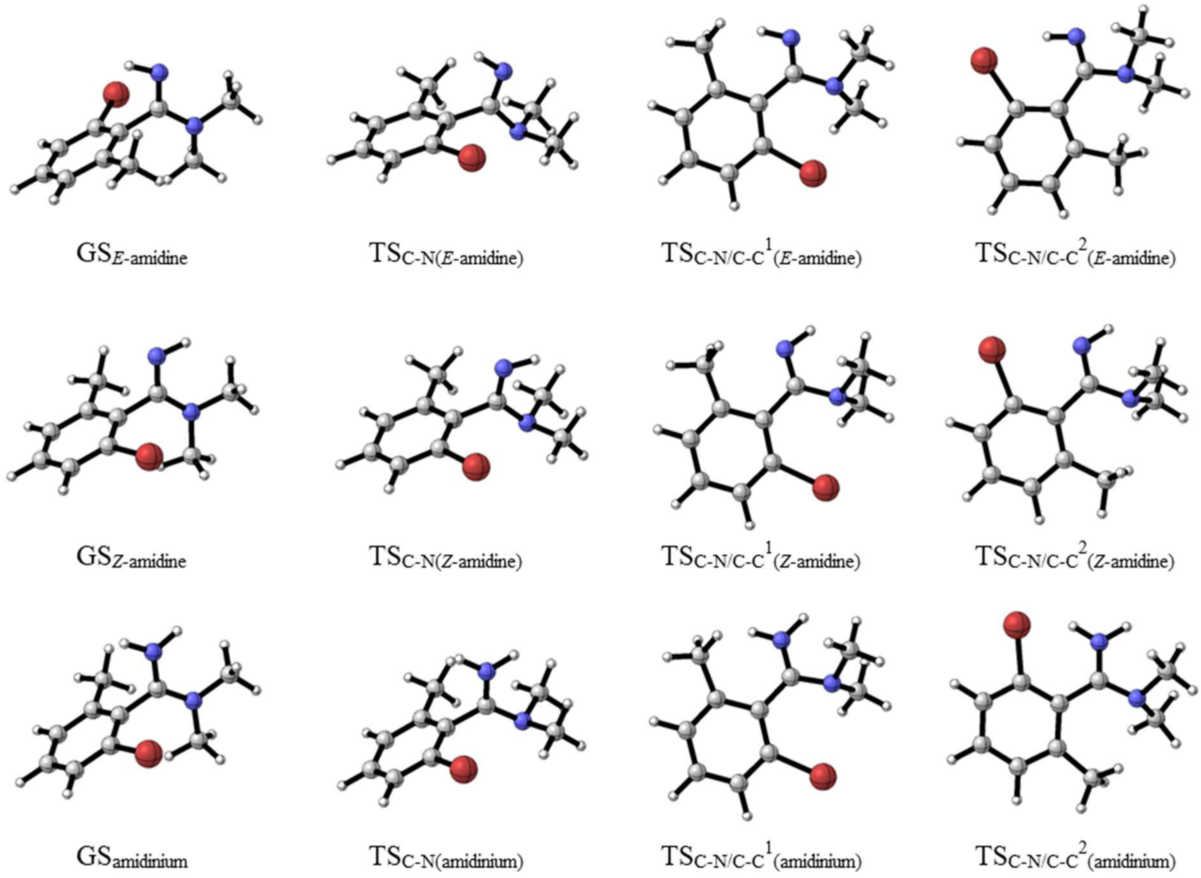


**Figure S8.1**. Equilibrium or transition state structures

**Table S8.1**. Calculated energy values of each structure in the singlet state

| structure | $E$ (hartrees) | $ZPE$ (hartrees) | $G$ (hartrees) | $\Delta G$ (kJ/mol) |
|---|---|---|---|---|
| **GS$_{E\text{-amidine}}$** | -3072.55772 | 0.172658 | -3072.385062 | 0.0 |
| TS$_{\text{C-N}(E\text{-amidine})}$ | -3072.53395 | 0.176065 | -3072.357885 | 71.4 |
| TS$_{\text{C-N/C-C}^1(E\text{-amidine})}$ | -3072.51716 | 0.177958 | -3072.339202 | 120.4 |
| TS$_{\text{C-N/C-C}^2(E\text{-amidine})}$ | -3072.55772 | 0.172658 | -3072.385062 | 131.0 |
| **GS$_{Z\text{-amidine}}$** | -3072.53362 | 0.176455 | -3072.357168 | 68.3 |
| TS$_{\text{C-N}(Z\text{-amidine})}$ | -3072.55637 | 0.173198 | -3072.383175 | 0.0 |
| TS$_{\text{C-N/C-C}^1(Z\text{-amidine})}$ | -3072.51873 | 0.177179 | -3072.341549 | 109.3 |
| TS$_{\text{C-N/C-C}^2(Z\text{-amidine})}$ | -3072.51532 | 0.176789 | -3072.338529 | 117.2 |
| **GS$_{\text{amidinium}}$** | -3073.0286 | 0.187634 | -3072.840962 | 0.0 |
| TS$_{\text{C-N(amidinium)}}$ | -3072.97991 | 0.189216 | -3072.790693 | 132.0 |
| TS$_{\text{C-N/C-C}^1\text{(amidinium)}}$ | -3072.97274 | 0.190261 | -3072.782483 | 153.5 |
| TS$_{\text{C-N/C-C}^2\text{(amidinium)}}$ | -3072.97017 | 0.190144 | -3072.780021 | 160.0 |

## Cartesian coordinates for the thermal rotational barrier

**GS$_{E\text{-amidine}}$**

| | | | |
|---|---|---|---|
| C | -2.17671 | -1.17197 | 0.239962 |
| C | -0.8659 | -0.78532 | -0.02982 |
| C | -0.50948 | 0.554875 | -0.1987 |
| C | -1.50269 | 1.546983 | -0.08733 |
| C | -2.81772 | 1.157534 | 0.184042 |
| C | -3.15398 | -0.18572 | 0.344502 |
| C | -1.1422 | 3.000066 | -0.23666 |
| Br | 0.469563 | -2.13388 | -0.15057 |
| C | 0.898699 | 0.941266 | -0.55865 |
| N | 1.767827 | 1.153537 | 0.473918 |
| C | 1.494542 | 0.709753 | 1.827797 |
| N | 1.265509 | 1.093662 | -1.78163 |
| C | 3.163274 | 1.344123 | 0.133421 |
| H | 0.459762 | 0.933907 | -2.38826 |
| H | -2.42714 | -2.22529 | 0.366218 |
| H | -3.58867 | 1.925398 | 0.270855 |
| H | -4.18579 | -0.47108 | 0.554188 |
| H | -2.04126 | 3.628243 | -0.21998 |
| H | -0.48098 | 3.320592 | 0.58239 |
| H | -0.603 | 3.175982 | -1.17761 |
| H | 0.420996 | 0.755112 | 2.044243 |
| H | 1.847672 | -0.32082 | 2.007728 |
| H | 2.007744 | 1.374177 | 2.536168 |
| H | 3.268626 | 2.124033 | -0.63027 |
| H | 3.712223 | 1.647025 | 1.033047 |
| H | 3.614736 | 0.4187 | -0.26606 |

Free energy correction (M06-2X/def2-SVP[SMD-water]): 0.172658 hartrees
*E* (M06-2X/def2-TZVP[SMD-water]): -3072.55772 hartrees

**TS$_{C\text{-}N(E\text{-amidine})}$**

| | | | |
|---|---|---|---|
| C | 0.303301 | 2.104754 | 0.080357 |
| C | 0.084623 | 0.712561 | 0.146655 |
| C | -0.773141 | 2.949528 | -0.212663 |
| H | -0.597358 | 4.026012 | -0.256123 |
| C | -1.2144 | 0.232141 | -0.064791 |
| C | -2.050325 | 2.44623 | -0.440636 |
| H | -2.876143 | 3.121159 | -0.669356 |
| C | -2.281219 | 1.076197 | -0.36261 |
| Br | -1.595034 | -1.627334 | 0.081088 |
| H | -3.278239 | 0.665981 | -0.522754 |
| C | 1.665877 | 2.704232 | 0.306389 |
| C | 1.218918 | -0.225199 | 0.474717 |
| N | 1.495925 | -0.591454 | 1.658142 |
| N | 2.000979 | -0.693602 | -0.640881 |
| C | 1.98804 | -2.153989 | -0.726288 |
| H | 2.457401 | -2.630329 | 0.153805 |
| H | 0.960643 | -2.524902 | -0.817209 |
| H | 2.544474 | -2.459038 | -1.622796 |
| C | 3.386952 | -0.234909 | -0.540528 |
| H | 3.932556 | -0.557271 | -1.437624 |
| H | 3.434372 | 0.858402 | -0.488531 |
| H | 3.899413 | -0.650086 | 0.346905 |
| H | 1.571831 | 3.749124 | 0.628513 |
| H | 2.251347 | 2.697061 | -0.62574 |
| H | 2.235922 | 2.153871 | 1.066358 |
| H | 0.839873 | -0.167165 | 2.322049 |

Free energy correction (M06-2X/def2-SVP[SMD-water]): 0.176065 hartrees
*E* (M06-2X/def2-TZVP[SMD-water]): -3072.53395 hartrees

**TS$_{C\text{-}N/C\text{-}C^1(E\text{-amidine})}$**

| | | | |
|---|---|---|---|
| C | 2.184712 | 0.70149 | -0.040586 |
| C | 0.784337 | 0.408387 | 0.025238 |
| C | 3.127616 | -0.328677 | 0.037979 |
| H | 4.185399 | -0.066255 | -0.008229 |
| C | 0.443775 | -0.971892 | 0.029445 |
| C | 2.756088 | -1.658853 | 0.156401 |
| H | 3.506369 | -2.446678 | 0.232857 |
| C | 1.408804 | -1.978084 | 0.124801 |
| Br | -1.29972 | -1.737435 | -0.166289 |
| H | 1.091434 | -3.019644 | 0.153723 |
| C | 2.790213 | 2.072441 | -0.257613 |
| C | -0.220683 | 1.548032 | 0.081295 |
| N | 0.063382 | 2.771837 | 0.298753 |
| N | -1.59131 | 1.231475 | -0.112199 |
| C | -2.341377 | 2.264154 | -0.808362 |
| H | -2.457526 | 3.188972 | -0.215741 |
| H | -1.846287 | 2.519898 | -1.75446 |
| H | -3.343483 | 1.872426 | -1.034529 |
| C | -2.223477 | 0.9357 | 1.170228 |
| H | -3.202907 | 0.462441 | 1.01119 |
| H | -1.606672 | 0.252137 | 1.767282 |
| H | -2.365818 | 1.863323 | 1.758989 |
| H | 3.822431 | 1.949259 | -0.605967 |
| H | 2.841492 | 2.663947 | 0.670018 |
| H | 2.249081 | 2.657059 | -1.012745 |
| H | 1.052146 | 2.933955 | 0.438912 |

Free energy correction (M06-2X/def2-SVP[SMD-water]): 0.177958 hartrees
*E* (M06-2X/def2-TZVP[SMD-water]): -3072.51716 hartrees

**TS$_{C\text{-}N/C\text{-}C^2(E\text{-amidine})}$**

| | | | |
|---|---|---|---|
| C | 0.810875 | 1.744 | -0.127645 |
| C | 0.220549 | 0.445785 | 0.009409 |
| C | -0.003446 | 2.88402 | -0.043175 |
| H | 0.472379 | 3.861841 | -0.125049 |
| C | -1.198872 | 0.422372 | 0.067425 |
| C | -1.377614 | 2.815408 | 0.117161 |
| H | -1.979819 | 3.722045 | 0.184587 |
| C | -1.984697 | 1.569246 | 0.137422 |
| Br | -2.29228 | -1.151946 | -0.076732 |
| H | -3.069142 | 1.481789 | 0.191541 |
| C | 2.263302 | 2.058986 | -0.422104 |

| | | | |
|---|---|---|---|
| C | 1.057118 | -0.819674 | 0.114057 |
| N | 0.654293 | -1.977451 | 0.45799 |
| N | 2.450205 | -0.683158 | -0.161736 |
| C | 3.010901 | -1.807824 | -0.895669 |
| H | 3.026692 | -2.741826 | -0.306509 |
| H | 2.434245 | -1.985407 | -1.813358 |
| H | 4.04437 | -1.560394 | -1.177705 |
| C | 3.181407 | -0.479671 | 1.085587 |
| H | 4.207159 | -0.142847 | 0.875415 |
| H | 2.68547 | 0.27382 | 1.711986 |
| H | 3.230746 | -1.417937 | 1.672366 |
| H | 2.34952 | 3.135896 | -0.611848 |
| H | 2.622279 | 1.51641 | -1.301638 |
| H | 2.938351 | 1.816889 | 0.405591 |
| H | -0.341061 | -2.013784 | 0.651653 |

Free energy correction (M06-2X/def2-SVP[SMD-water]): 0.177308 hartrees
*E* (M06-2X/def2-TZVP[SMD-water]): -3092.528427 hartrees

**GS$_{Z\text{-amidine}}$**

| | | | |
|---|---|---|---|
| C | 2.154731 | -1.222588 | 0.222919 |
| C | 0.853866 | -0.800135 | -0.042433 |
| C | 0.531229 | 0.54883 | -0.203239 |
| C | 1.549854 | 1.513333 | -0.089305 |
| C | 2.855095 | 1.089627 | 0.179012 |
| C | 3.157311 | -0.262598 | 0.331465 |
| C | 1.227758 | 2.974789 | -0.24198 |
| Br | -0.515729 | -2.114221 | -0.167324 |
| C | -0.869502 | 0.971694 | -0.551721 |
| N | -1.739259 | 1.146756 | 0.490543 |
| C | -1.450597 | 0.702762 | 1.842974 |
| N | -1.124986 | 1.154646 | -1.796468 |
| H | -2.102305 | 1.422676 | -1.931836 |
| C | -3.135129 | 1.372925 | 0.170988 |
| H | 2.377792 | -2.282816 | 0.342498 |
| H | 3.645477 | 1.837191 | 0.268876 |
| H | 4.181711 | -0.575502 | 0.538373 |
| H | 2.131431 | 3.586903 | -0.132534 |
| H | 0.780768 | 3.172068 | -1.226069 |
| H | 0.497355 | 3.29356 | 0.516345 |
| H | -1.816596 | -0.321327 | 2.030767 |
| H | -0.373099 | 0.731868 | 2.039839 |
| H | -1.939722 | 1.377978 | 2.558091 |
| H | -3.260714 | 2.271773 | -0.451003 |
| H | -3.575806 | 0.516004 | -0.368572 |
| H | -3.693745 | 1.52573 | 1.100971 |

Free energy correction (M06-2X/def2-SVP[SMD-water]): 0.173198 hartrees
*E* (M06-2X/def2-TZVP[SMD-water]): -3072.556373 hartrees

**TS$_{C\text{-}N(Z\text{-amidine})}$**

| | | | |
|---|---|---|---|
| C | 0.220189 | 2.118653 | 0.090502 |
| C | 0.05302 | 0.719747 | 0.158573 |
| C | -0.885279 | 2.924488 | -0.204622 |
| H | -0.748117 | 4.006557 | -0.249205 |
| C | -1.22701 | 0.19305 | -0.05398 |
| C | -2.14323 | 2.375177 | -0.432847 |
| H | -2.99298 | 3.019185 | -0.66328 |
| C | -2.323681 | 0.997707 | -0.35358 |
| Br | -1.541737 | -1.678846 | 0.090014 |
| H | -3.304963 | 0.551445 | -0.514083 |
| C | 1.56063 | 2.766262 | 0.315885 |
| C | 1.223852 | -0.172208 | 0.479861 |
| N | 1.460616 | -0.45211 | 1.695829 |
| N | 2.005063 | -0.623581 | -0.648086 |
| C | 2.03624 | -2.08342 | -0.739269 |
| H | 2.54192 | -2.554096 | 0.126302 |
| H | 1.020393 | -2.487091 | -0.813481 |
| H | 2.588148 | -2.367253 | -1.645236 |
| C | 3.377123 | -0.118813 | -0.574047 |
| H | 3.915171 | -0.422324 | -1.481969 |
| H | 3.388272 | 0.975276 | -0.519515 |
| H | 3.927927 | -0.519527 | 0.299021 |
| H | 1.430345 | 3.809994 | 0.629116 |
| H | 2.149875 | 2.771402 | -0.613855 |
| H | 2.144649 | 2.2404 | 1.082451 |
| H | 2.289297 | -1.058384 | 1.760181 |

Free energy correction (M06-2X/def2-SVP[SMD-water]): 0.176455 hartrees
*E* (M06-2X/def2-TZVP[SMD-water]): -3072.51248 hartrees

**TS$_{C\text{-}N/C\text{-}C^1(Z\text{-amidine})}$**

| | | | |
|---|---|---|---|
| C | 2.16804 | 0.783556 | 0.006408 |
| C | 0.782874 | 0.420654 | 0.037877 |
| C | 3.151265 | -0.211638 | -0.030636 |
| H | 4.197814 | 0.094921 | -0.057143 |
| C | 0.496449 | -0.968169 | -0.011411 |
| C | 2.83513 | -1.562105 | -0.040683 |
| H | 3.618914 | -2.320332 | -0.064234 |
| C | 1.502056 | -1.937753 | -0.03697 |
| Br | -1.233003 | -1.785227 | -0.050814 |
| H | 1.227536 | -2.991487 | -0.060701 |
| C | 2.699267 | 2.19656 | -0.022714 |
| C | -0.261699 | 1.512112 | 0.094089 |
| N | 0.087434 | 2.709779 | 0.357724 |
| N | -1.617825 | 1.164144 | -0.176728 |
| C | -2.307676 | 2.105874 | -1.043417 |
| H | -2.515661 | 3.082723 | -0.562991 |
| H | -1.722726 | 2.281105 | -1.956302 |
| H | -3.276304 | 1.673004 | -1.330582 |
| C | -2.358928 | 0.964343 | 1.063175 |
| H | -3.320872 | 0.475459 | 0.851347 |
| H | -1.795545 | 0.324613 | 1.754407 |
| H | -2.556031 | 1.929186 | 1.572154 |
| H | 3.782243 | 2.164124 | -0.196525 |
| H | 2.503881 | 2.726336 | 0.916913 |
| H | 2.226511 | 2.793793 | -0.811422 |
| H | -0.742583 | 3.311439 | 0.362286 |

Free energy correction (M06-2X/def2-SVP[SMD-water]): 0.177179 hartrees
*E* (M06-2X/def2-TZVP[SMD-water]):

-3072.518728 hartrees

**TS**$_{\mathbf{C\text{-}N/C\text{-}C^{2}}(\mathbf{\mathit{Z}\text{-}amidine})}$

| | | | |
|---|---|---|---|
| C | 0.78041 | 1.776271 | -0.083989 |
| C | 0.20433 | 0.469984 | 0.011149 |
| C | -0.055459 | 2.90196 | -0.057234 |
| H | 0.405753 | 3.888598 | -0.114779 |
| C | -1.214908 | 0.405943 | 0.037087 |
| C | -1.43584 | 2.804105 | 0.02672 |
| H | -2.058896 | 3.699171 | 0.047308 |
| C | -2.017881 | 1.546688 | 0.053633 |
| Br | -2.264011 | -1.186939 | -0.032203 |
| H | -3.101523 | 1.438689 | 0.078154 |
| C | 2.249387 | 2.10824 | -0.246761 |
| C | 1.046454 | -0.782268 | 0.092104 |
| N | 0.523648 | -1.905228 | 0.384195 |
| N | 2.450407 | -0.64224 | -0.154556 |
| C | 2.995966 | -1.669889 | -1.0274 |
| H | 3.021531 | -2.67624 | -0.563741 |
| H | 2.413344 | -1.726421 | -1.956834 |
| H | 4.031134 | -1.40295 | -1.283226 |
| C | 3.185184 | -0.58433 | 1.103943 |
| H | 4.218412 | -0.252568 | 0.923843 |
| H | 2.706707 | 0.119106 | 1.798901 |
| H | 3.217548 | -1.57563 | 1.599386 |
| H | 2.351577 | 3.197247 | -0.333143 |
| H | 2.673361 | 1.644572 | -1.143452 |
| H | 2.861129 | 1.78442 | 0.602187 |
| H | 1.256055 | -2.623089 | 0.40951 |

Free energy correction (M06-2X/def2-SVP[SMD-water]): 0.176789 hartrees
*E* (M06-2X/def2-TZVP[SMD-water]):
-3072.515318 hartrees

**GS**$_{\mathbf{amidinium}}$

| | | | |
|---|---|---|---|
| C | 2.500285 | -0.178791 | 0.226808 |
| C | 1.147685 | -0.393855 | -0.017876 |
| C | 0.26681 | 0.676452 | -0.195074 |
| C | 0.733643 | 2.000605 | -0.119921 |
| C | 2.094139 | 2.206093 | 0.12578 |
| C | 2.965887 | 1.132435 | 0.293804 |
| C | -0.219988 | 3.153974 | -0.261361 |
| Br | 0.495379 | -2.173593 | -0.087746 |
| C | -1.174981 | 0.428807 | -0.491959 |
| N | -2.051503 | 0.273231 | 0.473616 |
| C | -1.701037 | 0.324754 | 1.890014 |
| N | -1.522577 | 0.376752 | -1.765334 |
| C | -3.447355 | 0.001237 | 0.14209 |
| H | -2.481946 | 0.227901 | -2.067595 |
| H | -0.80956 | 0.507154 | -2.476742 |
| H | 3.177371 | -1.021813 | 0.362918 |
| H | 2.469504 | 3.228663 | 0.187731 |
| H | 4.024438 | 1.31429 | 0.484001 |
| H | 0.323465 | 4.106214 | -0.264268 |
| H | -0.802013 | 3.07925 | -1.190703 |
| H | -0.933441 | 3.16542 | 0.577137 |
| H | -1.873414 | -0.660038 | 2.345439 |
| H | -0.653634 | 0.612256 | 2.019863 |
| H | -2.341192 | 1.064488 | 2.387489 |
| H | -3.522078 | -0.90858 | -0.470911 |
| H | -4.004051 | -0.148313 | 1.071352 |
| H | -3.883678 | 0.848716 | -0.406411 |

Free energy correction (M06-2X/def2-SVP[SMD-water]): 0.187634 hartrees
*E* (M06-2X/def2-TZVP[SMD-water]):
-3073.028596 hartrees

**TS**$_{\mathbf{C\text{-}N(amidinium)}}$

| | | | |
|---|---|---|---|
| C | 0.14046 | 2.137094 | 0.064593 |
| C | 0.005391 | 0.736814 | 0.129441 |
| C | -1.00247 | 2.89867 | -0.201892 |
| H | -0.905277 | 3.984601 | -0.245471 |
| C | -1.252882 | 0.149843 | -0.060913 |
| C | -2.242737 | 2.30333 | -0.404027 |
| H | -3.118922 | 2.918433 | -0.612231 |
| C | -2.378478 | 0.919547 | -0.331418 |
| Br | -1.47502 | -1.731686 | 0.06969 |
| H | -3.348172 | 0.44323 | -0.475342 |
| C | 1.46413 | 2.825641 | 0.251635 |
| C | 1.203331 | -0.118667 | 0.400994 |
| N | 1.464362 | -0.435757 | 1.617141 |
| N | 2.021935 | -0.550308 | -0.656207 |
| C | 2.086905 | -2.018866 | -0.771667 |
| H | 2.58188 | -2.490271 | 0.095874 |
| H | 1.084304 | -2.439548 | -0.894597 |
| H | 2.670831 | -2.248061 | -1.670521 |
| C | 3.387125 | 0.001757 | -0.551124 |
| H | 3.923978 | -0.275077 | -1.465774 |
| H | 3.359041 | 1.093178 | -0.48127 |
| H | 3.932689 | -0.405282 | 0.318202 |
| H | 1.304822 | 3.866836 | 0.557418 |
| H | 2.024465 | 2.838456 | -0.695487 |
| H | 2.088581 | 2.33554 | 1.010934 |
| H | 2.278382 | -1.016688 | 1.848248 |
| H | 0.88038 | -0.104861 | 2.390614 |

Free energy correction (M06-2X/def2-SVP[SMD-water]): 0.189216 hartrees
*E* (M06-2X/def2-TZVP[SMD-water]):
-3072.979909 hartrees

**TS**$_{\mathbf{C\text{-}N/C\text{-}C^{1}(amidinium)}}$

| | | | |
|---|---|---|---|
| C | -2.168424 | 0.712628 | 0.010406 |
| C | -0.781867 | 0.358663 | -0.07141 |
| C | -3.133751 | -0.296377 | 0.053101 |
| H | -4.18275 | -0.007374 | 0.122099 |
| C | -0.460213 | -1.02749 | -0.013905 |
| C | -2.79215 | -1.639498 | 0.026726 |
| H | -3.564213 | -2.409462 | 0.049867 |
| C | -1.453187 | -2.002998 | 0.008855 |
| Br | 1.279243 | -1.788629 | 0.05206 |
| H | -1.170967 | -3.054727 | 0.027466 |
| C | -2.716835 | 2.115607 | 0.130405 |
| C | 0.244484 | 1.427081 | -0.151305 |

| | | | |
|---|---|---|---|
| N | -0.064711 | 2.618228 | -0.562018 |
| N | 1.566391 | 1.218703 | 0.236517 |
| C | 2.122961 | 2.272288 | 1.087707 |
| H | 2.351379 | 3.204347 | 0.539795 |
| H | 1.434841 | 2.48942 | 1.913867 |
| H | 3.064646 | 1.895042 | 1.506043 |
| C | 2.436887 | 0.96096 | -0.921852 |
| H | 3.361956 | 0.479983 | -0.579375 |
| H | 1.94481 | 0.304664 | -1.647948 |
| H | 2.683308 | 1.911913 | -1.427783 |
| H | -3.736967 | 2.059716 | 0.525254 |
| H | -2.794609 | 2.619026 | -0.84726 |
| H | -2.129857 | 2.746116 | 0.810818 |
| H | 0.658841 | 3.338108 | -0.608282 |
| H | -0.993095 | 2.881528 | -0.880522 |

Free energy correction (M06-2X/def2-SVP[SMD-water]): 0.190261 hartrees
*E* (M06-2X/def2-TZVP[SMD-water]):
-3072.972744 hartrees

**$TS_{C\text{-}N/C\text{-}C^2(amidinium)}$**

| | | | |
|---|---|---|---|
| C | 0.743622 | 1.843449 | -0.099666 |
| C | 0.207941 | 0.533036 | 0.082982 |
| C | -0.127704 | 2.942321 | -0.048786 |
| H | 0.300619 | 3.939358 | -0.156353 |
| C | -1.215699 | 0.420314 | 0.104653 |
| C | -1.494711 | 2.802836 | 0.10801 |
| H | -2.142637 | 3.678922 | 0.150002 |
| C | -2.049212 | 1.526919 | 0.136841 |
| Br | -2.184469 | -1.214809 | -0.135116 |
| H | -3.130443 | 1.395286 | 0.152267 |
| C | 2.18205 | 2.205262 | -0.373466 |
| C | 1.069044 | -0.672417 | 0.238686 |
| N | 0.624741 | -1.722023 | 0.862451 |
| N | 2.353785 | -0.74257 | -0.277415 |
| C | 2.727412 | -2.037521 | -0.852952 |
| H | 2.931722 | -2.809483 | -0.089474 |
| H | 1.94361 | -2.385583 | -1.536649 |
| H | 3.651686 | -1.886202 | -1.424258 |
| C | 3.382001 | -0.280108 | 0.675405 |
| H | 4.247127 | 0.116224 | 0.129599 |
| H | 2.98957 | 0.490075 | 1.345559 |
| H | 3.698611 | -1.132363 | 1.300212 |
| H | 2.195011 | 3.17369 | -0.887416 |
| H | 2.689278 | 1.469889 | -1.001603 |
| H | 2.754914 | 2.329388 | 0.555218 |
| H | 1.227688 | -2.537525 | 0.9826 |
| H | -0.298498 | -1.775748 | 1.283872 |

Free energy correction (M06-2X/def2-SVP[SMD-water]): 0.190144 hartrees
*E* (M06-2X/def2-TZVP[SMD-water]):
-3072.970165 hartrees

## 9. NMR spectra of the synthesized compounds

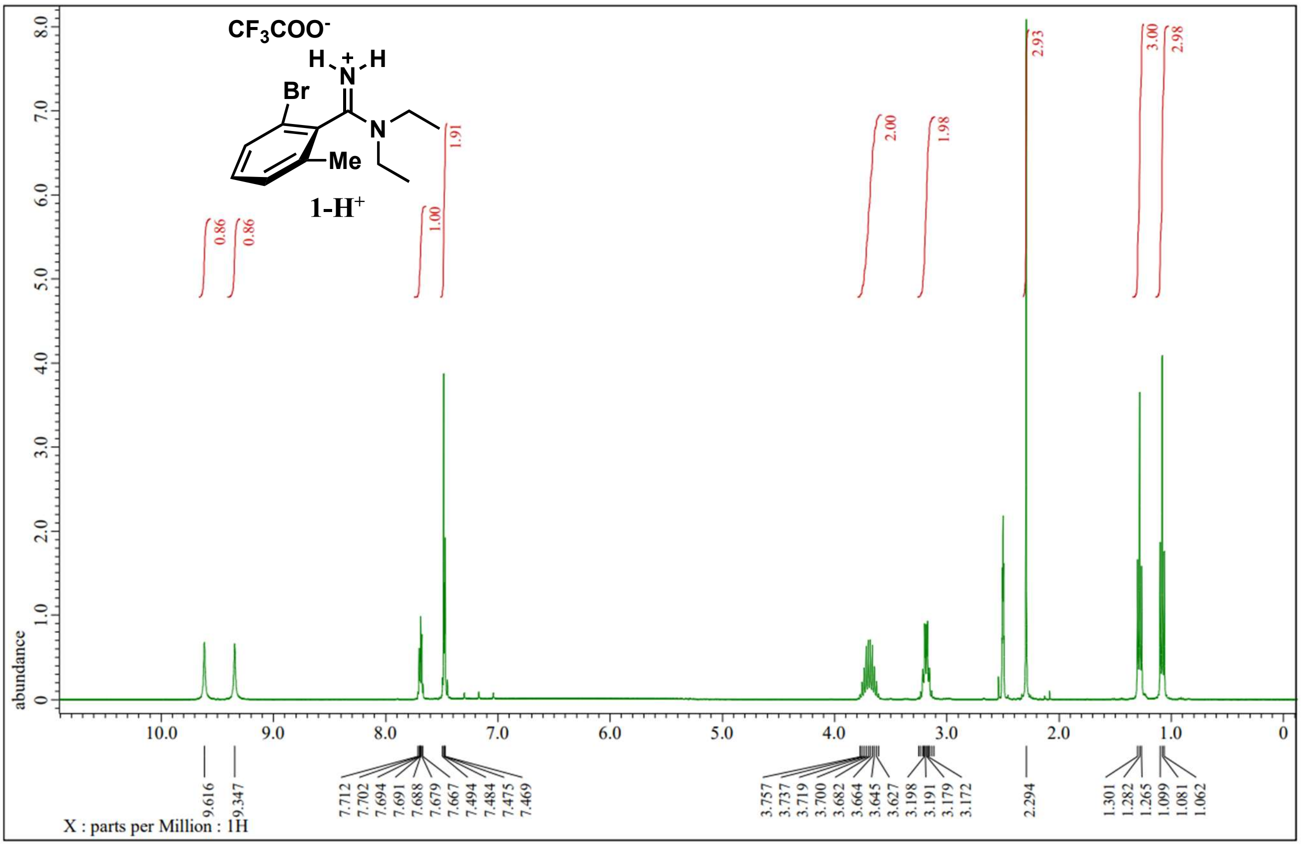


**Figure S9.1.** $^{1}$H NMR spectrum of **1** (DMSO-$d_6$, 400 MHz, 25 ˚C)

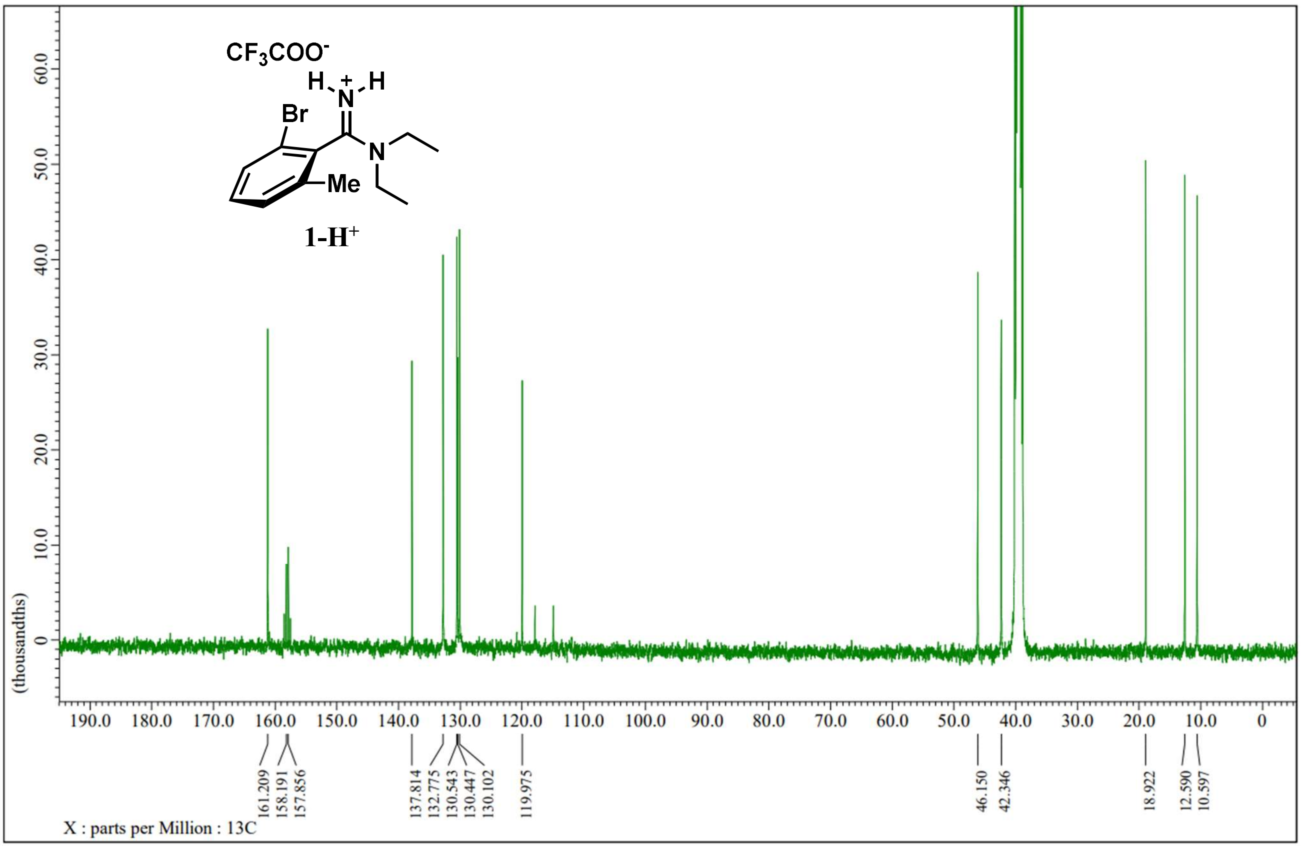


**Figure S9.2.** $^{13}$C NMR spectrum of **1** (DMSO-$d_6$, 100 MHz, 25 ˚C)

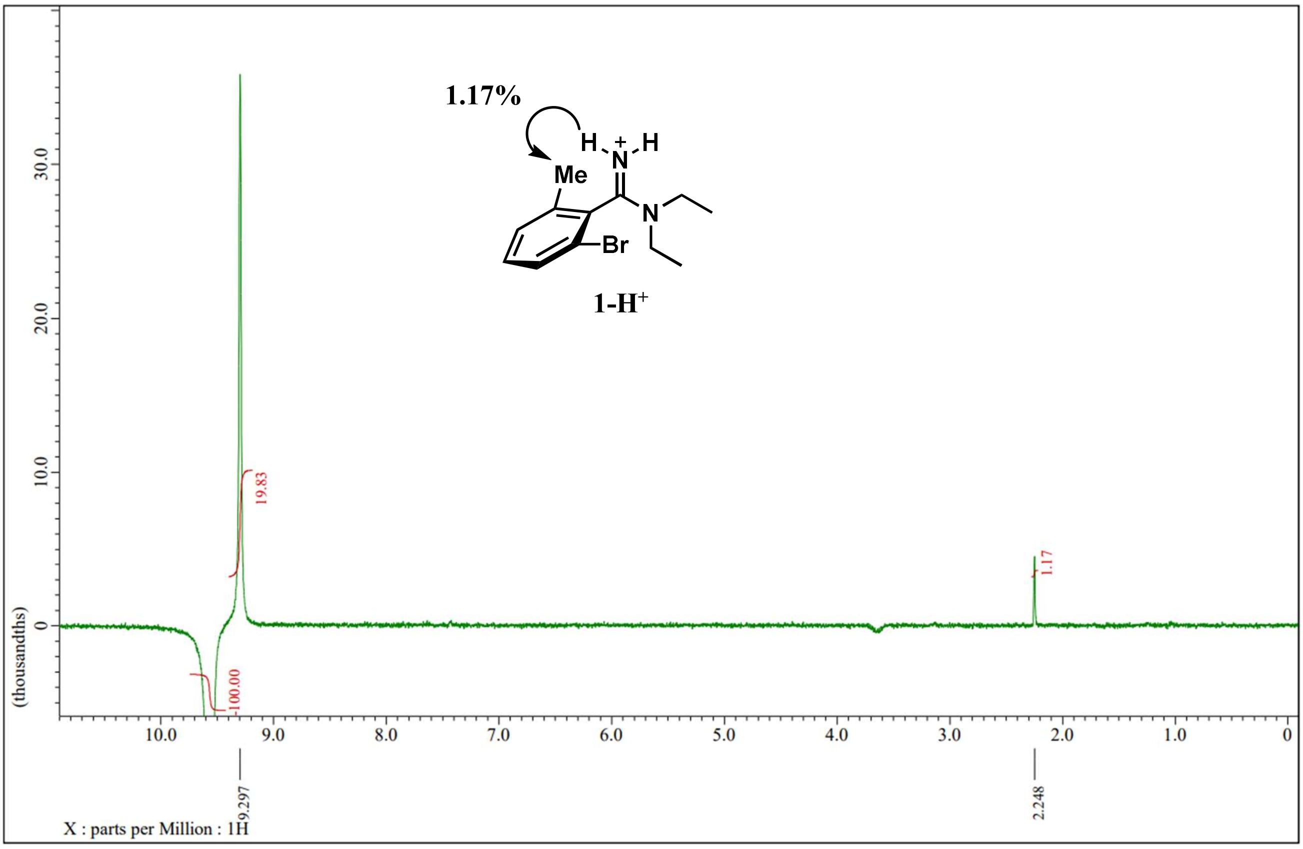


**Figure S9.3.** NOE spectrum of **1** (DMSO-$d_6$, 400 MHz, 25 ˚C)

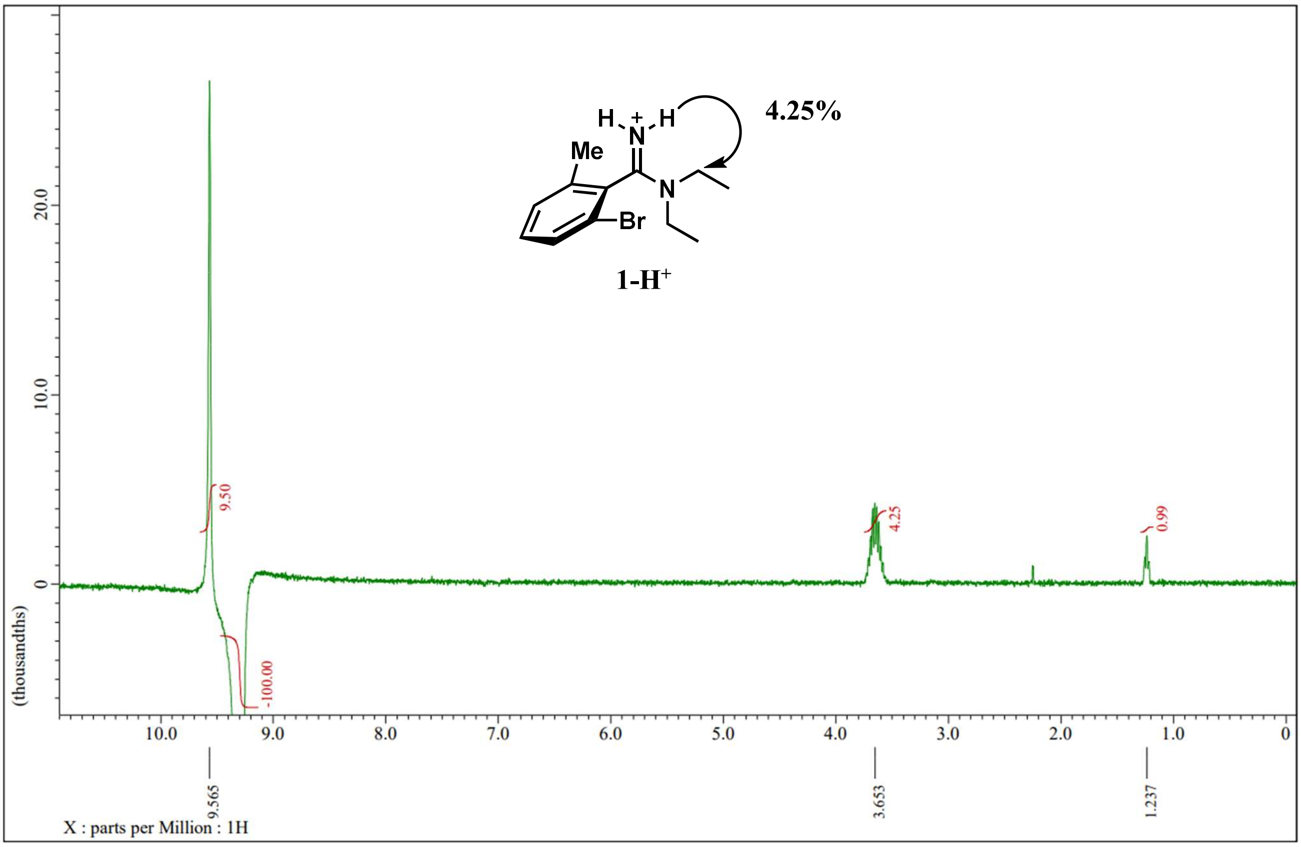


**Figure S9.4.** NOE spectrum of **1** (DMSO-$d_6$, 400 MHz, 25 ˚C)

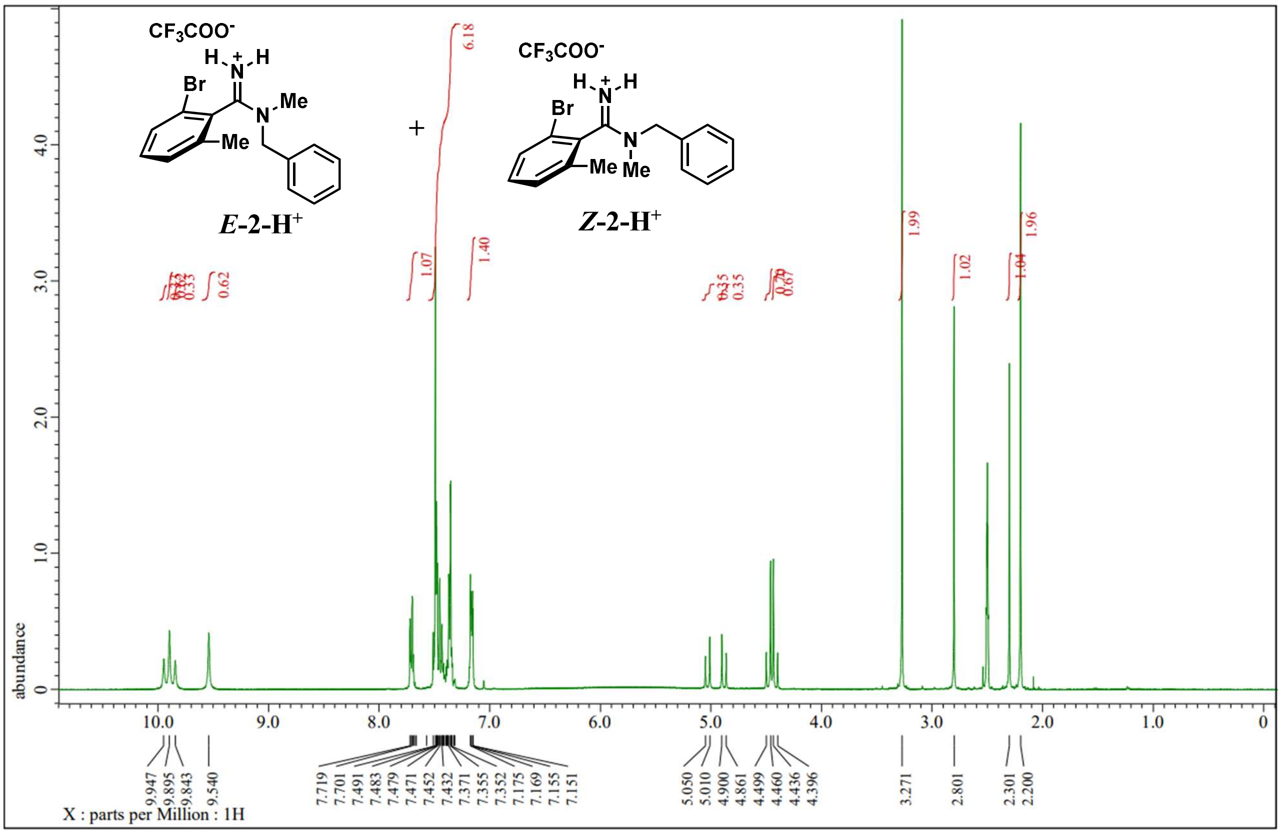

**Figure S9.5.** $^{1}$H NMR spectrum of **2** (DMSO-$d_6$, 400 MHz, 25 ˚C)

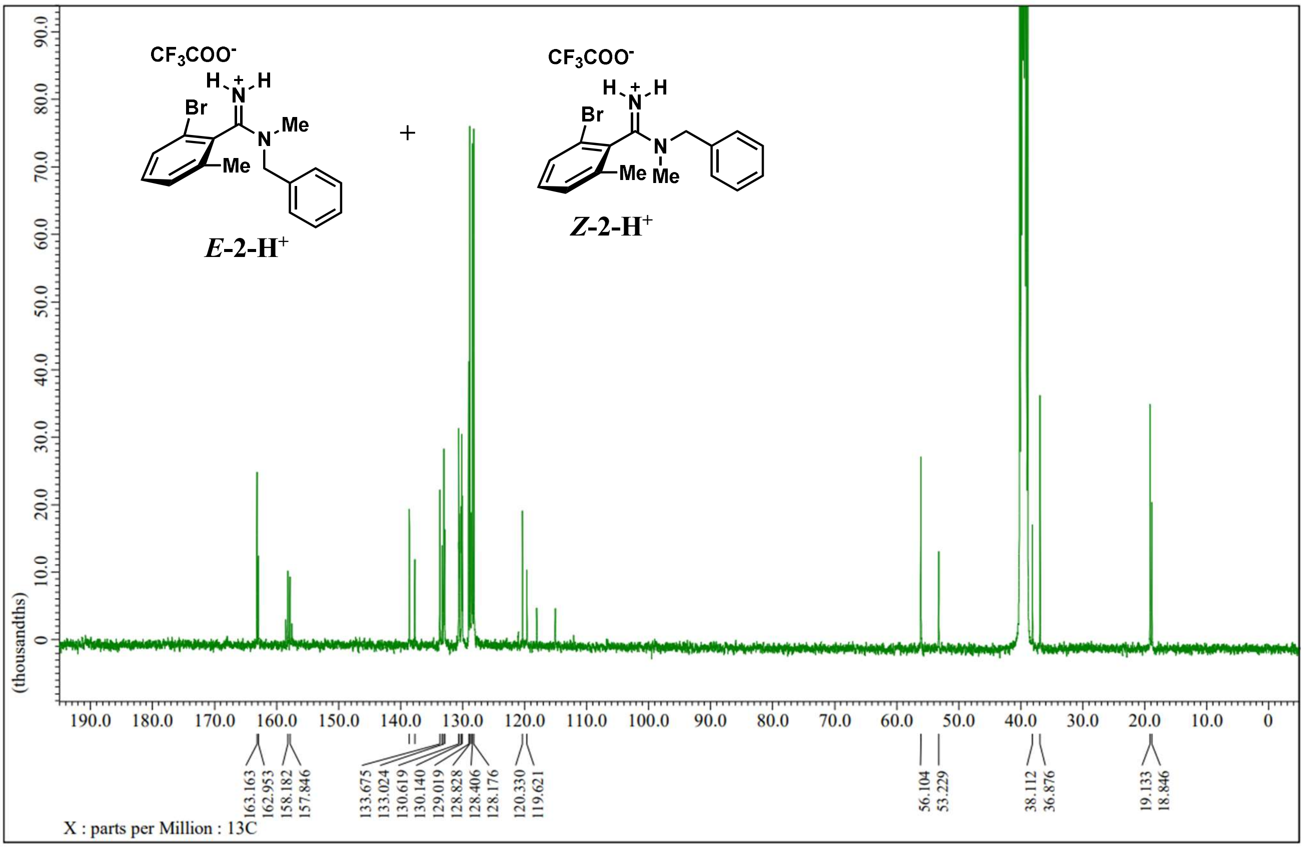

**Figure S9.6.** $^{13}$C NMR spectrum of **2** (DMSO-$d_6$, 100 MHz, 25 ˚C)

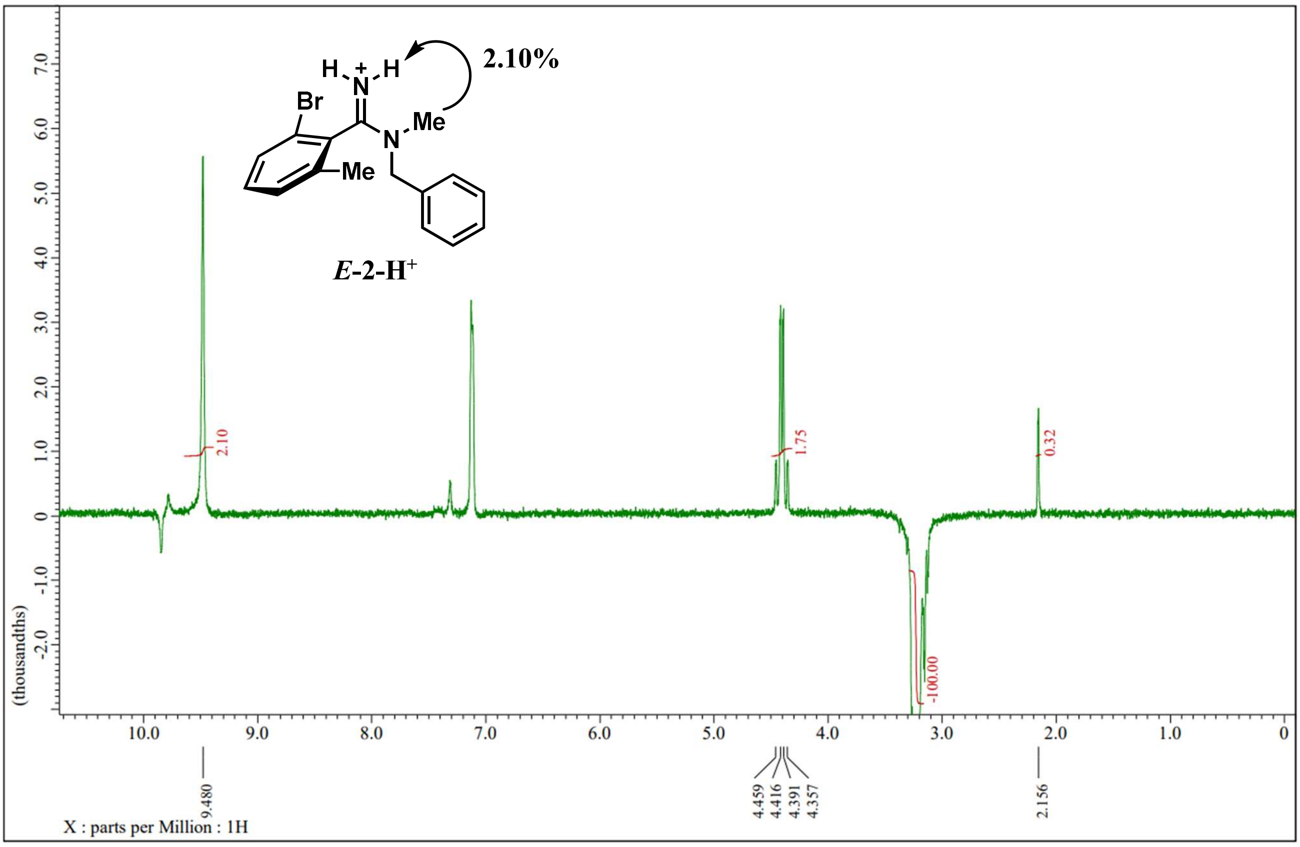


**Figure S9.7.** NOE spectrum of ***E*-2** (DMSO-$d_6$, 400 MHz, 25 ˚C)

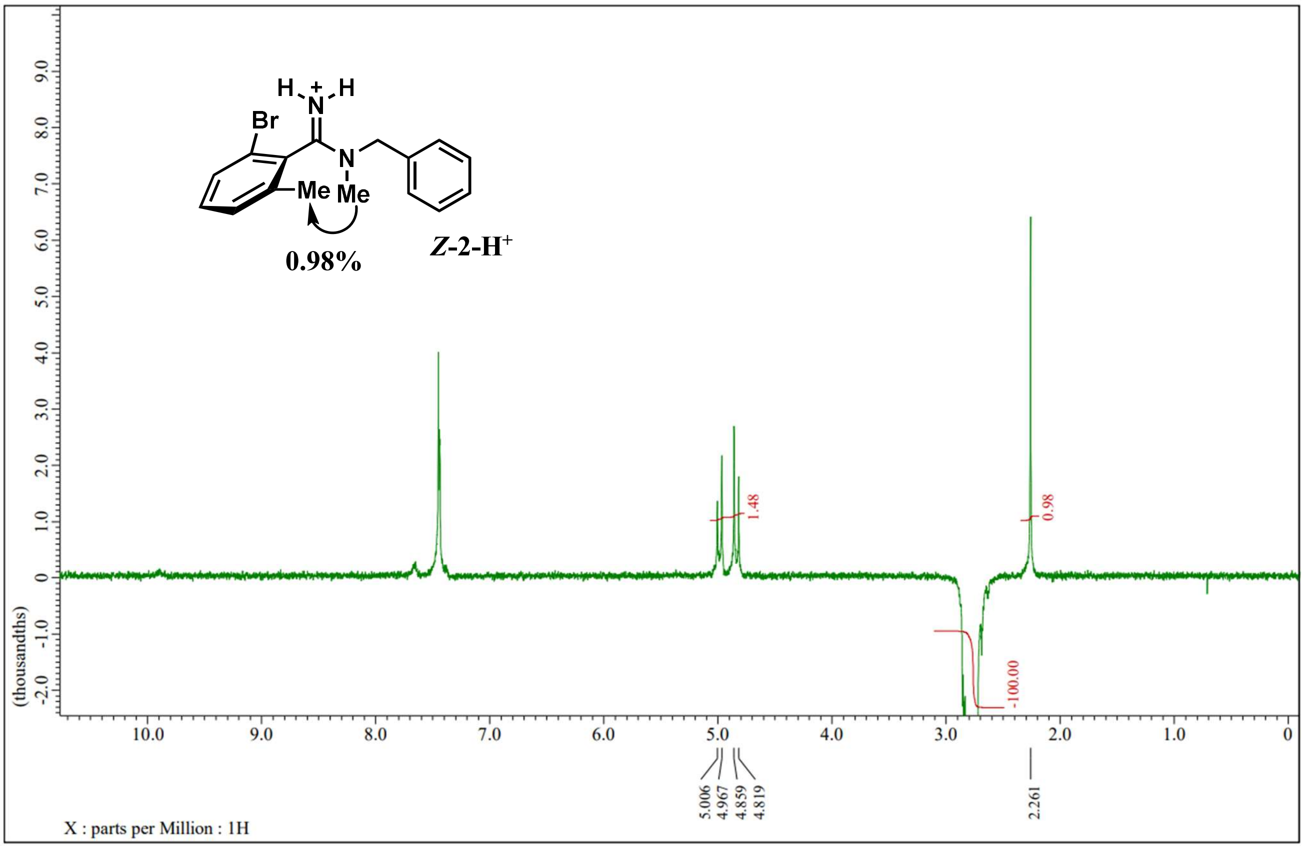


**Figure S9.8.** NOE spectrum of ***Z*-2** (DMSO-$d_6$, 400 MHz, 25 ˚C)

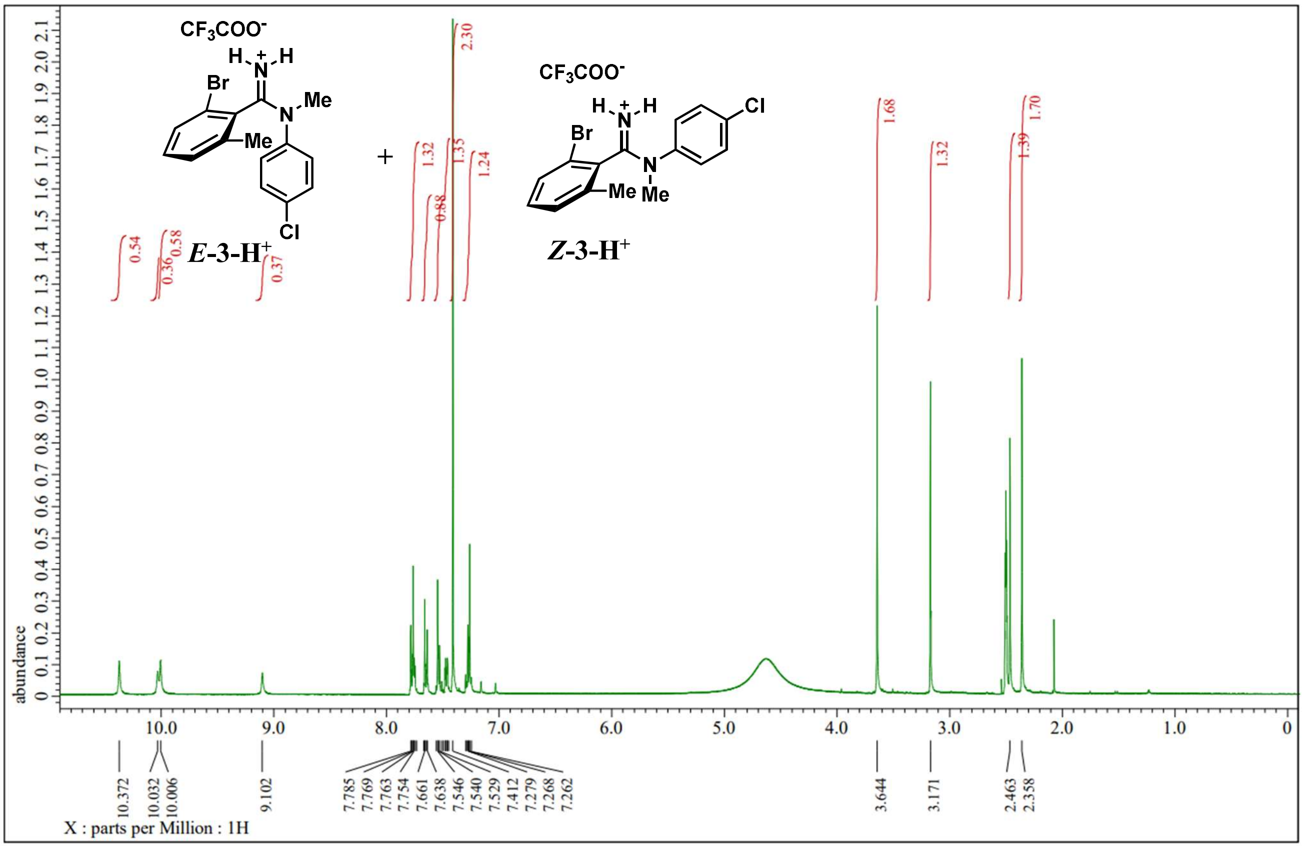

**Figure S9.9.** $^{1}$H NMR spectrum of **3** (DMSO-$d_6$, 400 MHz, 25 ˚C)

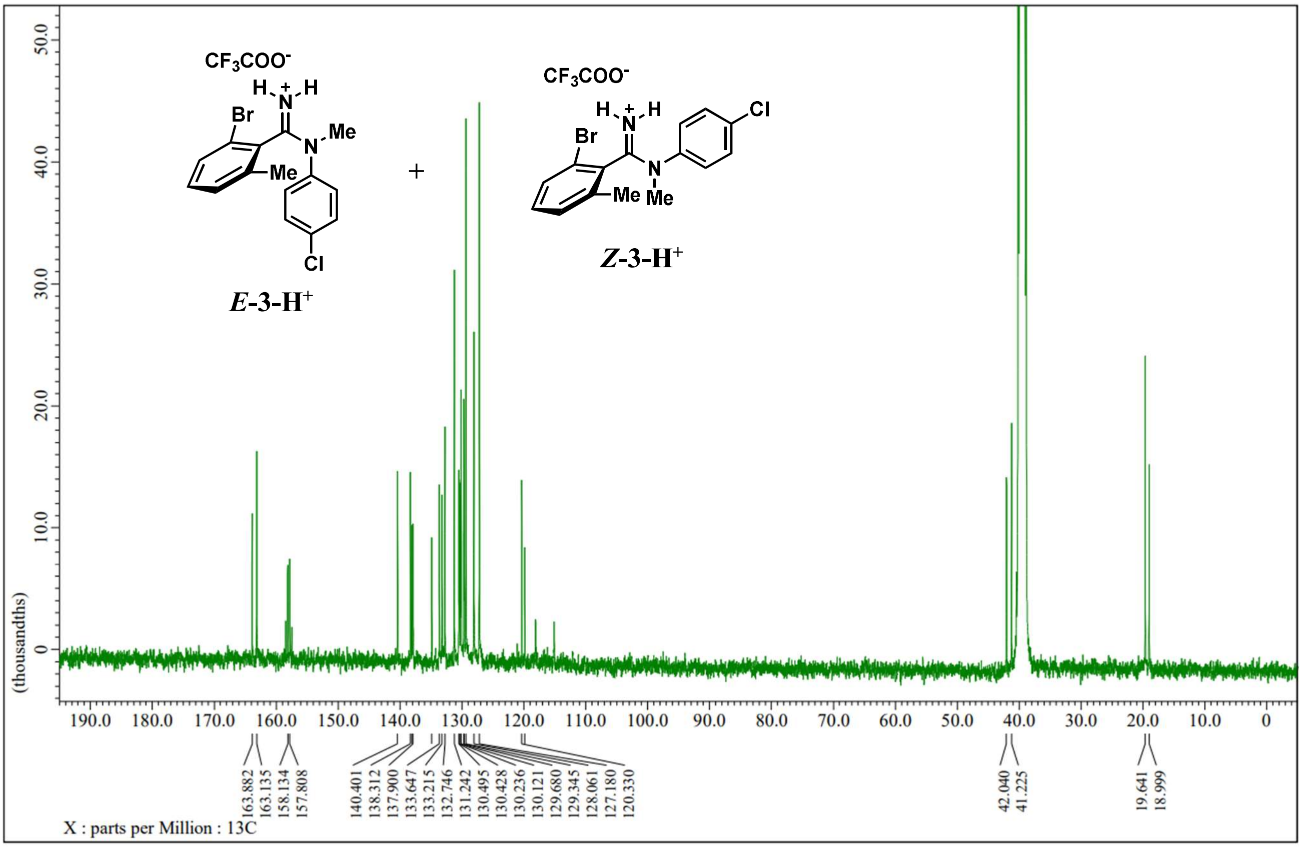

**Figure S9.10.** $^{13}$C NMR spectrum of **3** (DMSO-$d_6$, 100 MHz, 25 ˚C)

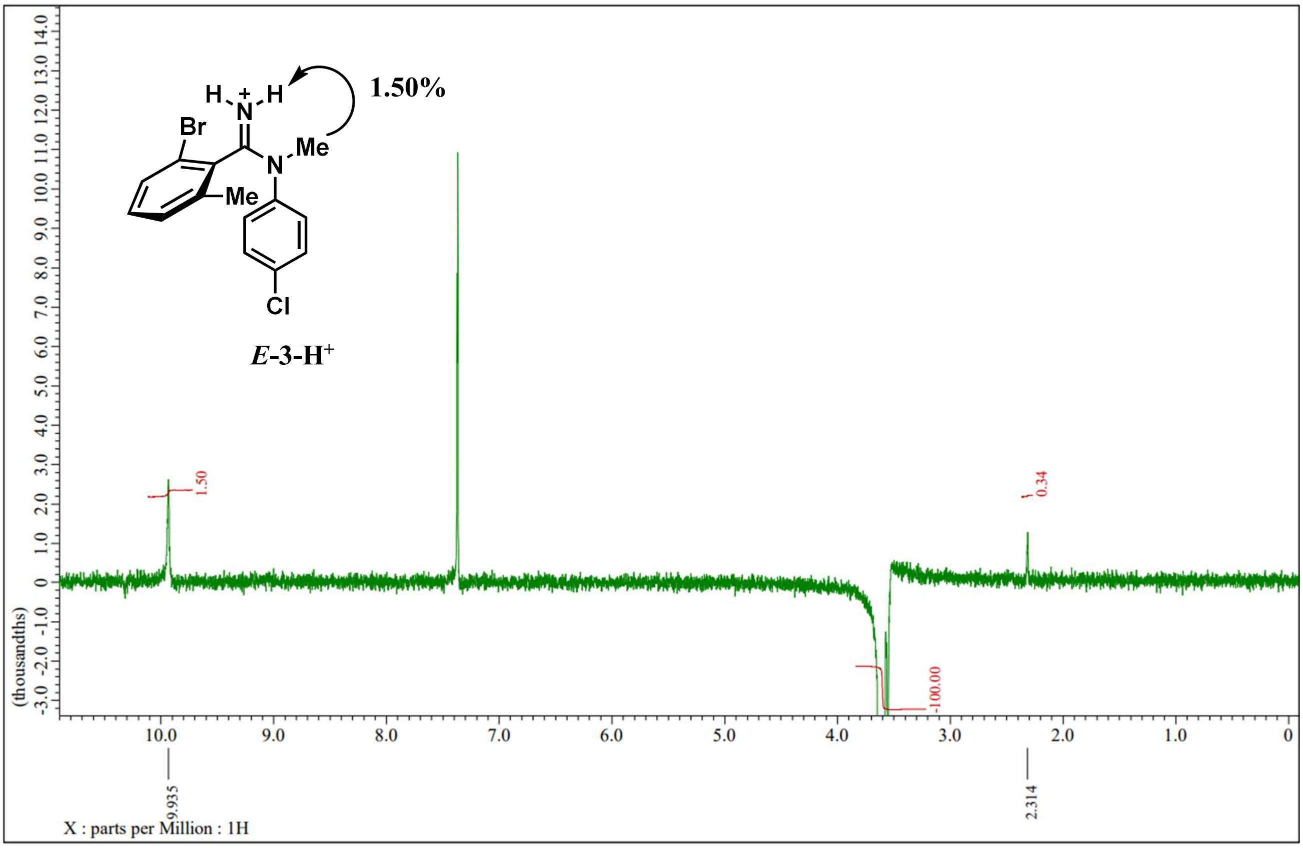


**Figure S9.11.** NOE spectrum of ***E*-3** (DMSO-$d_6$, 400 MHz, 25 ˚C)

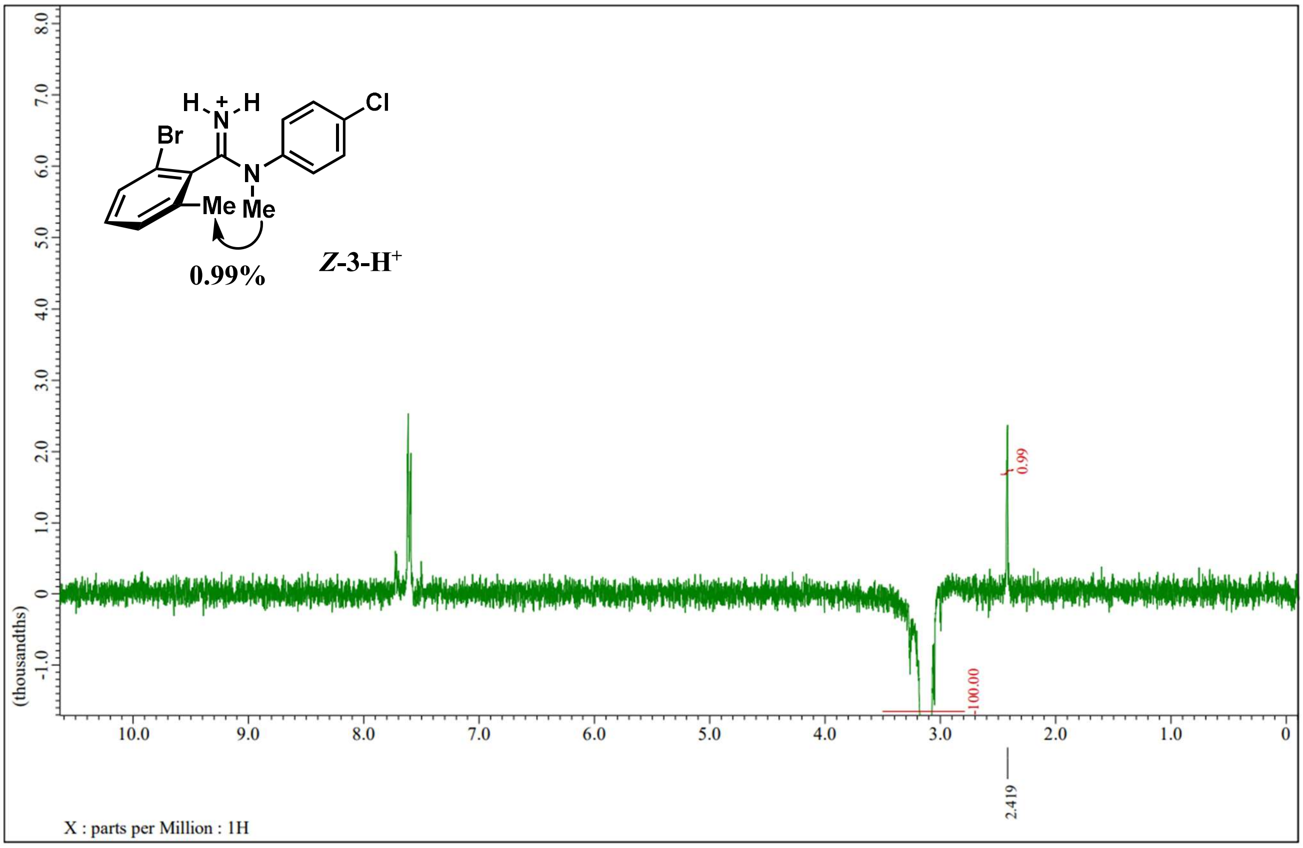


**Figure S9.12.** NOE spectrum of ***Z*-3** (DMSO-$d_6$, 400 MHz, 25 ˚C)

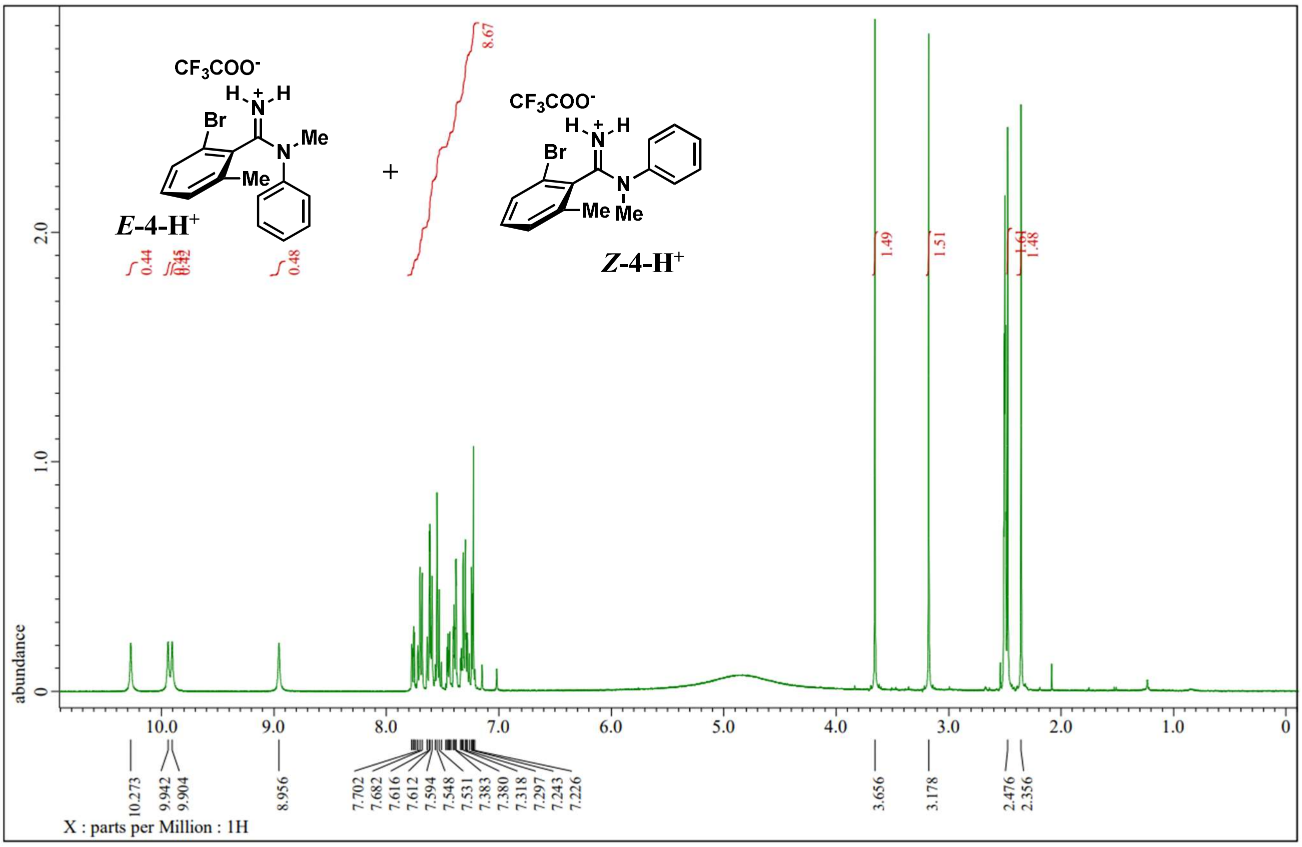


**Figure S9.13.** $^{1}H$ NMR spectrum of **4** (DMSO-$d_6$, 400 MHz, 25 ˚C)

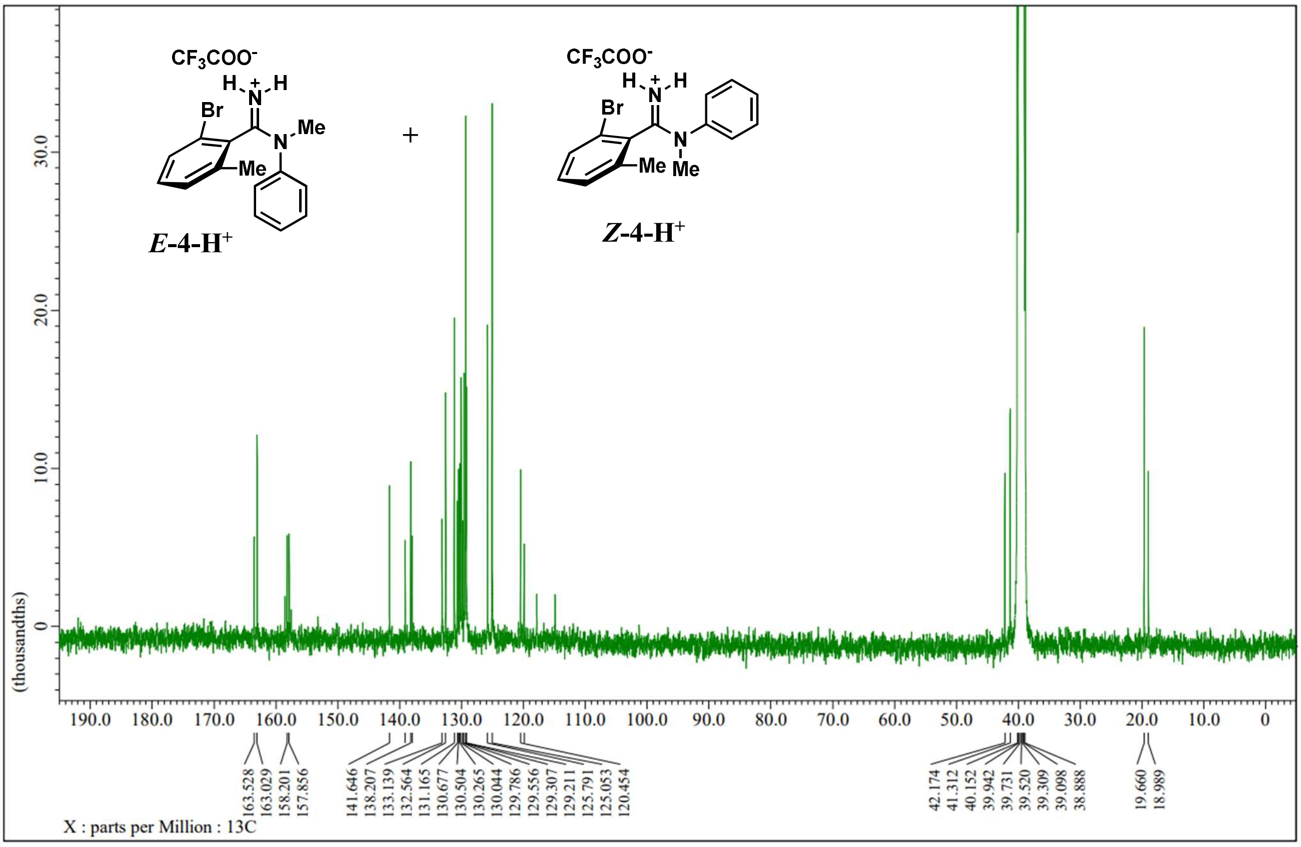


**Figure S9.14.** $^{13}C$ NMR spectrum of **4** (DMSO-$d_6$, 100 MHz, 25 ˚C)

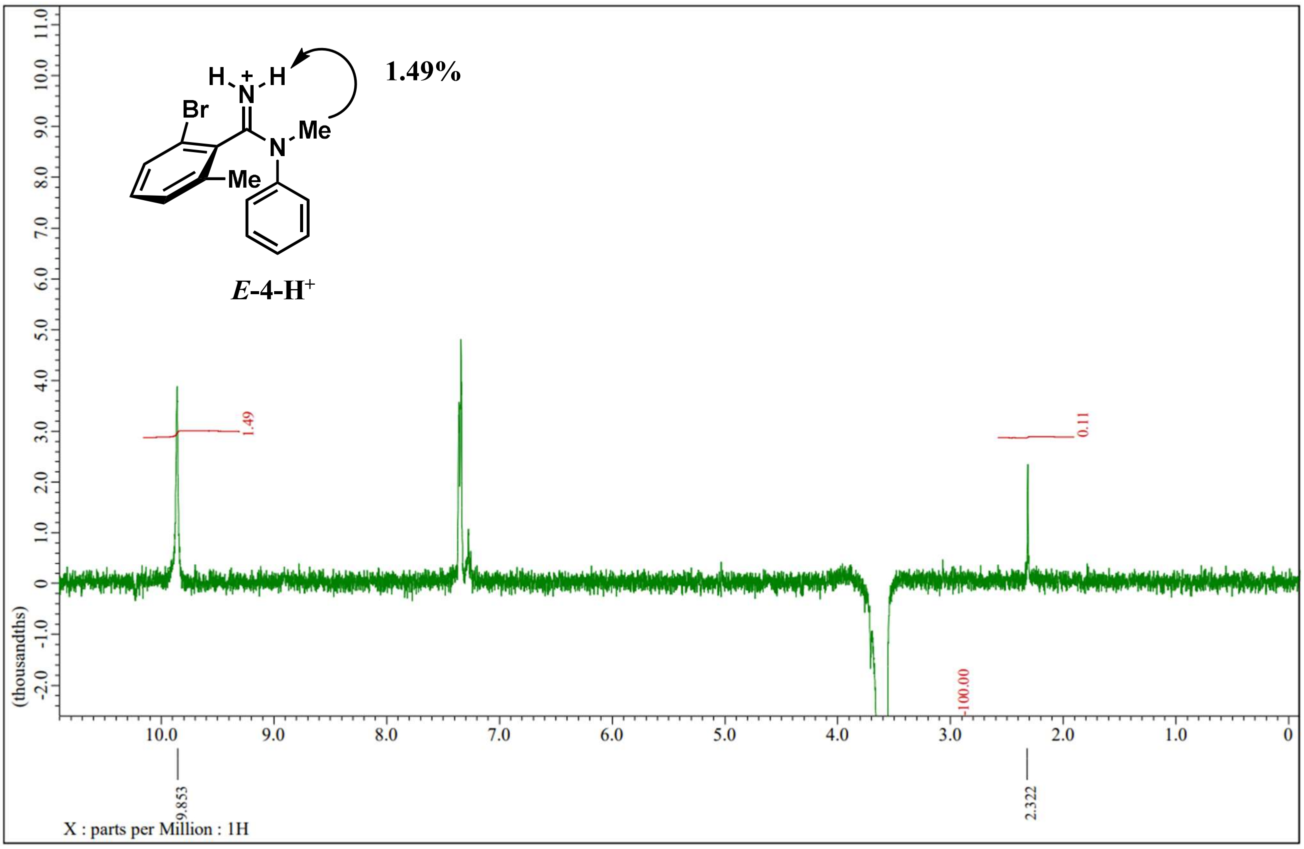


**Figure S9.15.** NOE spectrum of ***E*-4** (DMSO-$d_6$, 400 MHz, 25 ˚C)

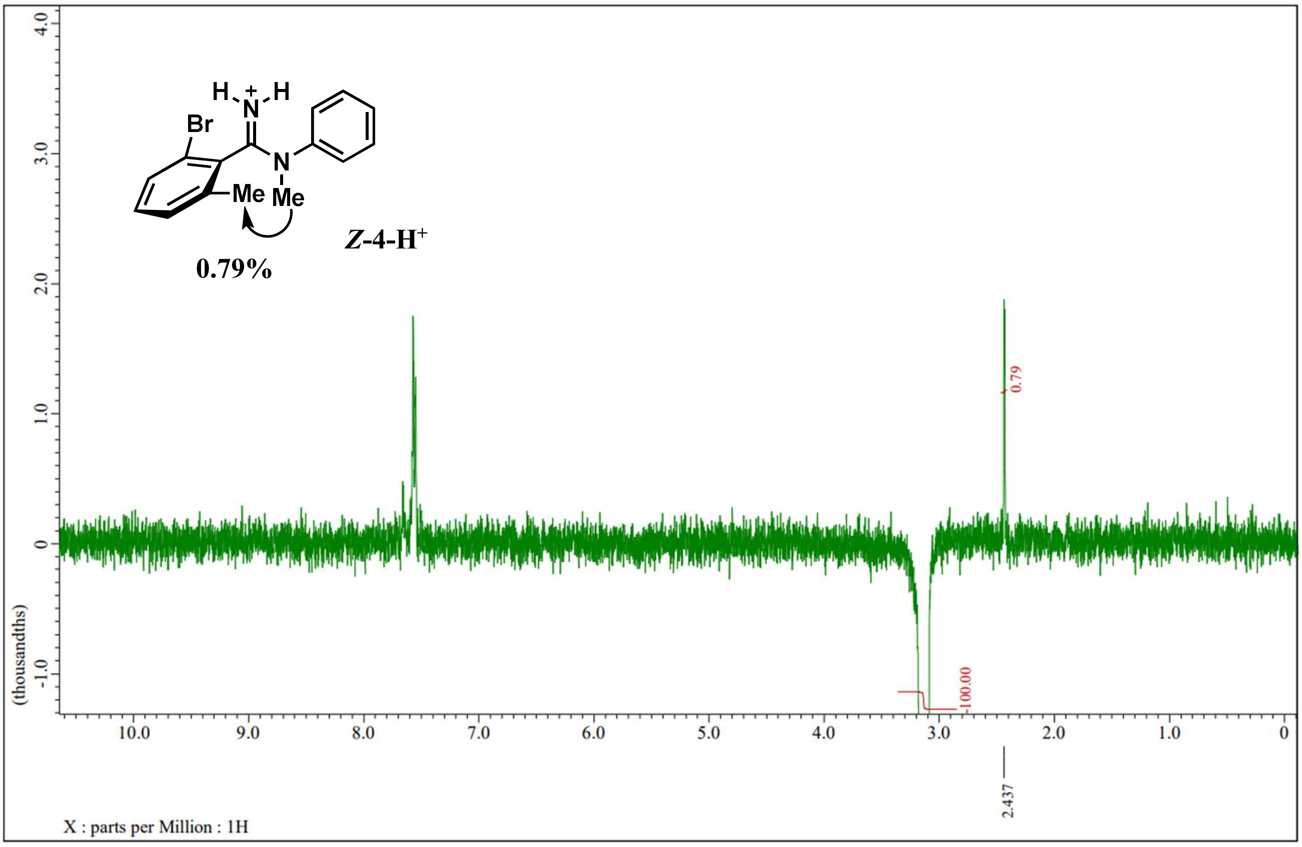


**Figure S9.16.** NOE spectrum of ***Z*-4** (DMSO-$d_6$, 400 MHz, 25 ˚C)

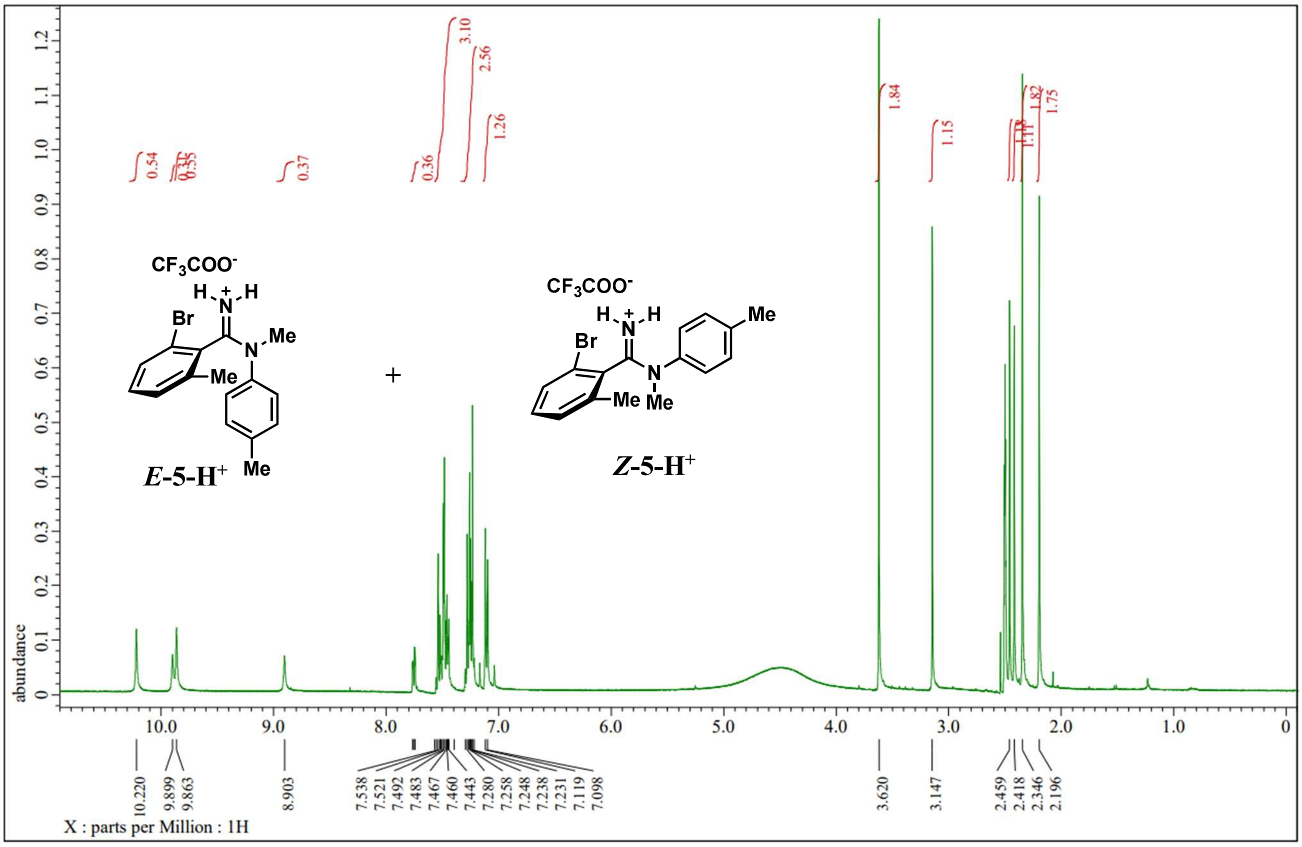


**Figure S9.17.** $^{1}$H NMR spectrum of **5** (DMSO-$d_6$, 400 MHz, 25 ˚C)

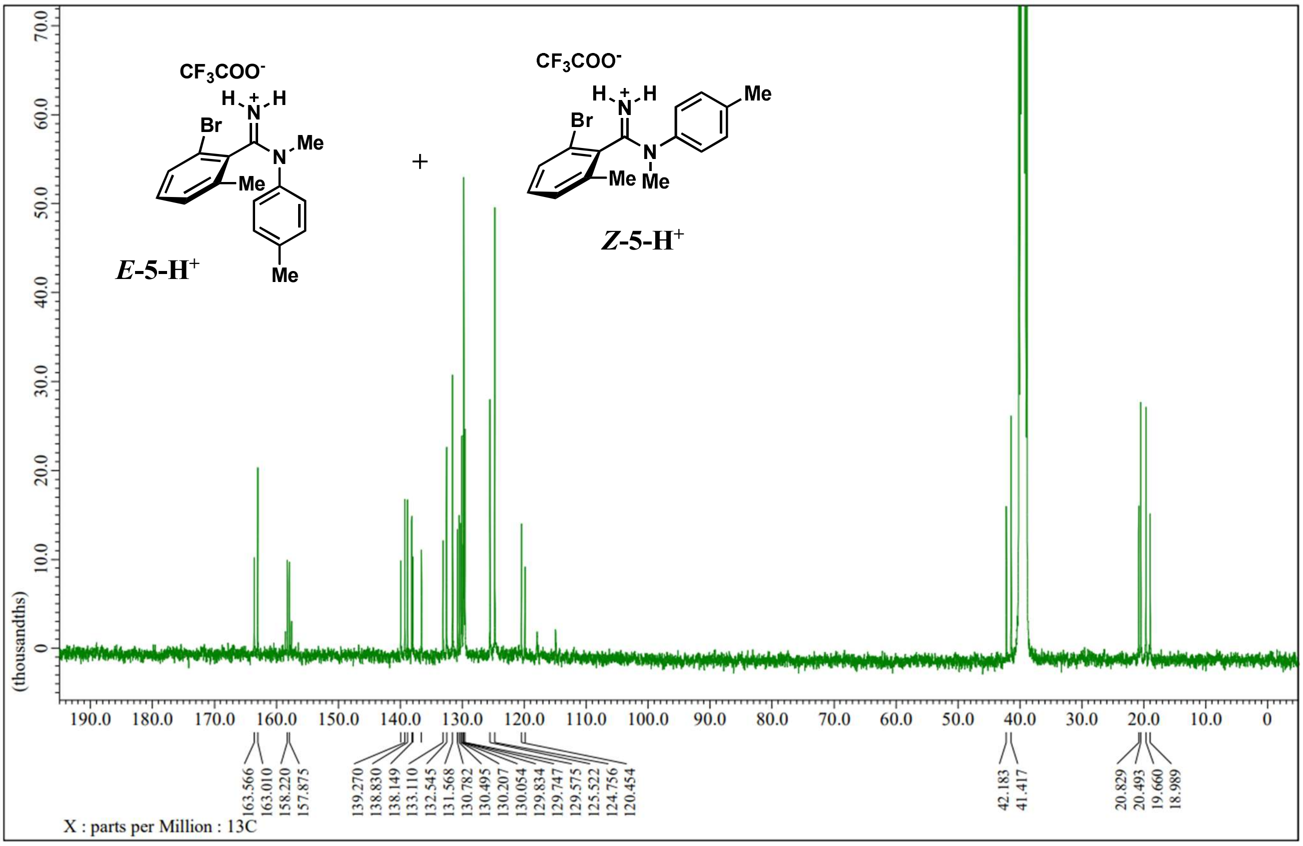


**Figure S9.18.** $^{13}$C NMR spectrum of **5** (DMSO-$d_6$, 100 MHz, 25 ˚C)

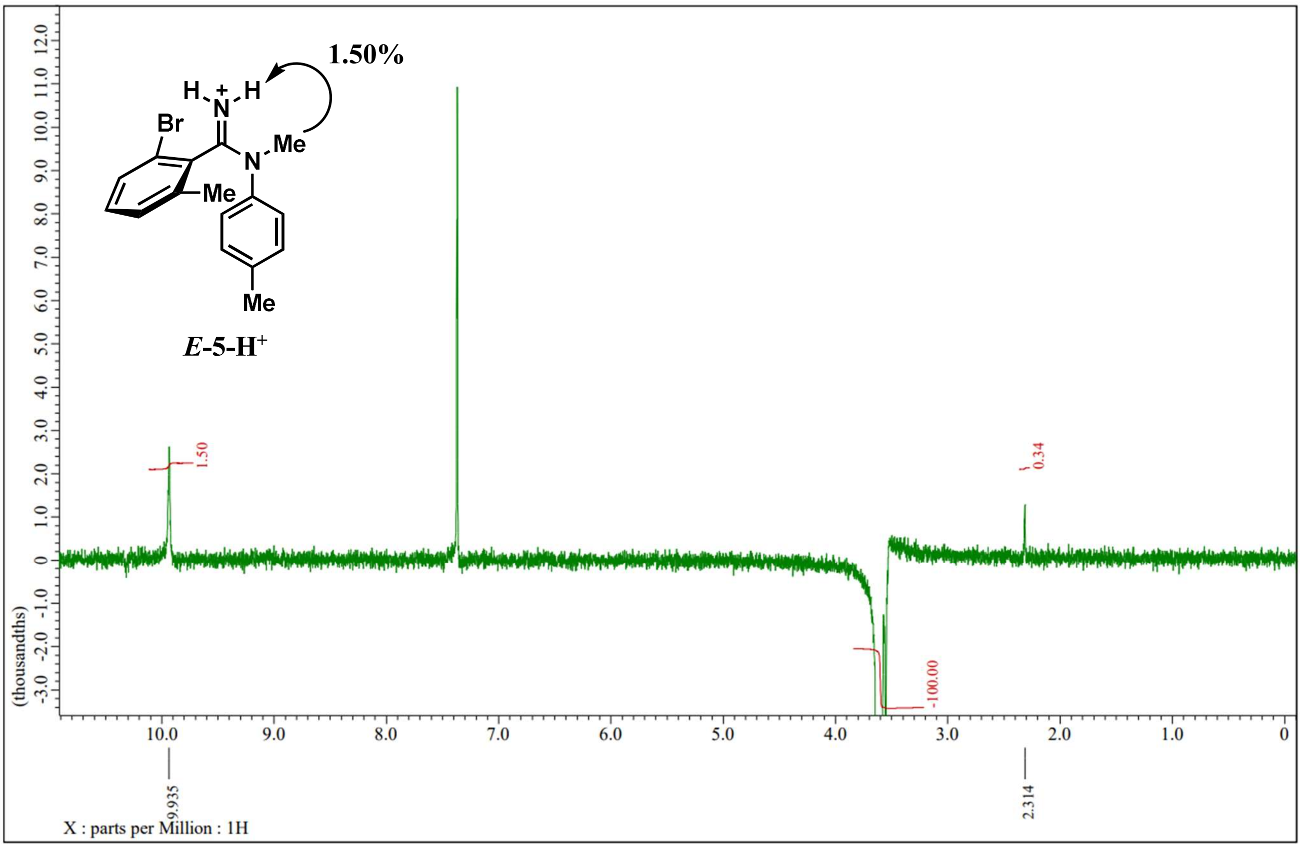


**Figure S9.19.** NOE spectrum of ***E*-5** (DMSO-$d_6$, 400 MHz, 25 ˚C)

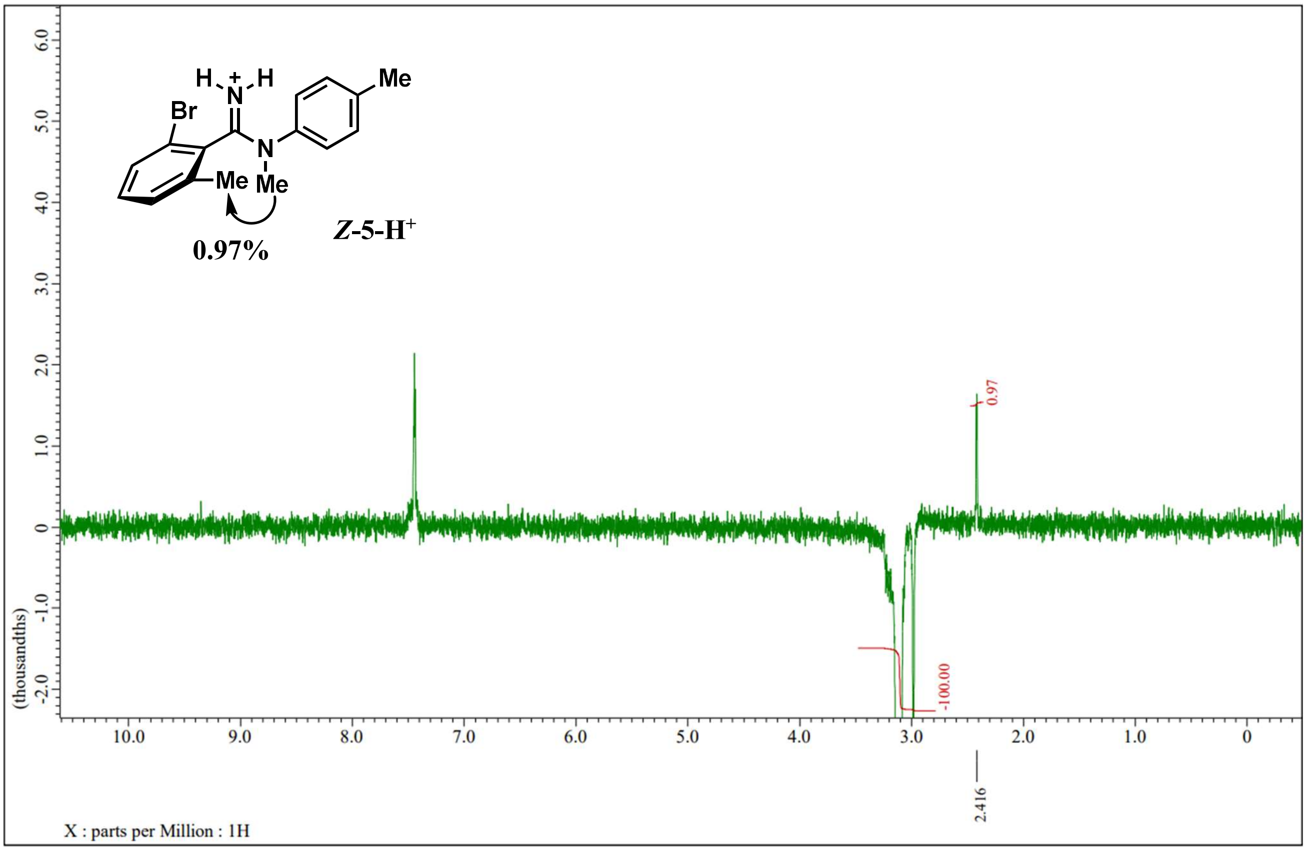


**Figure S9.20.** NOE spectrum of ***Z*-5** (DMSO-$d_6$, 400 MHz, 25 ˚C)

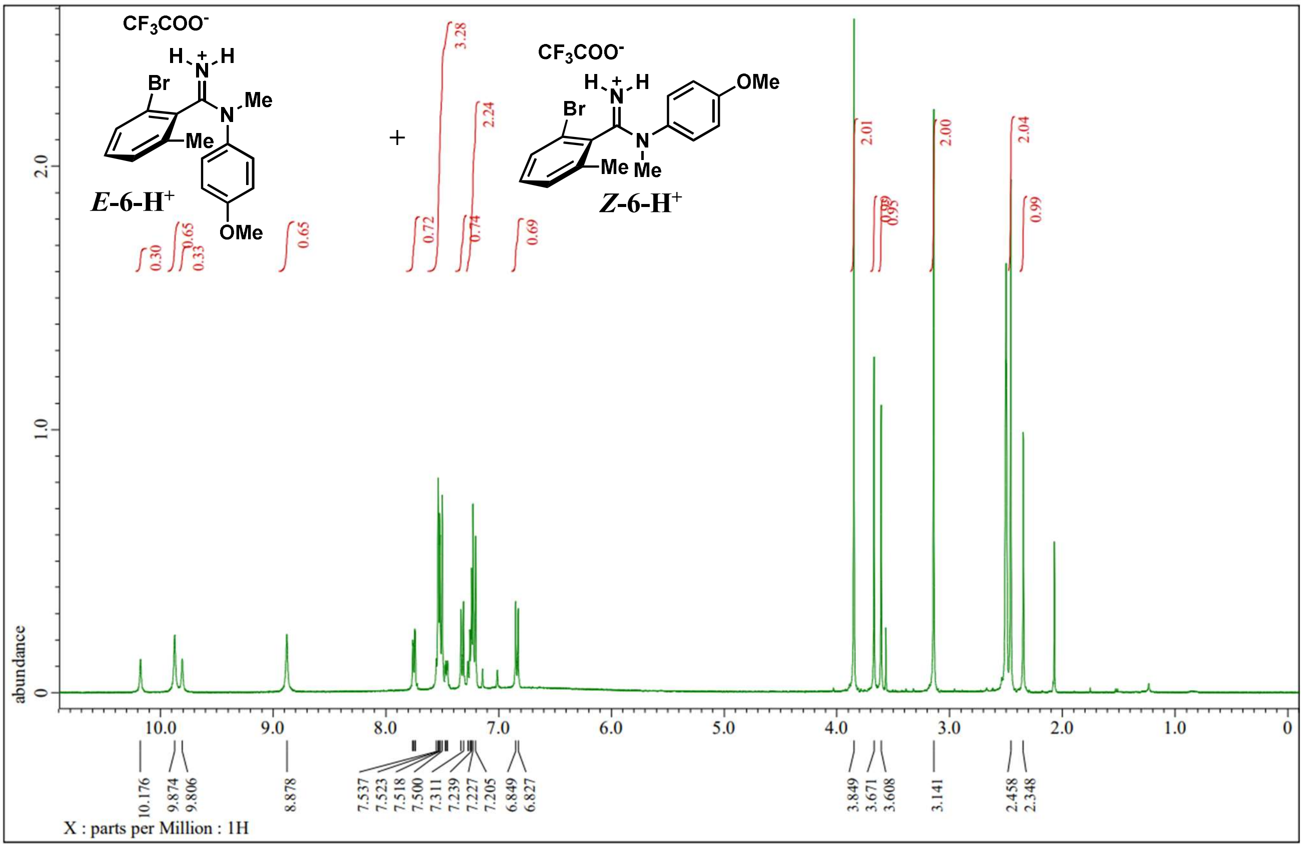


**Figure S9.21.** $^{1}$H NMR spectrum of **6** (DMSO-$d_6$, 400 MHz, 25 ˚C)

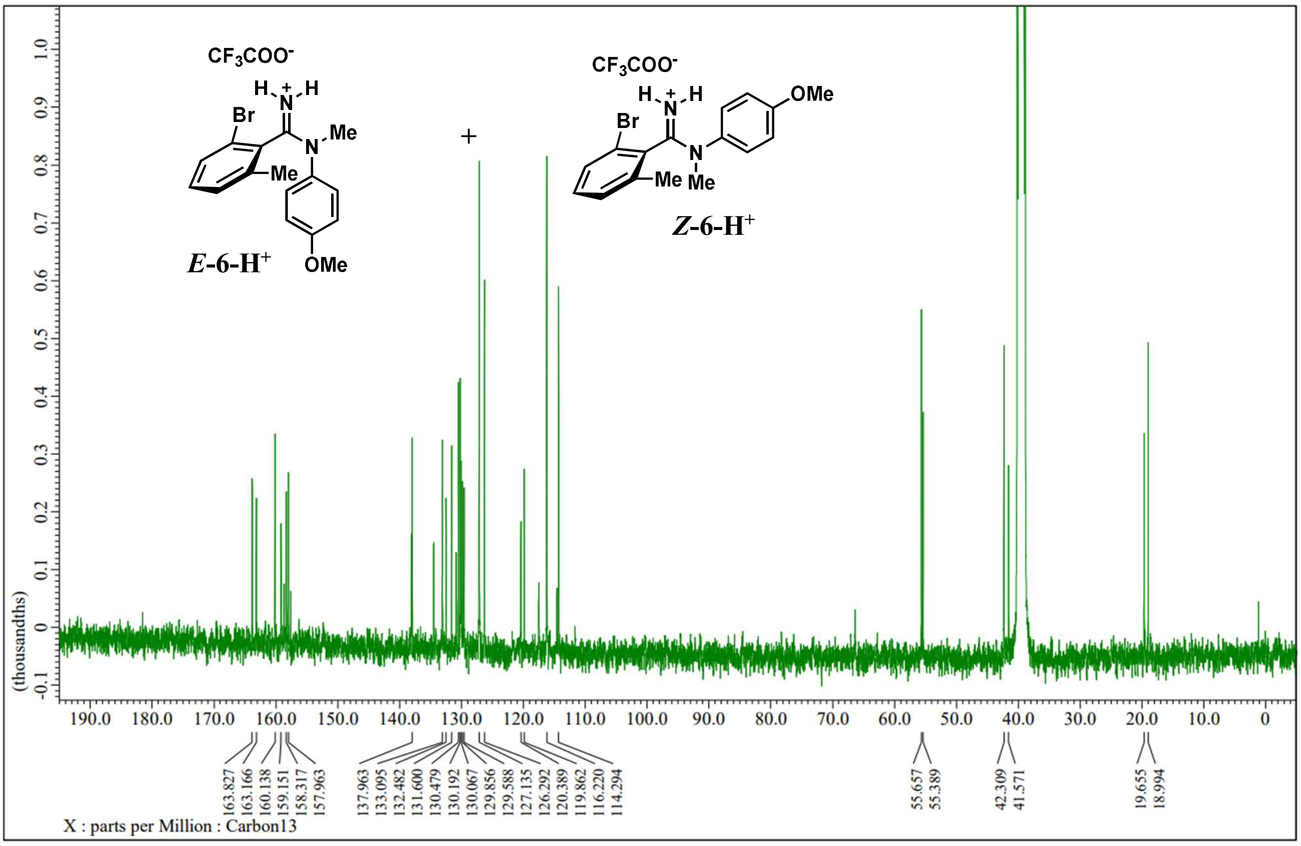


**Figure S9.22.** $^{13}$C NMR spectrum of **6** (DMSO-$d_6$, 100 MHz, 25 ˚C)

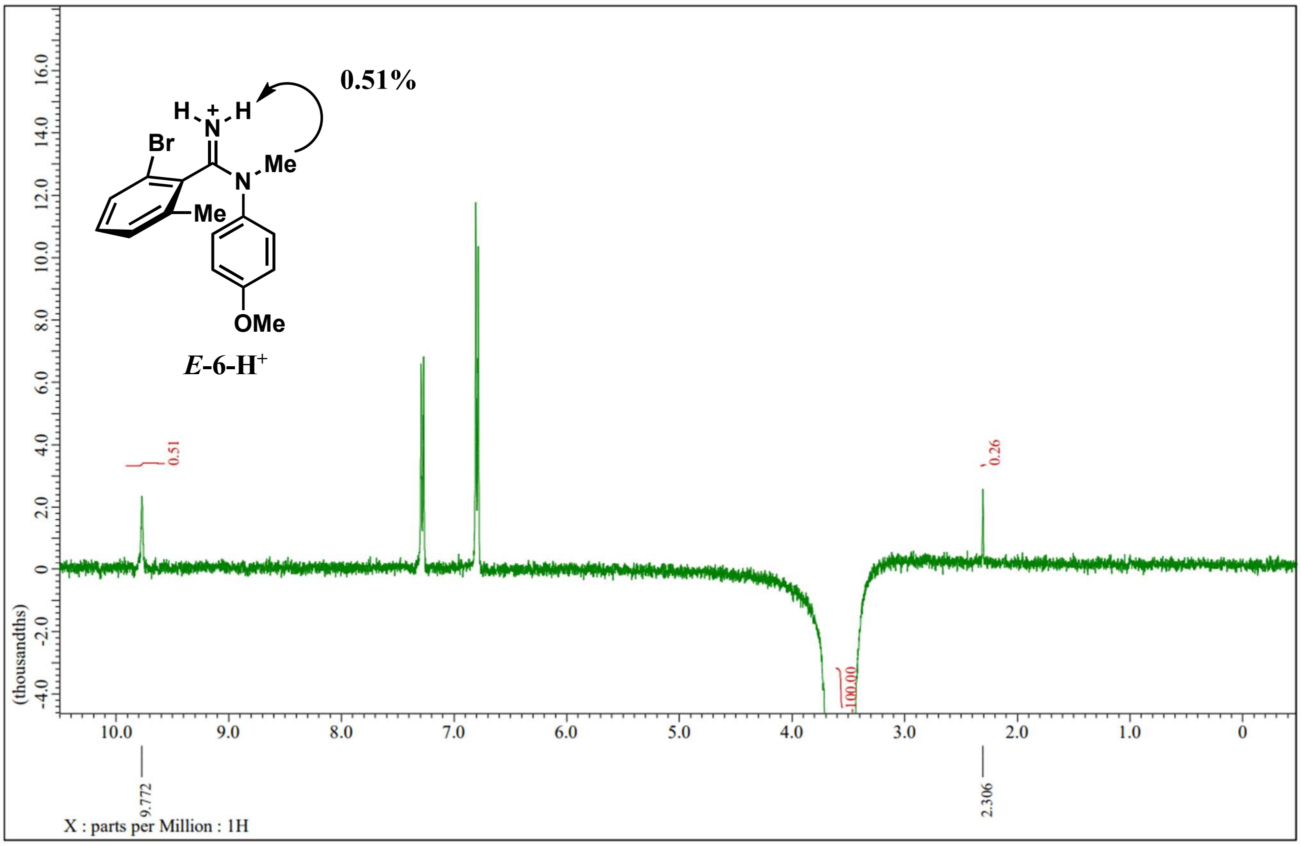


**Figure S9.23.** NOE spectrum of ***E*-6** (DMSO-$d_6$, 400 MHz, 25 ˚C)

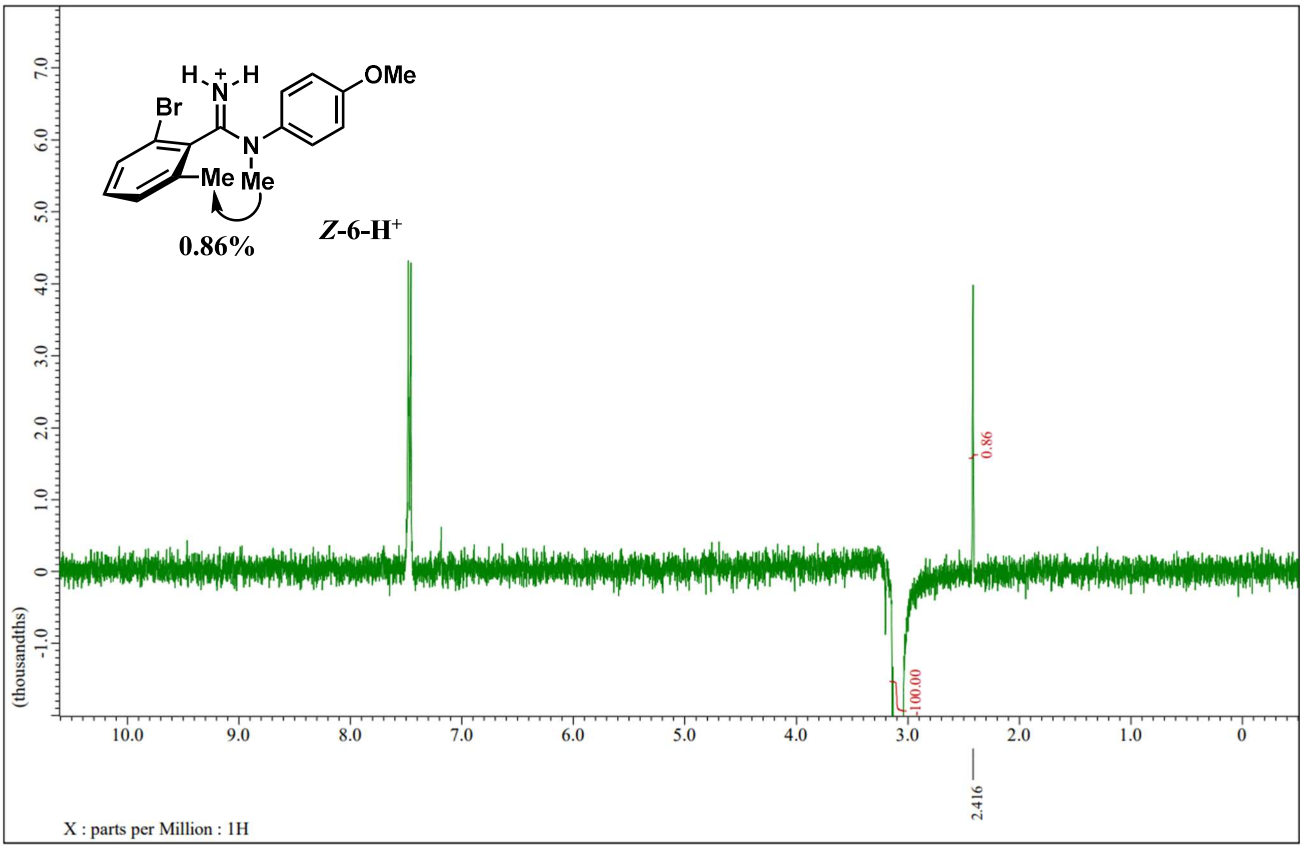


**Figure S9.24.** NOE spectrum of ***Z*-6** (DMSO-$d_6$, 400 MHz, 25 ˚C)

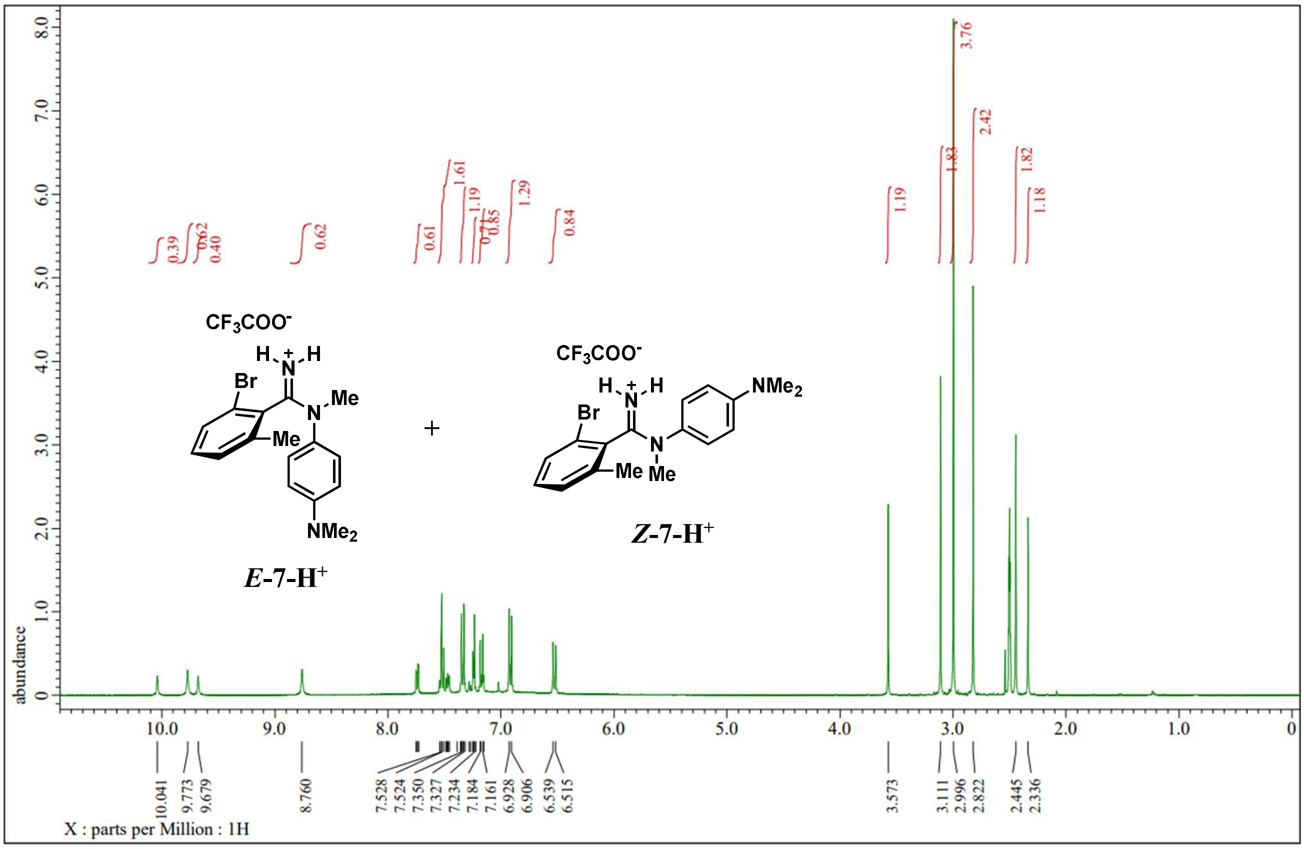


**Figure S9.25.** $^1$H NMR spectrum of **7** (DMSO-$d_6$, 400 MHz, 25 ˚C)

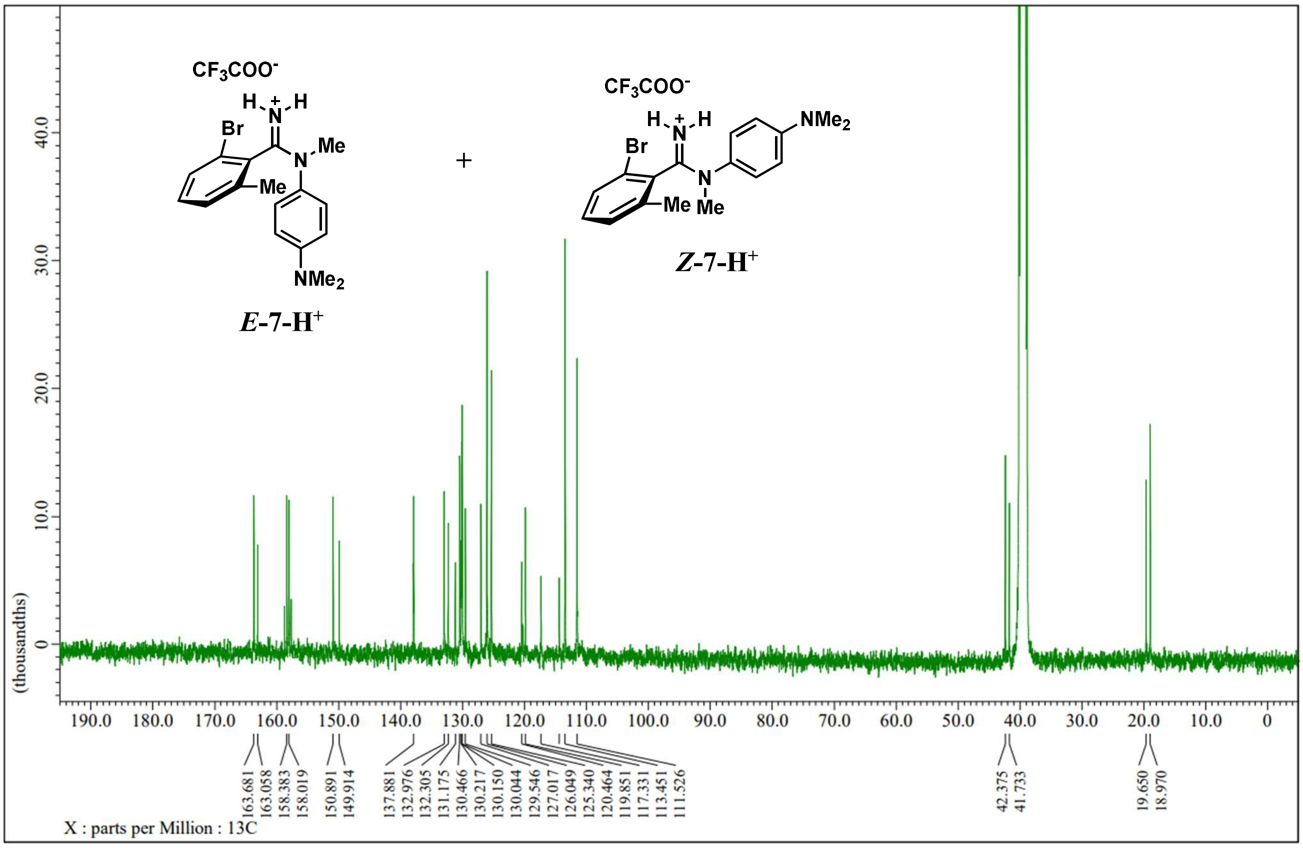


**Figure S9.26.** $^{13}$C NMR spectrum of **7** (DMSO-$d_6$, 100 MHz, 25 ˚C)

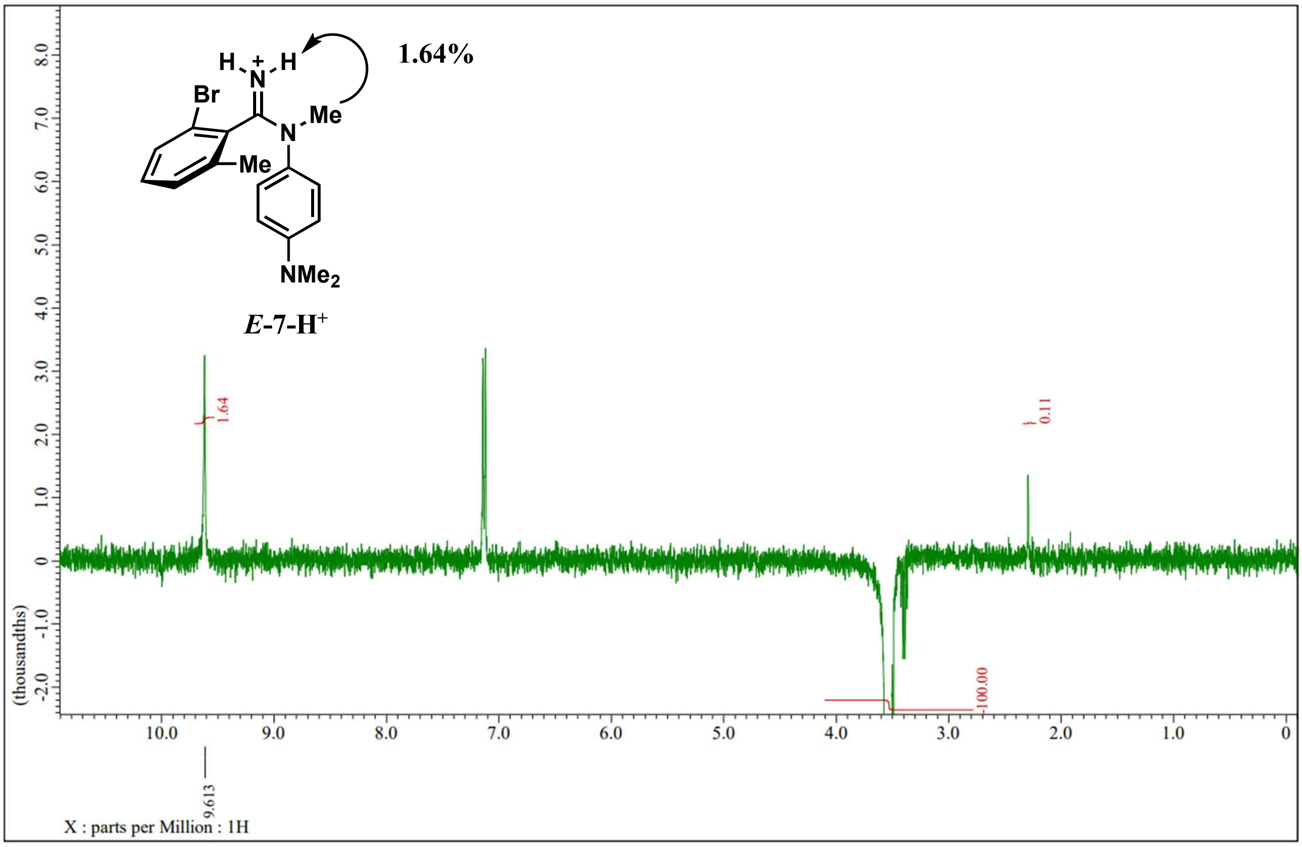


**Figure S9.27.** NOE spectrum of ***E*-7** (DMSO-$d_6$, 400 MHz, 25 ˚C)

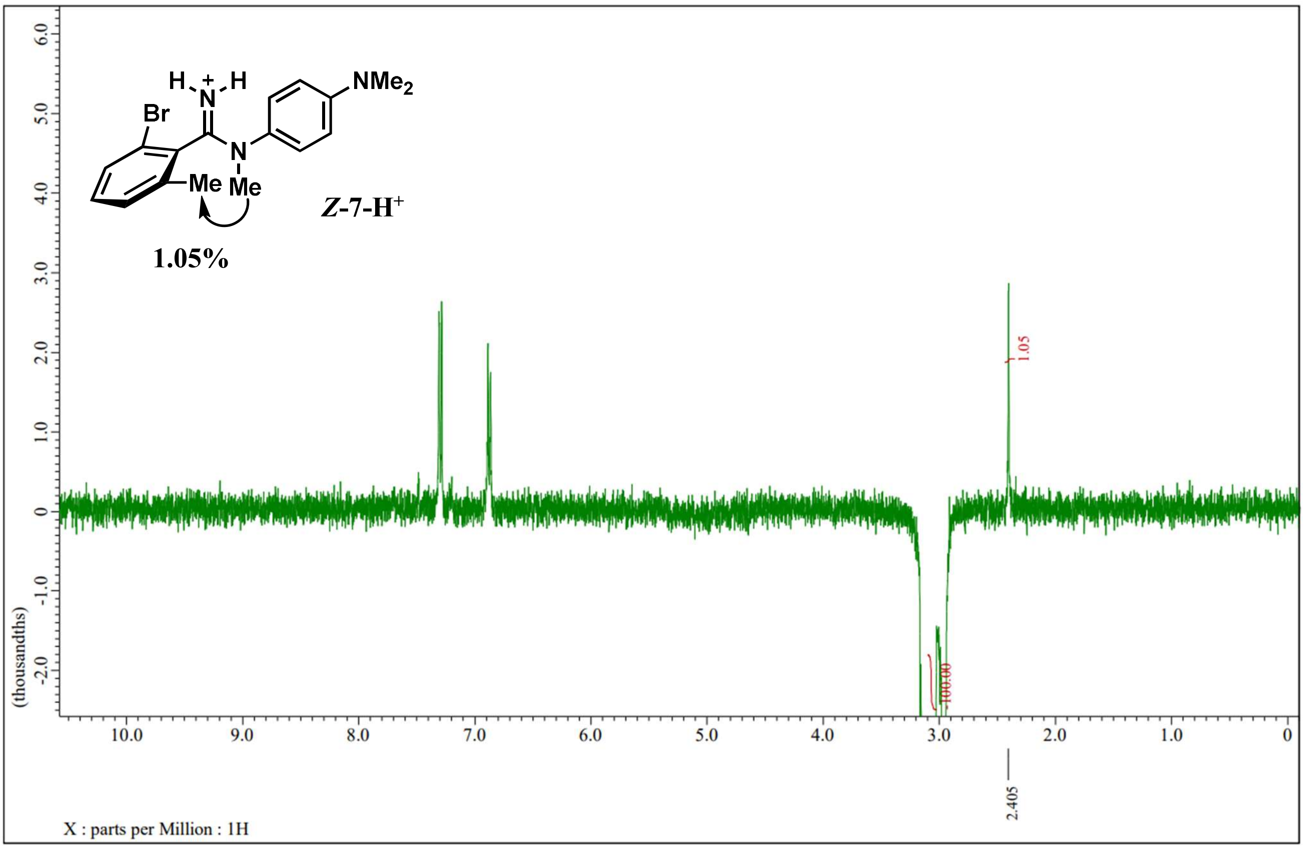


**Figure S9.28.** NOE spectrum of ***Z*-7** (DMSO-$d_6$, 400 MHz, 25 ˚C)

## 10. LR-MS and HR-MS charts of the synthesized compounds

### 2-Bromo-*N*,*N*-diethyl-6-methylbenzimidamide (1)

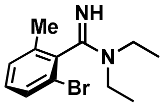


Sample No. : C:\Xcalibur\...\0418\250078_23KIM1-84_pn
Instrument : Exactive Plus
Mobile phase solvent : MeOH
Operator name : yamamoto hiroshi
Sample solvent : MeOH
Date : 04/18/25 13:17:42
Instrumental method : C:\Xcalibur\methods\ESI_100ul\S60_100ul_mz100_1500pn.meth
Instrumental Analysis Division, Global Facility Center, Creative Research Institution, Hokkaido University

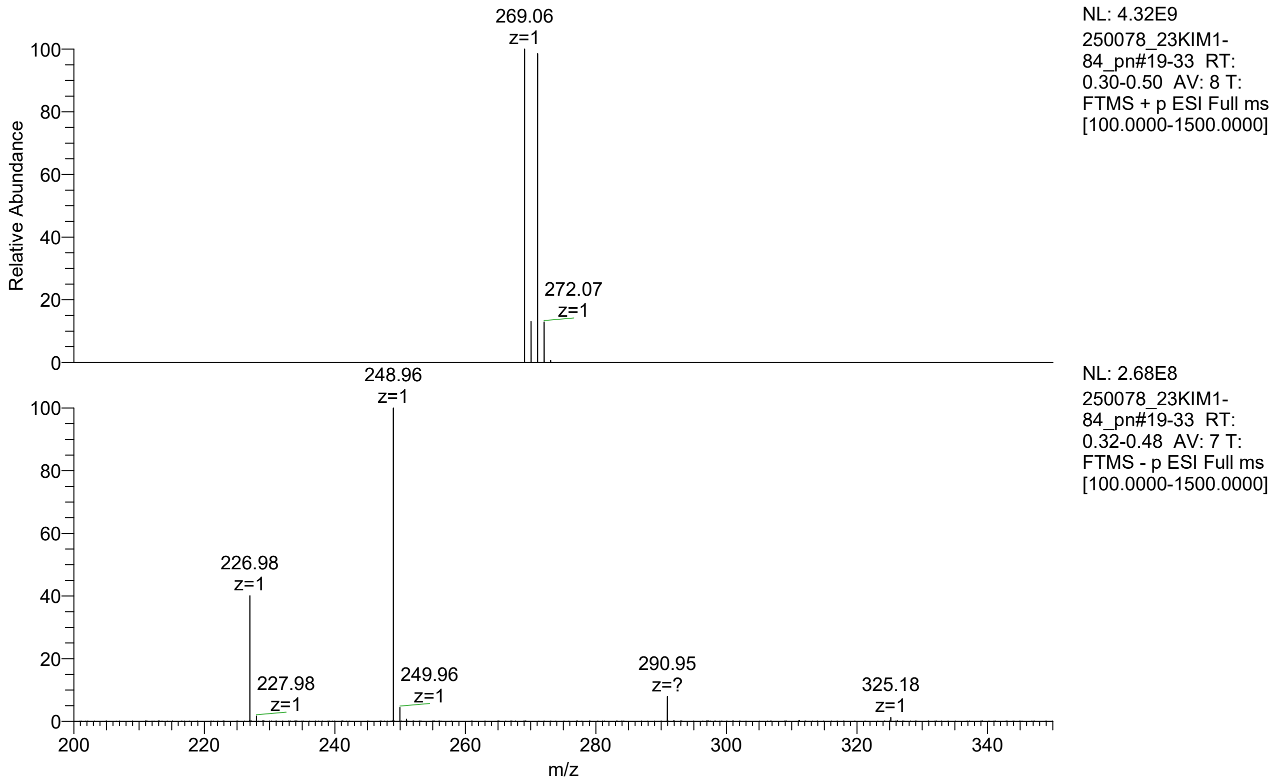


**Figure S10.1**. LR-MS spectra of compound **1**

Sample No. : C:\Xcalibur\...\0418\250078_23KIM1-84_pn    Instrument : Exactive Plus    Mobile phase solvent : MeOH
Operator name : yamamoto hiroshi    Sample solvent : MeOH
Date : 04/18/25 13:17:42
Instrumental method : C:\Xcalibur\methods\ESI_100ul\S60_100ul_mz100_1500pn.meth
Instrumental Analysis Division, Global Facility Center, Creative Research Institution, Hokkaido University

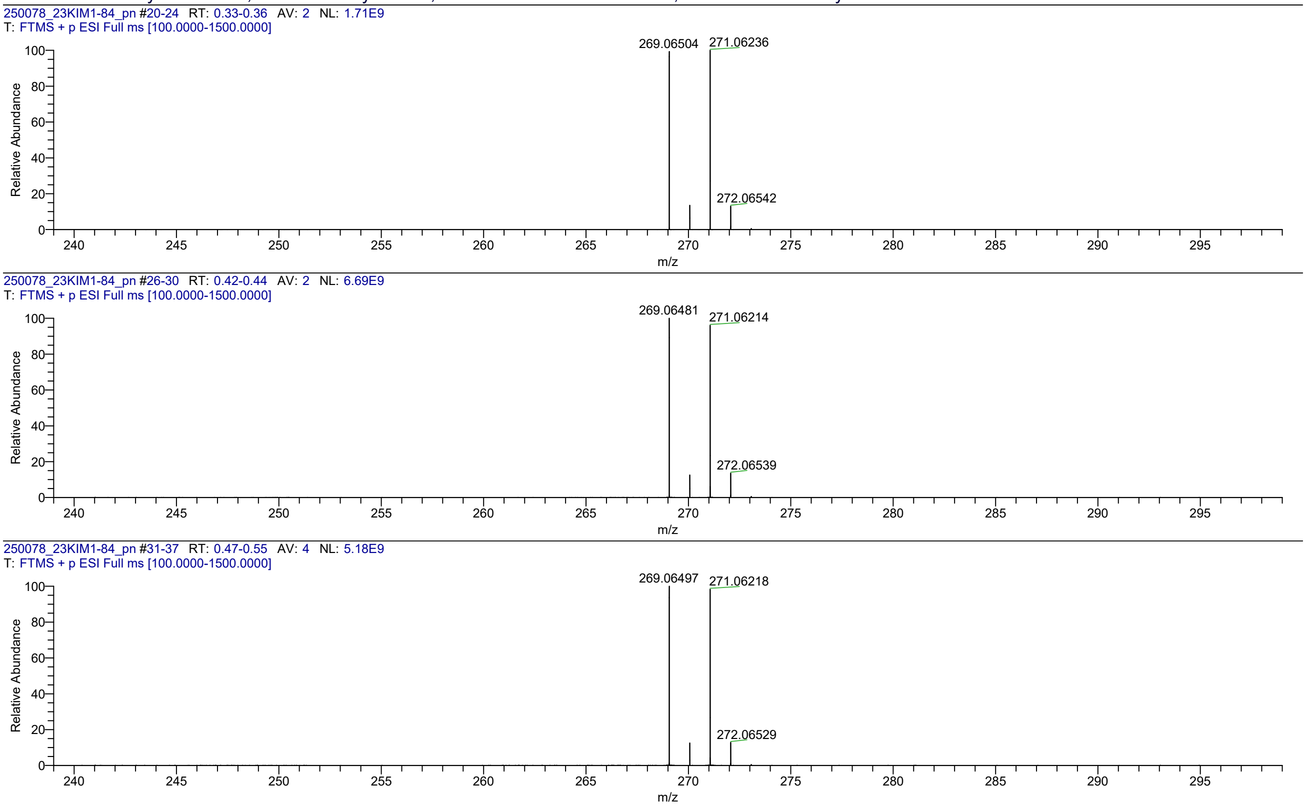


Elemental composition search on mass 269.06

m/z= 264.06-274.06

| Isotope | Min | Max |
|---|---|---|
| N-14 | 0 | 3 |
| O-16 | 0 | 3 |
| C-12 | 0 | 20 |
| H-1 | 0 | 25 |
| Na-23 | 0 | 0 |
| Br-79 | 1 | 1 |

Charge 1
Mass tolerance 5.00 ppm
Nitrogen rule not used
RDB equiv -1.00-100.00
max results 100

| m/z | Theo. Mass | Delta (ppm) | RDB equiv. | Composition |
|---|---|---|---|---|
| 269.06481 | 269.06479 | 0.08 | 4.5 | $C_{12}H_{18}N_2Br$ |

**Figure S10.2**. HR-MS spectra of compound **1**

## *N*-Benzyl-2-bromo-*N*,6-dimethylbenzimidamide (2)

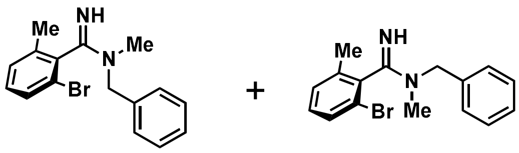


(*E*) (*Z*)

Sample No. : C:\Xcalibur\...\BG_250072_23KIM1-7_pn Instrument : Exactive Plus Mobile phase solvent : MeOH
Operator name : yamamoto hiroshi Sample solvent : MeOH
Date : 04/18/25 14:47:51
Instrumental method : C:\Xcalibur\methods\ESI_100ul\S60_100ul_mz150_2000pn.meth
Instrumental Analysis Division, Global Facility Center, Creative Research Institution, Hokkaido University

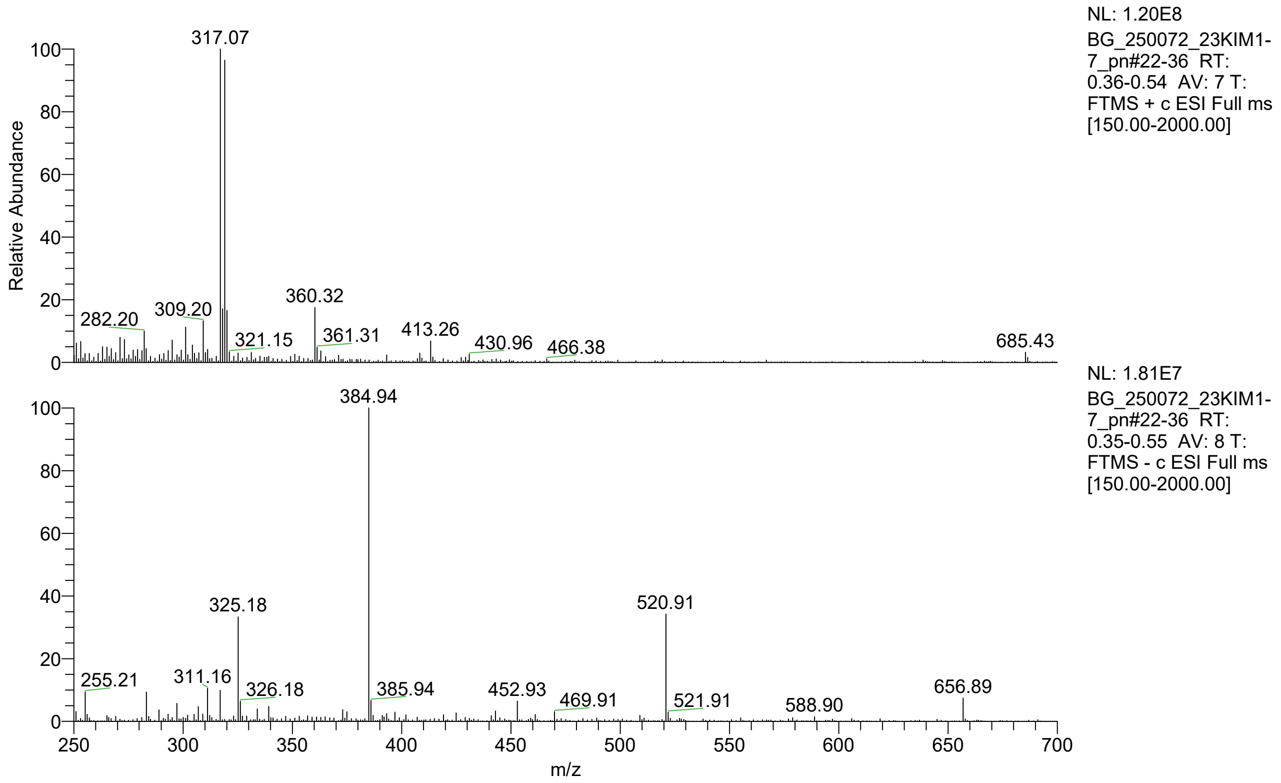


**Figure S10.3**. LR-MS spectra of compound **2**

Sample No. : C:\Xcalibur\...\0418\250072_23KIM1-7_pn Instrument : Exactive Plus Mobile phase solvent : MeOH
Operator name : yamamoto hiroshi Sample solvent : MeOH MeOH
Date : 04/18/25 12:47:05
Instrumental method : C:\Xcalibur\methods\ESI_100ul\S60_100ul_mz150_2000pn.meth
Instrumental Analysis Division, Global Facility Center, Creative Research Institution, Hokkaido University

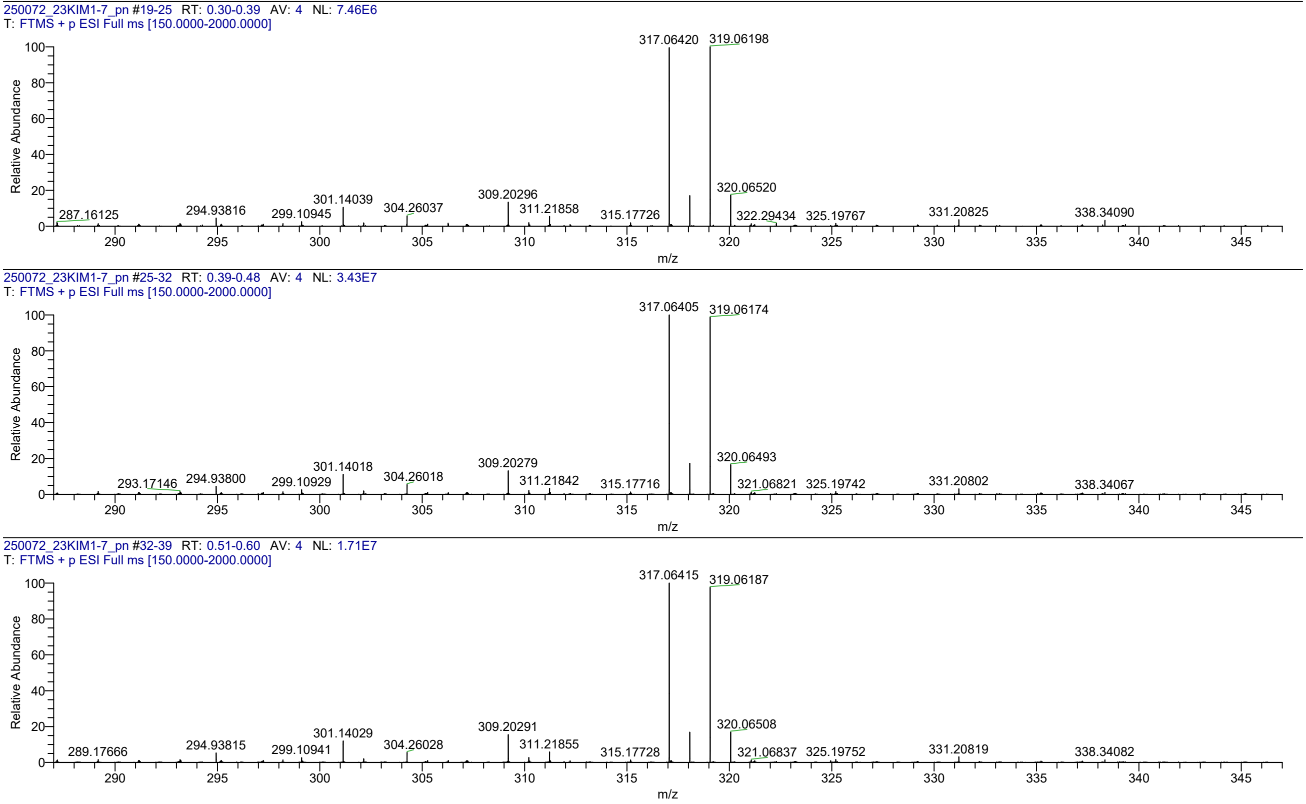


Elemental composition search on mass 317.06

m/z= 312.06-322.06

| Isotope | Min | Max |
|---|---|---|
| N-14 | 0 | 3 |
| O-16 | 0 | 0 |
| C-12 | 0 | 20 |
| H-1 | 0 | 20 |
| Na-23 | 0 | 1 |
| Br-79 | 1 | 1 |

Charge 1
Mass tolerance 5.00 ppm
Nitrogen rule not used
RDB equiv -1.00-100.00
max results 100

| m/z | Theo. Mass | Delta (ppm) | RDB equiv. | Composition |
|---|---|---|---|---|
| 317.06405 | 317.06479 | -2.33 | 8.5 | $C_{16}H_{18}N_2Br$ |

**Figure S10.4**. HR-MS spectra of compound **2**

**2-Bromo-*N*-(4-chlorophenyl)-*N*,6-dimethylbenzimidamide (3)**

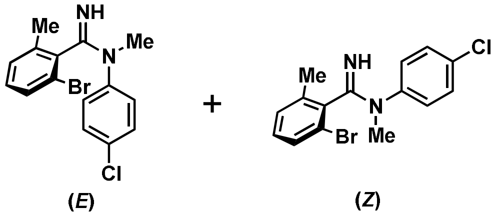


Sample No. : C:\Xcalibur\...\BG_250074_23KIM1-38_pn Instrument : Exactive Plus Mobile phase solvent : MeOH
Operator name : yamamoto hiroshi Sample solvent : MeOH
Date : 04/18/25 15:03:42
Instrumental method : C:\Xcalibur\methods\ESI_100ul\S60_100ul_mz150_2000pn.meth
Instrumental Analysis Division, Global Facility Center, Creative Research Institution, Hokkaido University

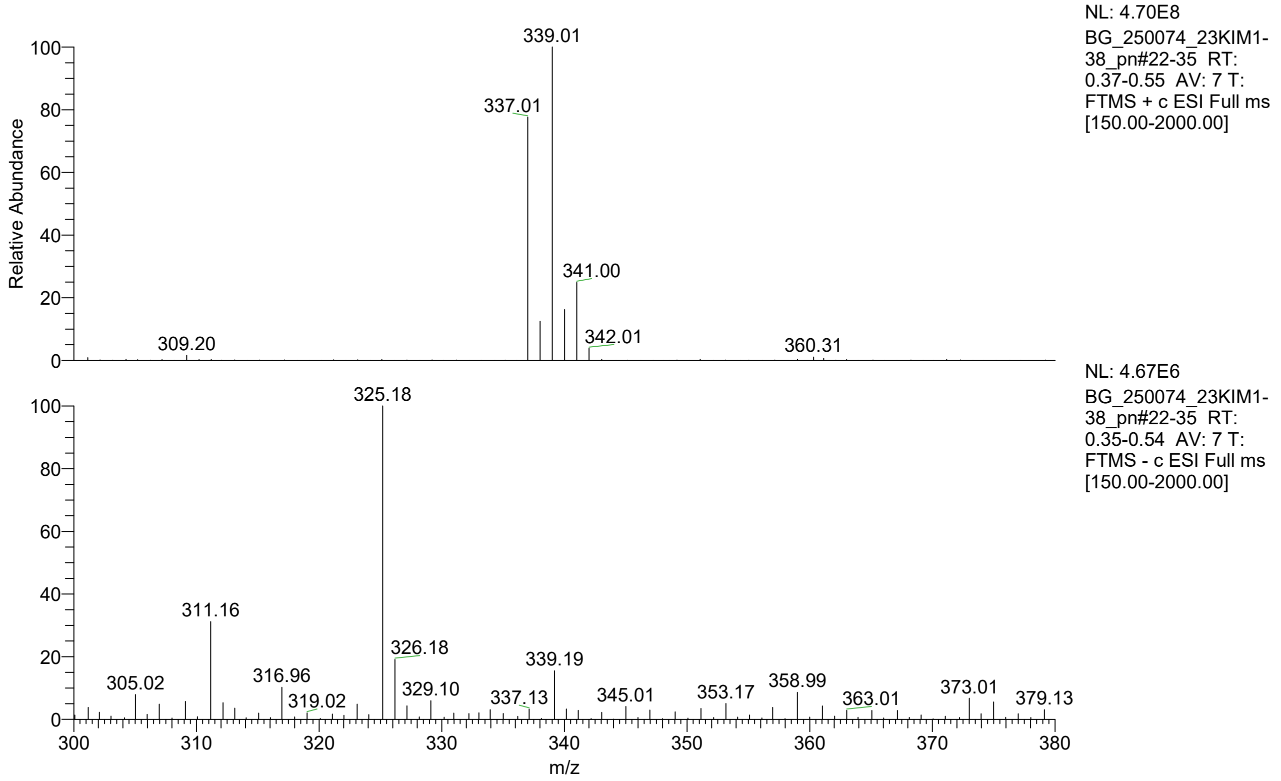


**Figure S10.5**. LR-MS spectra of compound **3**

Sample No. : C:\Xcalibur\...\0418\250074_23KIM1-38_pn
Instrument : Exactive Plus
Mobile phase solvent : MeOH
Operator name : yamamoto hiroshi
Sample solvent : MeOH MeOH
Date : 04/18/25 12:57:07
Instrumental method : C:\Xcalibur\methods\ESI_100ul\S60_100ul_mz150_2000pn.meth
Instrumental Analysis Division, Global Facility Center, Creative Research Institution, Hokkaido University

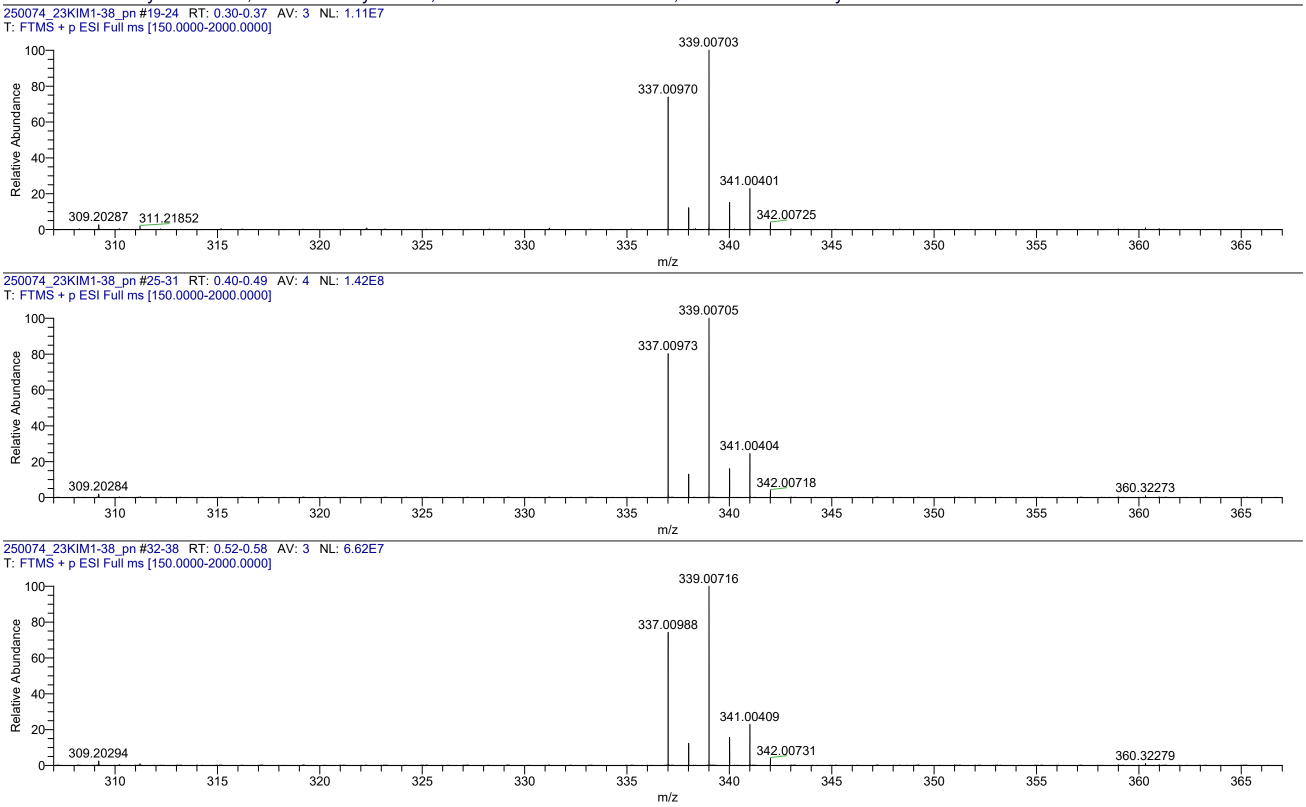


Elemental composition search on mass 337.01

m/z= 332.01-342.01

| Isotope | Min | Max |
|---|---|---|
| N-14 | 0 | 3 |
| O-16 | 0 | 0 |
| C-12 | 0 | 20 |
| H-1 | 0 | 20 |
| Na-23 | 0 | 1 |
| Br-79 | 1 | 1 |
| Cl-35 | 0 | 3 |

Charge 1
Mass tolerance 5.00 ppm
Nitrogen rule not used
RDB equiv -1.00-100.00
max results 100

| m/z | Theo. Mass | Delta (ppm) | RDB equiv. | Composition |
|---|---|---|---|---|
| 337.00973 | 337.01017 | -1.29 | 8.5 | $C_{15}H_{15}N_2BrCl$ |

**Figure S10.6**. HR-MS spectra of compound **3**

**2-Bromo-*N*,6-dimethyl-*N*-phenylbenzimidamide (4)**

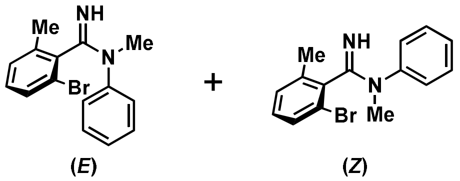


Sample No. : C:\Xcalibur\...\BG_250073_23KIM1-28_pn Instrument : Exactive Plus Mobile phase solvent : MeOH
Operator name : yamamoto hiroshi Sample solvent : MeOH
Date : 04/18/25 14:57:24
Instrumental method : C:\Xcalibur\methods\ESI_100ul\S60_100ul_mz150_2000pn.meth
Instrumental Analysis Division, Global Facility Center, Creative Research Institution, Hokkaido University

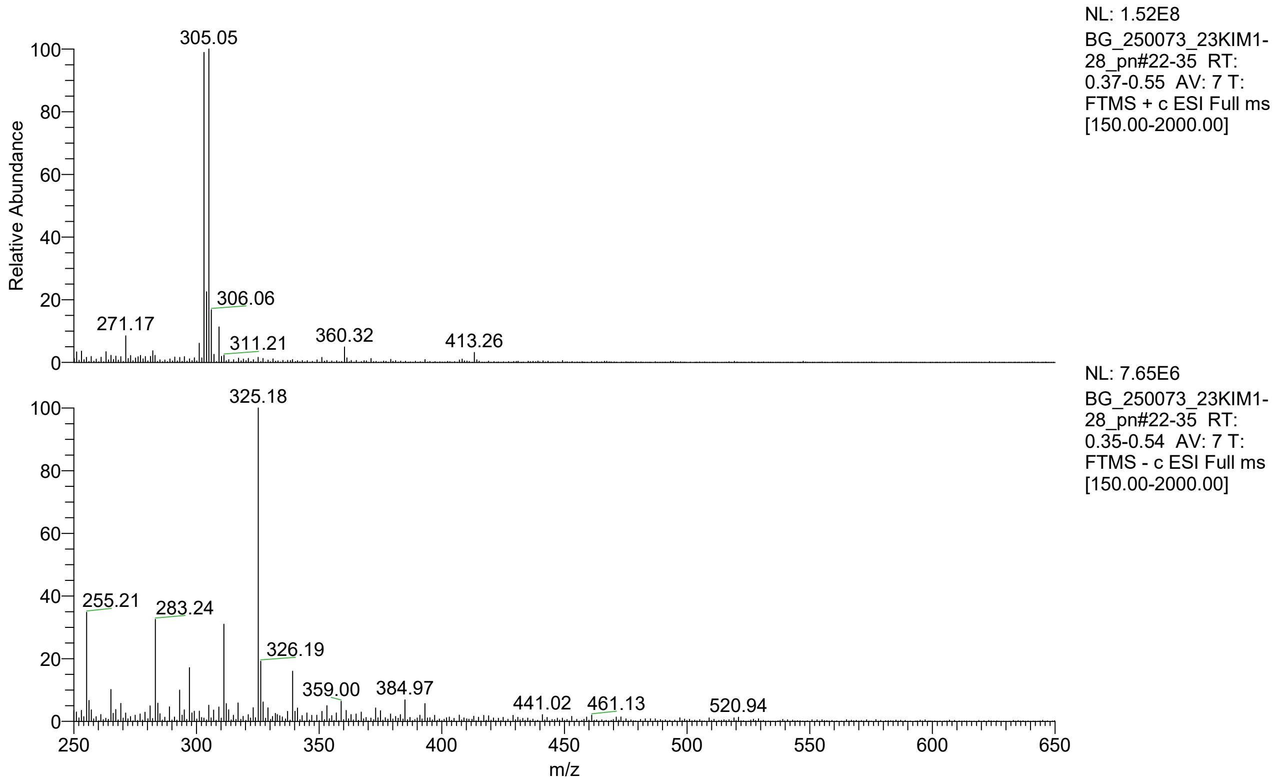


**Figure S10.7**. LR-MS spectra of compound **4**

Sample No. : C:\Xcalibur\...\0418\250073_23KIM1-28_pn Instrument : Exactive Plus Mobile phase solvent : MeOH
Operator name : yamamoto hiroshi Sample solvent : MeOH MeOH
Date : 04/18/25 12:52:06
Instrumental method : C:\Xcalibur\methods\ESI_100ul\S60_100ul_mz150_2000pn.meth
Instrumental Analysis Division, Global Facility Center, Creative Research Institution, Hokkaido University

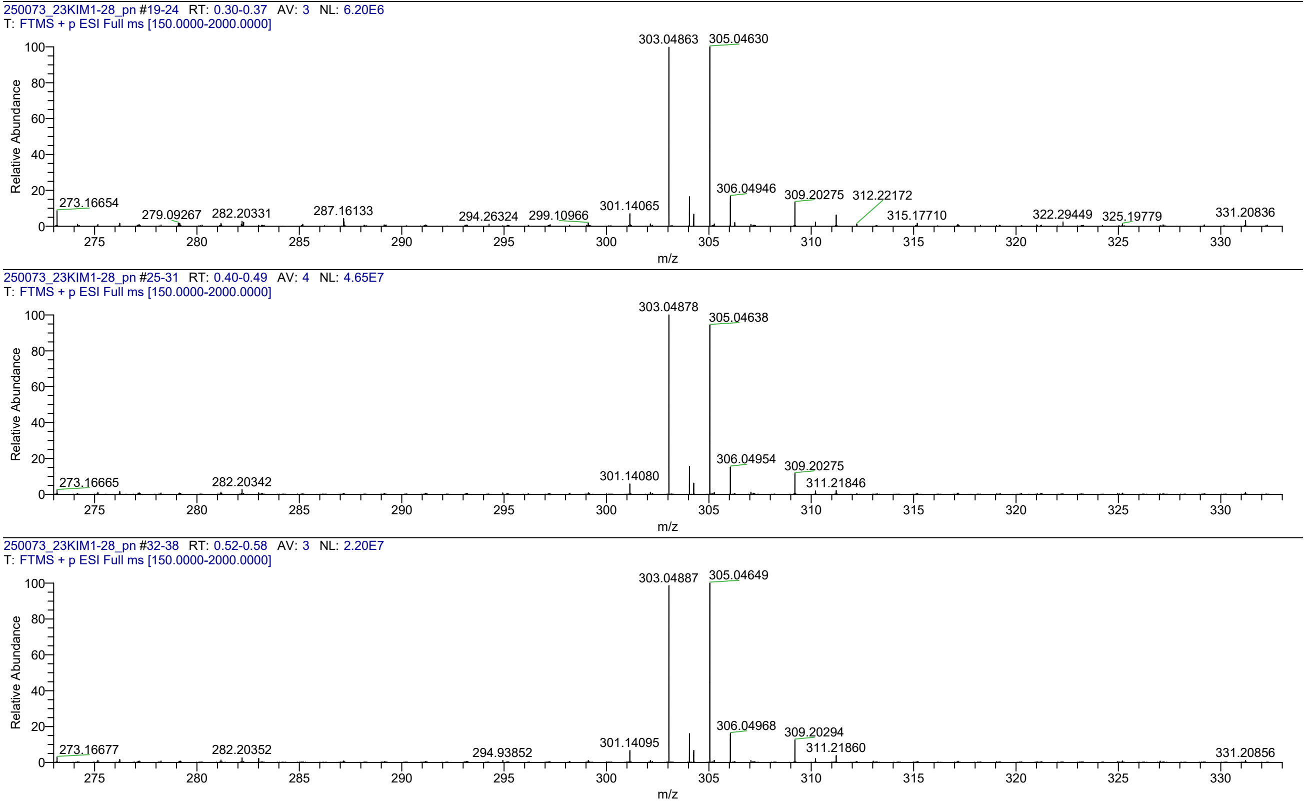


```
Elemental composition search on mass 303.05

m/z= 298.05-308.05
Isotope  Min   Max
N-14       0     3
O-16       0     0
C-12       0    20
H-1        0    20
Na-23      0     1
Br-79      1     1
Charge 1
Mass tolerance 5.00 ppm
Nitrogen rule not used
RDB equiv -1.00-100.00
max results 100
```

| m/z | Theo. Mass | Delta (ppm) | RDB equiv. | Composition |
|---|---|---|---|---|
| 303.04878 | 303.04914 | -1.18 | 8.5 | $C_{15}H_{16}N_2Br$ |

**Figure S10.8**. HR-MS spectra of compound **4**

**2-Bromo-*N*,6-dimethyl-*N*-(4-tolyl)benzimidamide (5)**

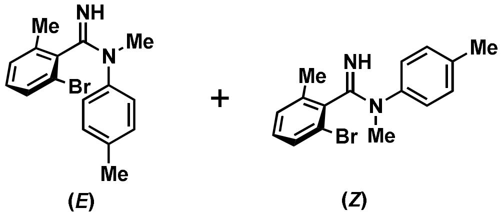


Sample No. : C:\Xcalibur\...\BG_250075_23KIM1-46_pn
Instrument : Exactive Plus
Mobile phase solvent : MeOH
Operator name : yamamoto hiroshi
Sample solvent : MeOH
Date : 04/18/25 15:11:30
Instrumental method : C:\Xcalibur\methods\ESI_100ul\S60_100ul_mz150_2000pn.meth
Instrumental Analysis Division, Global Facility Center, Creative Research Institution, Hokkaido University

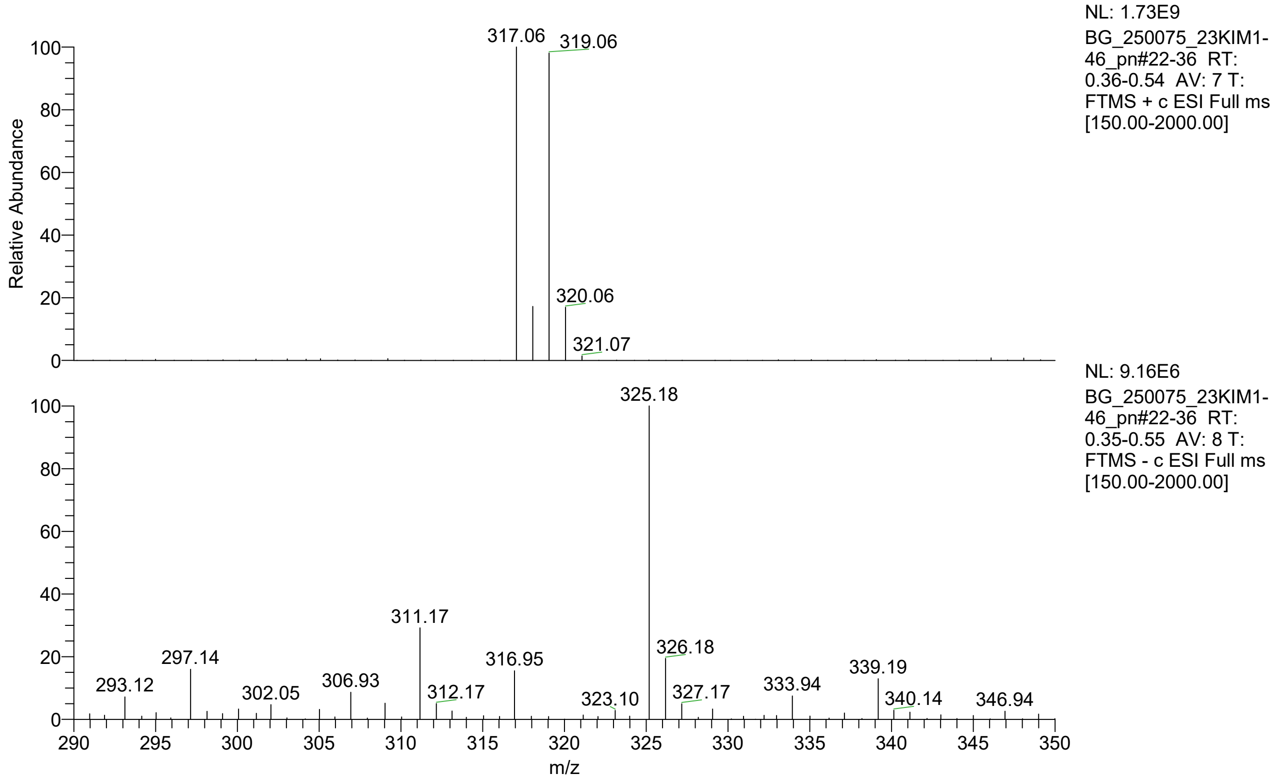


**Figure S10.9**. LR-MS spectra of compound **5**

Sample No. : C:\Xcalibur\...\0418\250075_23KIM1-46_pn Instrument : Exactive Plus Mobile phase solvent : MeOH
Operator name : yamamoto hiroshi Sample solvent : MeOH MeOH
Date : 04/18/25 13:02:08
Instrumental method : C:\Xcalibur\methods\ESI_100ul\S60_100ul_mz150_2000pn.meth
Instrumental Analysis Division, Global Facility Center, Creative Research Institution, Hokkaido University

250075_23KIM1-46_pn #19-25 RT: 0.30-0.39 AV: 4 NL: 1.01E8
T: FTMS + p ESI Full ms [150.0000-2000.0000]

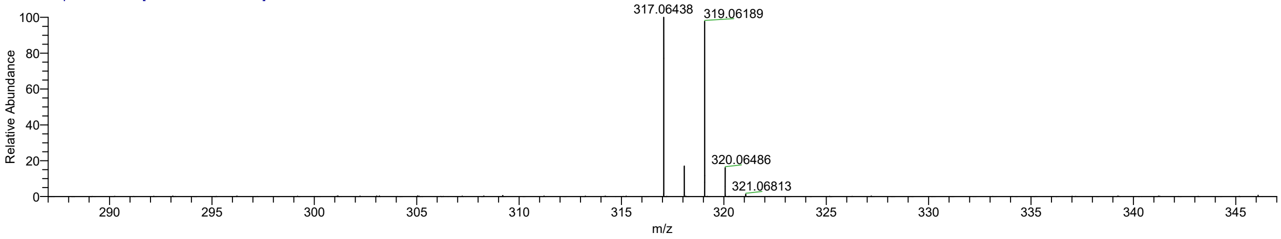


250075_23KIM1-46_pn #25-32 RT: 0.39-0.48 AV: 4 NL: 5.19E8
T: FTMS + p ESI Full ms [150.0000-2000.0000]

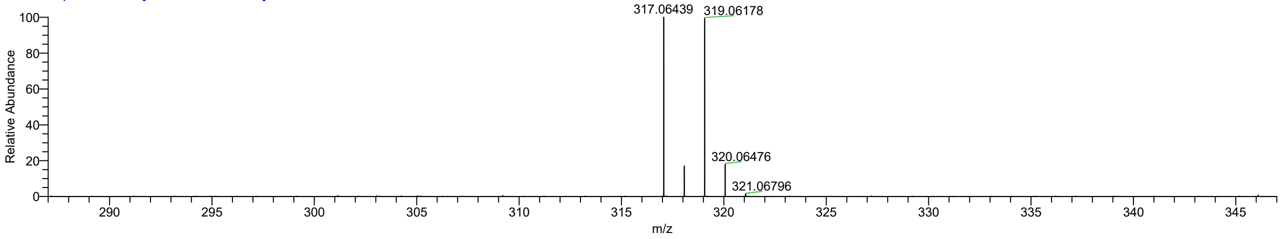


250075_23KIM1-46_pn #32-39 RT: 0.51-0.60 AV: 4 NL: 2.29E8
T: FTMS + p ESI Full ms [150.0000-2000.0000]

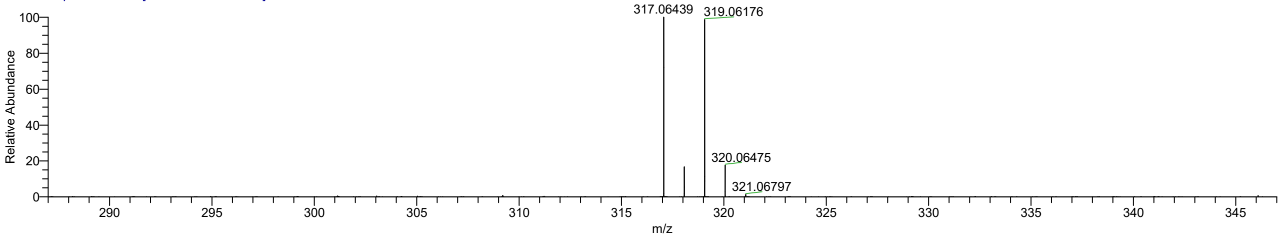


Elemental composition search on mass 317.06

m/z= 312.06-322.06

| Isotope | Min | Max |
|---|---|---|
| N-14 | 0 | 3 |
| O-16 | 0 | 0 |
| C-12 | 0 | 20 |
| H-1 | 0 | 20 |
| Na-23 | 0 | 1 |
| Br-79 | 1 | 1 |

Charge 1
Mass tolerance 5.00 ppm
Nitrogen rule not used
RDB equiv -1.00-100.00
max results 100

| m/z | Theo. Mass | Delta (ppm) | RDB equiv. | Composition |
|---|---|---|---|---|
| 317.06439 | 317.06479 | -1.25 | 8.5 | $C_{16}H_{18}N_2Br$ |

**Figure S10.10**. HR-MS spectra of compound **5**

**2-Bromo-N-(4-methoxyphenyl)-N,6-dimethylbenzimidamide (6)**

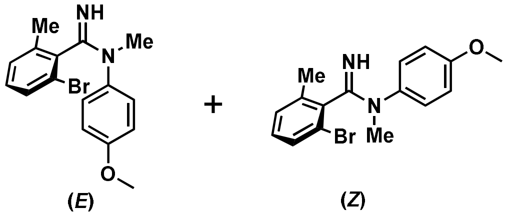


Sample No. : C:\Xcalibur\...\BG_250076_23KIM1-47_pn
Instrument : Exactive Plus
Mobile phase solvent : MeOH
Operator name : yamamoto hiroshi
Sample solvent : MeOH
Date : 04/18/25 15:18:45
Instrumental method : C:\Xcalibur\methods\ESI_100ul\S60_100ul_mz150_2000pn.meth
Instrumental Analysis Division, Global Facility Center, Creative Research Institution, Hokkaido University

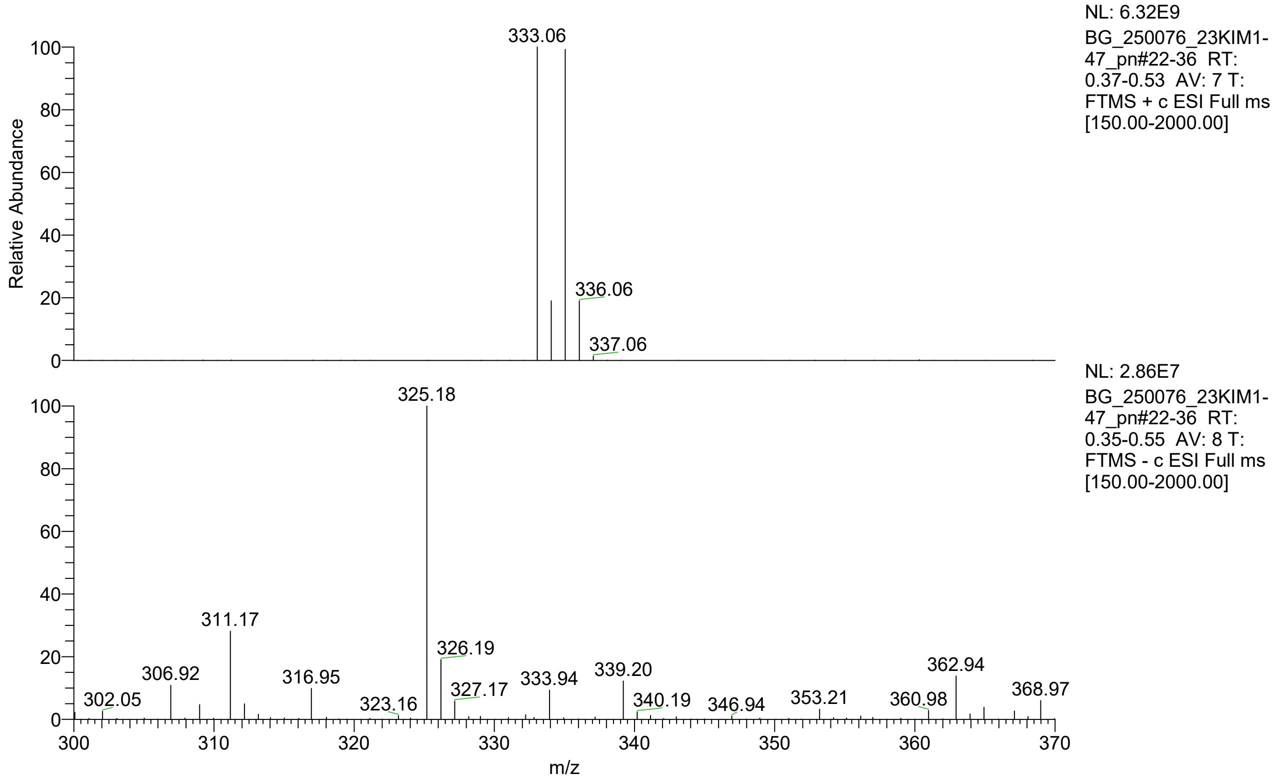


**Figure S10.11**. LR-MS spectra of compound **6**

Sample No. : C:\Xcalibur\...\0418\250076_23KIM1-47_pn Instrument : Exactive Plus Mobile phase solvent : MeOH
Operator name : yamamoto hiroshi Sample solvent : MeOH MeOH
Date : 04/18/25 13:07:12
Instrumental method : C:\Xcalibur\methods\ESI_100ul\S60_100ul_mz150_2000pn.meth
Instrumental Analysis Division, Global Facility Center, Creative Research Institution, Hokkaido University

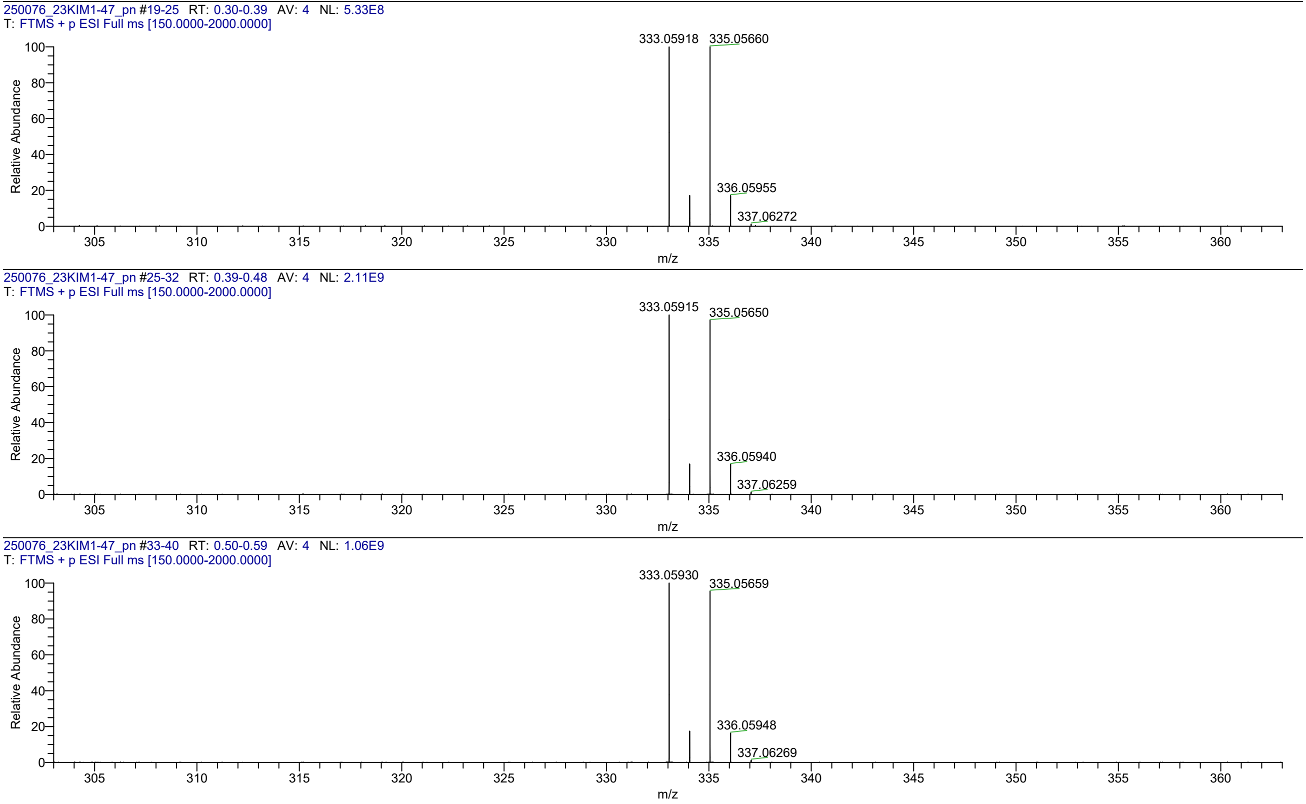


Elemental composition search on mass 333.06

m/z= 328.06-338.06

| Isotope | Min | Max |
|---|---|---|
| N-14 | 0 | 3 |
| O-16 | 0 | 3 |
| C-12 | 0 | 20 |
| H-1 | 0 | 20 |
| Na-23 | 0 | 1 |
| Br-79 | 1 | 1 |

Charge 1
Mass tolerance 5.00 ppm
Nitrogen rule not used
RDB equiv -1.00-100.00
max results 100

| m/z | Theo. Mass | Delta (ppm) | RDB equiv. | Composition |
|---|---|---|---|---|
| 333.05915 | 333.05970 | -1.66 | 8.5 | $C_{16}H_{18}ON_2Br$ |

**Figure S10.12**. HR-MS spectra of compound **6**

**2-Bromo-*N*-(4-(dimethylamino)phenyl)-*N*,6-dimethylbenzimidamide (7)**

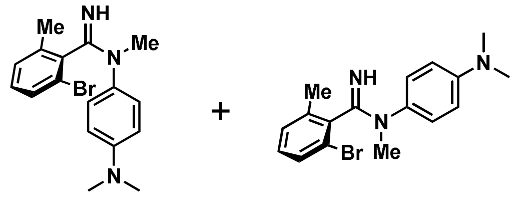


(*E*) (*Z*)

Sample No. : C:\Xcalibur\...\BG_250077_23KIM1-76_pn Instrument : Exactive Plus Mobile phase solvent : MeOH
Operator name : yamamoto hiroshi Sample solvent : MeOH
Date : 04/18/25 15:25:04
Instrumental method : C:\Xcalibur\methods\ESI_100ul\S60_100ul_mz150_2000pn.meth
Instrumental Analysis Division, Global Facility Center, Creative Research Institution, Hokkaido University

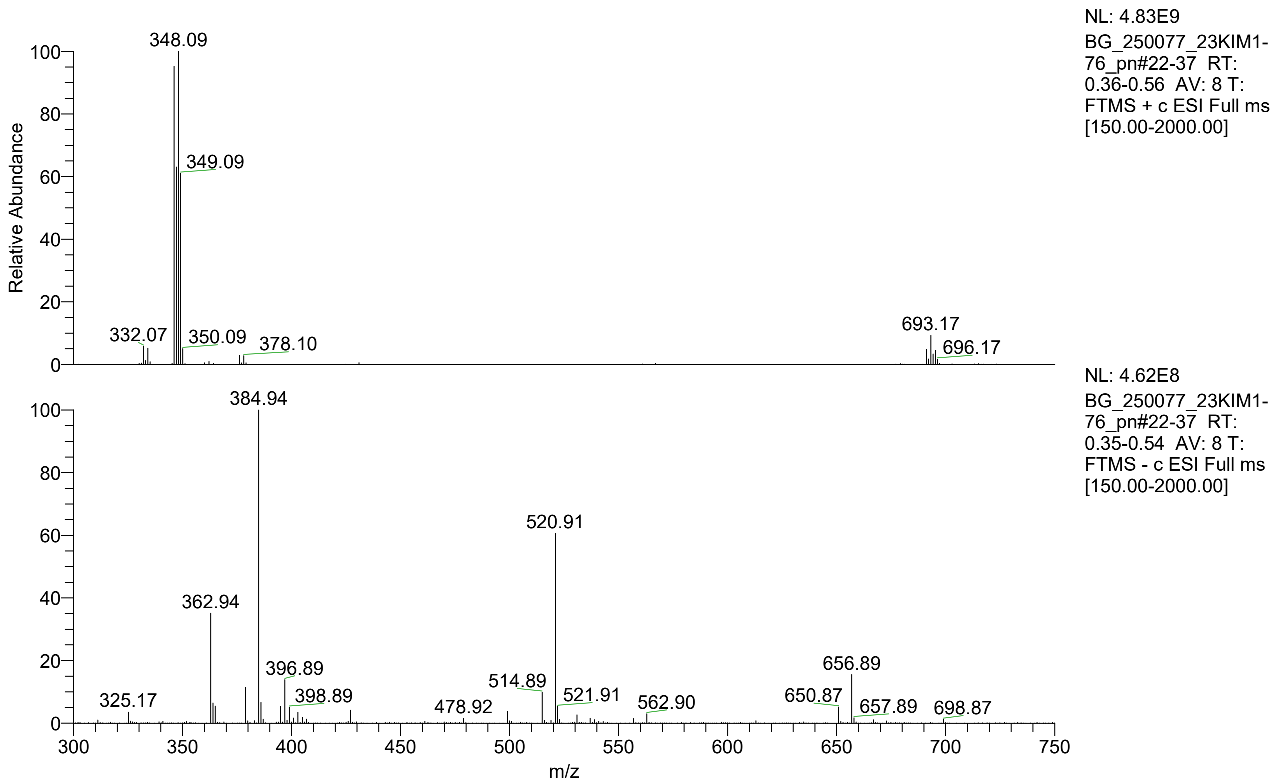


**Figure S10.13**. LR-MS spectra of compound **7**

Sample No. : C:\Xcalibur\...\0418\250077_23KIM1-76_pn
Operator name : yamamoto hiroshi
Date : 04/18/25 13:12:14
Instrumental method : C:\Xcalibur\methods\ESI_100ul\S60_100ul_mz150_2000pn.meth
Instrumental Analysis Division, Global Facility Center, Creative Research Institution, Hokkaido University

Instrument : Exactive Plus

Mobile phase solvent : MeOH
Sample solvent : MeOH MeOH

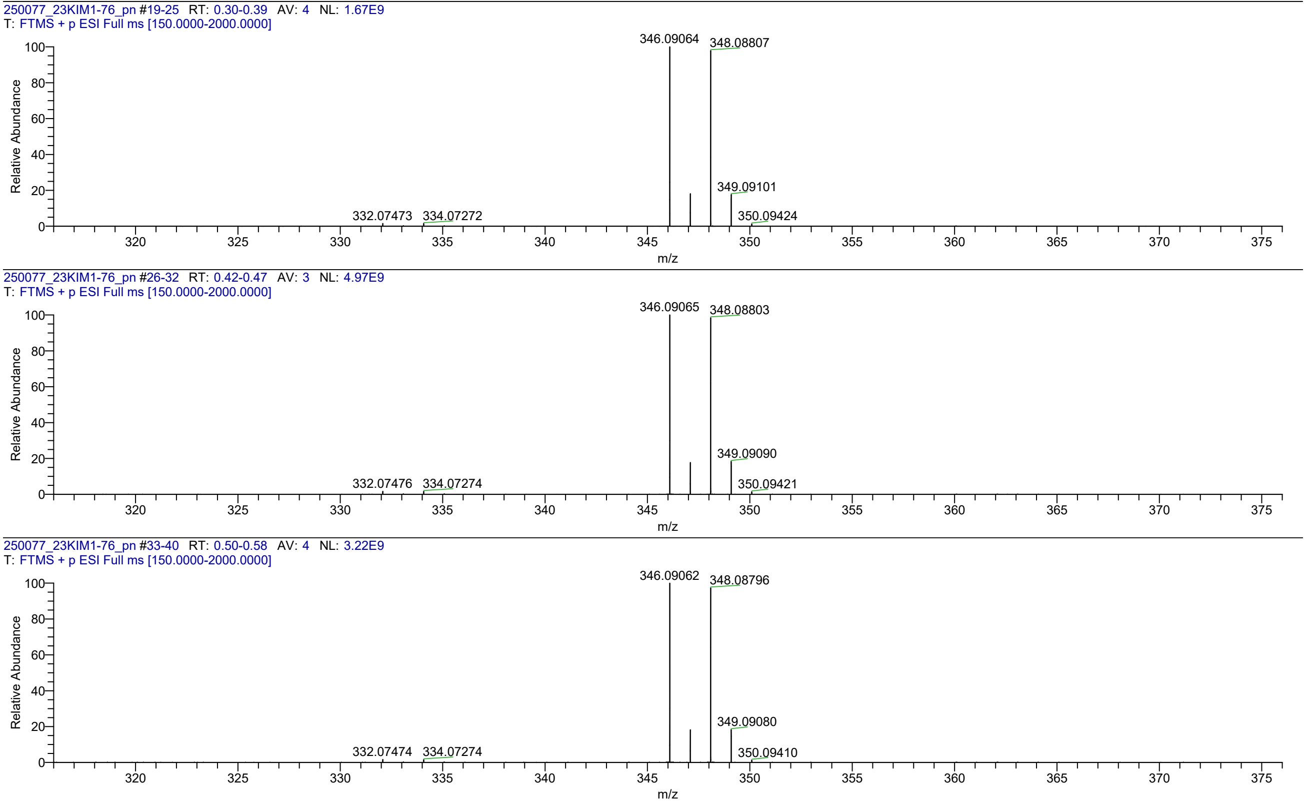


Elemental composition search on mass 346.09

m/z= 341.09-351.09

| Isotope | Min | Max |
|---|---|---|
| N-14 | 0 | 3 |
| O-16 | 0 | 3 |
| C-12 | 0 | 20 |
| H-1 | 0 | 25 |
| Na-23 | 0 | 0 |
| Br-79 | 1 | 1 |

Charge 1
Mass tolerance 5.00 ppm
Nitrogen rule not used
RDB equiv -1.00-100.00
max results 100

| m/z | Theo. Mass | Delta (ppm) | RDB equiv. | Composition |
|---|---|---|---|---|
| 346.09065 | 346.09134 | -1.98 | 8.5 | $C_{17}H_{21}N_3Br$ |

**Figure S10.14**. HR-MS spectra of compound **7**

## 11. Reference

1. Du, B.; Jiang, X.; Sun, P. J. Org. Chem. 2013, 78, 2786–2791.

2. Gaussian 16, Revision C.01, Frisch, M. J.; Trucks, G. W.; Schlegel, H. B.; Scuseria, G. E.; Robb, M. A.; Cheeseman, J. R.; Scalmani, G.; Barone, V.; Petersson, G. A.; Nakatsuji, H.; Li, X.; Caricato, M.; Marenich, A. V.; Bloino, J.; Janesko, B. G.; Gomperts, R.; Mennucci, B.; Hratchian, H. P.; Ortiz, J. V.; Izmaylov, A. F.; Sonnenberg, J. L.; Williams-Young, D.; Ding, F.; Lipparini, F.; Egidi, F.; Goings, J.; Peng, B.; Petrone, A.; Henderson, T.; Ranasinghe, D.; Zakrzewski, V. G.; Gao, J.; Rega, N.; Zheng, G.; Liang, W.; Hada, M.; Ehara, M.; Toyota, K.; Fukuda, R.; Hasegawa, J.; Ishida, M.; Nakajima, T.; Honda, Y.; Kitao, O.; Nakai, H.; Vreven, T.; Throssell, K.; Montgomery, J. A., Jr.; Peralta, J. E.; Ogliaro, F.; Bearpark, M. J.; Heyd, J. J.; Brothers, E. N.; Kudin, K. N.; Staroverov, V. N.; Keith, T. A.; Kobayashi, R.; Normand, J.; Raghavachari, K.; Rendell, A. P.; Burant, J. C.; Iyengar, S. S.; Tomasi, J.; Cossi, M.; Millam, J. M.; Klene, M.; Adamo, C.; Cammi, R.; Ochterski, J. W.; Martin, R. L.; Morokuma, K.; Farkas, O.; Foresman, J. B.; Fox, D. J. Gaussian, Inc., Wallingford CT (2016).
